# Parallel reaction monitoring revealed tolerance to drought proteins in weedy rice (*Oryza sativa *f. *spontanea*)

**DOI:** 10.1038/s41598-020-69739-9

**Published:** 2020-07-31

**Authors:** Bing Han, Xiaoding Ma, Di Cui, Leiyue Geng, Guilan Cao, Hui Zhang, Longzhi Han

**Affiliations:** 1 Institute of Crop Sciences, Chinese Academy of Agricultural Sciences, Beijing, 100081 China; 2 Coastal Agriculture Institute, Hebei Academy of Agricultural and Forestry Sciences, Tangshan, 063299 China

**Keywords:** Biochemistry, Biological techniques, Chemical biology, Genetics

## Abstract

Drought is a complicated abiotic stress factor with severe effects on rice growth and production. Weedy rice is a valuable genetic resource that possesses a strong capacity for drought tolerance, cold tolerance, and salt tolerance, and is an excellent material for studying rice tolerance. Here, according to comprehensive tolerance to drought index D, accession WR16 was selected based on strong drought tolerance among 133 studied weedy red rice germplasms. WR16 was compared with *Oryza sativa* ssp. *Japonica*. cv. IAPAR-9, a reference genotype originating from Brazil. In addition, accession WR24 was classified as moderately tolerant to drought accessions. Transcriptomic and proteomic analyses were combined to identify 38 co-upregulated proteins related to drought tolerance, and targeted parallel reaction monitoring (PRM) was used to precisely quantify and verify nine proteins in the complex backgrounds. Result showed that six proteins were significantly (Fisher's exact P value < 0.05) related to drought tolerance in accessions WR16 and WR24. Among them, OS09T0478300-01, OS09T0530300-01, and OS01T0800500-01 formed a combined defense system to respond to drought stress in weedy rice. Results of these studies provide comprehensive information for precisely identifying and verifying tolerance to drought proteins and lay a solid theoretical foundation for research on drought tolerance mechanisms.

## Introduction

Rice is a staple food crop around the globe, and drought has become a main obstacle affecting production and yield stability of rice. Compared to cultivated rice, weedy rice (*Oryza sativa* f. *spontanea*) has some desirable features, such as early flowering time, fitness advantage, heavy seed shattering, biotic and abiotic stress resistance, and intense seed dormancy. Weedy rice has become a valuable genetic resource for improving rice traits including drought tolerance, cold tolerance, salt tolerance, and water use efficiency^1^. Three accessions—two cultivated rice and one weedy rice—were selected in a previous study to analyze genomic variation, which is crucial to elucidate the genetic basis associated with some important traits^2^. Identifying drought tolerance germplasm in weedy rice accessions through new, precise methods is critical for further study of the genetic and molecular mechanism associated with drought tolerant traits. Combining omics analysis will greatly shorten the time of identifying germplasms and genes and accelerate the breeding process of tolerance to drought accession^3^. These genetic resources function directly in facilitating discovery of functional variants and enhancing our ability to control and utilize weedy rice for rice improvement.

Drought tolerance proteins are the primary component of the defense system in plant, and are a powerful weapon to combat adverse environments. Changes in the amounts of some proteins in the stress-response system reveal the effect of expression of some genes on protein synthesis and its possible mechanism under different conditions, such as drought, low temperature, and salt stress. In recent years, protein quantification techniques have been developed, in particular parallel reaction monitoring (PRM) technology. Through the PRM technique, proteins have received specific focus, reducing their complexity, maximizing their dynamics, and enhancing the validity of measurements for some low abundance proteins. Moreover, multiple lines of evidence show that changes in the amounts of some proteins are closely related to environmental change^6^. Many genes and proteins related to drought tolerance have been excavated and verified. For example, the *OsNAC9* gene enhanced drought tolerance through changing root architecture under stress conditions^11^. The *Dro1* gene adjusted the root architecture and increased the rice yield during drought stress^12^. The *DWA1* gene is essential in regulating drought tolerance by wax deposition under stress conditions^13^. Expression of *HARDY* can improve water use efficiency in rice^14^. A rice transcription factor gene *OsGRAS23* responds to drought stress by regulating the expression of some stress-induced genes^15^.

Mass spectrometry (MS), a powerful and systematic analysis tool, opened a new era in studying proteins based on proteomics in complicated biological backgrounds^4^. The development of MS-based high throughput proteomics has successfully gone through two phases, “shotgun” proteomics and “targeted” proteomics. The former depends on MS data and can detect thousands of proteins in per analysis. The latter can quantify targeted proteins reproducibly across a large number of samples, in targeted proteomics, the first generation was selected reaction monitoring (SRM), which has mainly been used in clinical biomarker discovery^5^. PRM is a new targeted method based on high resolution and accurate mass spectrometers, which permits simultaneous monitoring of all peptides accompanied by full tandem MS (MS/MS) scanning. PRM can be more selective and reliably quantify each target protein^6–9^. Protein degradation was evaluated in plant signaling using full mass range scans combined with data-dependent and targeted MS/MS^10^. Proteomic analysis by iTRAQ-PRM provides novel insights into the molecular mechanism response of plant to *B. tabaci* in Pepper^6^.

In this study, we applied phenotype identification of drought tolerance and evaluated the capacity of drought tolerance among 133 weedy rice accessions. The strong tolerance to drought accession WR16 was verified, and using the WR16 accession, combined transcriptomics and iTRAQ analysis were carried out and 38 proteins were verified as upregulated protein. Subsequently, nine proteins were further studied for validation using PRM technology and six proteins had a significant correlation with drought tolerance in weedy rice. This study can help us explain the network of protein changes related to drought tolerance capacity, and enhance the understanding of drought tolerance mechanisms, provide insights for improving drought tolerance in rice breeding.

## Results

### Identifying the strong drought tolerance accession WR16

**Figure 1:**
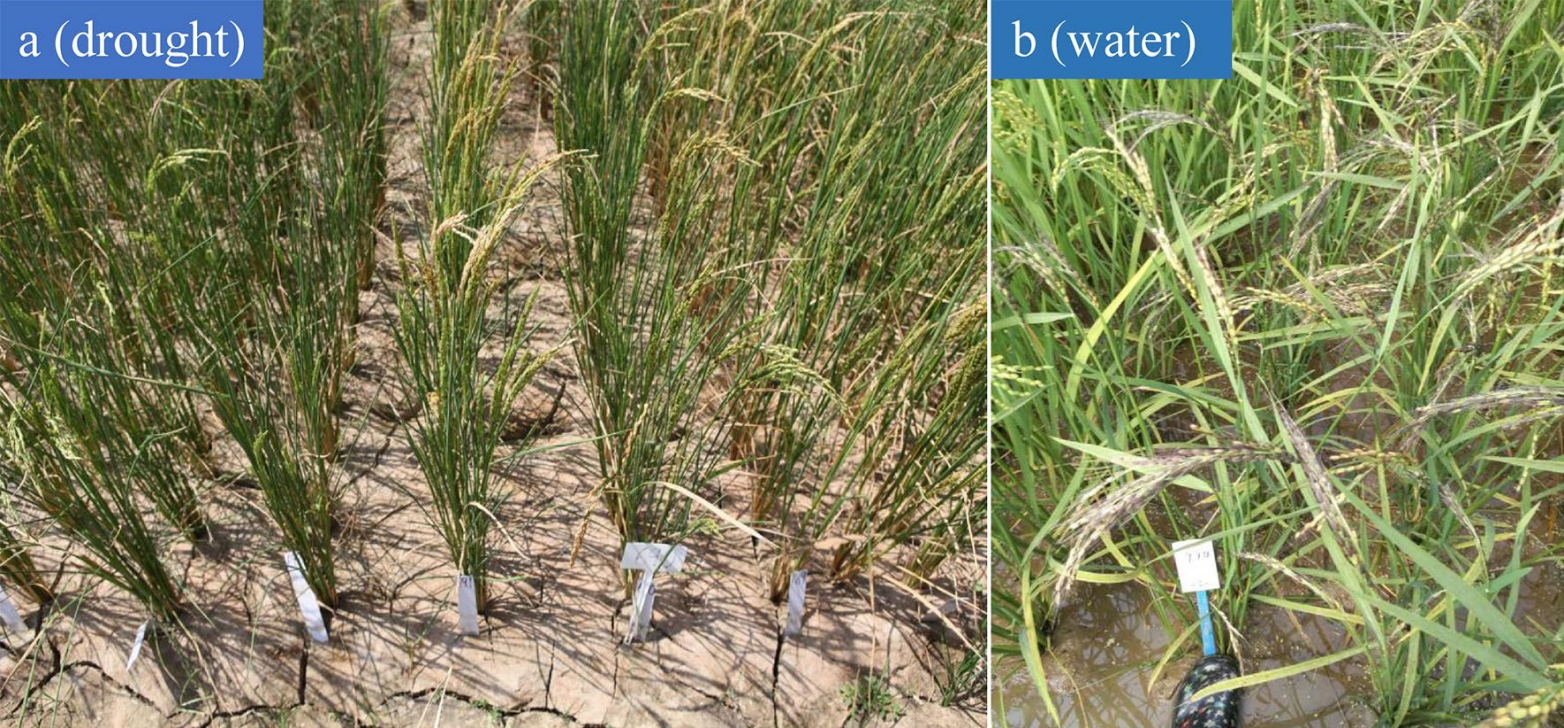
The overall effect picture of drought-stress treatment in Hainan experiment fields. (**a**) The effect picture of drought stress in Hainan experiment fields. (**b**) The picture of water field (normal condition) in Hainan experiment fields.

The identification experiment of drought tolerance for 133 weedy rice accessions was constructed at the Nanbin farm in Sanya city of Hainan province, China, from January 25 to March 22, 2017, total consecutive 57 days, it is not rain in the region (data from the weather forecast of 2017 (Tables S1, S2). We applied phenotype data of ten drought tolerance indices (Table S3) and evaluated the drought tolerance among 133 weedy rice accessions (Fig. 1). PCA of ten drought tolerance indices resulted in four comprehensive indices CI (1), CI (2), CI (3), and CI (4) with contribution rates (CRs) of 29.756%, 25.895%, 16.170%, and 11.096%, respectively. The cumulative CR reached 82.917% (Table 1). Four comprehensive indices were calculated for each accession (Table S4).

**Table 1:** Coefficients of comprehensive index CI(x) and contribution rate.

CI	PH	SL	TN	PL	CC	NC	WR1-1	WR1-2	WR2-1	WR2-2	CR (%)
CI (1)	− 0.266	− 0.248	− 0.171	− 0.09	0.608	0.637	0.74	0.642	0.743	0.721	29.756
CI (2)	0.894	0.902	0.761	− 0.09	− 0.151	− 0.146	0.123	0.387	0.233	0.354	25.895
CI (3)	0.24	0.208	0.218	0.145	0.757	0.733	− 0.198	− 0.378	− 0.146	− 0.366	16.17
CI (4)	− 0.047	− 0.071	0.011	0.485	0.091	0.077	− 0.49	0.485	− 0.453	0.414	11.096

According to formula (1): the subordinate function values *μ*(*x*) for each sample was calculated (Table S5), and the subordinate function values reflect the drought tolerance of each accession. According to formula (2): the weight function *w*_*i*_ was calculated and represents the relative importance for the *i*th comprehensive index, the comprehensive weights for the four comprehensive indices were 0.3589, 0.3123, 0.1951, and 0.1338, respectively, the total weight is 1. According to formula (3): the comprehensive evaluation value (D) of each accession for drought tolerance was calculated (Table S10). The D Value reflected the drought tolerance of each accession^28^.

According to D values (Table S10) for 133 weedy rice accessions, we classified all accessions into five types: very strong drought tolerance, when D > 0.8; strong drought tolerance, when 0.8 > D > 0.5; moderate drought tolerance, 0.5 > D > 0.4; relatively sensitive, 0.4 > D > 0.2; and sensitive, D < 0.2. WR157 had the maximum D **v**alue (0.9223), WR16 was 0.8956, IAPAR-9 was 0.8107 and WR24 was 0.4371. The D value for 74.6% of the accessions was greater than 0.5 (Table S10). These findings suggest that the weedy rice populations as whole have a relatively strong drought tolerance capacity. And the weedy rice accessions WR157 and WR16 have greater drought tolerance than the traditional drought tolerance control IAPAR-9, while WR24 has moderate drought tolerance than IAPAR-9. For further molecular study, accession WR16 was selected as the strong drought tolerance accession, and W24 was used as the moderate drought tolerance accession.

### Transcriptome and proteome crosstalk analysis based on WR16

**Figure 2:**
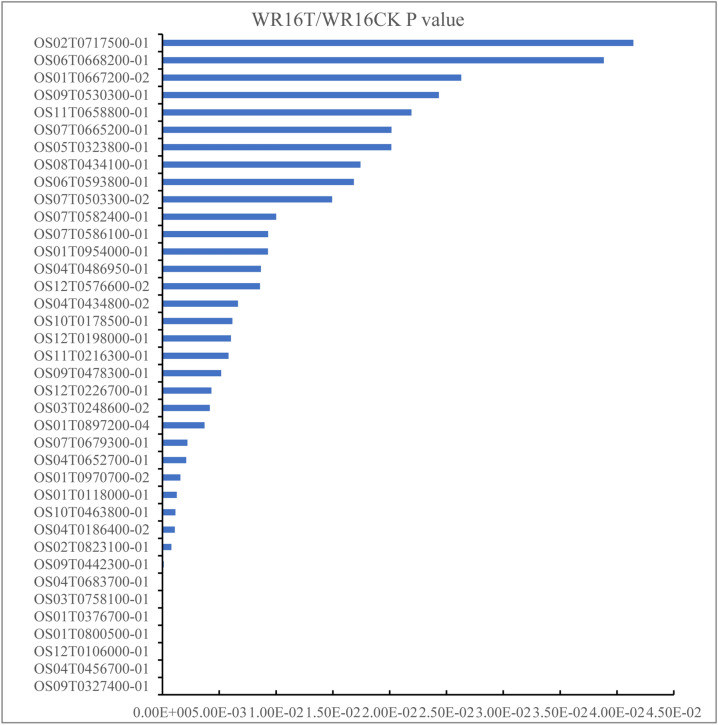
The P value of 38 Up-Up proteins based on transcriptomics and proteomics between WR16CK and WR16T. WR16CK represented WR16 samples from the water fields, WR16T represented WR16 samples from the drought fields.

**Figure 3:**
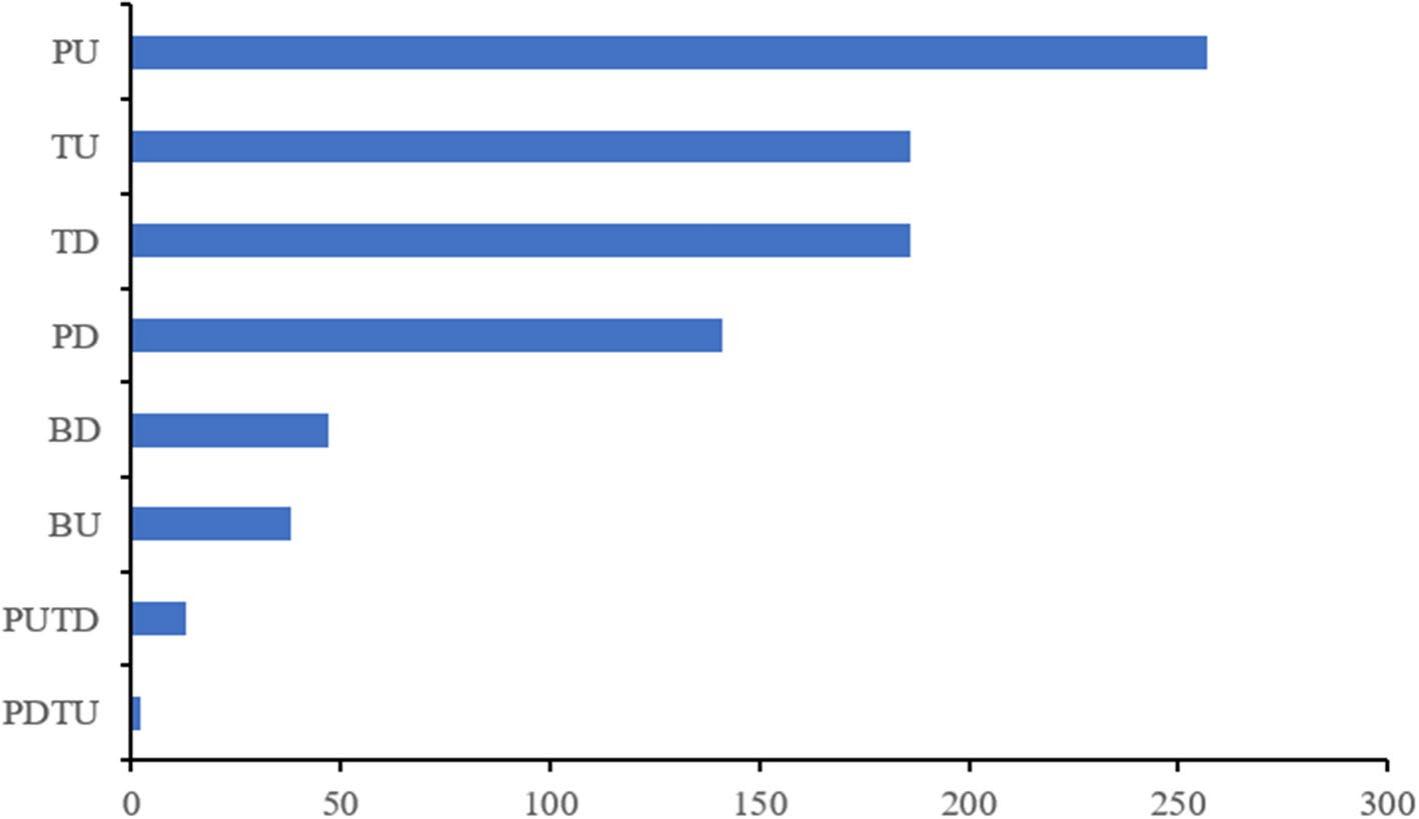
Bar diagram comparing significantly differentially expressed proteins or transcripts based on transcriptome and proteome for WR16. *BU* co-upregulated (BU) in proteins and transcripts, *BD* co-downregulated (BD) in proteins and transcripts, *PU* regulated in proteins and not changed in transcripts, *TU* proteins were not changed but their transcripts were upregulated, *PD* proteins were significantly downregulated in proteins and not changed in transcripts, *TD* proteins were not changed but their transcripts were downregulated, *PUTD* proteins were significantly upregulated in proteins and their transcripts were downregulated, *PDTU* proteins were significantly downregulated but their transcripts were significantly upregulated.

Transcriptome and proteome crosstalk analysis proceeded based on the strong drought tolerance accession WR16. Comparing the proteome and transcriptome datasets, total 4,968 proteins or transcripts were identified (Table S6). Among them, a total of 38 proteins were significantly co-upregulated (BU) in proteins and transcripts, 47 proteins were significantly co-downregulated (BD) in proteins and transcripts (Fig. 2, Table S7). In addition, 257 proteins were significantly upregulated in proteins and not changed in transcripts (PU); 186 proteins were not changed but their transcripts were upregulated (TU); 141 proteins were significantly downregulated in proteins and not changed in transcripts (PD); 186 proteins were not changed but their transcripts were downregulated (TD); 13 proteins were significantly upregulated in proteins and their transcripts were downregulated (PUTD); and two proteins were significantly downregulated but their transcripts were significantly upregulated (PDTU) (Fig. 3).

**Figure 4:**
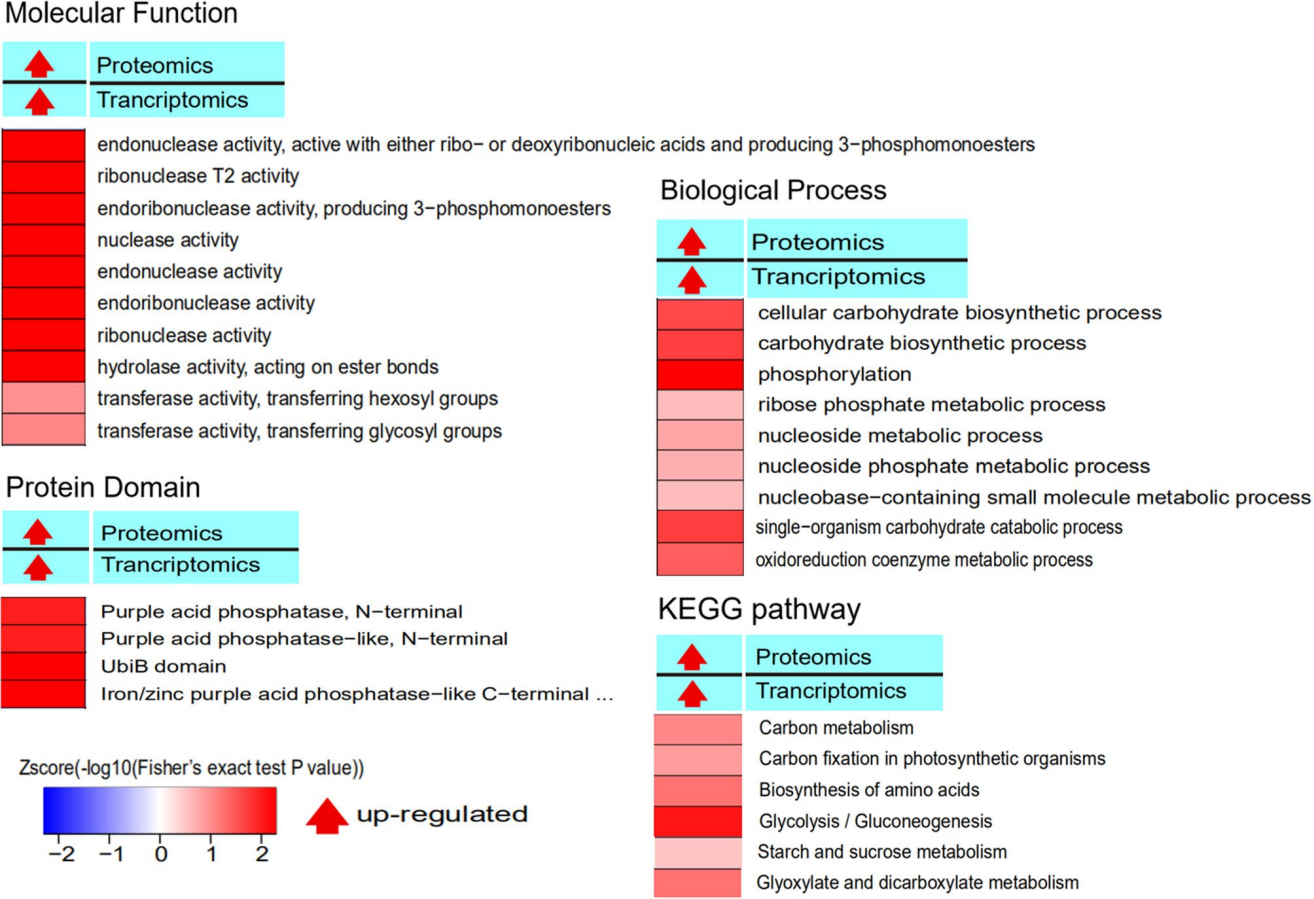
GO, protein domain, and KEGG pathway analyses of co-upregulated proteins for WR16. Different shades of red represent the differentiation of different processes in the bar. Each bar corresponds to the description of each process.

To find more biological information, the functions of co-upregulated proteins were analyzed using Gene Ontology (GO), protein domain, and KEGG (Kyoto Encyclopedia of Genes and Genomes) pathways (Fig. 4, Table S8). GO analyses were performed for 38 co-upregulated proteins, and results showed that for biological process, the 38 co-upregulated proteins were significantly enriched in five groups (Fig. 4, Table S8), including cellular carbohydrate metabolic biological process, phosphorylation, single-organism carbohydrate catabolic biological process, oxidoreduction coenzyme metabolic biological process, and the phosphorylation and oxidoreduction coenzyme metabolic biological process and so on.

At the molecular function level, 38 co-upregulated proteins (ID refer to the Table S7) were significantly enriched in ten groups (Fig. 4, Table S8), including endonuclease catalyze activity, endoribonuclease catalyze activity, nuclease catalyze activity, ribonuclease catalyze activity, and hydrolase catalyze activity. In protein domain, 38 proteins were significantly enriched into four protein domains: the ubiB domain; C-terminal domain with iron/zinc activity center (similar to purple acid phosphatase); N-terminal end with iron/zinc activity center (similar to purple acid phosphatase); and purple acid phosphatase (PAP)(Fig. 4, Table S8). For cellular component, no groups were significantly enriched. KEGG pathway analysis showed that 38 co-upregulated proteins were significantly enriched in six KEGG pathways (Fig. 4, Table S8), including glycolysis/gluconeogenesis, carbon metabolism, glyoxylate and dicarboxylate metabolism, carbon fixation in photosynthetic organisms, starch and sucrose metabolism, and biosynthesis of amino acids.

### Targeted parallel reaction monitoring proteomics between WR16 and WR24

According to the transcriptome and proteome crosstalk analysis, in the following study, we only considered the roles of the co-upregulated proteins under drought stress conditions in the tolerant to drought accession WR16. A total of 38 co-upregulated proteins were used for further PRM verification. We selected a moderate drought-tolerant accession WR24 as expanded verification material to confirm the existence of the 38 proteins in other materials.

**Figure 5:**
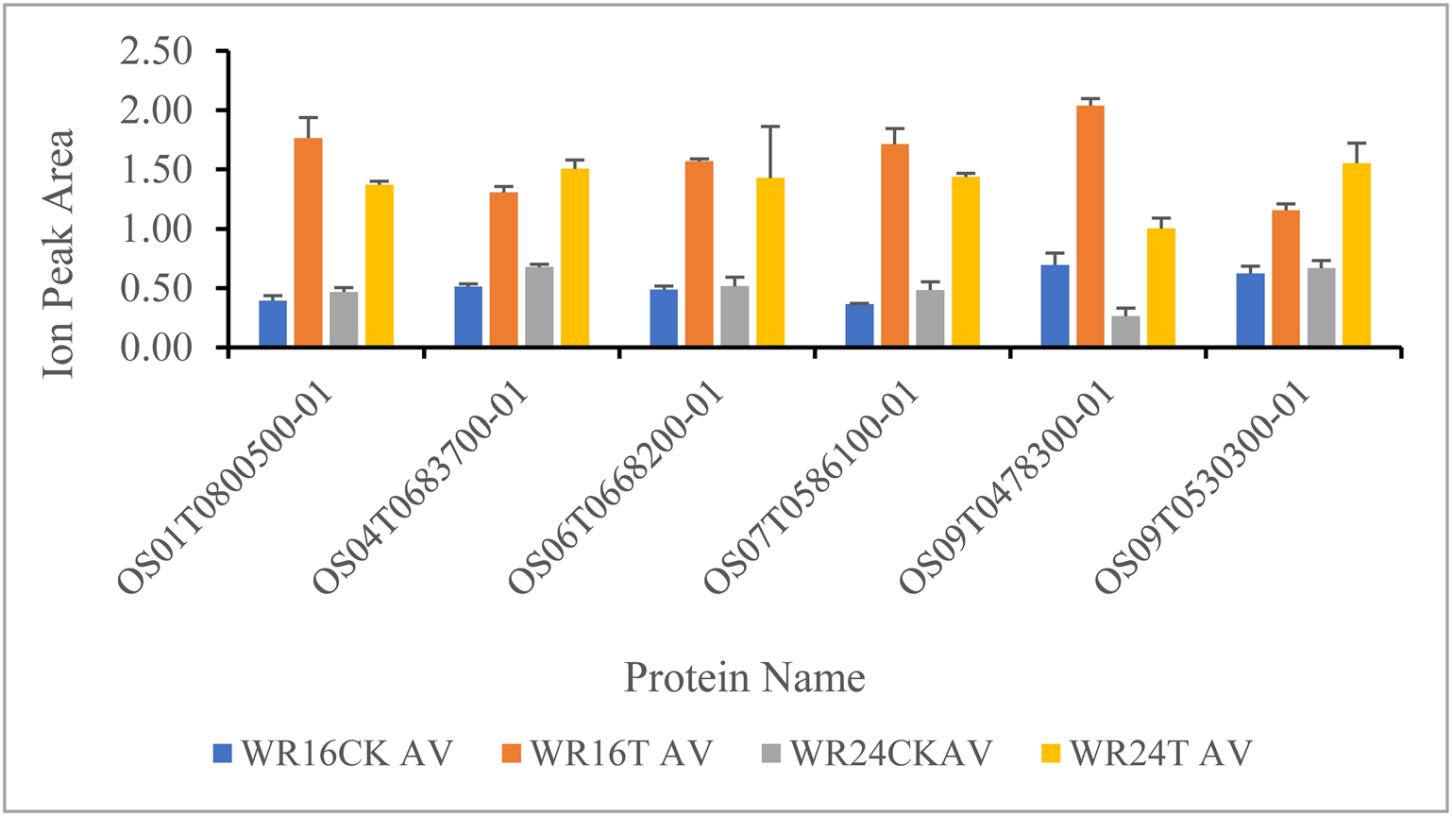
Ion peak area diagrams based on unique peptides for six proteins. T represents the treatment samples for accession WR16 and WR24, CK represents the control group for WR16 and WR24. X-axis represented the verified protein name Os01T0800500-01 OS04T0683700-01, OS06T0668200-01, OS09T0478300-01, OS09T0530300-01, OS07T586100-01. Y-axis represented the Ion peak area of each protein.

Using PRM, six proteins were verified in 12 samples of WR16 and WR24 accessions. According to the different chromosomes for different proteins, based on the different unique peptides, we drew the ion peak picture; the quantitated proteins were distributed on chromosomes 1, 4, 6, 7, 9 (Fig. 5). Among them, OS01T0800500-01 was verified through the unique peptide AQYLTSDPGYLGCK; OS04T0683700-01 was verified through two unique peptides DAAGQVHLAGFPASAAAAAK and IVAQHFVVPVLPTK, OS06T0668200-01 was verified through the unique peptide LAATLPDGGVLLLENVR; OS09T0478300-01 was verified through two unique peptides NHQPIVQVLIDGK and SLATCTYELR and OS09T0530300-01 was verified through two unique peptides NITFAPFGEQWR and TSLFVNAWAIGR. OS07T0586100-01 was verified through two unique peptides SQFVYSNIGGIYR and SVSWDGVHFTEAANR (Table S9).

### The interaction network of six major proteins

**Figure 6:**
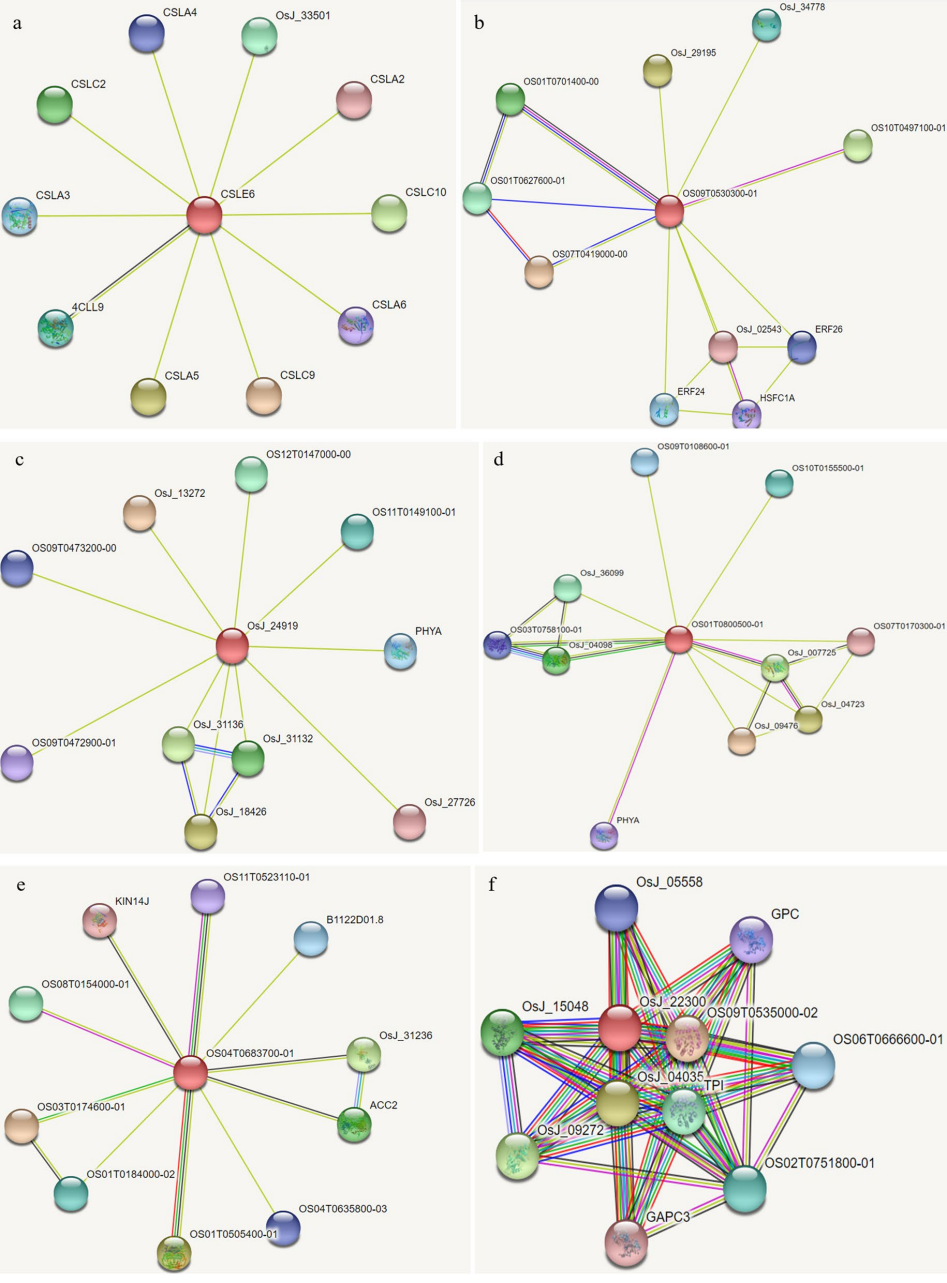
The functional protein association networks of the six verified proteins. (**a**) The association network of Os09T0478300-01 protein. (**b**) The association network of OS9T05300300-01 protein. (**c**) The association network of OS07T0586100-01 protein. (**d**) The association network of OS01T0800500-01 protein. (**e**) The association network of OS04T0683700-01 protein. (**f**) The association network of OS06T0668200-01 protein.

The OS09T0478300-01 protein, named CSLE6, interacted with proteins 4CLL9, CSLA3, CSLA6, CSLC2, CSLC9, and CSLC10 (Fig. 6a). The OS09T0530300-01 protein, interacted with OS01T0701400-00, OS07T0419000-00, OS01T0627600-01, ERF26, and OSJ_02543 (Fig. 6b). The OS07T0586100-01 protein, named OSJ_24919, interacted with OS09T0473200-00, OS09T0472900, OSJ_27726, OSJ_31136, and PHYA (Fig. 6c). The OS01T0800500-01 protein, interacted with OS09T0108600-01, OS07T0170300-01, OSJ_36099, OSJ_007725, and PHYA (Fig. 6d). The OS04T0683700-01 protein, interacted with OS01T0505400-01, OS01T0184000-02, and OSJ_31236 (Fig. 6e). The OS06T0668200-01 protein, named OSJ_22300, interacted with OS09T0535000-02, OSJ_04035, OSJ_15048, and OSJ_05558 (Fig. 6f).

## Discussion

Targeted proteomics can precisely quantify specific proteins under complex genetic backgrounds and allow researchers to more reliably quantify drought tolerance proteins. In our study, 38 co-upregulated proteins were identified as significantly enriched in five biological process (Fig. 4). Correlations between the phosphorylation and oxidoreduction coenzyme metabolic biological processes with the drought stress conditions have been verified in some previous studies. A relationship between several phosphorylation proteins and drought tolerance in apple was identified^17^, and phosphorylation of proteins in response to water deficit during wheat flag leaf and grain development was verified^18^. In protein domain, 38 proteins were significantly enriched in the four protein domains. Purple acid phosphatase (PAP) is related to drought tolerance in plants, and contains a C-terminal domain lacking a catalytic function and an N-terminal end with an iron/zinc activity center of PAP^16^. One study suggested that PAP protein is related to phosphorus metabolism, stress resistance, and anti-aging biological process^19^. AtPAP10 protein in Arabidopsis can degrade the substrate, release organophosphorus, and increase phosphorus absorption^20^. GmPAP3 in soybean can decrease the ROS accumulation of mitochondria to deal with some adversity^21^.

Targeted parallel reaction monitoring (PRM) proteomics is the most popular technology for verifying the quantified proteins through unique peptides. In the present study, accession WR16 of weedy red rice was identified as a drought tolerant accession among 133 germplasms and one cultivated *Japonica* rice reference *cv*. IAPAR-9. In drought tolerance accession WR16, iTraq targeted proteomics was combined with transcriptomics and 38 proteins were precisely quantified under complex drought stress backgrounds (Table S7). Targeted PRM further verified the existence of six selected proteins through unique peptides of each protein in WR16. We also verified the presence of these six proteins in another drought tolerance accessions WR24 and verified the reliability and authenticity of the six proteins.

The interacting network (Fig. 6) of six proteins mainly occurred on chromosomes 1, 4, 7, and 9. Among them, OS09T0478300-01, OS09T0530300-01, and OS01T0800500-01 connect with all other proteins, and are seen as the core of the network and function as a bridge in the crop defense system. Therefore, all proteins form a combined defense system to confront adverse conditions. Drought is the most complicated abiotic stress factor. When rice is subjected to drought stress at the reproductive stage, to ensure reproduction and propagate offspring, three major defensive systems are initiated in the organism: (1) a water retention system to reduce water transpiration and loss; (2) a scavenging system to eliminate toxic oxidation reductants produced under adverse environments; and (3) a positive coping system to the drought stress, such as phosphorylation pathways^1,40^.

In our study, OS09T0478300-01 and related proteins (Fig. 6a) are connected to the Golgi-localized beta-glycan synthase that polymerizes the backbones of non-cellulosic polysaccharides (hemicelluloses) of the plant cell wall, and accounts for the response of the plant cell related to the rice water retention system. According to the analysis, protein CSLE6 and 4CLL9 are co-expressed (Fig. 6a), and are located on chromosome 9 between 18,321.214 and 18,315.154 kbp. In our previous study, we identified the major *qLRI9-1* gene, which is related to the drought-resistance phenotype LRI (leaf rolling index)^3^ on chromosome 9 between RM3600-RM553 (17,108.844–19,325.247 kbp) using two RIL genetic populations. *qLRI9-1* thus contained the region of the gene *CSLE6*. The gene *Dro1* was identified at RM24393-RM7424 (16,679.9–17,287.9 kbp) for the ratio of deep rooting RT/RY^12^, and qRT9 was located at RM410-RM7048 (16,881.647–17,589.296 kbp) for root thickness and root length in upland rice^22^. These drought tolerance related genes have been located on chromosome 9 adjacent to the *CSLE6* gene, which suggests regions on chromosome 9 with many genes that assist *CSLE6* in resisting stress conditions. OS09T0530300-01 and related proteins are putative cytochrome P450s (Fig. 6b), which are involved with crop resistance to oxidation under drought stress conditions, and are part of the scavenging system. The corresponding gene, named *CGSNL*, was also located on chromosome 9 between 20,774.283 and 20,776.144 kbp. Analysis showed that OS09T0530300-01 is a putative cytochrome P450, which interacts with the proteins Os01T0701400-00, Os01T0627600-01, Os07T0419000-00; these proteins are also P450 family proteins related to crop resistance to oxidation under drought stress conditions (Fig. 6b). Therefore, we deduced that all above genes on chromosome 9 constructed a linkage group to respond to drought stress.

OS07T0586100-01 is putative early nodulin, named OSJ_24919 (Fig. 6c), interacting with Os09T0472900-01 and Os09T0473200-01. Nodulin-related protein was verified to function in heat stress of *Arabidopsis*^36^. OS01T0800500-01 is a purple acid phosphatase (PAP) protein, which is co-expressed with the proteins Os03T0758100-01, OsJ_04098, and OsJ_007725; all of these proteins are phosphorylating enzymes that are essential in responding to drought stress conditions (Fig. 6d). Phosphorylation-guarded enzymes were identified that contribute to blast resistance in rice^37^. OS04T0683700-01 belongs to Acyl-Activating Enzyme 3, adenosine monophosphate binding protein 3, mainly regulating rice blast resistance, floret development, and lignin biosynthesis, and interacting with Os01T0184000-02 and Os01T0505400-01 (Fig. 6e). OS04T0683700-01 can activate ligase enzyme activity and stress response in rice^38^. OS06T0668200-01 is a phosphoglycerate kinase named OSJ_22300 (Fig. 6f). Phosphoglycerate kinase appears to increase salinity tolerance and enhance yield in crop^39^.

When crops experience adverse conditions, the above three systems are mutually or independently initiated according to the level of stress. Under slight stress, maybe only retention water system was initiated in different accessions; under serious stress, these three systems may be initiated and interact with each other to respond to serious stress^23–26^. In our study, we chose the most serious period of drought stress to investigate the phenotype data consistent with our above analysis. For further study, we edited three genes, and also generated their overexpression vector pTCK303 with the ultimate goal of confirming its phenotype. All above analysis suggested that drought tolerance is quite complicated and requires many genes that interact with each other. Of these genes, few are major genes such as *CSLE6*; some are minor genes. However, the regulation systems and functional mechanisms in drought stress have not been fully studied, and require additional research in the future.

## Materials and methods

### Plant materials and drought stress

A total of 133 purified and stable weedy rice accessions including a strong drought tolerance reference accession IAPAR-9 from the Gene Bank of the Chinese Academy of Agricultural Sciences (CAAS) (Table S2), were sown in seedling fields at the Nanbin farm in Sanya city of Hainan province on December 11, 2016. Seedlings were grown in the same conditions for about one month. Then the seedlings were transferred to the control and treatment fields with two replicates on January 15, 2017. On January 25, 2017, the supply of water to the treatment fields was stopped and they were considered drought fields; water was still supplied in the control fields (water fields) according to the normal field management methods of the CAAS. From January 25 to March 22, 2017, Total consecutive 57 days, it is not rain in the region at the Nanbin farm in Sanya city of Hainan province, China (data from the weather forecast of 2017) (Table S1). Based on the criteria of drought (GBT 20481-2017), when days with no rain are between 45 and 60 days in spring, it is identified as severe meteorological drought (https://www.lddoc.cn/p-126352.html). From Mar 15 to Mar 22, this stage was regarded as the strong-stress treatment stage and occurred during the heading stage of rice (Fig. 1). Ten drought tolerance indices (Table S3), including data for agronomic traits (PH: plant height, SL: stem length, PL: panicle length, TN: tiller number), physical signs (CC: chlorophyll content, NC: nitrogen content), and four drought tolerance grades (WR1-1, WR1-2, WR2-1, WR2-2) from Mar 17 and Mar 21 were used to evaluate drought tolerance according to the drought-resistance evaluation standard (Table 2)^27^.

**Table 2:** The investigation of Leaf Wither Degree under the drought-resistance conditions.

Degree	LWD (leaf wither degree)	Drought-tolerance capacity
1	Normal leaf	Very strong
3	1/4 of total leaf area to wither	Strong
5	1/4–1/2 of total leaf area to wither	Middle strong
7	Greater than 2/3 of total leaf area to wither	Weak
9	Total plants wither	Very weak

### PCA analysis

Via correlation analyses between each index and drought-resistance capacity, significantly correlated indicators were selected to evaluate the drought tolerance of each variety. Though these methods decreased the number of evaluation indices, due to the difference in drought tolerance mechanisms in diverse accessions, information about each evaluation index can overlap; therefore, it is very difficult to correctly evaluate the drought tolerance of each variety using these indices. However, PCA (principal component analysis) can reduce the number of the variables (indices) to several potential factors and assure that the information of the variables (indices) is not lost or that little is lost. The information concerning the several factors highly summarizes and represents a large amount, and it not only reduces the number of the variables but also reestablishes the inner link among all the variables^41^. In this study, PCA of ten drought tolerance indices (Table S3) was carried out using Spass16.0 software.

According to formula (1): the subordinate function values *μ*(*x*) for the comprehensive index of each sample was calculated (Table S5), where *x*_*i*_ represents the *i*th comprehensive index, *x*_min_ represents the minimum for the *i*th comprehensive index, *x*_max_ represents the maximum for the *i*th comprehensive index, and the subordinate function values reflect the drought-resistance capacity of each variety

1 $$\mu (x_{i} ) = \frac{{x_{i} - x_{\min } }}{{x_{\max } - x_{\min } }},\quad i = 1,2,3, \ldots n$$

According to formula (2): the weight function *w*_*i*_ was calculated and represents the relative importance for the *i*th comprehensive index, and *p*_*i*_ represents the contribution for the *i*th comprehensive index. The comprehensive weights for the four comprehensive indices were 0.3589, 0.3123, 0.1951, and 0.1338, respectively, the total weight is 1

2 $$w_{i} = {{p_{i} } \mathord{\left/ {\vphantom {{p_{i} } {\sum\limits_{i = 1}^{n} {p_{i} } }}} \right. \kern-\nulldelimiterspace} {\sum\limits_{i = 1}^{n} {p_{i} } }},\quad i = 1,2,3, \ldots n$$

According to formula (3): the comprehensive evaluation value (D) for drought-resistance capacity at the heading period was calculated (Table S10). The D Value reflected the drought-resistance capacity of each sample^28^

3 $$D = \sum\limits_{i = 1}^{n} {\left[ {\mu (x_{i} ) * w_{i} } \right]} ,\quad i = 1,2,3, \ldots n$$

### RNA extraction and transcriptome sequencing

The drought tolerant accession WR16, with red seed coat and long awn, from Gene Bank of Chinese Academy of Agricultural Sciences, was selected based on the above comprehensive evaluation assay for further analysis; three replicates were used. On March 21, 2017, at 15:00, whole single flag leaves were collected from selected separate plants in each weedy red rice accession and cv. IAPAR-9 grown in the field trial. Leaf samples were collected in 2 ml plastic centrifuge tubes and immediately frozen in liquid nitrogen. Frozen leaf samples were transported to the laboratory and kept at − 80 °C until RNA extraction. RNA was extracted using RNA Easy Fast Plant Tissue Kit (DP452, TIANGEN BIOTECH, Beijing) and checked using an Agilent 2100 Bioanalyzer (CA, USA) for the control and treatment samples. RNA samples were adjusted with 40 µl sterile water to a concentration of 200–250 ng/µl, which was adequate for the following study. Samples were considered to be of adequate concentration and quality when the value of OD260/280 was more than 1.87 and less than 2.09; the value of OD260/230 was more than 2.02 and less than 2.51; the value of 28S/18S was more than 1.1 and less than 1.6; and the value of RIN was more than 7.5 and less than 8.2. The transcriptome sequencing was conducted by allwegene technologies company (Beijing, China). Sequencing data was then checked by Phred quality score and Q value, with sequence comparison and transcript splicing carried out by Tophat2 and Cufflinks software^34,35^. Gene quantification analysis was conducted by the fragments per kilobase of exon model per million mapped reads (FPKM), based on Gene Ontology (GO) and Kyoto Encyclopedia of Genes and Genomes (KEGG) analyses^29,30^. The Japonica rice cultivar Nipponbare was used as the reference genome.

### TMT proteome assay

Protein was extracted from WR16 samples, and 200 µg of each sample was digested using trypsin. For digestion, the protein solution was reduced with 5 mM dithiothreitol for 30 min at 56 °C, and alkylated with 11 mM iodoacetamide for 15 min at room temperature in darkness. The protein sample was then diluted to a urea concentration of less than 2 M^42^. Finally, trypsin was added at a 1:50 trypsin-to-protein mass ratio for the first digestion overnight and 1:100 trypsin to protein mass ratio for a second 4 h-digestion^43^.

In total, 100 µg of each sample was labeled with TMT6 plex according to the manufacturer’s instructions (TMT Mass Tagging Kits and Reagents, Thermo Fisher, CA, USA). After labeling, the samples were dried under vacuum, then labeled samples were combined and solubilized in ammonium formate (pH 10). Then high pH fractions were desalted and analyzed by liquid chromatography (LC) MS/MS using an RSLC system (Thermo Fisher, CA, USA). Using Proteome Discoverer (Thermo Fisher, version 1.4), the raw data was converted for further analysis. The LC-MSMS data was searched using multidimensional protein identification technology (MudPIT)^31^, against the reverse of all protein sequences of Japonica rice with a series of external contaminants from the common Repository of Adventitious Proteins (cRAP) (ftp://ftp.thegpm.org/fasta/cRAP/crap.fasta) using GPM-XE Manager version 3.0 (https://gpm-cyclone-xe.software.informer.com/versions/). A merged output file was produced using MudPIT, to conduct ion quantitation based on a TMT reporter^32,33^ using an in-house Perl script; the intensity of each TMT channel was recorded.

### PRM analysis based on LC–MS/MS

According to the transcriptome and proteome crosstalk analysis, we only considered the roles of co-upregulated proteins under drought stress conditions in the drought-resistance variety WR16. A total of 38 co-upregulated proteins from WR16 were selected for further PRM verification. We selected another drought tolerant accession WR24 to further verify the existence of the 38 proteins. According to the number of unique peptides, comprehensive score, and signal intensity, nine proteins were determined to be suitable for further PRM testing. Using PRM, we verified nine proteins in 12 samples of WR16 and WR24 accessions. Each variety had three replicates, containing the treatment and the control for a total of 12 samples. Each protein was quantitated using more than two unique peptides based on the protein ion peak area in the 12 samples.

Using 0.1% formic acid as solvent A, the dissolved tryptic peptides were loaded onto an in-house reversed-phase analytical column. In solvent B (0.1% formic acid in 90% acetonitrile), the gradient was set for an increase from 7 to 25% for 0–40 min, 25% to 35% for 40–52 min and raised to 80% from 52–56 min, then maintained at 80% in the last 56–60 min, with a flow rate of 350 nl/min on an EASY-nLC 1000 UPLC system. The peptides were injected into an NSI source and analyzed by MS/MS using a Q ExactiveTM Plus (Thermo Fisher, CA). The electrospray voltage was set at 2.0 kV. The scan range was designed for 350–1,000 m/z, and the scan resolution ratio was set at 70,000 for full scan MS. Peptides were then detected for MS/MS with an HCD setting of 27 with the Orbitrap (Thermo Fisher, CA) at a resolution of 17,500; data independent acquisition (DIA) was used. Automatic gain control (AGC) was designed at 3E6 for full MS with the maximum IT set at 50 ms for full MS and 1E5 for MS/MS with the maximum IT set at 80 ms. The isolation window for MS/MS was 1.6 m/z.

### Data analysis

The resulting data were processed using Skyline (https://skyline.ms/project/home/software/Skyline/). Peptide settings were as follows: trypsin [KR/P] was set for protein enzyme, maximum missing cut bits was set as 0.7–25 amino acid residue, carbamidomethyl on Cys was set as a fixed modification. Transition settings were set as follows: 2, 3 were set for precursor charges; 1 was set for ion charges; and b, y was set for ion types. The output ions were selected from ion 3 to the last ion, the ion match quality of error tolerance was set as 0.02 Da.

## Supplementary information

Supplementary Tables.

## Data Availability

The datasets generated during and analyzed during the current study are available from the corresponding author on reasonable request.
