# Supplementary material for: Parallel reaction monitoring revealed tolerance to drought proteins in weedy rice (Oryza sativa f. spontanea)
Source: Sci Rep. 2020 Jul 31;10:12935. doi: 10.1038/s41598-020-69739-9 (PMC7395730; doi:10.1038/s41598-020-69739-9)
Supplement: Supplementary file 1 — Supplementary Tables. [file 41598_2020_69739_MOESM1_ESM.docx]

Parallel reaction monitoring revealed tolerance to drought proteins in weedy rice

Bing Han^1^, Xiaoding Ma^1^, Di Cui^1^, Leiyue Geng^1, 2^, Guilan Cao^1^, Hui Zhang ^1^*, Longzhi Han^1^*

^1^Institute of Crop Sciences, Chinese Academy of Agricultural Sciences, Beijing 100081, China

^2^Coastal Agriculture Institute, Hebei Academy of Agricultural and Forestry Sciences, Tangshan 063299, China

*Corresponding author. E-mail address: [hanlongzhi@caas.cn](mailto:hanlongzhi@caas.cn), [zhanghui06@caas.cn](mailto:zhanghui06@caas.cn)

Bing Han: [hab_1011@163.com](mailto:hab_1011@163.com)

Xiaoding Ma: [maxiaoding@caas.cn](mailto:maxiaoding@caas.cn)

Di Cui: [cuidi@caas.cn](mailto:cuidi@caas.cn)

Leiyue Geng: [15733330272@163.com](mailto:15733330272@163.com)

Guilan Cao: [caoguilan@caas.cn](mailto:caoguilan@caas.cn)

Table S1 The rainfall of Hainan fields on treatment stage.

| Date | Tm（Max） | Tm（Min） | Rain | Wind | Air index |
| --- | --- | --- | --- | --- | --- |
| 2017/1/25 | 26℃ | 20℃ | cloudy | 3-4 grade | 28 |
| 2017/1/26 | 27℃ | 20℃ | cloudy | 3-4 grade | 35 |
| 2017/1/27 | 27℃ | 19℃ | cloudy | 3-4 grade | 43 |
| 2017/1/28 | 28℃ | 20℃ | cloudy | 3-4 grade | 47 |
| 2017/1/29 | 29℃ | 20℃ | cloudy | 3-4 grade | 41 |
| 2017/1/30 | 28℃ | 20℃ | cloudy | 3-4 grade | 34 |
| 2017/1/31 | 26℃ | 19℃ | cloudy | 3-4 grade | 33 |
| 2017/2/1 | 26℃ | 21℃ | cloudy | 3-4 grade | 23 |
| 2017/2/2 | 28℃ | 20℃ | cloudy | 3-4 grade | 26 |
| 2017/2/3 | 26℃ | 21℃ | cloudy | 3-4 grade | 26 |
| 2017/2/4 | 28℃ | 21℃ | cloudy | 3-4 grade | 27 |
| 2017/2/5 | 28℃ | 19℃ | cloudy | 3-4 grade | 25 |
| 2017/2/6 | 29℃ | 22℃ | cloudy | 3-4 grade | 33 |
| 2017/2/7 | 29℃ | 22℃ | cloudy | 3-4 grade | 30 |
| 2017/2/8 | 29℃ | 18℃ | cloudy | 3-4 grade | 45 |
| 2017/2/9 | 23℃ | 17℃ | cloudy | 3-4 grade | 25 |
| 2017/2/10 | 22℃ | 17℃ | cloudy | 3-4 grade | 37 |
| 2017/2/11 | 24℃ | 14℃ | cloudy | 3-4 grade | 50 |
| 2017/2/12 | 25℃ | 18℃ | cloudy | 3-4 grade | 64 |
| 2017/2/13 | 26℃ | 20℃ | cloudy | 3-4 grade | 53 |
| 2017/2/14 | 26℃ | 20℃ | cloudy | 3-4 grade | 38 |
| 2017/2/15 | 27℃ | 20℃ | cloudy | 3-4 grade | 34 |
| 2017/2/16 | 29℃ | 20℃ | cloudy | 3-4 grade | 31 |
| 2017/2/17 | 29℃ | 20℃ | cloudy | 3-4 grade | 31 |
| 2017/2/18 | 29℃ | 20℃ | cloudy | 3-4 grade | 30 |
| 2017/2/19 | 28℃ | 21℃ | cloudy | 3-4 grade | 36 |
| 2017/2/20 | 31℃ | 22℃ | cloudy | 3-4 grade | 37 |
| 2017/2/21 | 30℃ | 23℃ | cloudy | 3-4 grade | 26 |
| 2017/2/22 | 32℃ | 24℃ | cloudy | 3-4 grade | 17 |
| 2017/2/23 | 30℃ | 22℃ | cloudy-scouther | 3-4 grade | 21 |
| 2017/2/24 | 28℃ | 21℃ | cloudy | 3-4 grade | 25 |
| 2017/2/25 | 29℃ | 20℃ | cloudy | 3-4 grade | 23 |
| 2017/2/26 | 27℃ | 19℃ | cloudy | 3-4 grade | 20 |
| 2017/2/27 | 25℃ | 21℃ | cloudy | 3-4 grade | 28 |
| 2017/2/28 | 28℃ | 21℃ | cloudy | 3-4 grade | 36 |
| 2017/3/1 | 28℃ | 20℃ | cloudy | 3-4 grade | 46 |
| 2017/3/2 | 28℃ | 19℃ | cloudy | 3-4 grade | 64 |
| 2017/3/3 | 28℃ | 20℃ | cloudy | 3-4 grade | 62 |
| 2017/3/4 | 29℃ | 20℃ | cloudy | 3-4 grade | 52 |
| 2017/3/5 | 30℃ | 21℃ | cloudy | 3-4 grade | 48 |
| 2017/3/6 | 31℃ | 21℃ | cloudy | 3-4 grade | 40 |
| 2017/3/7 | 29℃ | 21℃ | cloudy | 3-4 grade | 35 |
| 2017/3/8 | 28℃ | 21℃ | cloudy | 3-4 grade | 37 |
| 2017/3/9 | 31℃ | 21℃ | cloudy | 3-4 grade | 32 |
| 2017/3/10 | 31℃ | 23℃ | cloudy | 3-4 grade | 43 |
| 2017/3/11 | 31℃ | 23℃ | cloudy | 3-4 grade | 42 |
| 2017/3/12 | 31℃ | 24℃ | cloudy | 3-4 grade | 35 |
| 2017/3/13 | 31℃ | 23℃ | cloudy | 3-4 grade | 39 |
| 2017/3/14 | 31℃ | 23℃ | cloudy | 3-4 grade | 42 |
| 2017/3/15 | 30℃ | 23℃ | cloudy | 3-4 grade | 33 |
| 2017/3/16 | 30℃ | 25℃ | cloudy | 3-4 grade | 35 |
| 2017/3/17 | 29℃ | 25℃ | cloudy | 3-4 grade | 39 |
| 2017/3/18 | 29℃ | 24℃ | cloudy | 3-4 grade | 37 |
| 2017/3/19 | 31℃ | 25℃ | cloudy | 3-4 grade | 38 |
| 2017/3/20 | 30℃ | 25℃ | cloudy | 3-4 grade | 35 |
| 2017/3/21 | 31℃ | 25℃ | cloudy | 3-4 grade | 31 |
| 2017/3/22 | 31℃ | 25℃ | cloudy | 3-4 grade | 28 |
| 2017/3/23 | 31℃ | 25℃ | cloudy | 3-4 grade | 31 |
| 2017/3/24 | 31℃ | 25℃ | cloudy | 3-4 grade | 26 |

| Table S2 the accession name of each samples | | | | | | | |  |  |  |  |  |  |  |
| --- | --- | --- | --- | --- | --- | --- | --- | --- | --- | --- | --- | --- | --- | --- |
| Num | Name | | Description | | | ID | |  |  |  |  |  |  |  |
| 1 | WR2 | | weedy rice | | | 2016WR1 | |  |  |  |  |  |  |  |
| 2 | WR3 | | weedy rice | | | 2016WR2 | |  |  |  |  |  |  |  |
| 3 | IAPAR-9 | | Japonica | | | 2016WR3 | |  |  |  |  |  |  |  |
| 4 | WR5 | | weedy rice | | | 2016WR4 | |  |  |  |  |  |  |  |
| 5 | WR6 | | weedy rice | | | 2016WR5 | |  |  |  |  |  |  |  |
| 6 | WR7 | | weedy rice | | | 2016WR6 | |  |  |  |  |  |  |  |
| 7 | WR8 | | weedy rice | | | 2016WR7 | |  |  |  |  |  |  |  |
| 8 | WR9 | | weedy rice | | | 2016WR8 | |  |  |  |  |  |  |  |
| 9 | WR10 | | weedy rice | | | 2016WR9 | |  |  |  |  |  |  |  |
| 10 | WR11 | | weedy rice | | | 2016WR10 | |  |  |  |  |  |  |  |
| 11 | WR12 | | weedy rice | | | 2016WR11 | |  |  |  |  |  |  |  |
| 12 | WR13 | | weedy rice | | | 2016WR12 | |  |  |  |  |  |  |  |
| 13 | WR15 | | weedy rice | | | 2016WR13 | |  |  |  |  |  |  |  |
| 14 | WR160 | | weedy rice | | | 2016WR14 | |  |  |  |  |  |  |  |
| 15 | WR17 | | weedy rice | | | 2016WR15 | |  |  |  |  |  |  |  |
| 16 | WR18 | | weedy rice | | | 2016WR16 | |  |  |  |  |  |  |  |
| 17 | WR19 | | weedy rice | | | 2016WR17 | |  |  |  |  |  |  |  |
| 18 | WR20 | | weedy rice | | | 2016WR18 | |  |  |  |  |  |  |  |
| 19 | WR21 | | weedy rice | | | 2016WR19 | |  |  |  |  |  |  |  |
| 20 | WR22 | | weedy rice | | | 2016WR20 | |  |  |  |  |  |  |  |
| 21 | WR23 | | weedy rice | | | 2016WR21 | |  |  |  |  |  |  |  |
| 22 | WR24 | | weedy rice | | | 2016WR22 | |  |  |  |  |  |  |  |
| 23 | WR25 | | weedy rice | | | 2016WR23 | |  |  |  |  |  |  |  |
| 24 | WR26 | | weedy rice | | | 2016WR24 | |  |  |  |  |  |  |  |
| 25 | WR27 | | weedy rice | | | 2016WR25 | |  |  |  |  |  |  |  |
| 26 | WR28 | | weedy rice | | | 2016WR26 | |  |  |  |  |  |  |  |
| 27 | WR29 | | weedy rice | | | 2016WR27 | |  |  |  |  |  |  |  |
| 28 | WR30 | | weedy rice | | | 2016WR28 | |  |  |  |  |  |  |  |
| 29 | WR31 | | weedy rice | | | 2016WR29 | |  |  |  |  |  |  |  |
| 30 | WR32 | | weedy rice | | | 2016WR30 | |  |  |  |  |  |  |  |
| 31 | WR33 | | weedy rice | | | 2016WR31 | |  |  |  |  |  |  |  |
| 32 | WR94 | | weedy rice | | | 2016WR32 | |  |  |  |  |  |  |  |
| 33 | WR95 | | weedy rice | | | 2016WR33 | |  |  |  |  |  |  |  |
| 34 | WR96 | | weedy rice | | | 2016WR34 | |  |  |  |  |  |  |  |
| 35 | WR97 | | weedy rice | | | 2016WR35 | |  |  |  |  |  |  |  |
| 36 | WR98 | | weedy rice | | | 2016WR36 | |  |  |  |  |  |  |  |
| 37 | WR99 | | weedy rice | | | 2016WR37 | |  |  |  |  |  |  |  |
| 38 | WR100 | | weedy rice | | | 2016WR38 | |  |  |  |  |  |  |  |
| 39 | WR101 | | weedy rice | | | 2016WR39 | |  |  |  |  |  |  |  |
| 40 | WR102 | | weedy rice | | | 2016WR40 | |  |  |  |  |  |  |  |
| 41 | WR143 | | weedy rice | | | 2016WR41 | |  |  |  |  |  |  |  |
| 42 | WR144 | | weedy rice | | | 2016WR42 | |  |  |  |  |  |  |  |
| 43 | WR145 | | weedy rice | | | 2016WR43 | |  |  |  |  |  |  |  |
| 44 | WR146 | | weedy rice | | | 2016WR44 | |  |  |  |  |  |  |  |
| 45 | WR147 | | weedy rice | | | 2016WR45 | |  |  |  |  |  |  |  |
| 46 | WR148 | | weedy rice | | | 2016WR46 | |  |  |  |  |  |  |  |
| 47 | WR149 | | weedy rice | | | 2016WR47 | |  |  |  |  |  |  |  |
| 48 | WR150 | | weedy rice | | | 2016WR48 | |  |  |  |  |  |  |  |
| 49 | WR151 | | weedy rice | | | 2016WR49 | |  |  |  |  |  |  |  |
| 50 | WR152 | | weedy rice | | | 2016WR50 | |  |  |  |  |  |  |  |
| 51 | WR153 | | weedy rice | | | 2016WR51 | |  |  |  |  |  |  |  |
| 52 | WR154 | | weedy rice | | | 2016WR52 | |  |  |  |  |  |  |  |
| 53 | WR155 | | weedy rice | | | 2016WR53 | |  |  |  |  |  |  |  |
| 54 | WR156 | | weedy rice | | | 2016WR54 | |  |  |  |  |  |  |  |
| 55 | WR157 | | weedy rice | | | 2016WR55 | |  |  |  |  |  |  |  |
| 56 | WR158 | | weedy rice | | | 2016WR56 | |  |  |  |  |  |  |  |
| 57 | WR159 | | weedy rice | | | 2016WR57 | |  |  |  |  |  |  |  |
| 58 | WR16 | | weedy rice | | | 2016WR58 | |  |  |  |  |  |  |  |
| 59 | WR161 | | weedy rice | | | 2016WR59 | |  |  |  |  |  |  |  |
| 60 | WR162 | | weedy rice | | | 2016WR60 | |  |  |  |  |  |  |  |
| 61 | WR163 | | weedy rice | | | 2016WR61 | |  |  |  |  |  |  |  |
| 62 | WR164 | | weedy rice | | | 2016WR62 | |  |  |  |  |  |  |  |
| 63 | WR165 | | weedy rice | | | 2016WR63 | |  |  |  |  |  |  |  |
| 64 | WR166 | | weedy rice | | | 2016WR64 | |  |  |  |  |  |  |  |
| 65 | WR167 | | weedy rice | | | 2016WR65 | |  |  |  |  |  |  |  |
| 66 | WR168 | | weedy rice | | | 2016WR66 | |  |  |  |  |  |  |  |
| 67 | WR169 | | weedy rice | | | 2016WR67 | |  |  |  |  |  |  |  |
| 68 | WR170 | | weedy rice | | | 2016WR68 | |  |  |  |  |  |  |  |
| 69 | WR171 | | weedy rice | | | 2016WR69 | |  |  |  |  |  |  |  |
| 70 | WR172 | | weedy rice | | | 2016WR70 | |  |  |  |  |  |  |  |
| 71 | WR233 | | weedy rice | | | 2016WR71 | |  |  |  |  |  |  |  |
| 72 | WR234 | | weedy rice | | | 2016WR72 | |  |  |  |  |  |  |  |
| 73 | WR235 | | weedy rice | | | 2016WR73 | |  |  |  |  |  |  |  |
| 74 | WR236 | | weedy rice | | | 2016WR74 | |  |  |  |  |  |  |  |
| 75 | WR237 | | weedy rice | | | 2016WR75 | |  |  |  |  |  |  |  |
| 76 | WR238 | | weedy rice | | | 2016WR76 | |  |  |  |  |  |  |  |
| 77 | WR239 | | weedy rice | | | 2016WR77 | |  |  |  |  |  |  |  |
| 78 | WR240 | | weedy rice | | | 2016WR78 | |  |  |  |  |  |  |  |
| 79 | WR241 | | weedy rice | | | 2016WR79 | |  |  |  |  |  |  |  |
| 80 | WR242 | | weedy rice | | | 2016WR80 | |  |  |  |  |  |  |  |
| 81 | WR243 | | weedy rice | | | 2016WR81 | |  |  |  |  |  |  |  |
| 82 | WR244 | | weedy rice | | | 2016WR82 | |  |  |  |  |  |  |  |
| 83 | WR245 | | weedy rice | | | 2016WR83 | |  |  |  |  |  |  |  |
| 84 | WR246 | | weedy rice | | | 2016WR84 | |  |  |  |  |  |  |  |
| 85 | WR247 | | weedy rice | | | 2016WR85 | |  |  |  |  |  |  |  |
| 86 | WR248 | | weedy rice | | | 2016WR86 | |  |  |  |  |  |  |  |
| 87 | WR249 | | weedy rice | | | 2016WR87 | |  |  |  |  |  |  |  |
| 88 | WR250 | | weedy rice | | | 2016WR88 | |  |  |  |  |  |  |  |
| 89 | WR251 | | weedy rice | | | 2016WR89 | |  |  |  |  |  |  |  |
| 90 | WR252 | | weedy rice | | | 2016WR90 | |  |  |  |  |  |  |  |
| 91 | WR253 | | weedy rice | | | 2016WR91 | |  |  |  |  |  |  |  |
| 92 | WR254 | | weedy rice | | | 2016WR92 | |  |  |  |  |  |  |  |
| 93 | WR255 | | weedy rice | | | 2016WR93 | |  |  |  |  |  |  |  |
| 94 | WR298 | | weedy rice | | | 2016WR94 | |  |  |  |  |  |  |  |
| 95 | WR299 | | weedy rice | | | 2016WR95 | |  |  |  |  |  |  |  |
| 96 | WR300 | | weedy rice | | | 2016WR96 | |  |  |  |  |  |  |  |
| 97 | WR301 | | weedy rice | | | 2016WR97 | |  |  |  |  |  |  |  |
| 98 | WR302 | | weedy rice | | | 2016WR98 | |  |  |  |  |  |  |  |
| 99 | WR303 | | weedy rice | | | 2016WR99 | |  |  |  |  |  |  |  |
| 100 | WR304 | | weedy rice | | | 2016WR100 | |  |  |  |  |  |  |  |
| 101 | WR305 | | weedy rice | | | 2016WR101 | |  |  |  |  |  |  |  |
| 102 | WR306 | | weedy rice | | | 2016WR102 | |  |  |  |  |  |  |  |
| 103 | WR307 | | weedy rice | | | 2016WR103 | |  |  |  |  |  |  |  |
| 104 | WR393 | | weedy rice | | | 2016WR104 | |  |  |  |  |  |  |  |
| 105 | WR394 | | weedy rice | | | 2016WR105 | |  |  |  |  |  |  |  |
| 106 | WR395 | | weedy rice | | | 2016WR106 | |  |  |  |  |  |  |  |
| 107 | WR396 | | weedy rice | | | 2016WR107 | |  |  |  |  |  |  |  |
| 108 | WR397 | | weedy rice | | | 2016WR108 | |  |  |  |  |  |  |  |
| 109 | WR398 | | weedy rice | | | 2016WR109 | |  |  |  |  |  |  |  |
| 110 | WR399 | | weedy rice | | | 2016WR110 | |  |  |  |  |  |  |  |
| 111 | WR400 | | weedy rice | | | 2016WR111 | |  |  |  |  |  |  |  |
| 112 | WR401 | | weedy rice | | | 2016WR112 | |  |  |  |  |  |  |  |
| 113 | WR402 | | weedy rice | | | 2016WR113 | |  |  |  |  |  |  |  |
| 114 | WR403 | | weedy rice | | | 2016WR114 | |  |  |  |  |  |  |  |
| 115 | WR404 | | weedy rice | | | 2016WR115 | |  |  |  |  |  |  |  |
| 116 | WR405 | | weedy rice | | | 2016WR116 | |  |  |  |  |  |  |  |
| 117 | WR406 | | weedy rice | | | 2016WR117 | |  |  |  |  |  |  |  |
| 118 | WR407 | | weedy rice | | | 2016WR118 | |  |  |  |  |  |  |  |
| 119 | WR408 | | weedy rice | | | 2016WR119 | |  |  |  |  |  |  |  |
| 120 | WR409 | | weedy rice | | | 2016WR120 | |  |  |  |  |  |  |  |
| 121 | WR410 | | weedy rice | | | 2016WR121 | |  |  |  |  |  |  |  |
| 122 | WR411 | | weedy rice | | | 2016WR122 | |  |  |  |  |  |  |  |
| 123 | WR412 | | weedy rice | | | 2016WR123 | |  |  |  |  |  |  |  |
| 124 | WR413 | | weedy rice | | | 2016WR124 | |  |  |  |  |  |  |  |
| 125 | WR414 | | weedy rice | | | 2016WR125 | |  |  |  |  |  |  |  |
| 126 | WR415 | | weedy rice | | | 2016WR126 | |  |  |  |  |  |  |  |
| 127 | WR416 | | weedy rice | | | 2016WR127 | |  |  |  |  |  |  |  |
| 128 | WR417 | | weedy rice | | | 2016WR128 | |  |  |  |  |  |  |  |
| 129 | WR418 | | weedy rice | | | 2016WR129 | |  |  |  |  |  |  |  |
| 130 | WR419 | | weedy rice | | | 2016WR130 | |  |  |  |  |  |  |  |
| 131 | WR420 | | weedy rice | | | 2016WR131 | |  |  |  |  |  |  |  |
| 132 | WR421 | | weedy rice | | | 2016WR132 | |  |  |  |  |  |  |  |
| 133 | WR422 | | weedy rice | | | 2016WR133 | |  |  |  |  |  |  |  |
| 134 | WR14 | | weedy rice | | | 2016WR134 | |  |  |  |  |  |  |  |
| Table S3 the value of the ten evaluation index | | | | | | | | |  |  |  |  |  |  |
| Name | | PH | | SL | TN | | PL | | CC | NC | WR1-1 | WR1-2 | WR2-1 | WR2-2 |
| WR2 | | 0.06 | | 0.14 | 0.04 | | -0.38 | | 0.07 | 0.05 | 5.20 | 7.10 | 7.10 | 9.10 |
| WR3 | | 0.15 | | 0.29 | 0.39 | | -0.62 | | 0.05 | 0.03 | 5.20 | 7.10 | 7.10 | 9.10 |
| IAPAR-9 | | 0.30 | | 0.34 | 0.76 | | 0.12 | | 0.30 | 0.27 | 7.10 | 7.20 | 7.20 | 9.20 |
| WR5 | | 0.23 | | 0.32 | -0.16 | | -0.16 | | 0.08 | 0.06 | 5.20 | 7.20 | 7.10 | 9.10 |
| WR6 | | 0.23 | | 0.31 | 0.08 | | -0.16 | | 0.01 | 0.01 | 5.10 | 7.10 | 7.10 | 7.20 |
| WR7 | | 0.44 | | 0.48 | 0.41 | | 0.20 | | 0.07 | 0.05 | 5.10 | 7.20 | 7.10 | 9.10 |
| WR8 | | 0.09 | | 0.13 | 0.38 | | -0.10 | | -0.04 | -0.01 | 5.10 | 7.10 | 5.20 | 7.20 |
| WR9 | | 0.14 | | 0.21 | 0.45 | | -0.26 | | 0.01 | -0.15 | 5.10 | 7.10 | 5.20 | 7.20 |
| WR10 | | 0.13 | | 0.17 | 0.38 | | -0.04 | | 0.27 | 0.26 | 5.10 | 7.10 | 5.20 | 7.20 |
| WR11 | | 0.16 | | 0.19 | 0.34 | | 0.02 | | -0.15 | -0.14 | 1.20 | 5.20 | 1.20 | 1.20 |
| WR12 | | 0.21 | | 0.22 | 0.35 | | 0.17 | | -0.01 | -0.03 | 3.20 | 7.10 | 5.20 | 7.10 |
| WR13 | | 0.31 | | 0.37 | 0.37 | | -0.03 | | -0.07 | -0.05 | 3.20 | 7.10 | 5.20 | 7.10 |
| WR15 | | 0.19 | | 0.21 | 0.65 | | 0.11 | | 0.04 | 0.02 | 5.20 | 5.20 | 5.20 | 5.20 |
| WR160 | | 0.11 | | 0.12 | 0.57 | | 0.05 | | 0.49 | 0.13 | 3.10 | 5.20 | 3.10 | 3.10 |
| WR17 | | 0.18 | | 0.20 | 0.62 | | 0.09 | | 0.07 | 0.06 | 3.10 | 5.20 | 3.10 | 7.10 |
| WR18 | | 0.13 | | 0.17 | 0.22 | | -0.10 | | -0.04 | -0.10 | 7.10 | 7.10 | 7.20 | 7.20 |
| WR19 | | 0.07 | | 0.09 | 0.26 | | 0.00 | | -0.02 | 0.02 | 5.20 | 5.20 | 7.10 | 5.20 |
| WR20 | | 0.02 | | 0.05 | -0.11 | | -0.08 | | -0.01 | -0.11 | 5.20 | 5.20 | 5.20 | 5.20 |
| WR21 | | 0.04 | | 0.06 | -0.38 | | -0.07 | | 0.03 | 0.03 | 7.10 | 7.10 | 7.10 | 9.10 |
| WR22 | | -0.12 | | -0.15 | -0.04 | | -0.05 | | -0.04 | -0.04 | 1.20 | 3.10 | 1.20 | 3.10 |
| WR23 | | 0.27 | | 0.30 | 0.10 | | 0.18 | | 0.04 | 0.05 | 1.10 | 1.10 | 1.10 | 1.10 |
| WR24 | | 0.04 | | 0.04 | -0.03 | | 0.04 | | -0.06 | -0.04 | 3.10 | 7.10 | 3.10 | 7.20 |
| WR25 | | 0.10 | | 0.16 | 0.11 | | -0.17 | | -0.12 | -0.11 | 3.10 | 7.10 | 3.10 | 7.10 |
| WR26 | | 0.10 | | 0.16 | -0.48 | | -0.17 | | -0.13 | -0.13 | 3.10 | 7.10 | 5.20 | 7.10 |
| WR27 | | 0.12 | | 0.15 | 0.22 | | -0.01 | | -0.01 | 0.04 | 3.20 | 7.10 | 5.20 | 7.10 |
| WR28 | | 0.21 | | 0.28 | 0.00 | | -0.15 | | -0.15 | -0.11 | 1.20 | 7.10 | 3.10 | 7.20 |
| WR29 | | -0.03 | | 0.06 | -0.34 | | -0.38 | | 0.07 | 0.05 | 1.20 | 7.10 | 5.10 | 7.20 |
| WR30 | | 0.08 | | 0.18 | -0.30 | | -0.44 | | 0.02 | 0.00 | 7.10 | 7.20 | 7.10 | 9.10 |
| WR31 | | 0.05 | | 0.13 | -0.47 | | -0.32 | | 0.01 | 0.02 | 7.10 | 7.20 | 7.10 | 9.10 |
| WR32 | | 0.04 | | 0.04 | 0.30 | | 0.04 | | 0.21 | 0.22 | 5.20 | 7.20 | 5.20 | 7.20 |
| WR33 | | 0.09 | | 0.13 | -0.36 | | -0.04 | | 0.01 | 0.02 | 7.10 | 7.20 | 7.10 | 9.10 |
| WR94 | | 0.14 | | 0.15 | 0.40 | | 0.06 | | 0.62 | 0.56 | 5.20 | 7.10 | 7.10 | 7.10 |
| WR95 | | 0.11 | | 0.14 | 0.08 | | 0.00 | | -0.40 | -0.28 | 5.10 | 7.10 | 5.20 | 7.10 |
| WR96 | | 0.14 | | 0.15 | 0.29 | | 0.09 | | -0.11 | 0.19 | 5.10 | 7.10 | 7.10 | 7.10 |
| WR97 | | 0.19 | | 0.22 | 0.33 | | 0.09 | | 0.15 | 0.21 | 5.10 | 7.10 | 7.10 | 7.10 |
| WR98 | | 0.14 | | 0.21 | 0.45 | | -0.20 | | -0.09 | -0.10 | 5.10 | 7.10 | 7.10 | 7.10 |
| WR99 | | 0.09 | | 0.17 | 0.22 | | -0.23 | | 0.14 | 0.01 | 5.10 | 7.10 | 7.10 | 7.10 |
| WR100 | | 0.14 | | 0.15 | 0.36 | | 0.10 | | 0.75 | 0.63 | 7.10 | 7.20 | 9.10 | 9.10 |
| WR101 | | 0.13 | | 0.16 | -0.08 | | -0.05 | | 0.69 | 0.79 | 7.10 | 7.20 | 9.10 | 9.10 |
| WR102 | | 0.08 | | 0.08 | 0.24 | | 0.05 | | 0.91 | 0.87 | 7.10 | 7.20 | 9.10 | 9.10 |
| WR143 | | -0.04 | | 0.07 | 0.51 | | -0.52 | | 0.15 | 0.13 | 5.10 | 5.10 | 7.10 | 5.10 |
| WR144 | | 0.38 | | 0.48 | 0.78 | | -0.09 | | 0.04 | 0.05 | 5.10 | 7.20 | 5.20 | 7.20 |
| WR145 | | 0.26 | | 0.36 | 0.27 | | -0.17 | | 0.11 | 0.10 | 5.10 | 7.20 | 5.20 | 7.20 |
| WR146 | | 0.32 | | 0.38 | 0.17 | | 0.06 | | 0.07 | 0.07 | 5.10 | 5.20 | 5.20 | 5.20 |
| WR147 | | 0.26 | | 0.36 | 0.39 | | -0.25 | | 0.22 | 0.18 | 1.20 | 3.20 | 3.10 | 3.20 |
| WR148 | | 0.51 | | 0.57 | 0.80 | | 0.21 | | 0.08 | 0.05 | 5.10 | 5.20 | 5.20 | 5.20 |
| WR149 | | 0.34 | | 0.44 | 0.40 | | -0.21 | | 0.13 | 0.14 | 1.20 | 3.20 | 3.10 | 3.20 |
| WR150 | | 0.35 | | 0.47 | 0.77 | | -0.29 | | 0.08 | 0.09 | 5.10 | 5.10 | 5.20 | 5.10 |
| WR151 | | 0.30 | | 0.38 | 0.32 | | -0.06 | | 0.00 | 0.03 | 5.10 | 7.10 | 5.20 | 7.10 |
| WR152 | | 0.27 | | 0.41 | 0.84 | | -0.39 | | 0.10 | 0.07 | 3.20 | 7.10 | 5.20 | 7.10 |
| WR153 | | 0.52 | | 0.61 | 0.83 | | 0.11 | | 0.07 | 0.07 | 3.20 | 7.20 | 5.20 | 7.20 |
| WR154 | | 0.49 | | 0.58 | 0.86 | | 0.10 | | 0.06 | 0.09 | 3.20 | 9.10 | 5.20 | 9.10 |
| WR155 | | 0.47 | | 0.51 | 0.56 | | 0.29 | | 0.04 | 0.03 | 3.20 | 7.20 | 5.20 | 7.20 |
| WR156 | | 0.45 | | 0.54 | 0.24 | | 0.07 | | 0.23 | 0.16 | 3.20 | 7.20 | 5.20 | 7.20 |
| WR157 | | 0.66 | | 0.70 | 0.91 | | 0.48 | | 0.02 | 0.03 | 3.20 | 9.10 | 5.20 | 9.10 |
| WR158 | | 0.24 | | 0.34 | 0.51 | | -0.26 | | 0.05 | 0.07 | 3.20 | 9.10 | 5.20 | 9.10 |
| WR159 | | NA | | NA | NA | | NA | | 0.06 | 0.05 | 3.20 | 9.10 | 5.20 | 9.10 |
| WR16 | | 0.51 | | 0.59 | 0.82 | | 0.13 | | 0.27 | 0.24 | 5.10 | 9.10 | 5.20 | 9.10 |
| WR161 | | 0.52 | | 0.57 | 0.69 | | 0.31 | | 0.33 | 0.28 | 5.10 | 9.10 | 5.20 | 9.10 |
| WR162 | | 0.47 | | 0.55 | 0.57 | | 0.13 | | 0.09 | 0.08 | 5.10 | 7.20 | 5.20 | 7.20 |
| WR163 | | 0.48 | | 0.58 | 0.88 | | -0.08 | | -0.15 | -0.13 | 5.10 | 9.10 | 5.20 | 9.10 |
| WR164 | | 0.36 | | 0.44 | 0.64 | | -0.04 | | 0.09 | 0.09 | 5.10 | 7.10 | 7.10 | 7.10 |
| WR165 | | 0.35 | | 0.42 | 0.52 | | 0.09 | | 0.22 | 0.19 | 5.10 | 7.10 | 7.10 | 7.10 |
| WR166 | | 0.53 | | 0.63 | 0.78 | | 0.04 | | -0.01 | 0.00 | 3.10 | 7.10 | 5.10 | 7.10 |
| WR167 | | 0.53 | | 0.66 | 0.78 | | -0.23 | | 0.02 | 0.07 | 3.10 | 5.20 | 5.10 | 5.20 |
| WR168 | | 0.29 | | 0.34 | 0.48 | | 0.08 | | 0.05 | 0.04 | 3.10 | 5.20 | 5.20 | 5.20 |
| WR169 | | 0.22 | | 0.26 | 0.60 | | 0.08 | | 0.05 | 0.04 | 3.10 | 5.20 | 5.10 | 5.20 |
| WR170 | | 0.42 | | 0.55 | 0.68 | | -0.14 | | 0.11 | 0.10 | 3.10 | 5.20 | 5.20 | 5.20 |
| WR171 | | 0.47 | | 0.57 | 0.71 | | 0.02 | | 0.10 | 0.10 | 5.10 | 7.10 | 7.10 | 7.10 |
| WR172 | | 0.35 | | 0.40 | 0.33 | | 0.17 | | 0.08 | 0.11 | 3.10 | 3.10 | 5.10 | 3.10 |
| WR233 | | 0.05 | | 0.04 | 0.10 | | 0.08 | | 0.30 | 0.27 | 3.10 | 5.20 | 3.10 | 5.20 |
| WR234 | | 0.35 | | 0.43 | -0.15 | | 0.10 | | 0.13 | 0.12 | 3.10 | 7.20 | 3.10 | 7.20 |
| WR235 | | 0.23 | | 0.27 | 0.02 | | 0.10 | | 0.40 | 0.36 | 3.10 | 7.10 | 3.10 | 7.10 |
| WR236 | | -0.01 | | 0.00 | 0.21 | | -0.02 | | 0.24 | 0.20 | 3.10 | 7.20 | 3.10 | 7.20 |
| WR237 | | -0.15 | | -0.16 | 0.13 | | -0.12 | | 0.21 | 0.17 | 3.20 | 7.20 | 3.20 | 7.20 |
| WR238 | | 0.07 | | 0.08 | 0.24 | | 0.04 | | 0.14 | 0.12 | 3.20 | 7.20 | 3.20 | 7.20 |
| WR239 | | 0.05 | | 0.09 | 0.05 | | -0.11 | | 0.25 | 0.34 | 3.20 | 7.20 | 3.20 | 7.20 |
| WR240 | | 0.03 | | 0.08 | 0.34 | | -0.15 | | 0.23 | 0.31 | 3.20 | 7.20 | 3.20 | 7.20 |
| WR241 | | 0.02 | | -0.06 | 0.13 | | 0.23 | | 0.45 | 0.44 | 7.10 | 7.20 | 7.10 | 7.20 |
| WR242 | | -0.03 | | -0.01 | 0.25 | | -0.10 | | 0.41 | 0.41 | 7.10 | 7.20 | 7.10 | 7.20 |
| WR243 | | 0.05 | | 0.10 | 0.30 | | -0.13 | | 0.53 | 0.46 | 7.10 | 7.20 | 7.20 | 7.20 |
| WR244 | | -0.07 | | -0.06 | 0.34 | | -0.09 | | 0.72 | 0.71 | 7.20 | 7.20 | 9.10 | 7.20 |
| WR245 | | -0.04 | | 0.01 | -0.25 | | -0.21 | | 0.26 | 0.23 | 3.10 | 7.10 | 3.20 | 7.10 |
| WR246 | | 0.06 | | 0.10 | 0.35 | | -0.08 | | 0.40 | 0.40 | 5.10 | 7.10 | 5.20 | 7.10 |
| WR247 | | -0.05 | | -0.06 | -0.17 | | -0.04 | | 0.50 | 0.48 | 7.10 | 7.10 | 7.10 | 7.10 |
| WR248 | | -0.03 | | -0.02 | -0.30 | | -0.04 | | 0.39 | 0.39 | 7.10 | 7.10 | 7.10 | 7.10 |
| WR249 | | 0.04 | | 0.05 | -0.36 | | -0.03 | | 0.51 | 0.49 | 5.20 | 5.20 | 7.10 | 5.20 |
| WR250 | | 0.01 | | 0.11 | 0.19 | | -0.36 | | 0.39 | 0.24 | 5.10 | 7.20 | 5.20 | 7.20 |
| WR251 | | 0.17 | | 0.19 | -0.24 | | 0.09 | | 0.73 | 0.72 | 7.10 | 7.20 | 7.10 | 7.20 |
| WR252 | | 0.14 | | 0.20 | 0.07 | | -0.09 | | 0.45 | 0.44 | 5.20 | 7.20 | 7.10 | 7.20 |
| WR253 | | 0.00 | | 0.00 | -0.33 | | -0.01 | | 0.41 | 0.40 | 7.10 | 7.10 | 7.20 | 7.10 |
| WR254 | | 0.37 | | 0.54 | -0.39 | | -0.49 | | 0.14 | 0.12 | 5.20 | 5.20 | 7.10 | 5.20 |
| WR255 | | 0.28 | | 0.38 | -0.90 | | -0.13 | | 0.11 | 0.08 | 5.20 | 5.20 | 7.10 | 5.20 |
| WR298 | | 0.23 | | 0.41 | 0.15 | | -0.22 | | -0.03 | -0.03 | 3.20 | 3.20 | 5.20 | 3.20 |
| WR299 | | 0.44 | | 0.54 | 0.33 | | 0.19 | | 0.04 | 0.02 | 3.20 | 3.20 | 5.10 | 3.20 |
| WR300 | | NA | | NA | NA | | NA | | -0.05 | -0.03 | 3.20 | 3.20 | 5.10 | 3.20 |
| WR301 | | 0.43 | | 0.54 | 0.90 | | 0.12 | | 0.02 | 0.01 | 3.20 | 5.20 | 5.10 | 5.20 |
| WR302 | | NA | | NA | NA | | NA | | -0.11 | -0.10 | 5.20 | 5.20 | 5.20 | 5.20 |
| WR303 | | NA | | NA | NA | | NA | | 0.05 | 0.05 | 5.20 | 5.20 | 5.20 | 5.20 |
| WR304 | | 0.43 | | 0.56 | 0.84 | | 0.00 | | 0.13 | 0.10 | 5.20 | 5.20 | 5.20 | 5.20 |
| WR305 | | NA | | NA | NA | | NA | | -0.10 | -0.09 | 5.20 | 5.20 | 5.20 | 5.20 |
| WR306 | | NA | | NA | NA | | NA | | -0.04 | -0.03 | 5.20 | 5.20 | 5.20 | 5.20 |
| WR307 | | NA | | NA | NA | | NA | | -0.07 | -0.02 | 5.20 | 5.20 | 5.20 | 5.20 |
| WR393 | | 0.20 | | 0.25 | 0.51 | | 0.04 | | -0.06 | -0.05 | 7.10 | 7.10 | 7.10 | 7.10 |
| WR394 | | 0.21 | | 0.20 | 0.20 | | 0.27 | | -0.24 | -0.19 | 7.10 | 9.10 | 7.10 | 9.10 |
| WR395 | | 0.29 | | 0.33 | 0.19 | | 0.11 | | 0.12 | 0.14 | 5.10 | 5.20 | 5.10 | 5.20 |
| WR396 | | NA | | NA | NA | | NA | | -0.04 | -0.08 | 7.10 | 7.10 | 7.10 | 7.10 |
| WR397 | | 0.23 | | 0.41 | 0.66 | | -17.25 | | 0.02 | 0.08 | 7.10 | 7.10 | 7.10 | 7.10 |
| WR398 | | 0.27 | | 0.31 | 0.38 | | 0.07 | | -0.21 | 0.02 | 7.10 | 7.10 | 7.10 | 7.10 |
| WR399 | | 0.27 | | 0.30 | 0.50 | | 0.09 | | 0.00 | 0.01 | 7.10 | 7.10 | 7.10 | 7.10 |
| WR400 | | NA | | NA | NA | | NA | | -0.27 | -0.23 | 3.10 | 5.20 | 5.10 | 5.20 |
| WR401 | | NA | | NA | NA | | NA | | -0.10 | -0.06 | 3.10 | 5.20 | 5.10 | 5.20 |
| WR402 | | NA | | NA | NA | | NA | | -0.04 | -0.04 | 3.10 | 5.30 | 5.20 | 5.30 |
| WR403 | | 0.39 | | 0.42 | 0.47 | | 0.24 | | -0.07 | -0.07 | 3.10 | 7.10 | 5.20 | 7.10 |
| WR404 | | 0.31 | | 0.34 | 0.54 | | 0.20 | | 0.18 | 0.15 | 7.10 | 7.10 | 7.20 | 7.10 |
| WR405 | | 0.42 | | 0.46 | 0.48 | | 0.30 | | 0.07 | 0.06 | 3.10 | 5.30 | 7.10 | 5.30 |
| WR406 | | NA | | NA | NA | | NA | | 0.03 | 0.01 | 3.10 | 7.10 | 5.10 | 7.10 |
| WR407 | | NA | | NA | NA | | NA | | -0.16 | -0.13 | 3.10 | 7.10 | 5.10 | 7.10 |
| WR408 | | 0.28 | | 0.34 | 0.63 | | 0.05 | | -0.11 | -0.13 | 7.10 | 7.20 | 7.20 | 7.20 |
| WR409 | | NA | | NA | NA | | NA | | -0.21 | -0.16 | 7.10 | 7.10 | 7.10 | 7.10 |
| WR410 | | 0.32 | | 0.38 | 0.37 | | 0.02 | | -0.28 | -0.23 | 7.10 | 7.10 | 7.20 | 7.10 |
| WR411 | | 0.44 | | 0.52 | 0.68 | | 0.12 | | 0.11 | 0.11 | 7.10 | 9.10 | 7.20 | 9.10 |
| WR412 | | 0.27 | | 0.34 | 0.50 | | -0.03 | | -0.10 | -0.05 | 7.10 | 9.10 | 7.20 | 9.10 |
| WR413 | | 0.31 | | 0.25 | 0.46 | | 0.45 | | -0.03 | -0.03 | 5.20 | 7.10 | 7.10 | 7.10 |
| WR414 | | NA | | NA | NA | | NA | | 0.07 | 0.04 | 5.20 | 3.10 | 5.20 | 3.10 |
| WR415 | | 0.29 | | 0.33 | 0.06 | | 0.13 | | 0.02 | 0.02 | 7.10 | 3.10 | 7.10 | 3.10 |
| WR416 | | 0.40 | | 0.45 | 0.51 | | 0.09 | | -0.01 | 0.00 | 7.10 | 3.10 | 7.10 | 3.10 |
| WR417 | | 0.26 | | 0.30 | 0.36 | | 0.11 | | -0.07 | -0.07 | 7.10 | 7.10 | 7.10 | 7.10 |
| WR418 | | 0.35 | | 0.31 | 0.51 | | 0.44 | | 0.05 | 0.02 | 7.10 | 3.10 | 7.10 | 3.10 |
| WR419 | | 0.24 | | 0.26 | 0.31 | | 0.17 | | -0.06 | -0.04 | 7.10 | 7.10 | 7.20 | 7.10 |
| WR420 | | NA | | NA | NA | | NA | | -0.14 | -0.13 | 5.10 | 5.20 | 5.20 | 5.20 |
| WR421 | | 0.21 | | 0.26 | 0.24 | | -0.01 | | 0.62 | 0.60 | 7.10 | 7.20 | 7.10 | 7.20 |
| WR422 | | 0.19 | | 0.24 | 0.36 | | -0.02 | | 0.26 | 0.27 | 7.10 | 7.20 | 7.10 | 7.20 |
| WR14 | | 0.35 | | 0.19 | 0.20 | | 1.00 | | 0.17 | 0.20 | 1.00 | 1.00 | 1.00 | 1.00 |

| Table S4 The F factor of each accession | | |  |  |
| --- | --- | --- | --- | --- |
| Name | F(1) | F(2) | F(3) | F(4) |
| WR2 | 0.22960027 | -0.0717965 | -0.0116095 | 0.02405169 |
| WR3 | 0.16804999 | 0.12488123 | 0.02019329 | 0.0076751 |
| IAPAR-9 | 0.34888017 | 0.28628735 | 0.04629266 | -0.0040401 |
| WR5 | 0.19747272 | 0.05605491 | 0.00906408 | 0.02278453 |
| WR6 | 0.07022416 | 0.07248528 | 0.01172087 | -0.0239929 |
| WR7 | 0.10617833 | 0.3584127 | 0.05795533 | 0.02712972 |
| WR8 | -0.0057994 | -0.0408183 | -0.0066003 | 0.03922436 |
| WR9 | -0.0545398 | 0.04753027 | 0.00768565 | 0.02681016 |
| WR10 | 0.14379587 | -0.0328504 | -0.0053119 | 0.06023907 |
| WR11 | -0.751586 | -0.2117641 | -0.0342423 | 0.05406304 |
| WR12 | -0.1181938 | 0.04326537 | 0.00699601 | 0.09178056 |
| WR13 | -0.1746647 | 0.16934294 | 0.02738275 | 0.07415568 |
| WR15 | -0.1673567 | 0.01459701 | 0.00236034 | -0.0604266 |
| WR16 | -0.2503819 | -0.2046352 | -0.0330895 | 0.03378288 |
| WR17 | -0.250364 | -0.0113601 | -0.0018369 | 0.10101244 |
| WR18 | 0.13386927 | 0.01765003 | 0.00285401 | -0.0783455 |
| WR19 | -0.0467497 | -0.1478863 | -0.0239132 | -0.1130682 |
| WR20 | -0.1377881 | -0.2947939 | -0.0476682 | -0.0658814 |
| WR21 | 0.32122792 | -0.191377 | -0.0309457 | -0.0194702 |
| WR22 | -0.596337 | -0.6215603 | -0.1005063 | 0.05720543 |
| WR23 | -0.8395655 | -0.2901302 | -0.046914 | -0.0555754 |
| WR24 | -0.1613073 | -0.2386483 | -0.0385894 | 0.15852522 |
| WR25 | -0.233659 | -0.1094659 | -0.0177006 | 0.13856796 |
| WR26 | -0.1170363 | -0.212036 | -0.0342862 | 0.07787284 |
| WR27 | -0.0676294 | -0.0714806 | -0.0115584 | 0.09322415 |
| WR28 | -0.343417 | -0.0329333 | -0.0053253 | 0.18538864 |
| WR29 | -0.0607052 | -0.3274138 | -0.0529428 | 0.1508135 |
| WR30 | 0.28958242 | -0.087406 | -0.0141335 | -0.0350412 |
| WR31 | 0.31225054 | -0.1643711 | -0.0265788 | -0.0284646 |
| WR32 | 0.16275044 | -0.1532285 | -0.024777 | 0.0670032 |
| WR33 | 0.29836341 | -0.1210539 | -0.0195744 | -0.0203867 |
| WR94 | 0.41741717 | -0.0564874 | -0.009134 | 0.03210628 |
| WR95 | -0.1817199 | -0.0482838 | -0.0078075 | 0.01345945 |
| WR96 | 0.11015969 | -0.0057927 | -0.0009367 | -0.0075342 |
| WR97 | 0.16820472 | 0.04240824 | 0.00685741 | 6.5466E-05 |
| WR98 | 0.01274747 | 0.0782521 | 0.01265336 | -0.0286354 |
| WR99 | 0.13407161 | -0.0420069 | -0.0067925 | -0.0135427 |
| WR100 | 0.73008726 | 0.00302195 | 0.00048865 | -0.0182685 |
| WR101 | 0.78658796 | -0.1048022 | -0.0169465 | -0.0206208 |
| WR102 | 0.87118427 | -0.1129825 | -0.0182693 | -0.0005493 |
| WR143 | 0.03128248 | -0.1820652 | -0.0294399 | -0.1167787 |
| WR144 | -0.0709502 | 0.35279089 | 0.05704629 | 0.02873975 |
| WR145 | 0.02255505 | 0.11424097 | 0.01847277 | 0.03680765 |
| WR146 | -0.1644346 | 0.04805594 | 0.00777065 | -0.0673117 |
| WR147 | -0.5051974 | -0.1036137 | -0.0167543 | -0.0043607 |
| WR148 | -0.2521137 | 0.36671204 | 0.05929734 | -0.0726377 |
| WR149 | -0.565849 | -0.0146255 | -0.0023649 | -0.0137113 |
| WR150 | -0.2051736 | 0.22877974 | 0.03699368 | -0.0847046 |
| WR151 | -0.0494634 | 0.16285883 | 0.02633427 | 0.02620875 |
| WR152 | -0.108779 | 0.2536959 | 0.04102263 | 0.07545058 |
| WR153 | -0.175611 | 0.47403128 | 0.07665086 | 0.08162883 |
| WR154 | -0.016949 | 0.53700533 | 0.08683376 | 0.18423355 |
| WR155 | -0.1650238 | 0.34820748 | 0.05630515 | 0.08848394 |
| WR156 | -0.056343 | 0.25937468 | 0.04194089 | 0.09277366 |
| WR157 | -0.0907618 | 0.687722 | 0.11120465 | 0.18392952 |
| WR158 | 0.06224063 | 0.22374316 | 0.03617927 | 0.18563053 |
| WR159 | 0.15971533 | -0.1640008 | -0.0265189 | 0.21053883 |
| WR160 | 0.16187859 | 0.53088116 | 0.08584348 | 0.14548077 |
| WR161 | 0.19718111 | 0.49009395 | 0.07924819 | 0.15535399 |
| WR162 | -0.06122 | 0.37198168 | 0.06014944 | 0.03355319 |
| WR163 | -0.057929 | 0.57754654 | 0.09338928 | 0.1095963 |
| WR164 | 0.04985915 | 0.30591058 | 0.04946574 | -0.0220566 |
| WR165 | 0.12169014 | 0.24911767 | 0.04028233 | -0.0084807 |
| WR166 | -0.2398542 | 0.48369529 | 0.07821353 | 0.07191761 |
| WR167 | -0.363297 | 0.41076721 | 0.06642106 | -0.0346029 |
| WR168 | -0.2683872 | 0.07137698 | 0.01154166 | -0.0098022 |
| WR169 | -0.2567348 | 0.02394252 | 0.0038715 | -0.0021238 |
| WR170 | -0.2900109 | 0.27391472 | 0.04429201 | -0.0233127 |
| WR171 | 0.01667526 | 0.43524834 | 0.07037966 | -0.0269866 |
| WR172 | -0.4217526 | -0.0019577 | -0.0003166 | -0.1164703 |
| WR233 | -0.1332712 | -0.3319221 | -0.0536718 | 0.08243662 |
| WR234 | -0.1465096 | 0.04827864 | 0.00780666 | 0.15428877 |
| WR235 | 0.022317 | -0.0840526 | -0.0135913 | 0.17843145 |
| WR236 | -0.0013777 | -0.2597812 | -0.0420066 | 0.18398389 |
| WR237 | 0.03454969 | -0.413916 | -0.0669302 | 0.18181573 |
| WR238 | -0.0674807 | -0.1642261 | -0.0265554 | 0.16871912 |
| WR239 | 0.03854592 | -0.2294323 | -0.0370992 | 0.17569295 |
| WR240 | 0.01685975 | -0.1801593 | -0.0291318 | 0.17441691 |
| WR241 | 0.47817892 | -0.2350463 | -0.038007 | -0.0110505 |
| WR242 | 0.45881074 | -0.2023746 | -0.032724 | -0.0242403 |
| WR243 | 0.4801934 | -0.1120218 | -0.0181139 | -0.0273705 |
| WR244 | 0.73804104 | -0.2440345 | -0.0394604 | -0.0547699 |
| WR245 | 0.03500793 | -0.3758478 | -0.0607746 | 0.17044788 |
| WR246 | 0.24213647 | -0.1343692 | -0.0217275 | 0.07226936 |
| WR247 | 0.52330092 | -0.3482878 | -0.0563181 | -0.020663 |
| WR248 | 0.46511008 | -0.3350217 | -0.054173 | -0.0305994 |
| WR249 | 0.28117337 | -0.3925604 | -0.063477 | -0.0747427 |
| WR250 | 0.21463598 | -0.1715104 | -0.0277332 | 0.0624039 |
| WR251 | 0.60757289 | -0.1640241 | -0.0265227 | -0.0076751 |
| WR252 | 0.35376908 | -0.0807665 | -0.0130599 | 0.01869343 |
| WR253 | 0.47145109 | -0.315717 | -0.0510514 | -0.0331216 |
| WR254 | -0.0379091 | 0.04832525 | 0.00781419 | -0.1447373 |
| WR255 | 1.4878E-05 | -0.183769 | -0.0297155 | -0.1289832 |
| WR298 | -0.4488603 | -0.073995 | -0.011965 | -0.1366572 |
| WR299 | -0.4785003 | 0.11960124 | 0.01933952 | -0.1258098 |
| WR300 | -0.3661922 | -0.4107025 | -0.0664106 | -0.1071286 |
| WR301 | -0.3545487 | 0.3287914 | 0.05316557 | -0.0209626 |
| WR302 | -0.1566981 | -0.2990562 | -0.0483574 | -0.0640206 |
| WR303 | -0.0682275 | -0.3201761 | -0.0517725 | -0.0520258 |
| WR304 | -0.2062448 | 0.32717297 | 0.05290387 | -0.0756891 |
| WR305 | -0.1529577 | -0.2999599 | -0.0485035 | -0.0634969 |
| WR306 | -0.1150307 | -0.3089843 | -0.0499628 | -0.0583994 |
| WR307 | -0.1207885 | -0.3075135 | -0.0497249 | -0.0593325 |
| WR393 | 0.10222674 | 0.14339874 | 0.02318758 | -0.0755982 |
| WR394 | 0.19061694 | 0.16635984 | 0.02690039 | 0.02642956 |
| WR395 | -0.123904 | 0.00109536 | 0.00017712 | -0.0555843 |
| WR396 | 0.18465978 | -0.1818839 | -0.0294106 | -0.0645599 |
| WR397 | 0.23511406 | 0.35686417 | 0.05770494 | -0.6319682 |
| WR398 | 0.07070918 | 0.18218168 | 0.02945878 | -0.0824599 |
| WR399 | 0.12052965 | 0.19231958 | 0.03109808 | -0.0729318 |
| WR400 | -0.3320353 | -0.2951823 | -0.047731 | -0.0145535 |
| WR401 | -0.2351438 | -0.3182988 | -0.0514689 | -0.0014392 |
| WR402 | -0.2003799 | -0.3179 | -0.0514044 | 0.004242 |
| WR403 | -0.2086253 | 0.25392378 | 0.04105948 | 0.08169652 |
| WR404 | 0.19933247 | 0.21988739 | 0.03555579 | -0.0625149 |
| WR405 | -0.1994932 | 0.22366029 | 0.03616587 | -0.0564786 |
| WR406 | -0.0275541 | -0.2488768 | -0.0402434 | 0.10727169 |
| WR407 | -0.1218538 | -0.2263042 | -0.0365934 | 0.09439145 |
| WR408 | 0.04516068 | 0.2676792 | 0.04328373 | -0.0830968 |
| WR409 | 0.11348343 | -0.1646948 | -0.0266311 | -0.0745185 |
| WR410 | -0.0342224 | 0.25947567 | 0.04195722 | -0.1028666 |
| WR411 | 0.28361336 | 0.48848587 | 0.07898817 | 0.02794639 |
| WR412 | 0.23354592 | 0.30956437 | 0.05005656 | 0.01864239 |
| WR413 | 0.01725848 | 0.17212665 | 0.02783288 | -0.0115631 |
| WR414 | -0.2334239 | -0.4124115 | -0.0666869 | -0.1632077 |
| WR415 | -0.1778605 | -0.0548171 | -0.0088639 | -0.2866019 |
| WR416 | -0.2448264 | 0.154531 | 0.02498766 | -0.2952202 |
| WR417 | 0.08477187 | 0.16334048 | 0.02641216 | -0.0784698 |
| WR418 | -0.1979458 | 0.05926071 | 0.00958246 | -0.2744019 |
| WR419 | 0.10941876 | 0.1223228 | 0.0197796 | -0.076214 |
| WR420 | -0.1768221 | -0.2959565 | -0.0478562 | -0.0633992 |
| WR421 | 0.50191231 | 0.01001878 | 0.00162004 | -0.0219812 |
| WR422 | 0.3057667 | 0.06560498 | 0.01060833 | -0.0474088 |
| WR14 | -0.7822644 | -0.3109808 | -0.0502856 | -0.0157896 |

Table S5 the subordinate function values for each sample

| Name | μ(1) | μ(2） | μ(3) | μ(4) |
| --- | --- | --- | --- | --- |
| WR2 | 0.624968009 | 0.419897595 | 0.418245978 | 1.24655008 |
| WR3 | 0.588989344 | 0.570115809 | 0.567873752 | 1.42415192 |
| IAPAR-9 | 0.694692001 | 0.693394348 | 0.690667737 | 1.56990319 |
| WR5 | 0.606188137 | 0.517547741 | 0.515512305 | 1.3620011 |
| WR6 | 0.531806138 | 0.530096911 | 0.52801215 | 1.37683789 |
| WR7 | 0.552822827 | 0.748482139 | 0.745539002 | 1.63503306 |
| WR8 | 0.487367225 | 0.443558071 | 0.441813456 | 1.27452368 |
| WR9 | 0.458876505 | 0.511036808 | 0.509026964 | 1.35430327 |
| WR10 | 0.574811819 | 0.449643776 | 0.44787524 | 1.28171876 |
| WR11 | 0.051424424 | 0.312993301 | 0.311761878 | 1.12015796 |
| WR12 | 0.421668112 | 0.507779364 | 0.505782323 | 1.35045202 |
| WR13 | 0.388658534 | 0.604074714 | 0.601699179 | 1.46430127 |
| WR15 | 0.392930403 | 0.48588309 | 0.483972113 | 1.32456422 |
| WR16 | 0.344398775 | 0.318438197 | 0.317185372 | 1.12659542 |
| WR17 | 0.344409211 | 0.466057576 | 0.464224525 | 1.30112467 |
| WR18 | 0.569009313 | 0.488214921 | 0.486294779 | 1.32732113 |
| WR19 | 0.46343015 | 0.361781785 | 0.360358595 | 1.17784021 |
| WR20 | 0.410214425 | 0.249576974 | 0.248594812 | 1.04518132 |
| WR21 | 0.678528127 | 0.328564556 | 0.327271929 | 1.13856773 |
| WR22 | 0.142173803 | -2.09657E-07 | -1.39446E-06 | 0.75010814 |
| WR23 | -3.23836E-06 | 0.253139 | 0.252142838 | 1.04939268 |
| WR24 | 0.396466522 | 0.292459734 | 0.291309019 | 1.09588128 |
| WR25 | 0.354173979 | 0.391126471 | 0.389587941 | 1.2125342 |
| WR26 | 0.422344723 | 0.312785631 | 0.311555024 | 1.11991243 |
| WR27 | 0.45122506 | 0.420138887 | 0.418486322 | 1.24683536 |
| WR28 | 0.290015939 | 0.449580486 | 0.447812199 | 1.28164394 |
| WR29 | 0.455272551 | 0.22466257 | 0.223778335 | 1.01572521 |
| WR30 | 0.660030032 | 0.407975389 | 0.406370633 | 1.23245455 |
| WR31 | 0.673280477 | 0.349191081 | 0.34781738 | 1.16295431 |
| WR32 | 0.585891543 | 0.357701574 | 0.356294422 | 1.1730162 |
| WR33 | 0.665162884 | 0.382275796 | 0.380772054 | 1.20207012 |
| WR94 | 0.734754685 | 0.431590376 | 0.4298928 | 1.26037437 |
| WR95 | 0.384534511 | 0.437856061 | 0.436133857 | 1.26778224 |
| WR96 | 0.555150091 | 0.470309856 | 0.468460091 | 1.30615211 |
| WR97 | 0.58907979 | 0.507124711 | 0.505130243 | 1.34967803 |
| WR98 | 0.498208657 | 0.534501482 | 0.532399408 | 1.38204538 |
| WR99 | 0.56912759 | 0.442650259 | 0.440909212 | 1.27345038 |
| WR100 | 0.917523174 | 0.477042303 | 0.475166076 | 1.31411183 |
| WR101 | 0.950550145 | 0.394688497 | 0.393135967 | 1.21674556 |
| WR102 | 1.000000155 | 0.388440613 | 0.386912639 | 1.20935873 |
| WR143 | 0.509043131 | 0.335676742 | 0.33435616 | 1.14697641 |
| WR144 | 0.449283934 | 0.744188325 | 0.741262066 | 1.62995652 |
| WR145 | 0.503941588 | 0.56198901 | 0.559778895 | 1.41454367 |
| WR146 | 0.394638455 | 0.511438303 | 0.50942688 | 1.35477796 |
| WR147 | 0.195448666 | 0.39559631 | 0.394040211 | 1.21781886 |
| WR148 | 0.343386467 | 0.754821003 | 0.751852951 | 1.64252745 |
| WR149 | 0.159995287 | 0.463563565 | 0.461740316 | 1.29817602 |
| WR150 | 0.370824874 | 0.649471263 | 0.646917294 | 1.51797326 |
| WR151 | 0.461843853 | 0.599122292 | 0.596766222 | 1.45844606 |
| WR152 | 0.427171448 | 0.668501699 | 0.66587293 | 1.5404728 |
| WR153 | 0.388105418 | 0.836789136 | 0.833498904 | 1.73943774 |
| WR154 | 0.480849842 | 0.884887366 | 0.881408081 | 1.79630391 |
| WR155 | 0.394294061 | 0.740687611 | 0.737775111 | 1.62581765 |
| WR156 | 0.457822453 | 0.672839025 | 0.670193207 | 1.54560079 |
| WR157 | 0.437703273 | 1.000001527 | 0.996069779 | 1.9324025 |
| WR158 | 0.527139435 | 0.645624433 | 0.643085585 | 1.51342519 |
| WR159 | 0.584117396 | 0.349473907 | 0.348099094 | 1.1632887 |
| WR160 | 0.585381911 | 0.880209858 | 0.876748959 | 1.79077373 |
| WR161 | 0.60601768 | 0.849057458 | 0.845719005 | 1.75394249 |
| WR162 | 0.454971641 | 0.758845835 | 0.755861963 | 1.64728598 |
| WR163 | 0.456895374 | 0.915851875 | 0.912250882 | 1.83291301 |
| WR164 | 0.519901957 | 0.708382151 | 0.705596629 | 1.58762315 |
| WR165 | 0.561890112 | 0.665004941 | 0.662389916 | 1.53633861 |
| WR166 | 0.350552631 | 0.844170303 | 0.840851059 | 1.74816444 |
| WR167 | 0.278395274 | 0.788469392 | 0.785369083 | 1.68230968 |
| WR168 | 0.333873907 | 0.529250411 | 0.527168977 | 1.37583707 |
| WR169 | 0.340685241 | 0.493020986 | 0.491081954 | 1.3330033 |
| WR170 | 0.321233976 | 0.683944397 | 0.68125493 | 1.55873058 |
| WR171 | 0.500504612 | 0.807167557 | 0.803993754 | 1.70441638 |
| WR172 | 0.244225543 | 0.473238985 | 0.471377707 | 1.3096152 |
| WR233 | 0.412854774 | 0.221219212 | 0.220348512 | 1.01165416 |
| WR234 | 0.40511636 | 0.511608394 | 0.509596303 | 1.35497905 |
| WR235 | 0.503802439 | 0.410536646 | 0.408921823 | 1.2354827 |
| WR236 | 0.489951914 | 0.27631887 | 0.275231598 | 1.07679806 |
| WR237 | 0.510952949 | 0.158594007 | 0.157969459 | 0.93761286 |
| WR238 | 0.451312028 | 0.349301838 | 0.347927702 | 1.16308526 |
| WR239 | 0.51328891 | 0.29949874 | 0.298320358 | 1.10420344 |
| WR240 | 0.500612453 | 0.337132406 | 0.335806102 | 1.14869744 |
| WR241 | 0.770272419 | 0.29521086 | 0.294049331 | 1.09913391 |
| WR242 | 0.758950924 | 0.32016482 | 0.318905209 | 1.12863679 |
| WR243 | 0.771449966 | 0.389174378 | 0.387643521 | 1.21022626 |
| WR244 | 0.922172484 | 0.2883459 | 0.287211355 | 1.09101753 |
| WR245 | 0.51122081 | 0.187669713 | 0.186930881 | 0.97198884 |
| WR246 | 0.632295933 | 0.372105925 | 0.370642156 | 1.19004636 |
| WR247 | 0.796648077 | 0.208719487 | 0.207897918 | 0.99687583 |
| WR248 | 0.76263315 | 0.21885178 | 0.217990385 | 1.00885516 |
| WR249 | 0.6551146 | 0.174904962 | 0.174216302 | 0.95689718 |
| WR250 | 0.616220767 | 0.343738273 | 0.342386005 | 1.1565075 |
| WR251 | 0.845908497 | 0.349456106 | 0.348081364 | 1.16326765 |
| WR252 | 0.69754977 | 0.41304648 | 0.411421792 | 1.23845006 |
| WR253 | 0.766339726 | 0.233596313 | 0.232676964 | 1.02628751 |
| WR254 | 0.468597789 | 0.511643994 | 0.509631763 | 1.35502114 |
| WR255 | 0.490765935 | 0.334375346 | 0.33305988 | 1.14543779 |
| WR298 | 0.228379972 | 0.418218439 | 0.416573422 | 1.24456483 |
| WR299 | 0.211054205 | 0.566083066 | 0.563856859 | 1.41938404 |
| WR300 | 0.276702877 | 0.161048463 | 0.160414267 | 0.94051474 |
| WR301 | 0.283508993 | 0.725858032 | 0.723003821 | 1.60828475 |
| WR302 | 0.399160791 | 0.246321508 | 0.245352142 | 1.04133241 |
| WR303 | 0.450875448 | 0.230190534 | 0.229284571 | 1.02226088 |
| WR304 | 0.370198705 | 0.724621904 | 0.721772552 | 1.60682329 |
| WR305 | 0.401347167 | 0.245631254 | 0.244664601 | 1.04051633 |
| WR306 | 0.423517052 | 0.238738605 | 0.237799044 | 1.0323672 |
| WR307 | 0.42015139 | 0.239861998 | 0.238918022 | 1.03369538 |
| WR393 | 0.550512957 | 0.58425909 | 0.581961441 | 1.44087341 |
| WR394 | 0.602180651 | 0.601796283 | 0.599429704 | 1.4616075 |
| WR395 | 0.41833028 | 0.475570816 | 0.473700373 | 1.3123721 |
| WR396 | 0.598698452 | 0.335815188 | 0.334494062 | 1.1471401 |
| WR397 | 0.628191043 | 0.747299412 | 0.744360924 | 1.63363473 |
| WR398 | 0.532089654 | 0.61388067 | 0.611466591 | 1.47589478 |
| WR399 | 0.561211761 | 0.621623775 | 0.619179262 | 1.48504939 |
| WR400 | 0.296668992 | 0.249280304 | 0.248299308 | 1.04483057 |
| WR401 | 0.353306039 | 0.231624442 | 0.230712844 | 1.02395618 |
| WR402 | 0.373626984 | 0.231929024 | 0.231016228 | 1.02431629 |
| WR403 | 0.368807217 | 0.668675746 | 0.666046293 | 1.54067857 |
| WR404 | 0.607275237 | 0.642679482 | 0.640152209 | 1.50994339 |
| WR405 | 0.374145313 | 0.645561144 | 0.643022544 | 1.51335036 |
| WR406 | 0.474650762 | 0.284647405 | 0.283527397 | 1.08664482 |
| WR407 | 0.419528699 | 0.301887928 | 0.300700155 | 1.10702816 |
| WR408 | 0.517155507 | 0.679181844 | 0.676511096 | 1.55309985 |
| WR409 | 0.557092957 | 0.348943855 | 0.347571126 | 1.16266202 |
| WR410 | 0.470752856 | 0.672916159 | 0.670270038 | 1.54569198 |
| WR411 | 0.656540875 | 0.847829242 | 0.844495616 | 1.75249037 |
| WR412 | 0.627274401 | 0.711172833 | 0.708376343 | 1.59092255 |
| WR413 | 0.500845527 | 0.606200854 | 0.603816962 | 1.46681499 |
| WR414 | 0.354311389 | 0.159743112 | 0.159114047 | 0.93897144 |
| WR415 | 0.386790461 | 0.43286606 | 0.43116347 | 1.2618826 |
| WR416 | 0.347646161 | 0.592761672 | 0.590430603 | 1.45092595 |
| WR417 | 0.54030987 | 0.599490163 | 0.597132648 | 1.45888099 |
| WR418 | 0.37504978 | 0.519996263 | 0.517951203 | 1.36489597 |
| WR419 | 0.554716991 | 0.568161739 | 0.565927361 | 1.42184164 |
| WR420 | 0.387397498 | 0.24868894 | 0.247710269 | 1.04413141 |
| WR421 | 0.784145556 | 0.482386331 | 0.480489099 | 1.32043003 |
| WR422 | 0.669490412 | 0.524841884 | 0.522777778 | 1.37062491 |
| WR14 | 0.033491621 | 0.237213718 | 0.23628015 | 1.03056434 |

Table S6 the 4968 transcripts identified by proteome and transcriptome analysis.

| **Protein accession** | **Regulation for proteome** | **Protein accession for transcriptome** | | **Identified ID for transcriptome** | | **Regulation**  **For transcriptome** | | **Type** | |
| --- | --- | --- | --- | --- | --- | --- | --- | --- | --- |
| OS01T0100900-01 |  | OS01g0100900 | | Os01g0100900 | |  | | - | |
| OS01T0101600-02 |  | OS01g0101600 | | Os01g0101600 | |  | | - | |
| OS01T0102300-01 |  | OS01g0102300 | | Os01g0102300 | |  | | - | |
| OS01T0102600-01 | Down | OS01g0102600 | | Os01g0102600 | |  | | Down- | |
| OS01T0102900-01 |  | OS01g0102900 | | Os01g0102900 | |  | | - | |
| OS01T0103600-02 |  | OS01g0103600 | | Os01g0103600 | |  | | - | |
| OS01T0104100-02 |  | OS01g0104100 | | Os01g0104100 | |  | | - | |
| OS01T0104400-03 |  | OS01g0104400 | | Os01g0104400 | |  | | - | |
| OS01T0104800-02 |  | OS01g0104800 | | Os01g0104800 | |  | | - | |
| OS01T0106400-01 |  | OS01g0106400 | | Os01g0106400 | | Up | | -Up | |
| OS01T0106500-01 |  | OS01g0106500 | | Os01g0106500 | |  | | - | |
| OS01T0106800-01 |  | OS01g0106800 | | Os01g0106800 | |  | | - | |
| OS01T0106900-02 |  | OS01g0106900 | | Os01g0106900 | |  | | - | |
| OS01T0107000-01 |  | OS01g0107000 | | Os01g0107000 | |  | | - | |
| OS01T0107900-02 |  | OS01g0107900 | | Os01g0107900 | |  | | - | |
| OS01T0108000-01 |  | OS01g0108000 | | Os01g0108000 | |  | | - | |
| OS01T0108200-01 |  | OS01g0108200 | | Os01g0108200 | |  | | - | |
| OS01T0109700-01 |  | OS01g0109700 | | Os01g0109700 | |  | | - | |
| OS01T0110400-01 |  | OS01g0110400 | | Os01g0110400 | |  | | - | |
| OS01T0111100-01 | Down | OS01g0111100 | | Os01g0111100 | | Down | | Down-Down | |
| OS01T0111200-01 |  | OS01g0111200 | | Os01g0111200 | |  | | - | |
| OS01T0111400-02 |  | OS01g0111400 | | Os01g0111400 | |  | | - | |
| OS01T0116600-02 |  | OS01g0116600 | | Os01g0116600 | |  | | - | |
| OS01T0118000-01 | Up | OS01g0118000 | | Os01g0118000 | | Up | | Up-Up | |
| OS01T0120300-00 |  | OS01g0120300 | | Os01g0120300 | |  | | - | |
| OS01T0120500-01 |  | OS01g0120500 | | Os01g0120500 | |  | | - | |
| OS01T0120800-03 |  | OS01g0120800 | | Os01g0120800 | |  | | - | |
| OS01T0121100-01 |  | OS01g0121100 | | Os01g0121100 | |  | | - | |
| OS01T0123900-01 |  | OS01g0123900 | | Os01g0123900 | | Down | | -Down | |
| OS01T0124000-01 |  | OS01g0124000 | | Os01g0124000 | | Down | | -Down | |
| OS01T0124200-02 |  | OS01g0124200 | | Os01g0124200 | |  | | - | |
| OS01T0125800-01 |  | OS01g0125800 | | Os01g0125800 | |  | | - | |
| OS01T0125900-01 |  | OS01g0125900 | | Os01g0125900 | |  | | - | |
| OS01T0126200-01 |  | OS01g0126200 | | Os01g0126200 | |  | | - | |
| OS01T0127300-01 |  | OS01g0127300 | | Os01g0127300 | |  | | - | |
| OS01T0128200-01 |  | OS01g0128200 | | Os01g0128200 | | Up | | -Up | |
| OS01T0130400-01 |  | OS01g0130400 | | Os01g0130400 | |  | | - | |
| OS01T0132000-01 |  | OS01g0132000 | | Os01g0132000 | | Up | | -Up | |
| OS01T0133400-01 |  | OS01g0133400 | | Os01g0133400 | |  | | - | |
| OS01T0135700-01 | Up | OS01g0135700 | | Os01g0135700 | |  | | Up- | |
| OS01T0138900-01 |  | OS01g0138900 | | Os01g0138900 | |  | | - | |
| OS01T0139200-02 |  | OS01g0139200 | | Os01g0139200 | |  | | - | |
| OS01T0140500-01 |  | OS01g0140500 | | Os01g0140500 | |  | | - | |
| OS01T0141100-01 |  | OS01g0141100 | | Os01g0141100 | |  | | - | |
| OS01T0141100-02 |  | OS01g0141100 | | Os01g0141100 | |  | | - | |
| OS01T0143300-01 |  | OS01g0143300 | | Os01g0143300 | |  | | - | |
| OS01T0143800-03 |  | OS01g0143800 | | Os01g0143800 | |  | | - | |
| OS01T0144100-01 |  | OS01g0144100 | | Os01g0144100 | | Down | | -Down | |
| OS01T0144340-00 |  | OS01g0144340 | | Os01g0144340 | |  | | - | |
| OS01T0144700-01 |  | OS01g0144700 | | Os01g0144700 | |  | | - | |
| OS01T0147700-01 |  | OS01g0147700 | | Os01g0147700 | |  | | - | |
| OS01T0147900-01 |  | OS01g0147900 | | Os01g0147900 | |  | | - | |
| OS01T0150000-02 |  | OS01g0150000 | | Os01g0150000 | |  | | - | |
| OS01T0151100-01 |  | OS01g0151100 | | Os01g0151100 | |  | | - | |
| OS01T0151200-01 |  | OS01g0151200 | | Os01g0151200 | |  | | - | |
| OS01T0153400-01 |  | OS01g0153400 | | Os01g0153400 | |  | | - | |
| OS01T0155400-01 |  | OS01g0155400 | | Os01g0155400 | |  | | - | |
| OS01T0155600-02 |  | OS01g0155600 | | Os01g0155600 | |  | | - | |
| OS01T0158100-01 |  | OS01g0158100 | | Os01g0158100 | |  | | - | |
| OS01T0158200-01 |  | OS01g0158200 | | Os01g0158200 | |  | | - | |
| OS01T0159400-01 |  | OS01g0159400 | | Os01g0159400 | |  | | - | |
| OS01T0160100-01 |  | OS01g0160100 | | Os01g0160100 | |  | | - | |
| OS01T0164600-01 | Down | OS01g0164600 | | Os01g0164600 | |  | | Down- | |
| OS01T0166700-01 |  | OS01g0166700 | | Os01g0166700 | |  | | - | |
| OS01T0167400-01 |  | OS01g0167400 | | Os01g0167400 | |  | | - | |
| OS01T0168100-01 |  | OS01g0168100 | | Os01g0168100 | |  | | - | |
| OS01T0168300-01 |  | OS01g0168300 | | Os01g0168300 | |  | | - | |
| OS01T0169900-01 |  | OS01g0169900 | | Os01g0169900 | |  | | - | |
| OS01T0171000-01 |  | OS01g0171000 | | Os01g0171000 | | Down | | -Down | |
| OS01T0172200-01 |  | OS01g0172200 | | Os01g0172200 | |  | | - | |
| OS01T0172400-03 |  | OS01g0172400 | | Os01g0172400 | |  | | - | |
| OS01T0173000-01 |  | OS01g0173000 | | Os01g0173000 | |  | | - | |
| OS01T0173900-01 |  | OS01g0173900 | | Os01g0173900 | |  | | - | |
| OS01T0174000-00 |  | OS01g0174000 | | Os01g0174000 | |  | | - | |
| OS01T0174300-02 |  | OS01g0174300 | | Os01g0174300 | |  | | - | |
| OS01T0174900-01 |  | OS01g0174900 | | Os01g0174900 | |  | | - | |
| OS01T0175000-01 |  | OS01g0175000 | | Os01g0175000 | |  | | - | |
| OS01T0177200-01 |  | OS01g0177200 | | Os01g0177200 | |  | | - | |
| OS01T0178000-01 | Up | OS01g0178000 | | Os01g0178000 | |  | | Up- | |
| OS01T0179200-01 |  | OS01g0179200 | | Os01g0179200 | |  | | - | |
| OS01T0179400-01 |  | OS01g0179400 | | Os01g0179400 | |  | | - | |
| OS01T0179600-01 |  | OS01g0179600 | | Os01g0179600 | | Up | | -Up | |
| OS01T0180000-01 |  | OS01g0180000 | | Os01g0180000 | |  | | - | |
| OS01T0180300-01 | Down | OS01g0180300 | | Os01g0180300 | | Down | | Down-Down | |
| OS01T0180800-01 |  | OS01g0180800 | | Os01g0180800 | |  | | - | |
| OS01T0182200-01 |  | OS01g0182200 | | Os01g0182200 | |  | | - | |
| OS01T0182600-03 |  | OS01g0182600 | | Os01g0182600 | |  | | - | |
| OS01T0184100-01 |  | OS01g0184100 | | Os01g0184100 | |  | | - | |
| OS01T0184500-01 |  | OS01g0184500 | | Os01g0184500 | |  | | - | |
| OS01T0184900-02 |  | OS01g0184900 | | Os01g0184900 | |  | | - | |
| OS01T0185200-03 |  | OS01g0185200 | | Os01g0185200 | |  | | - | |
| OS01T0185300-01 |  | OS01g0185300 | | Os01g0185300 | |  | | - | |
| OS01T0186700-01 |  | OS01g0186700 | | Os01g0186700 | |  | | - | |
| OS01T0187400-01 |  | OS01g0187400 | | Os01g0187400 | |  | | - | |
| OS01T0188100-01 |  | OS01g0188100 | | Os01g0188100 | | Up | | -Up | |
| OS01T0189800-01 |  | OS01g0189800 | | Os01g0189800 | |  | | - | |
| OS01T0190000-01 | Up | OS01g0190000 | | Os01g0190000 | |  | | Up- | |
| OS01T0191100-01 |  | OS01g0191100 | | Os01g0191100 | |  | | - | |
| OS01T0191200-02 |  | OS01g0191200 | | Os01g0191200 | | Down | | -Down | |
| OS01T0191500-01 |  | OS01g0191500 | | Os01g0191500 | |  | | - | |
| OS01T0191700-01 |  | OS01g0191700 | | Os01g0191700 | |  | | - | |
| OS01T0193600-01 |  | OS01g0193600 | | Os01g0193600 | |  | | - | |
| OS01T0195500-01 |  | OS01g0195500 | | Os01g0195500 | |  | | - | |
| OS01T0196500-01 |  | OS01g0196500 | | Os01g0196500 | |  | | - | |
| OS01T0196600-01 |  | OS01g0196600 | | Os01g0196600 | |  | | - | |
| OS01T0197200-01 |  | OS01g0197200 | | Os01g0197200 | |  | | - | |
| OS01T0198900-02 |  | OS01g0198900 | | Os01g0198900 | | Up | | -Up | |
| OS01T0199900-01 |  | OS01g0199900 | | Os01g0199900 | |  | | - | |
| OS01T0200500-01 |  | OS01g0200500 | | Os01g0200500 | |  | | - | |
| OS01T0200700-01 |  | OS01g0200700 | | Os01g0200700 | |  | | - | |
| OS01T0205100-01 |  | OS01g0205100 | | Os01g0205100 | |  | | - | |
| OS01T0207200-01 |  | OS01g0207200 | | Os01g0207200 | |  | | - | |
| OS01T0207400-01 |  | OS01g0207400 | | Os01g0207400 | | Down | | -Down | |
| OS01T0210500-01 | Up | OS01g0210500 | | Os01g0210500 | |  | | Up- | |
| OS01T0210600-02 |  | OS01g0210600 | | Os01g0210600 | |  | | - | |
| OS01T0212400-01 |  | OS01g0212400 | | Os01g0212400 | | Down | | -Down | |
| OS01T0214600-01 |  | OS01g0214600 | | Os01g0214600 | |  | | - | |
| OS01T0217800-01 |  | OS01g0217800 | | Os01g0217800 | |  | | - | |
| OS01T0218700-01 |  | OS01g0218700 | | Os01g0218700 | |  | | - | |
| OS01T0219000-00 |  | OS01g0219000 | | Os01g0219000 | |  | | - | |
| OS01T0221400-00 |  | OS01g0221400 | | Os01g0221400 | |  | | - | |
| OS01T0222600-01 |  | OS01g0222600 | | Os01g0222600 | | Down | | -Down | |
| OS01T0223200-01 |  | OS01g0223200 | | Os01g0223200 | |  | | - | |
| OS01T0224300-01 |  | OS01g0224300 | | Os01g0224300 | |  | | - | |
| OS01T0225400-01 |  | OS01g0225400 | | Os01g0225400 | | Down | | -Down | |
| OS01T0227200-01 |  | OS01g0227200 | | Os01g0227200 | |  | | - | |
| OS01T0227800-01 |  | OS01g0227800 | | Os01g0227800 | |  | | - | |
| OS01T0228600-03 |  | OS01g0228600 | | Os01g0228600 | |  | | - | |
| OS01T0229200-01 |  | OS01g0229200 | | Os01g0229200 | |  | | - | |
| OS01T0231900-01 |  | OS01g0231900 | | Os01g0231900 | |  | | - | |
| OS01T0232400-02 |  | OS01g0232400 | | Os01g0232400 | |  | | - | |
| OS01T0232700-01 |  | OS01g0232700 | | Os01g0232700 | |  | | - | |
| OS01T0233000-01 | Up | OS01g0233000 | | Os01g0233000 | |  | | Up- | |
| OS01T0235100-01 |  | OS01g0235100 | | Os01g0235100 | | Up | | -Up | |
| OS01T0235400-01 |  | OS01g0235400 | | Os01g0235400 | |  | | - | |
| OS01T0235400-02 |  | OS01g0235400 | | Os01g0235400 | |  | | - | |
| OS01T0235700-02 |  | OS01g0235700 | | Os01g0235700 | |  | | - | |
| OS01T0235800-01 |  | OS01g0235800 | | Os01g0235800 | |  | | - | |
| OS01T0235900-00 |  | OS01g0235900 | | Os01g0235900 | | Up | | -Up | |
| OS01T0237100-01 |  | OS01g0237100 | | Os01g0237100 | |  | | - | |
| OS01T0238500-02 |  | OS01g0238500 | | Os01g0238500 | | Down | | -Down | |
| OS01T0238600-01 |  | OS01g0238600 | | Os01g0238600 | |  | | - | |
| OS01T0242300-01 |  | OS01g0242300 | | Os01g0242300 | |  | | - | |
| OS01T0246700-01 |  | OS01g0246700 | | Os01g0246700 | |  | | - | |
| OS01T0248400-01 |  | OS01g0248400 | | Os01g0248400 | |  | | - | |
| OS01T0248500-01 |  | OS01g0248500 | | Os01g0248500 | |  | | - | |
| OS01T0249200-01 |  | OS01g0249200 | | Os01g0249200 | |  | | - | |
| OS01T0249300-02 |  | OS01g0249300 | | Os01g0249300 | |  | | - | |
| OS01T0250900-01 |  | OS01g0250900 | | Os01g0250900 | |  | | - | |
| OS01T0251000-01 |  | OS01g0251000 | | Os01g0251000 | |  | | - | |
| OS01T0251100-02 |  | OS01g0251100 | | Os01g0251100 | |  | | - | |
| OS01T0253300-02 |  | OS01g0253300 | | Os01g0253300 | |  | | - | |
| OS01T0254100-01 |  | OS01g0254100 | | Os01g0254100 | |  | | - | |
| OS01T0255000-01 |  | OS01g0255000 | | Os01g0255000 | |  | | - | |
| OS01T0256400-01 |  | OS01g0256400 | | Os01g0256400 | |  | | - | |
| OS01T0256600-01 |  | OS01g0256600 | | Os01g0256600 | |  | | - | |
| OS01T0256900-01 |  | OS01g0256900 | | Os01g0256900 | |  | | - | |
| OS01T0258700-03 |  | OS01g0258700 | | Os01g0258700 | |  | | - | |
| OS01T0259600-01 |  | OS01g0259600 | | Os01g0259600 | |  | | - | |
| OS01T0260700-01 |  | OS01g0260700 | | Os01g0260700 | |  | | - | |
| OS01T0263500-01 |  | OS01g0263500 | | Os01g0263500 | |  | | - | |
| OS01T0263600-01 |  | OS01g0263600 | | Os01g0263600 | |  | | - | |
| OS01T0265200-01 |  | OS01g0265200 | | Os01g0265200 | |  | | - | |
| OS01T0265800-02 |  | OS01g0265800 | | Os01g0265800 | |  | | - | |
| OS01T0265900-00 |  | OS01g0265900 | | Os01g0265900 | |  | | - | |
| OS01T0266000-01 |  | OS01g0266000 | | Os01g0266000 | |  | | - | |
| OS01T0266500-01 |  | OS01g0266500 | | Os01g0266500 | |  | | - | |
| OS01T0266600-02 |  | OS01g0266600 | | Os01g0266600 | |  | | - | |
| OS01T0267600-01 |  | OS01g0267600 | | Os01g0267600 | |  | | - | |
| OS01T0268300-01 |  | OS01g0268300 | | Os01g0268300 | |  | | - | |
| OS01T0269000-01 |  | OS01g0269000 | | Os01g0269000 | |  | | - | |
| OS01T0269100-01 |  | OS01g0269100 | | Os01g0269100 | |  | | - | |
| OS01T0270100-02 |  | OS01g0270100 | | Os01g0270100 | |  | | - | |
| OS01T0271400-00 |  | OS01g0271400 | | Os01g0271400 | |  | | - | |
| OS01T0271500-01 |  | OS01g0271500 | | Os01g0271500 | |  | | - | |
| OS01T0275600-01 |  | OS01g0275600 | | Os01g0275600 | |  | | - | |
| OS01T0276000-01 |  | OS01g0276000 | | Os01g0276000 | |  | | - | |
| OS01T0276100-01 | Up | OS01g0276100 | | Os01g0276100 | |  | | Up- | |
| OS01T0276200-00 |  | OS01g0276200 | | Os01g0276200 | |  | | - | |
| OS01T0276500-02 |  | OS01g0276500 | | Os01g0276500 | |  | | - | |
| OS01T0276700-04 |  | OS01g0276700 | | Os01g0276700 | |  | | - | |
| OS01T0276800-01 |  | OS01g0276800 | | Os01g0276800 | |  | | - | |
| OS01T0279100-01 |  | OS01g0279100 | | Os01g0279100 | |  | | - | |
| OS01T0279200-02 | Up | OS01g0279200 | | Os01g0279200 | |  | | Up- | |
| OS01T0279900-01 |  | OS01g0279900 | | Os01g0279900 | |  | | - | |
| OS01T0280400-00 |  | OS01g0280400 | | Os01g0280400 | | Down | | -Down | |
| OS01T0281400-00 |  | OS01g0281400 | | Os01g0281400 | |  | | - | |
| OS01T0283100-02 |  | OS01g0283100 | | Os01g0283100 | |  | | - | |
| OS01T0284500-01 |  | OS01g0284500 | | Os01g0284500 | | Up | | -Up | |
| OS01T0284700-01 | Down | OS01g0284700 | | Os01g0284700 | |  | | Down- | |
| OS01T0286600-01 |  | OS01g0286600 | | Os01g0286600 | |  | | - | |
| OS01T0289600-00 |  | OS01g0289600 | | Os01g0289600 | | Up | | -Up | |
| OS01T0290000-03 |  | OS01g0290000 | | Os01g0290000 | |  | | - | |
| OS01T0290100-01 |  | OS01g0290100 | | Os01g0290100 | |  | | - | |
| OS01T0293000-01 |  | OS01g0293000 | | Os01g0293000 | | Up | | -Up | |
| OS01T0294700-01 |  | OS01g0294700 | | Os01g0294700 | | Down | | -Down | |
| OS01T0300200-02 |  | OS01g0300200 | | Os01g0300200 | |  | | - | |
| OS01T0300600-01 |  | OS01g0300600 | | Os01g0300600 | |  | | - | |
| OS01T0303000-02 | Down | OS01g0303000 | | Os01g0303000 | |  | | Down- | |
| OS01T0303800-02 |  | OS01g0303800 | | Os01g0303800 | |  | | - | |
| OS01T0309800-01 |  | OS01g0309800 | | Os01g0309800 | |  | | - | |
| OS01T0314100-01 |  | OS01g0314100 | | Os01g0314100 | |  | | - | |
| OS01T0315800-01 |  | OS01g0315800 | | Os01g0315800 | |  | | - | |
| OS01T0317800-01 |  | OS01g0317800 | | Os01g0317800 | |  | | - | |
| OS01T0321300-00 |  | OS01g0321300 | | Os01g0321300 | |  | | - | |
| OS01T0321700-01 |  | OS01g0321700 | | Os01g0321700 | |  | | - | |
| OS01T0322800-01 |  | OS01g0322800 | | Os01g0322800 | |  | | - | |
| OS01T0323000-02 |  | OS01g0323000 | | Os01g0323000 | |  | | - | |
| OS01T0323300-00 |  | OS01g0323300 | | Os01g0323300 | |  | | - | |
| OS01T0323600-01 | Down | OS01g0323600 | | Os01g0323600 | |  | | Down- | |
| OS01T0326000-01 |  | OS01g0326000 | | Os01g0326000 | |  | | - | |
| OS01T0327100-01 |  | OS01g0327100 | | Os01g0327100 | | Up | | -Up | |
| OS01T0327400-01 | Up | OS01g0327400 | | Os01g0327400 | |  | | Up- | |
| OS01T0328400-01 |  | OS01g0328400 | | Os01g0328400 | |  | | - | |
| OS01T0328700-01 |  | OS01g0328700 | | Os01g0328700 | | Down | | -Down | |
| OS01T0332100-01 |  | OS01g0332100 | | Os01g0332100 | |  | | - | |
| OS01T0332800-02 |  | OS01g0332800 | | Os01g0332800 | |  | | - | |
| OS01T0337900-01 |  | OS01g0337900 | | Os01g0337900 | |  | | - | |
| OS01T0338200-01 |  | OS01g0338200 | | Os01g0338200 | |  | | - | |
| OS01T0338600-01 |  | OS01g0338600 | | Os01g0338600 | |  | | - | |
| OS01T0339900-01 |  | OS01g0339900 | | Os01g0339900 | |  | | - | |
| OS01T0348600-01 |  | OS01g0348600 | | Os01g0348600 | |  | | - | |
| OS01T0348900-01 |  | OS01g0348900 | | Os01g0348900 | |  | | - | |
| OS01T0351300-01 |  | OS01g0351300 | | Os01g0351300 | |  | | - | |
| OS01T0355600-01 |  | OS01g0355600 | | Os01g0355600 | |  | | - | |
| OS01T0356400-01 |  | OS01g0356400 | | Os01g0356400 | |  | | - | |
| OS01T0357100-01 | Up | OS01g0357100 | | Os01g0357100 | |  | | Up- | |
| OS01T0358300-01 |  | OS01g0358300 | | Os01g0358300 | |  | | - | |
| OS01T0358400-01 |  | OS01g0358400 | | Os01g0358400 | |  | | - | |
| OS01T0360200-03 |  | OS01g0360200 | | Os01g0360200 | |  | | - | |
| OS01T0360600-01 |  | OS01g0360600 | | Os01g0360600 | |  | | - | |
| OS01T0362100-01 |  | OS01g0362100 | | Os01g0362100 | | Up | | -Up | |
| OS01T0367100-04 | Down | OS01g0367100 | | Os01g0367100 | |  | | Down- | |
| OS01T0367400-01 |  | OS01g0367400 | | Os01g0367400 | |  | | - | |
| OS01T0367400-02 |  | OS01g0367400 | | Os01g0367400 | |  | | - | |
| OS01T0368000-00 |  | OS01g0368000 | | Os01g0368000 | |  | | - | |
| OS01T0369000-02 |  | OS01g0369000 | | Os01g0369000 | |  | | - | |
| OS01T0371200-01 | Up | OS01g0371200 | | Os01g0371200 | |  | | Up- | |
| OS01T0372700-01 |  | OS01g0372700 | | Os01g0372700 | |  | | - | |
| OS01T0375000-01 |  | OS01g0375000 | | Os01g0375000 | |  | | - | |
| OS01T0375200-02 | Up | OS01g0375200 | | Os01g0375200 | |  | | Up- | |
| OS01T0376700-01 | Up | OS01g0376700 | | Os01g0376700 | | Up | | Up-Up | |
| OS01T0377500-01 |  | OS01g0377500 | | Os01g0377500 | |  | | - | |
| OS01T0377700-02 |  | OS01g0377700 | | Os01g0377700 | |  | | - | |
| OS01T0378100-01 |  | OS01g0378100 | | Os01g0378100 | |  | | - | |
| OS01T0382000-01 |  | OS01g0382000 | | Os01g0382000 | | Down | | -Down | |
| OS01T0382400-01 |  | OS01g0382400 | | Os01g0382400 | | Down | | -Down | |
| OS01T0384300-00 |  | OS01g0384300 | | Os01g0384300 | |  | | - | |
| OS01T0500900-01 |  | OS01g0500900 | | Os01g0500900 | |  | | - | |
| OS01T0501700-01 |  | OS01g0501700 | | Os01g0501700 | |  | | - | |
| OS01T0501800-01 | Down | OS01g0501800 | | Os01g0501800 | |  | | Down- | |
| OS01T0505400-01 |  | OS01g0505400 | | Os01g0505400 | |  | | - | |
| OS01T0505400-02 |  | OS01g0505400 | | Os01g0505400 | |  | | - | |
| OS01T0505700-01 |  | OS01g0505700 | | Os01g0505700 | |  | | - | |
| OS01T0507300-01 |  | OS01g0507300 | | Os01g0507300 | |  | | - | |
| OS01T0507900-01 |  | OS01g0507900 | | Os01g0507900 | |  | | - | |
| OS01T0508000-02 |  | OS01g0508000 | | Os01g0508000 | |  | | - | |
| OS01T0510100-01 |  | OS01g0510100 | | Os01g0510100 | |  | | - | |
| OS01T0510600-01 |  | OS01g0510600 | | Os01g0510600 | |  | | - | |
| OS01T0510800-01 |  | OS01g0510800 | | Os01g0510800 | |  | | - | |
| OS01T0511100-01 |  | OS01g0511100 | | Os01g0511100 | | Up | | -Up | |
| OS01T0511200-01 |  | OS01g0511200 | | Os01g0511200 | |  | | - | |
| OS01T0511600-01 |  | OS01g0511600 | | Os01g0511600 | |  | | - | |
| OS01T0511600-02 |  | OS01g0511600 | | Os01g0511600 | |  | | - | |
| OS01T0512100-01 | Down | OS01g0512100 | | Os01g0512100 | |  | | Down- | |
| OS01T0518500-02 | Up | OS01g0518500 | | Os01g0518500 | |  | | Up- | |
| OS01T0523100-00 |  | OS01g0523100 | | Os01g0523100 | |  | | - | |
| OS01T0523401-00 |  | OS01g0523401 | | Os01g0523401 | |  | | - | |
| OS01T0524700-01 |  | OS01g0524700 | | Os01g0524700 | |  | | - | |
| OS01T0526100-01 |  | OS01g0526100 | | Os01g0526100 | |  | | - | |
| OS01T0527400-01 |  | OS01g0527400 | | Os01g0527400 | |  | | - | |
| OS01T0528800-01 | Up | OS01g0528800 | | Os01g0528800 | |  | | Up- | |
| OS01T0530400-02 |  | OS01g0530400 | | Os01g0530400 | |  | | - | |
| OS01T0531500-02 |  | OS01g0531500 | | Os01g0531500 | |  | | - | |
| OS01T0532200-01 |  | OS01g0532200 | | Os01g0532200 | |  | | - | |
| OS01T0542000-00 |  | OS01g0542000 | | Os01g0542000 | |  | | - | |
| OS01T0543100-01 |  | OS01g0543100 | | Os01g0543100 | |  | | - | |
| OS01T0546800-01 |  | OS01g0546800 | | Os01g0546800 | |  | | - | |
| OS01T0549400-01 |  | OS01g0549400 | | Os01g0549400 | |  | | - | |
| OS01T0550000-01 |  | OS01g0550000 | | Os01g0550000 | |  | | - | |
| OS01T0550100-02 |  | OS01g0550100 | | Os01g0550100 | |  | | - | |
| OS01T0552300-01 |  | OS01g0552300 | | Os01g0552300 | |  | | - | |
| OS01T0555200-01 |  | OS01g0555200 | | Os01g0555200 | |  | | - | |
| OS01T0555300-01 |  | OS01g0555300 | | Os01g0555300 | |  | | - | |
| OS01T0556400-01 |  | OS01g0556400 | | Os01g0556400 | |  | | - | |
| OS01T0558300-01 |  | OS01g0558300 | | Os01g0558300 | |  | | - | |
| OS01T0558600-01 |  | OS01g0558600 | | Os01g0558600 | |  | | - | |
| OS01T0559100-02 |  | OS01g0559100 | | Os01g0559100 | |  | | - | |
| OS01T0559600-01 |  | OS01g0559600 | | Os01g0559600 | | Up | | -Up | |
| OS01T0560000-01 |  | OS01g0560000 | | Os01g0560000 | |  | | - | |
| OS01T0560200-01 |  | OS01g0560200 | | Os01g0560200 | |  | | - | |
| OS01T0563000-04 |  | OS01g0563000 | | Os01g0563000 | | Down | | -Down | |
| OS01T0564300-01 |  | OS01g0564300 | | Os01g0564300 | |  | | - | |
| OS01T0565600-01 |  | OS01g0565600 | | Os01g0565600 | |  | | - | |
| OS01T0566900-01 |  | OS01g0566900 | | Os01g0566900 | |  | | - | |
| OS01T0570500-03 |  | OS01g0570500 | | Os01g0570500 | |  | | - | |
| OS01T0570700-01 |  | OS01g0570700 | | Os01g0570700 | |  | | - | |
| OS01T0571100-01 |  | OS01g0571100 | | Os01g0571100 | |  | | - | |
| OS01T0574500-01 |  | OS01g0574500 | | Os01g0574500 | |  | | - | |
| OS01T0574600-01 |  | OS01g0574600 | | Os01g0574600 | |  | | - | |
| OS01T0575000-03 |  | OS01g0575000 | | Os01g0575000 | |  | | - | |
| OS01T0575500-01 |  | OS01g0575500 | | Os01g0575500 | |  | | - | |
| OS01T0579900-01 |  | OS01g0579900 | | Os01g0579900 | |  | | - | |
| OS01T0580000-01 |  | OS01g0580000 | | Os01g0580000 | |  | | - | |
| OS01T0581300-01 |  | OS01g0581300 | | Os01g0581300 | | Down | | -Down | |
| OS01T0582400-01 |  | OS01g0582400 | | Os01g0582400 | |  | | - | |
| OS01T0582600-01 |  | OS01g0582600 | | Os01g0582600 | |  | | - | |
| OS01T0583100-01 |  | OS01g0583100 | | Os01g0583100 | | Up | | -Up | |
| OS01T0585700-01 |  | OS01g0585700 | | Os01g0585700 | |  | | - | |
| OS01T0586600-01 |  | OS01g0586600 | | Os01g0586600 | |  | | - | |
| OS01T0588000-01 |  | OS01g0588000 | | Os01g0588000 | |  | | - | |
| OS01T0588200-02 |  | OS01g0588200 | | Os01g0588200 | |  | | - | |
| OS01T0588800-01 |  | OS01g0588800 | | Os01g0588800 | |  | | - | |
| OS01T0588900-02 |  | OS01g0588900 | | Os01g0588900 | |  | | - | |
| OS01T0589000-01 |  | OS01g0589000 | | Os01g0589000 | |  | | - | |
| OS01T0589800-01 | Down | OS01g0589800 | | Os01g0589800 | |  | | Down- | |
| OS01T0591000-02 |  | OS01g0591000 | | Os01g0591000 | |  | | - | |
| OS01T0591300-01 |  | OS01g0591300 | | Os01g0591300 | |  | | - | |
| OS01T0593500-00 |  | OS01g0593500 | | Os01g0593500 | |  | | - | |
| OS01T0597800-01 |  | OS01g0597800 | | Os01g0597800 | | Up | | -Up | |
| OS01T0600000-01 | Up | OS01g0600000 | | Os01g0600000 | |  | | Up- | |
| OS01T0600300-01 |  | OS01g0600300 | | Os01g0600300 | |  | | - | |
| OS01T0600900-02 |  | OS01g0600900 | | Os01g0600900 | | Down | | -Down | |
| OS01T0602400-01 |  | OS01g0602400 | | Os01g0602400 | |  | | - | |
| OS01T0603000-01 |  | OS01g0603000 | | Os01g0603000 | |  | | - | |
| OS01T0605650-01 |  | OS01g0605650 | | Os01g0605650 | |  | | - | |
| OS01T0606133-00 |  | OS01g0606133 | | Os01g0606133 | |  | | - | |
| OS01T0607400-01 |  | OS01g0607400 | | Os01g0607400 | |  | | - | |
| OS01T0607900-01 |  | OS01g0607900 | | Os01g0607900 | |  | | - | |
| OS01T0610400-01 |  | OS01g0610400 | | Os01g0610400 | |  | | - | |
| OS01T0610500-01 |  | OS01g0610500 | | Os01g0610500 | |  | | - | |
| OS01T0610600-01 |  | OS01g0610600 | | Os01g0610600 | |  | | - | |
| OS01T0611000-02 | Down | OS01g0611000 | | Os01g0611000 | | Down | | Down-Down | |
| OS01T0611100-01 |  | OS01g0611100 | | Os01g0611100 | |  | | - | |
| OS01T0612200-01 | Up | OS01g0612200 | | Os01g0612200 | |  | | Up- | |
| OS01T0613300-01 |  | OS01g0613300 | | Os01g0613300 | |  | | - | |
| OS01T0614700-01 |  | OS01g0614700 | | Os01g0614700 | |  | | - | |
| OS01T0614900-01 |  | OS01g0614900 | | Os01g0614900 | |  | | - | |
| OS01T0615050-00 |  | OS01g0615050 | | Os01g0615050 | |  | | - | |
| OS01T0615200-01 |  | OS01g0615200 | | Os01g0615200 | |  | | - | |
| OS01T0615300-02 |  | OS01g0615300 | | Os01g0615300 | |  | | - | |
| OS01T0616100-01 |  | OS01g0616100 | | Os01g0616100 | |  | | - | |
| OS01T0616400-01 |  | OS01g0616400 | | Os01g0616400 | |  | | - | |
| OS01T0616500-01 |  | OS01g0616500 | | Os01g0616500 | |  | | - | |
| OS01T0616600-01 |  | OS01g0616600 | | Os01g0616600 | |  | | - | |
| OS01T0616900-00 |  | OS01g0616900 | | Os01g0616900 | |  | | - | |
| OS01T0617500-01 |  | OS01g0617500 | | Os01g0617500 | |  | | - | |
| OS01T0617900-02 | Down | OS01g0617900 | | Os01g0617900 | |  | | Down- | |
| OS01T0618100-01 | Up | OS01g0618100 | | Os01g0618100 | |  | | Up- | |
| OS01T0618400-01 |  | OS01g0618400 | | Os01g0618400 | |  | | - | |
| OS01T0618500-01 |  | OS01g0618500 | | Os01g0618500 | |  | | - | |
| OS01T0618800-02 |  | OS01g0618800 | | Os01g0618800 | |  | | - | |
| OS01T0618900-02 |  | OS01g0618900 | | Os01g0618900 | |  | | - | |
| OS01T0619000-02 |  | OS01g0619000 | | Os01g0619000 | |  | | - | |
| OS01T0620100-01 |  | OS01g0620100 | | Os01g0620100 | |  | | - | |
| OS01T0622300-01 |  | OS01g0622300 | | Os01g0622300 | |  | | - | |
| OS01T0622600-01 | Down | OS01g0622600 | | Os01g0622600 | |  | | Down- | |
| OS01T0624000-02 |  | OS01g0624000 | | Os01g0624000 | |  | | - | |
| OS01T0624500-01 |  | OS01g0624500 | | Os01g0624500 | |  | | - | |
| OS01T0626300-01 |  | OS01g0626300 | | Os01g0626300 | |  | | - | |
| OS01T0627500-01 | Up | OS01g0627500 | | Os01g0627500 | |  | | Up- | |
| OS01T0633200-01 |  | OS01g0633200 | | Os01g0633200 | |  | | - | |
| OS01T0635550-00 |  | OS01g0635550 | | Os01g0635550 | |  | | - | |
| OS01T0637600-01 |  | OS01g0637600 | | Os01g0637600 | |  | | - | |
| OS01T0638600-00 |  | OS01g0638600 | | Os01g0638600 | |  | | - | |
| OS01T0639900-01 |  | OS01g0639900 | | Os01g0639900 | | Down | | -Down | |
| OS01T0641000-01 |  | OS01g0641000 | | Os01g0641000 | |  | | - | |
| OS01T0642200-01 |  | OS01g0642200 | | Os01g0642200 | |  | | - | |
| OS01T0642900-01 |  | OS01g0642900 | | Os01g0642900 | |  | | - | |
| OS01T0644000-02 |  | OS01g0644000 | | Os01g0644000 | | Up | | -Up | |
| OS01T0647100-01 |  | OS01g0647100 | | Os01g0647100 | |  | | - | |
| OS01T0649100-02 |  | OS01g0649100 | | Os01g0649100 | |  | | - | |
| OS01T0652600-01 |  | OS01g0652600 | | Os01g0652600 | |  | | - | |
| OS01T0653800-02 |  | OS01g0653800 | | Os01g0653800 | |  | | - | |
| OS01T0654500-01 |  | OS01g0654500 | | Os01g0654500 | |  | | - | |
| OS01T0655800-01 |  | OS01g0655800 | | Os01g0655800 | |  | | - | |
| OS01T0658400-01 |  | OS01g0658400 | | Os01g0658400 | |  | | - | |
| OS01T0659200-01 | Up | OS01g0659200 | | Os01g0659200 | |  | | Up- | |
| OS01T0660200-01 | Up | OS01g0660200 | | Os01g0660200 | |  | | Up- | |
| OS01T0660900-00 |  | OS01g0660900 | | Os01g0660900 | |  | | - | |
| OS01T0662300-01 |  | OS01g0662300 | | Os01g0662300 | | Down | | -Down | |
| OS01T0662600-02 |  | OS01g0662600 | | Os01g0662600 | |  | | - | |
| OS01T0663500-01 | Up | OS01g0663500 | | Os01g0663500 | |  | | Up- | |
| OS01T0663800-01 |  | OS01g0663800 | | Os01g0663800 | |  | | - | |
| OS01T0664000-00 |  | OS01g0664000 | | Os01g0664000 | |  | | - | |
| OS01T0664500-03 |  | OS01g0664500 | | Os01g0664500 | |  | | - | |
| OS01T0665400-01 | Up | OS01g0665400 | | Os01g0665400 | |  | | Up- | |
| OS01T0666600-00 |  | OS01g0666600 | | Os01g0666600 | | Up | | -Up | |
| OS01T0666800-01 |  | OS01g0666800 | | Os01g0666800 | |  | | - | |
| OS01T0667200-02 | Up | OS01g0667200 | | Os01g0667200 | | Up | | Up-Up | |
| OS01T0668100-01 |  | OS01g0668100 | | Os01g0668100 | |  | | - | |
| OS01T0672400-04 |  | OS01g0672400 | | Os01g0672400 | |  | | - | |
| OS01T0672800-01 |  | OS01g0672800 | | Os01g0672800 | |  | | - | |
| OS01T0673600-01 |  | OS01g0673600 | | Os01g0673600 | |  | | - | |
| OS01T0673800-01 |  | OS01g0673800 | | Os01g0673800 | |  | | - | |
| OS01T0675100-01 |  | OS01g0675100 | | Os01g0675100 | |  | | - | |
| OS01T0676200-02 | Down | OS01g0676200 | | Os01g0676200 | |  | | Down- | |
| OS01T0678600-01 |  | OS01g0678600 | | Os01g0678600 | | Down | | -Down | |
| OS01T0679000-01 |  | OS01g0679000 | | Os01g0679000 | |  | | - | |
| OS01T0679600-02 |  | OS01g0679600 | | Os01g0679600 | |  | | - | |
| OS01T0681200-02 |  | OS01g0681200 | | Os01g0681200 | |  | | - | |
| OS01T0682001-01 |  | OS01g0682001 | | Os01g0682001 | |  | | - | |
| OS01T0682500-01 |  | OS01g0682500 | | Os01g0682500 | |  | | - | |
| OS01T0685800-02 |  | OS01g0685800 | | Os01g0685800 | |  | | - | |
| OS01T0686800-02 |  | OS01g0686800 | | Os01g0686800 | |  | | - | |
| OS01T0687400-01 |  | OS01g0687400 | | Os01g0687400 | | Down | | -Down | |
| OS01T0687500-01 |  | OS01g0687500 | | Os01g0687500 | |  | | - | |
| OS01T0687600-01 |  | OS01g0687600 | | Os01g0687600 | |  | | - | |
| OS01T0691500-01 |  | OS01g0691500 | | Os01g0691500 | |  | | - | |
| OS01T0691700-01 |  | OS01g0691700 | | Os01g0691700 | |  | | - | |
| OS01T0693800-01 |  | OS01g0693800 | | Os01g0693800 | |  | | - | |
| OS01T0693900-02 |  | OS01g0693900 | | Os01g0693900 | |  | | - | |
| OS01T0695100-01 |  | OS01g0695100 | | Os01g0695100 | |  | | - | |
| OS01T0695300-01 |  | OS01g0695300 | | Os01g0695300 | |  | | - | |
| OS01T0695800-01 |  | OS01g0695800 | | Os01g0695800 | |  | | - | |
| OS01T0696800-01 |  | OS01g0696800 | | Os01g0696800 | | Down | | -Down | |
| OS01T0698000-02 |  | OS01g0698000 | | Os01g0698000 | |  | | - | |
| OS01T0698300-01 |  | OS01g0698300 | | Os01g0698300 | |  | | - | |
| OS01T0700000-02 |  | OS01g0700000 | | Os01g0700000 | | Up | | -Up | |
| OS01T0703400-01 | Up | OS01g0703400 | | Os01g0703400 | |  | | Up- | |
| OS01T0703600-01 |  | OS01g0703600 | | Os01g0703600 | |  | | - | |
| OS01T0704200-01 |  | OS01g0704200 | | Os01g0704200 | |  | | - | |
| OS01T0705200-01 |  | OS01g0705200 | | Os01g0705200 | |  | | - | |
| OS01T0706400-03 |  | OS01g0706400 | | Os01g0706400 | |  | | - | |
| OS01T0706500-00 |  | OS01g0706500 | | Os01g0706500 | |  | | - | |
| OS01T0708000-00 |  | OS01g0708000 | | Os01g0708000 | |  | | - | |
| OS01T0708100-01 |  | OS01g0708100 | | Os01g0708100 | |  | | - | |
| OS01T0709400-01 |  | OS01g0709400 | | Os01g0709400 | |  | | - | |
| OS01T0710000-01 |  | OS01g0710000 | | Os01g0710000 | |  | | - | |
| OS01T0710700-01 |  | OS01g0710700 | | Os01g0710700 | |  | | - | |
| OS01T0711100-01 |  | OS01g0711100 | | Os01g0711100 | |  | | - | |
| OS01T0711400-01 |  | OS01g0711400 | | Os01g0711400 | | Up | | -Up | |
| OS01T0712600-00 |  | OS01g0712600 | | Os01g0712600 | |  | | - | |
| OS01T0713200-01 |  | OS01g0713200 | | Os01g0713200 | | Down | | -Down | |
| OS01T0713800-00 |  | OS01g0713800 | | Os01g0713800 | |  | | - | |
| OS01T0714100-01 |  | OS01g0714100 | | Os01g0714100 | |  | | - | |
| OS01T0714900-01 |  | OS01g0714900 | | Os01g0714900 | |  | | - | |
| OS01T0715400-02 |  | OS01g0715400 | | Os01g0715400 | |  | | - | |
| OS01T0716400-01 |  | OS01g0716400 | | Os01g0716400 | |  | | - | |
| OS01T0717700-01 | Up | OS01g0717700 | | Os01g0717700 | |  | | Up- | |
| OS01T0718900-01 |  | OS01g0718900 | | Os01g0718900 | |  | | - | |
| OS01T0720300-00 |  | OS01g0720300 | | Os01g0720300 | |  | | - | |
| OS01T0720500-01 | Down | OS01g0720500 | | Os01g0720500 | |  | | Down- | |
| OS01T0721900-01 |  | OS01g0721900 | | Os01g0721900 | |  | | - | |
| OS01T0723200-01 |  | OS01g0723200 | | Os01g0723200 | |  | | - | |
| OS01T0723400-02 |  | OS01g0723400 | | Os01g0723400 | |  | | - | |
| OS01T0723600-01 |  | OS01g0723600 | | Os01g0723600 | |  | | - | |
| OS01T0725600-01 |  | OS01g0725600 | | Os01g0725600 | |  | | - | |
| OS01T0727400-01 |  | OS01g0727400 | | Os01g0727400 | |  | | - | |
| OS01T0730300-03 |  | OS01g0730300 | | Os01g0730300 | |  | | - | |
| OS01T0730500-01 |  | OS01g0730500 | | Os01g0730500 | |  | | - | |
| OS01T0732000-01 |  | OS01g0732000 | | Os01g0732000 | |  | | - | |
| OS01T0734600-01 |  | OS01g0734600 | | Os01g0734600 | |  | | - | |
| OS01T0734800-02 |  | OS01g0734800 | | Os01g0734800 | |  | | - | |
| OS01T0736900-02 |  | OS01g0736900 | | Os01g0736900 | |  | | - | |
| OS01T0737300-00 |  | OS01g0737300 | | Os01g0737300 | |  | | - | |
| OS01T0738600-01 |  | OS01g0738600 | | Os01g0738600 | |  | | - | |
| OS01T0739000-01 |  | OS01g0739000 | | Os01g0739000 | |  | | - | |
| OS01T0739400-01 |  | OS01g0739400 | | Os01g0739400 | |  | | - | |
| OS01T0740500-00 |  | OS01g0740500 | | Os01g0740500 | |  | | - | |
| OS01T0740600-01 | Up | OS01g0740600 | | Os01g0740600 | |  | | Up- | |
| OS01T0742300-01 |  | OS01g0742300 | | Os01g0742300 | |  | | - | |
| OS01T0742500-01 |  | OS01g0742500 | | Os01g0742500 | |  | | - | |
| OS01T0743300-01 |  | OS01g0743300 | | Os01g0743300 | |  | | - | |
| OS01T0743400-01 |  | OS01g0743400 | | Os01g0743400 | | Down | | -Down | |
| OS01T0744300-01 |  | OS01g0744300 | | Os01g0744300 | |  | | - | |
| OS01T0747500-01 |  | OS01g0747500 | | Os01g0747500 | |  | | - | |
| OS01T0747700-01 |  | OS01g0747700 | | Os01g0747700 | |  | | - | |
| OS01T0748600-02 |  | OS01g0748600 | | Os01g0748600 | |  | | - | |
| OS01T0748900-02 |  | OS01g0748900 | | Os01g0748900 | |  | | - | |
| OS01T0749200-01 | Down | OS01g0749200 | | Os01g0749200 | | Down | | Down-Down | |
| OS01T0749400-02 |  | OS01g0749400 | | Os01g0749400 | |  | | - | |
| OS01T0750800-02 |  | OS01g0750800 | | Os01g0750800 | |  | | - | |
| OS01T0752200-01 |  | OS01g0752200 | | Os01g0752200 | |  | | - | |
| OS01T0752300-01 |  | OS01g0752300 | | Os01g0752300 | |  | | - | |
| OS01T0752700-01 | Up | OS01g0752700 | | Os01g0752700 | |  | | Up- | |
| OS01T0752800-01 |  | OS01g0752800 | | Os01g0752800 | |  | | - | |
| OS01T0753100-01 | Up | OS01g0753100 | | Os01g0753100 | |  | | Up- | |
| OS01T0754100-01 |  | OS01g0754100 | | Os01g0754100 | |  | | - | |
| OS01T0754500-01 | Up | OS01g0754500 | | Os01g0754500 | |  | | Up- | |
| OS01T0757500-02 |  | OS01g0757500 | | Os01g0757500 | |  | | - | |
| OS01T0758300-02 |  | OS01g0758300 | | Os01g0758300 | |  | | - | |
| OS01T0760600-04 |  | OS01g0760600 | | Os01g0760600 | |  | | - | |
| OS01T0761300-01 |  | OS01g0761300 | | Os01g0761300 | |  | | - | |
| OS01T0763300-01 |  | OS01g0763300 | | Os01g0763300 | |  | | - | |
| OS01T0763600-02 |  | OS01g0763600 | | Os01g0763600 | | Up | | -Up | |
| OS01T0763700-01 |  | OS01g0763700 | | Os01g0763700 | | Down | | -Down | |
| OS01T0764000-03 |  | OS01g0764000 | | Os01g0764000 | |  | | - | |
| OS01T0764400-01 |  | OS01g0764400 | | Os01g0764400 | |  | | - | |
| OS01T0764600-02 |  | OS01g0764600 | | Os01g0764600 | |  | | - | |
| OS01T0764900-01 |  | OS01g0764900 | | Os01g0764900 | |  | | - | |
| OS01T0767100-01 |  | OS01g0767100 | | Os01g0767100 | |  | | - | |
| OS01T0767700-01 |  | OS01g0767700 | | Os01g0767700 | |  | | - | |
| OS01T0769900-01 |  | OS01g0769900 | | Os01g0769900 | |  | | - | |
| OS01T0770500-02 |  | OS01g0770500 | | Os01g0770500 | |  | | - | |
| OS01T0770700-00 |  | OS01g0770700 | | Os01g0770700 | |  | | - | |
| OS01T0771400-00 |  | OS01g0771400 | | Os01g0771400 | |  | | - | |
| OS01T0771900-01 |  | OS01g0771900 | | Os01g0771900 | |  | | - | |
| OS01T0772000-01 |  | OS01g0772000 | | Os01g0772000 | |  | | - | |
| OS01T0772200-02 |  | OS01g0772200 | | Os01g0772200 | |  | | - | |
| OS01T0772400-00 |  | OS01g0772400 | | Os01g0772400 | |  | | - | |
| OS01T0772800-01 |  | OS01g0772800 | | Os01g0772800 | |  | | - | |
| OS01T0773200-01 |  | OS01g0773200 | | Os01g0773200 | |  | | - | |
| OS01T0776600-02 |  | OS01g0776600 | | Os01g0776600 | | Up | | -Up | |
| OS01T0778700-01 |  | OS01g0778700 | | Os01g0778700 | |  | | - | |
| OS01T0778800-01 |  | OS01g0778800 | | Os01g0778800 | |  | | - | |
| OS01T0779300-01 |  | OS01g0779300 | | Os01g0779300 | |  | | - | |
| OS01T0780500-01 |  | OS01g0780500 | | Os01g0780500 | |  | | - | |
| OS01T0783500-01 |  | OS01g0783500 | | Os01g0783500 | |  | | - | |
| OS01T0783600-01 |  | OS01g0783600 | | Os01g0783600 | |  | | - | |
| OS01T0784800-01 | Up | OS01g0784800 | | Os01g0784800 | |  | | Up- | |
| OS01T0788500-01 |  | OS01g0788500 | | Os01g0788500 | |  | | - | |
| OS01T0788700-00 |  | OS01g0788700 | | Os01g0788700 | |  | | - | |
| OS01T0793200-01 |  | OS01g0793200 | | Os01g0793200 | |  | | - | |
| OS01T0793500-01 |  | OS01g0793500 | | Os01g0793500 | | Up | | -Up | |
| OS01T0794400-01 |  | OS01g0794400 | | Os01g0794400 | | Up | | -Up | |
| OS01T0794800-01 |  | OS01g0794800 | | Os01g0794800 | |  | | - | |
| OS01T0795000-01 | Up | OS01g0795000 | | Os01g0795000 | | Down | | Up-Down | |
| OS01T0795200-01 | Up | OS01g0795200 | | Os01g0795200 | | Down | | Up-Down | |
| OS01T0796400-01 |  | OS01g0796400 | | Os01g0796400 | |  | | - | |
| OS01T0799900-01 |  | OS01g0799900 | | Os01g0799900 | |  | | - | |
| OS01T0800500-01 | Up | OS01g0800500 | | Os01g0800500 | | Up | | Up-Up | |
| OS01T0801000-01 |  | OS01g0801000 | | Os01g0801000 | |  | | - | |
| OS01T0801100-01 |  | OS01g0801100 | | Os01g0801100 | |  | | - | |
| OS01T0801700-01 |  | OS01g0801700 | | Os01g0801700 | |  | | - | |
| OS01T0802100-01 |  | OS01g0802100 | | Os01g0802100 | |  | | - | |
| OS01T0803200-02 | Up | OS01g0803200 | | Os01g0803200 | |  | | Up- | |
| OS01T0805200-02 |  | OS01g0805200 | | Os01g0805200 | |  | | - | |
| OS01T0805300-02 | Down | OS01g0805300 | | Os01g0805300 | | Down | | Down-Down | |
| OS01T0805800-02 |  | OS01g0805800 | | Os01g0805800 | |  | | - | |
| OS01T0805900-01 |  | OS01g0805900 | | Os01g0805900 | |  | | - | |
| OS01T0807900-02 | Down | OS01g0807900 | | Os01g0807900 | |  | | Down- | |
| OS01T0809900-01 |  | OS01g0809900 | | Os01g0809900 | |  | | - | |
| OS01T0811100-02 |  | OS01g0811100 | | Os01g0811100 | |  | | - | |
| OS01T0811300-01 |  | OS01g0811300 | | Os01g0811300 | |  | | - | |
| OS01T0812900-01 |  | OS01g0812900 | | Os01g0812900 | |  | | - | |
| OS01T0813900-01 |  | OS01g0813900 | | Os01g0813900 | |  | | - | |
| OS01T0814900-01 |  | OS01g0814900 | | Os01g0814900 | |  | | - | |
| OS01T0815700-01 |  | OS01g0815700 | | Os01g0815700 | |  | | - | |
| OS01T0817700-02 |  | OS01g0817700 | | Os01g0817700 | |  | | - | |
| OS01T0819200-00 |  | OS01g0819200 | | Os01g0819200 | |  | | - | |
| OS01T0819900-01 |  | OS01g0819900 | | Os01g0819900 | |  | | - | |
| OS01T0821700-01 |  | OS01g0821700 | | Os01g0821700 | |  | | - | |
| OS01T0821800-03 | Down | OS01g0821800 | | Os01g0821800 | |  | | Down- | |
| OS01T0822900-03 |  | OS01g0822900 | | Os01g0822900 | |  | | - | |
| OS01T0826900-01 |  | OS01g0826900 | | Os01g0826900 | | Up | | -Up | |
| OS01T0828100-00 |  | OS01g0828100 | | Os01g0828100 | |  | | - | |
| OS01T0830000-01 |  | OS01g0830000 | | Os01g0830000 | |  | | - | |
| OS01T0830100-01 | Up | OS01g0830100 | | Os01g0830100 | |  | | Up- | |
| OS01T0833150-00 |  | OS01g0833150 | | Os01g0833150 | |  | | - | |
| OS01T0833800-01 |  | OS01g0833800 | | Os01g0833800 | |  | | - | |
| OS01T0834700-01 |  | OS01g0834700 | | Os01g0834700 | |  | | - | |
| OS01T0835600-05 |  | OS01g0835600 | | Os01g0835600 | |  | | - | |
| OS01T0836800-04 |  | OS01g0836800 | | Os01g0836800 | |  | | - | |
| OS01T0837500-01 |  | OS01g0837500 | | Os01g0837500 | |  | | - | |
| OS01T0839300-01 |  | OS01g0839300 | | Os01g0839300 | |  | | - | |
| OS01T0840100-01 | Up | OS01g0840100 | | Os01g0840100 | |  | | Up- | |
| OS01T0843300-00 |  | OS01g0843300 | | Os01g0843300 | |  | | - | |
| OS01T0844800-03 |  | OS01g0844800 | | Os01g0844800 | |  | | - | |
| OS01T0846800-01 |  | OS01g0846800 | | Os01g0846800 | |  | | - | |
| OS01T0847700-01 |  | OS01g0847700 | | Os01g0847700 | | Up | | -Up | |
| OS01T0847800-01 |  | OS01g0847800 | | Os01g0847800 | |  | | - | |
| OS01T0849000-02 |  | OS01g0849000 | | Os01g0849000 | | Up | | -Up | |
| OS01T0850900-01 |  | OS01g0850900 | | Os01g0850900 | | Up | | -Up | |
| OS01T0851000-02 |  | OS01g0851000 | | Os01g0851000 | |  | | - | |
| OS01T0851400-01 |  | OS01g0851400 | | Os01g0851400 | |  | | - | |
| OS01T0851700-01 |  | OS01g0851700 | | Os01g0851700 | |  | | - | |
| OS01T0853800-04 |  | OS01g0853800 | | Os01g0853800 | |  | | - | |
| OS01T0856600-01 |  | OS01g0856600 | | Os01g0856600 | |  | | - | |
| OS01T0857700-01 |  | OS01g0857700 | | Os01g0857700 | |  | | - | |
| OS01T0858500-01 |  | OS01g0858500 | | Os01g0858500 | |  | | - | |
| OS01T0860400-01 |  | OS01g0860400 | | Os01g0860400 | |  | | - | |
| OS01T0860500-02 |  | OS01g0860500 | | Os01g0860500 | | Down | | -Down | |
| OS01T0862200-01 |  | OS01g0862200 | | Os01g0862200 | |  | | - | |
| OS01T0862300-02 |  | OS01g0862300 | | Os01g0862300 | |  | | - | |
| OS01T0865100-01 |  | OS01g0865100 | | Os01g0865100 | | Down | | -Down | |
| OS01T0865400-01 |  | OS01g0865400 | | Os01g0865400 | |  | | - | |
| OS01T0866100-01 |  | OS01g0866100 | | Os01g0866100 | |  | | - | |
| OS01T0866300-02 |  | OS01g0866300 | | Os01g0866300 | |  | | - | |
| OS01T0866400-01 |  | OS01g0866400 | | Os01g0866400 | |  | | - | |
| OS01T0866600-01 |  | OS01g0866600 | | Os01g0866600 | |  | | - | |
| OS01T0867800-03 |  | OS01g0867800 | | Os01g0867800 | |  | | - | |
| OS01T0868900-01 |  | OS01g0868900 | | Os01g0868900 | |  | | - | |
| OS01T0869800-01 |  | OS01g0869800 | | Os01g0869800 | |  | | - | |
| OS01T0871300-01 |  | OS01g0871300 | | Os01g0871300 | |  | | - | |
| OS01T0872700-02 |  | OS01g0872700 | | Os01g0872700 | |  | | - | |
| OS01T0873100-01 |  | OS01g0873100 | | Os01g0873100 | |  | | - | |
| OS01T0874800-04 |  | OS01g0874800 | | Os01g0874800 | |  | | - | |
| OS01T0874900-01 |  | OS01g0874900 | | Os01g0874900 | |  | | - | |
| OS01T0875400-01 |  | OS01g0875400 | | Os01g0875400 | |  | | - | |
| OS01T0877300-01 |  | OS01g0877300 | | Os01g0877300 | |  | | - | |
| OS01T0878200-01 |  | OS01g0878200 | | Os01g0878200 | |  | | - | |
| OS01T0881500-02 |  | OS01g0881500 | | Os01g0881500 | |  | | - | |
| OS01T0882500-00 |  | OS01g0882500 | | Os01g0882500 | |  | | - | |
| OS01T0885000-01 |  | OS01g0885000 | | Os01g0885000 | |  | | - | |
| OS01T0886300-03 |  | OS01g0886300 | | Os01g0886300 | |  | | - | |
| OS01T0887100-01 |  | OS01g0887100 | | Os01g0887100 | |  | | - | |
| OS01T0887400-01 |  | OS01g0887400 | | Os01g0887400 | |  | | - | |
| OS01T0888500-01 |  | OS01g0888500 | | Os01g0888500 | | Down | | -Down | |
| OS01T0889000-01 |  | OS01g0889000 | | Os01g0889000 | |  | | - | |
| OS01T0889800-01 |  | OS01g0889800 | | Os01g0889800 | |  | | - | |
| OS01T0891300-02 |  | OS01g0891300 | | Os01g0891300 | |  | | - | |
| OS01T0892600-02 |  | OS01g0892600 | | Os01g0892600 | | Up | | -Up | |
| OS01T0894300-01 |  | OS01g0894300 | | Os01g0894300 | |  | | - | |
| OS01T0894500-01 |  | OS01g0894500 | | Os01g0894500 | |  | | - | |
| OS01T0894700-01 | Down | OS01g0894700 | | Os01g0894700 | | Down | | Down-Down | |
| OS01T0895100-02 |  | OS01g0895100 | | Os01g0895100 | |  | | - | |
| OS01T0895600-01 |  | OS01g0895600 | | Os01g0895600 | |  | | - | |
| OS01T0896500-01 |  | OS01g0896500 | | Os01g0896500 | |  | | - | |
| OS01T0896700-00 |  | OS01g0896700 | | Os01g0896700 | |  | | - | |
| OS01T0897200-04 | Up | OS01g0897200 | | Os01g0897200 | | Up | | Up-Up | |
| OS01T0897800-04 |  | OS01g0897800 | | Os01g0897800 | |  | | - | |
| OS01T0901500-01 |  | OS01g0901500 | | Os01g0901500 | |  | | - | |
| OS01T0902200-01 |  | OS01g0902200 | | Os01g0902200 | |  | | - | |
| OS01T0904200-01 |  | OS01g0904200 | | Os01g0904200 | |  | | - | |
| OS01T0905800-03 | Up | OS01g0905800 | | Os01g0905800 | |  | | Up- | |
| OS01T0907300-02 |  | OS01g0907300 | | Os01g0907300 | |  | | - | |
| OS01T0908400-02 |  | OS01g0908400 | | Os01g0908400 | |  | | - | |
| OS01T0910900-01 |  | OS01g0910900 | | Os01g0910900 | |  | | - | |
| OS01T0911100-01 |  | OS01g0911100 | | Os01g0911100 | |  | | - | |
| OS01T0911100-02 |  | OS01g0911100 | | Os01g0911100 | |  | | - | |
| OS01T0911200-01 | Up | OS01g0911200 | | Os01g0911200 | |  | | Up- | |
| OS01T0911300-01 |  | OS01g0911300 | | Os01g0911300 | |  | | - | |
| OS01T0911900-01 |  | OS01g0911900 | | Os01g0911900 | |  | | - | |
| OS01T0912700-01 |  | OS01g0912700 | | Os01g0912700 | |  | | - | |
| OS01T0913000-01 |  | OS01g0913000 | | Os01g0913000 | | Up | | -Up | |
| OS01T0914800-01 |  | OS01g0914800 | | Os01g0914800 | |  | | - | |
| OS01T0916200-01 |  | OS01g0916200 | | Os01g0916200 | |  | | - | |
| OS01T0916200-02 |  | OS01g0916200 | | Os01g0916200 | |  | | - | |
| OS01T0916300-02 |  | OS01g0916300 | | Os01g0916300 | |  | | - | |
| OS01T0916400-01 |  | OS01g0916400 | | Os01g0916400 | |  | | - | |
| OS01T0916600-01 | Down | OS01g0916600 | | Os01g0916600 | |  | | Down- | |
| OS01T0916800-00 |  | OS01g0916800 | | Os01g0916800 | |  | | - | |
| OS01T0917400-02 | Down | OS01g0917400 | | Os01g0917400 | |  | | Down- | |
| OS01T0918300-01 |  | OS01g0918300 | | Os01g0918300 | |  | | - | |
| OS01T0919200-01 |  | OS01g0919200 | | Os01g0919200 | |  | | - | |
| OS01T0919400-00 |  | OS01g0919400 | | Os01g0919400 | | Up | | -Up | |
| OS01T0919700-01 |  | OS01g0919700 | | Os01g0919700 | |  | | - | |
| OS01T0919900-02 |  | OS01g0919900 | | Os01g0919900 | |  | | - | |
| OS01T0920100-01 |  | OS01g0920100 | | Os01g0920100 | |  | | - | |
| OS01T0920400-01 |  | OS01g0920400 | | Os01g0920400 | |  | | - | |
| OS01T0921200-01 |  | OS01g0921200 | | Os01g0921200 | |  | | - | |
| OS01T0921600-01 |  | OS01g0921600 | | Os01g0921600 | |  | | - | |
| OS01T0923300-01 |  | OS01g0923300 | | Os01g0923300 | |  | | - | |
| OS01T0924000-01 | Down | OS01g0924000 | | Os01g0924000 | |  | | Down- | |
| OS01T0924300-01 |  | OS01g0924300 | | Os01g0924300 | |  | | - | |
| OS01T0924600-01 |  | OS01g0924600 | | Os01g0924600 | |  | | - | |
| OS01T0924800-00 |  | OS01g0924800 | | Os01g0924800 | |  | | - | |
| OS01T0924900-01 | Up | OS01g0924900 | | Os01g0924900 | |  | | Up- | |
| OS01T0925200-01 |  | OS01g0925200 | | Os01g0925200 | |  | | - | |
| OS01T0925300-01 |  | OS01g0925300 | | Os01g0925300 | |  | | - | |
| OS01T0925800-01 |  | OS01g0925800 | | Os01g0925800 | |  | | - | |
| OS01T0926300-01 | Up | OS01g0926300 | | Os01g0926300 | |  | | Up- | |
| OS01T0927900-01 |  | OS01g0927900 | | Os01g0927900 | |  | | - | |
| OS01T0929100-03 |  | OS01g0929100 | | Os01g0929100 | | Down | | -Down | |
| OS01T0930800-01 |  | OS01g0930800 | | Os01g0930800 | |  | | - | |
| OS01T0930900-00 |  | OS01g0930900 | | Os01g0930900 | |  | | - | |
| OS01T0931100-02 |  | OS01g0931100 | | Os01g0931100 | |  | | - | |
| OS01T0931400-02 |  | OS01g0931400 | | Os01g0931400 | |  | | - | |
| OS01T0932950-00 |  | OS01g0932950 | | Os01g0932950 | |  | | - | |
| OS01T0933900-02 |  | OS01g0933900 | | Os01g0933900 | |  | | - | |
| OS01T0934000-02 |  | OS01g0934000 | | Os01g0934000 | |  | | - | |
| OS01T0934100-01 |  | OS01g0934100 | | Os01g0934100 | |  | | - | |
| OS01T0934200-01 |  | OS01g0934200 | | Os01g0934200 | |  | | - | |
| OS01T0935400-00 |  | OS01g0935400 | | Os01g0935400 | |  | | - | |
| OS01T0935600-02 |  | OS01g0935600 | | Os01g0935600 | |  | | - | |
| OS01T0935700-02 | Up | OS01g0935700 | | Os01g0935700 | |  | | Up- | |
| OS01T0937100-01 |  | OS01g0937100 | | Os01g0937100 | |  | | - | |
| OS01T0938100-01 | Down | OS01g0938100 | | Os01g0938100 | |  | | Down- | |
| OS01T0938200-01 |  | OS01g0938200 | | Os01g0938200 | |  | | - | |
| OS01T0938900-02 |  | OS01g0938900 | | Os01g0938900 | |  | | - | |
| OS01T0939400-01 |  | OS01g0939400 | | Os01g0939400 | |  | | - | |
| OS01T0939700-01 |  | OS01g0939700 | | Os01g0939700 | |  | | - | |
| OS01T0940600-01 |  | OS01g0940600 | | Os01g0940600 | |  | | - | |
| OS01T0940700-01 |  | OS01g0940700 | | Os01g0940700 | | Down | | -Down | |
| OS01T0941800-01 |  | OS01g0941800 | | Os01g0941800 | |  | | - | |
| OS01T0944700-01 |  | OS01g0944700 | | Os01g0944700 | | Down | | -Down | |
| OS01T0945800-01 |  | OS01g0945800 | | Os01g0945800 | |  | | - | |
| OS01T0946500-01 |  | OS01g0946500 | | Os01g0946500 | |  | | - | |
| OS01T0948400-02 |  | OS01g0948400 | | Os01g0948400 | |  | | - | |
| OS01T0949060-01 |  | OS01g0949060 | | Os01g0949060 | |  | | - | |
| OS01T0949400-01 |  | OS01g0949400 | | Os01g0949400 | |  | | - | |
| OS01T0950800-01 |  | OS01g0950800 | | Os01g0950800 | |  | | - | |
| OS01T0950866-00 |  | OS01g0950866 | | Os01g0950866 | |  | | - | |
| OS01T0951000-01 |  | OS01g0951000 | | Os01g0951000 | |  | | - | |
| OS01T0951000-02 |  | OS01g0951000 | | Os01g0951000 | |  | | - | |
| OS01T0951200-01 |  | OS01g0951200 | | Os01g0951200 | |  | | - | |
| OS01T0951500-01 |  | OS01g0951500 | | Os01g0951500 | |  | | - | |
| OS01T0952100-00 |  | OS01g0952100 | | Os01g0952100 | |  | | - | |
| OS01T0952600-01 |  | OS01g0952600 | | Os01g0952600 | |  | | - | |
| OS01T0953400-01 |  | OS01g0953400 | | Os01g0953400 | |  | | - | |
| OS01T0953600-02 | Up | OS01g0953600 | | Os01g0953600 | |  | | Up- | |
| OS01T0954000-01 | Up | OS01g0954000 | | Os01g0954000 | | Up | | Up-Up | |
| OS01T0958100-01 | Down | OS01g0958100 | | Os01g0958100 | |  | | Down- | |
| OS01T0959000-01 |  | OS01g0959000 | | Os01g0959000 | |  | | - | |
| OS01T0959800-01 |  | OS01g0959800 | | Os01g0959800 | |  | | - | |
| OS01T0959900-02 | Down | OS01g0959900 | | Os01g0959900 | |  | | Down- | |
| OS01T0960300-02 |  | OS01g0960300 | | Os01g0960300 | |  | | - | |
| OS01T0960400-01 | Up | OS01g0960400 | | Os01g0960400 | |  | | Up- | |
| OS01T0960500-01 |  | OS01g0960500 | | Os01g0960500 | |  | | - | |
| OS01T0961200-01 |  | OS01g0961200 | | Os01g0961200 | | Up | | -Up | |
| OS01T0962600-01 |  | OS01g0962600 | | Os01g0962600 | |  | | - | |
| OS01T0962700-01 | Up | OS01g0962700 | | Os01g0962700 | |  | | Up- | |
| OS01T0963000-04 |  | OS01g0963000 | | Os01g0963000 | |  | | - | |
| OS01T0963400-01 |  | OS01g0963400 | | Os01g0963400 | |  | | - | |
| OS01T0963600-01 |  | OS01g0963600 | | Os01g0963600 | |  | | - | |
| OS01T0965900-01 |  | OS01g0965900 | | Os01g0965900 | | Up | | -Up | |
| OS01T0966000-02 |  | OS01g0966000 | | Os01g0966000 | |  | | - | |
| OS01T0966200-01 |  | OS01g0966200 | | Os01g0966200 | | Down | | -Down | |
| OS01T0966300-01 |  | OS01g0966300 | | Os01g0966300 | |  | | - | |
| OS01T0966700-01 |  | OS01g0966700 | | Os01g0966700 | |  | | - | |
| OS01T0967900-02 |  | OS01g0967900 | | Os01g0967900 | |  | | - | |
| OS01T0969000-01 |  | OS01g0969000 | | Os01g0969000 | |  | | - | |
| OS01T0969100-01 |  | OS01g0969100 | | Os01g0969100 | | Down | | -Down | |
| OS01T0970400-01 |  | OS01g0970400 | | Os01g0970400 | |  | | - | |
| OS01T0970700-02 | Up | OS01g0970700 | | Os01g0970700 | | Up | | Up-Up | |
| OS01T0971500-01 |  | OS01g0971500 | | Os01g0971500 | |  | | - | |
| OS01T0973100-01 |  | OS01g0973100 | | Os01g0973100 | | Down | | -Down | |
| OS01T0973400-01 |  | OS01g0973400 | | Os01g0973400 | |  | | - | |
| OS01T0974000-02 |  | OS01g0974000 | | Os01g0974000 | |  | | - | |
| OS01T0975900-02 |  | OS01g0975900 | | Os01g0975900 | | Up | | -Up | |
| OS01T0977200-00 |  | OS01g0977200 | | Os01g0977200 | |  | | - | |
| OS01T0978100-03 |  | OS01g0978100 | | Os01g0978100 | |  | | - | |
| OS01T0978400-01 |  | OS01g0978400 | | Os01g0978400 | |  | | - | |
| OS02T0100100-01 |  | OS02g0100100 | | Os02g0100100 | |  | | - | |
| OS02T0100300-01 |  | OS02g0100300 | | Os02g0100300 | |  | | - | |
| OS02T0100700-01 |  | OS02g0100700 | | Os02g0100700 | |  | | - | |
| OS02T0101100-01 |  | OS02g0101100 | | Os02g0101100 | |  | | - | |
| OS02T0101500-02 |  | OS02g0101500 | | Os02g0101500 | |  | | - | |
| OS02T0101700-02 |  | OS02g0101700 | | Os02g0101700 | |  | | - | |
| OS02T0102400-02 |  | OS02g0102400 | | Os02g0102400 | |  | | - | |
| OS02T0102900-01 |  | OS02g0102900 | | Os02g0102900 | |  | | - | |
| OS02T0103800-02 | Down | OS02g0103800 | | Os02g0103800 | |  | | Down- | |
| OS02T0104700-01 |  | OS02g0104700 | | Os02g0104700 | |  | | - | |
| OS02T0104800-01 |  | OS02g0104800 | | Os02g0104800 | |  | | - | |
| OS02T0105200-01 |  | OS02g0105200 | | Os02g0105200 | |  | | - | |
| OS02T0105400-02 |  | OS02g0105400 | | Os02g0105400 | |  | | - | |
| OS02T0105500-02 |  | OS02g0105500 | | Os02g0105500 | |  | | - | |
| OS02T0105900-01 |  | OS02g0105900 | | Os02g0105900 | |  | | - | |
| OS02T0106100-01 |  | OS02g0106100 | | Os02g0106100 | |  | | - | |
| OS02T0106800-01 |  | OS02g0106800 | | Os02g0106800 | |  | | - | |
| OS02T0106966-00 |  | OS02g0106966 | | Os02g0106966 | |  | | - | |
| OS02T0107600-01 |  | OS02g0107600 | | Os02g0107600 | |  | | - | |
| OS02T0108400-00 |  | OS02g0108400 | | Os02g0108400 | |  | | - | |
| OS02T0109100-01 |  | OS02g0109100 | | Os02g0109100 | |  | | - | |
| OS02T0109800-01 |  | OS02g0109800 | | Os02g0109800 | |  | | - | |
| OS02T0110200-02 |  | OS02g0110200 | | Os02g0110200 | | Down | | -Down | |
| OS02T0110400-01 |  | OS02g0110400 | | Os02g0110400 | |  | | - | |
| OS02T0114000-01 |  | OS02g0114000 | | Os02g0114000 | |  | | - | |
| OS02T0115600-01 | Down | OS02g0115600 | | Os02g0115600 | |  | | Down- | |
| OS02T0115700-01 | Up | OS02g0115700 | | Os02g0115700 | |  | | Up- | |
| OS02T0115900-02 |  | OS02g0115900 | | Os02g0115900 | |  | | - | |
| OS02T0116900-01 |  | OS02g0116900 | | Os02g0116900 | |  | | - | |
| OS02T0117100-01 |  | OS02g0117100 | | Os02g0117100 | |  | | - | |
| OS02T0117200-01 |  | OS02g0117200 | | Os02g0117200 | |  | | - | |
| OS02T0117600-02 |  | OS02g0117600 | | Os02g0117600 | |  | | - | |
| OS02T0118200-01 |  | OS02g0118200 | | Os02g0118200 | |  | | - | |
| OS02T0119200-01 |  | OS02g0119200 | | Os02g0119200 | |  | | - | |
| OS02T0120400-01 |  | OS02g0120400 | | Os02g0120400 | |  | | - | |
| OS02T0120900-01 |  | OS02g0120900 | | Os02g0120900 | |  | | - | |
| OS02T0121000-01 |  | OS02g0121000 | | Os02g0121000 | |  | | - | |
| OS02T0121100-01 |  | OS02g0121100 | | Os02g0121100 | |  | | - | |
| OS02T0121300-01 |  | OS02g0121300 | | Os02g0121300 | |  | | - | |
| OS02T0122000-01 |  | OS02g0122000 | | Os02g0122000 | |  | | - | |
| OS02T0122500-00 |  | OS02g0122500 | | Os02g0122500 | |  | | - | |
| OS02T0122800-02 |  | OS02g0122800 | | Os02g0122800 | |  | | - | |
| OS02T0123500-02 |  | OS02g0123500 | | Os02g0123500 | |  | | - | |
| OS02T0125000-01 |  | OS02g0125000 | | Os02g0125000 | |  | | - | |
| OS02T0125100-01 |  | OS02g0125100 | | Os02g0125100 | |  | | - | |
| OS02T0125700-01 |  | OS02g0125700 | | Os02g0125700 | |  | | - | |
| OS02T0127100-00 |  | OS02g0127100 | | Os02g0127100 | |  | | - | |
| OS02T0127700-02 |  | OS02g0127700 | | Os02g0127700 | |  | | - | |
| OS02T0129900-01 | Down | OS02g0129900 | | Os02g0129900 | |  | | Down- | |
| OS02T0130000-01 |  | OS02g0130000 | | Os02g0130000 | |  | | - | |
| OS02T0130100-01 |  | OS02g0130100 | | Os02g0130100 | |  | | - | |
| OS02T0131100-01 |  | OS02g0131100 | | Os02g0131100 | |  | | - | |
| OS02T0131100-02 |  | OS02g0131100 | | Os02g0131100 | |  | | - | |
| OS02T0131200-01 | Up | OS02g0131200 | | Os02g0131200 | |  | | Up- | |
| OS02T0131300-01 | Up | OS02g0131300 | | Os02g0131300 | |  | | Up- | |
| OS02T0131700-00 |  | OS02g0131700 | | Os02g0131700 | |  | | - | |
| OS02T0133300-01 |  | OS02g0133300 | | Os02g0133300 | |  | | - | |
| OS02T0133800-01 |  | OS02g0133800 | | Os02g0133800 | |  | | - | |
| OS02T0135800-02 |  | OS02g0135800 | | Os02g0135800 | |  | | - | |
| OS02T0135850-00 |  | OS02g0135850 | | Os02g0135850 | |  | | - | |
| OS02T0135900-02 |  | OS02g0135900 | | Os02g0135900 | |  | | - | |
| OS02T0137100-01 |  | OS02g0137100 | | Os02g0137100 | |  | | - | |
| OS02T0137200-01 | Down | OS02g0137200 | | Os02g0137200 | |  | | Down- | |
| OS02T0137400-01 |  | OS02g0137400 | | Os02g0137400 | |  | | - | |
| OS02T0137800-01 |  | OS02g0137800 | | Os02g0137800 | |  | | - | |
| OS02T0138600-01 |  | OS02g0138600 | | Os02g0138600 | |  | | - | |
| OS02T0140300-01 |  | OS02g0140300 | | Os02g0140300 | |  | | - | |
| OS02T0141000-01 |  | OS02g0141000 | | Os02g0141000 | |  | | - | |
| OS02T0141300-01 |  | OS02g0141300 | | Os02g0141300 | |  | | - | |
| OS02T0143100-01 |  | OS02g0143100 | | Os02g0143100 | |  | | - | |
| OS02T0146400-02 |  | OS02g0146400 | | Os02g0146400 | |  | | - | |
| OS02T0146600-01 |  | OS02g0146600 | | Os02g0146600 | |  | | - | |
| OS02T0146700-02 |  | OS02g0146700 | | Os02g0146700 | |  | | - | |
| OS02T0146900-01 |  | OS02g0146900 | | Os02g0146900 | |  | | - | |
| OS02T0147700-03 |  | OS02g0147700 | | Os02g0147700 | |  | | - | |
| OS02T0148500-01 |  | OS02g0148500 | | Os02g0148500 | |  | | - | |
| OS02T0149800-01 |  | OS02g0149800 | | Os02g0149800 | |  | | - | |
| OS02T0150100-02 |  | OS02g0150100 | | Os02g0150100 | |  | | - | |
| OS02T0152200-02 |  | OS02g0152200 | | Os02g0152200 | |  | | - | |
| OS02T0152600-01 |  | OS02g0152600 | | Os02g0152600 | |  | | - | |
| OS02T0152900-01 |  | OS02g0152900 | | Os02g0152900 | | Down | | -Down | |
| OS02T0158200-00 | Down | OS02g0158200 | | Os02g0158200 | |  | | Down- | |
| OS02T0161200-01 |  | OS02g0161200 | | Os02g0161200 | |  | | - | |
| OS02T0162000-02 |  | OS02g0162000 | | Os02g0162000 | |  | | - | |
| OS02T0162200-00 |  | OS02g0162200 | | Os02g0162200 | |  | | - | |
| OS02T0162500-01 | Down | OS02g0162500 | | Os02g0162500 | |  | | Down- | |
| OS02T0167000-00 |  | OS02g0167000 | | Os02g0167000 | |  | | - | |
| OS02T0167100-01 | Down | OS02g0167100 | | Os02g0167100 | |  | | Down- | |
| OS02T0167500-01 |  | OS02g0167500 | | Os02g0167500 | |  | | - | |
| OS02T0167700-01 |  | OS02g0167700 | | Os02g0167700 | |  | | - | |
| OS02T0168100-01 |  | OS02g0168100 | | Os02g0168100 | | Up | | -Up | |
| OS02T0168700-01 |  | OS02g0168700 | | Os02g0168700 | |  | | - | |
| OS02T0168800-01 | Down | OS02g0168800 | | Os02g0168800 | | Down | | Down-Down | |
| OS02T0169300-01 | Up | OS02g0169300 | | Os02g0169300 | |  | | Up- | |
| OS02T0169900-03 |  | OS02g0169900 | | Os02g0169900 | |  | | - | |
| OS02T0170100-02 |  | OS02g0170100 | | Os02g0170100 | |  | | - | |
| OS02T0170200-01 |  | OS02g0170200 | | Os02g0170200 | |  | | - | |
| OS02T0171100-02 |  | OS02g0171100 | | Os02g0171100 | |  | | - | |
| OS02T0173100-03 |  | OS02g0173100 | | Os02g0173100 | | Down | | -Down | |
| OS02T0173500-01 |  | OS02g0173500 | | Os02g0173500 | |  | | - | |
| OS02T0173900-01 |  | OS02g0173900 | | Os02g0173900 | |  | | - | |
| OS02T0175800-02 | Up | OS02g0175800 | | Os02g0175800 | |  | | Up- | |
| OS02T0175900-02 |  | OS02g0175900 | | Os02g0175900 | |  | | - | |
| OS02T0177600-01 |  | OS02g0177600 | | Os02g0177600 | |  | | - | |
| OS02T0177800-01 |  | OS02g0177800 | | Os02g0177800 | |  | | - | |
| OS02T0177900-02 |  | OS02g0177900 | | Os02g0177900 | |  | | - | |
| OS02T0178500-01 |  | OS02g0178500 | | Os02g0178500 | |  | | - | |
| OS02T0179100-01 |  | OS02g0179100 | | Os02g0179100 | |  | | - | |
| OS02T0179300-01 |  | OS02g0179300 | | Os02g0179300 | |  | | - | |
| OS02T0180000-01 |  | OS02g0180000 | | Os02g0180000 | |  | | - | |
| OS02T0180100-01 |  | OS02g0180100 | | Os02g0180100 | |  | | - | |
| OS02T0180200-01 |  | OS02g0180200 | | Os02g0180200 | | Down | | -Down | |
| OS02T0180500-01 |  | OS02g0180500 | | Os02g0180500 | |  | | - | |
| OS02T0181800-01 |  | OS02g0181800 | | Os02g0181800 | |  | | - | |
| OS02T0181900-01 |  | OS02g0181900 | | Os02g0181900 | |  | | - | |
| OS02T0182100-01 |  | OS02g0182100 | | Os02g0182100 | |  | | - | |
| OS02T0182800-01 |  | OS02g0182800 | | Os02g0182800 | |  | | - | |
| OS02T0184200-01 |  | OS02g0184200 | | Os02g0184200 | |  | | - | |
| OS02T0184400-03 |  | OS02g0184400 | | Os02g0184400 | |  | | - | |
| OS02T0185300-01 |  | OS02g0185300 | | Os02g0185300 | | Up | | -Up | |
| OS02T0187100-00 |  | OS02g0187100 | | Os02g0187100 | |  | | - | |
| OS02T0187500-01 |  | OS02g0187500 | | Os02g0187500 | |  | | - | |
| OS02T0187800-01 |  | OS02g0187800 | | Os02g0187800 | |  | | - | |
| OS02T0188000-01 |  | OS02g0188000 | | Os02g0188000 | | Up | | -Up | |
| OS02T0190600-00 |  | OS02g0190600 | | Os02g0190600 | |  | | - | |
| OS02T0192100-01 |  | OS02g0192100 | | Os02g0192100 | |  | | - | |
| OS02T0192700-02 |  | OS02g0192700 | | Os02g0192700 | | Down | | -Down | |
| OS02T0194000-01 |  | OS02g0194000 | | Os02g0194000 | |  | | - | |
| OS02T0194200-01 |  | OS02g0194200 | | Os02g0194200 | |  | | - | |
| OS02T0194700-01 |  | OS02g0194700 | | Os02g0194700 | | Up | | -Up | |
| OS02T0194800-02 |  | OS02g0194800 | | Os02g0194800 | |  | | - | |
| OS02T0195500-02 |  | OS02g0195500 | | Os02g0195500 | |  | | - | |
| OS02T0195800-01 |  | OS02g0195800 | | Os02g0195800 | |  | | - | |
| OS02T0196300-01 |  | OS02g0196300 | | Os02g0196300 | |  | | - | |
| OS02T0196800-01 |  | OS02g0196800 | | Os02g0196800 | |  | | - | |
| OS02T0197500-01 |  | OS02g0197500 | | Os02g0197500 | |  | | - | |
| OS02T0197600-03 |  | OS02g0197600 | | Os02g0197600 | |  | | - | |
| OS02T0198300-01 |  | OS02g0198300 | | Os02g0198300 | |  | | - | |
| OS02T0198600-01 |  | OS02g0198600 | | Os02g0198600 | |  | | - | |
| OS02T0198700-01 |  | OS02g0198700 | | Os02g0198700 | | Down | | -Down | |
| OS02T0199900-01 |  | OS02g0199900 | | Os02g0199900 | |  | | - | |
| OS02T0200800-01 |  | OS02g0200800 | | Os02g0200800 | |  | | - | |
| OS02T0202250-01 |  | OS02g0202250 | | Os02g0202250 | |  | | - | |
| OS02T0202500-01 |  | OS02g0202500 | | Os02g0202500 | |  | | - | |
| OS02T0203700-02 |  | OS02g0203700 | | Os02g0203700 | |  | | - | |
| OS02T0205400-01 |  | OS02g0205400 | | Os02g0205400 | |  | | - | |
| OS02T0206100-02 |  | OS02g0206100 | | Os02g0206100 | | Up | | -Up | |
| OS02T0206700-01 |  | OS02g0206700 | | Os02g0206700 | | Up | | -Up | |
| OS02T0208100-01 |  | OS02g0208100 | | Os02g0208100 | |  | | - | |
| OS02T0208600-01 |  | OS02g0208600 | | Os02g0208600 | |  | | - | |
| OS02T0209000-01 |  | OS02g0209000 | | Os02g0209000 | |  | | - | |
| OS02T0215000-01 |  | OS02g0215000 | | Os02g0215000 | |  | | - | |
| OS02T0217500-01 |  | OS02g0217500 | | Os02g0217500 | |  | | - | |
| OS02T0217600-01 |  | OS02g0217600 | | Os02g0217600 | |  | | - | |
| OS02T0218200-01 | Up | OS02g0218200 | | Os02g0218200 | |  | | Up- | |
| OS02T0220500-01 |  | OS02g0220500 | | Os02g0220500 | |  | | - | |
| OS02T0220600-02 |  | OS02g0220600 | | Os02g0220600 | |  | | - | |
| OS02T0221500-01 |  | OS02g0221500 | | Os02g0221500 | |  | | - | |
| OS02T0223700-01 |  | OS02g0223700 | | Os02g0223700 | |  | | - | |
| OS02T0224200-01 |  | OS02g0224200 | | Os02g0224200 | |  | | - | |
| OS02T0228300-01 | Up | OS02g0228300 | | Os02g0228300 | |  | | Up- | |
| OS02T0229400-02 |  | OS02g0229400 | | Os02g0229400 | |  | | - | |
| OS02T0232400-01 |  | OS02g0232400 | | Os02g0232400 | |  | | - | |
| OS02T0234500-01 |  | OS02g0234500 | | Os02g0234500 | |  | | - | |
| OS02T0234800-01 |  | OS02g0234800 | | Os02g0234800 | |  | | - | |
| OS02T0235600-01 |  | OS02g0235600 | | Os02g0235600 | |  | | - | |
| OS02T0236000-01 |  | OS02g0236000 | | Os02g0236000 | |  | | - | |
| OS02T0238500-01 |  | OS02g0238500 | | Os02g0238500 | |  | | - | |
| OS02T0240100-01 |  | OS02g0240100 | | Os02g0240100 | |  | | - | |
| OS02T0240300-01 | Down | OS02g0240300 | | Os02g0240300 | | Down | | Down-Down | |
| OS02T0244700-02 | Up | OS02g0244700 | | Os02g0244700 | |  | | Up- | |
| OS02T0245000-02 |  | OS02g0245000 | | Os02g0245000 | |  | | - | |
| OS02T0245100-01 |  | OS02g0245100 | | Os02g0245100 | |  | | - | |
| OS02T0246300-00 |  | OS02g0246300 | | Os02g0246300 | |  | | - | |
| OS02T0247200-01 |  | OS02g0247200 | | Os02g0247200 | |  | | - | |
| OS02T0250300-01 |  | OS02g0250300 | | Os02g0250300 | |  | | - | |
| OS02T0250400-02 |  | OS02g0250400 | | Os02g0250400 | |  | | - | |
| OS02T0251700-01 |  | OS02g0251700 | | Os02g0251700 | |  | | - | |
| OS02T0254700-01 |  | OS02g0254700 | | Os02g0254700 | |  | | - | |
| OS02T0255100-01 |  | OS02g0255100 | | Os02g0255100 | |  | | - | |
| OS02T0255700-01 |  | OS02g0255700 | | Os02g0255700 | |  | | - | |
| OS02T0257300-00 | Down | OS02g0257300 | | Os02g0257300 | | Down | | Down-Down | |
| OS02T0259600-01 | Down | OS02g0259600 | | Os02g0259600 | | Down | | Down-Down | |
| OS02T0265900-04 |  | OS02g0265900 | | Os02g0265900 | |  | | - | |
| OS02T0267000-01 |  | OS02g0267000 | | Os02g0267000 | |  | | - | |
| OS02T0269600-00 |  | OS02g0269600 | | Os02g0269600 | |  | | - | |
| OS02T0273100-02 |  | OS02g0273100 | | Os02g0273100 | | Down | | -Down | |
| OS02T0273700-01 |  | OS02g0273700 | | Os02g0273700 | |  | | - | |
| OS02T0274100-01 |  | OS02g0274100 | | Os02g0274100 | |  | | - | |
| OS02T0274700-01 |  | OS02g0274700 | | Os02g0274700 | |  | | - | |
| OS02T0276400-01 |  | OS02g0276400 | | Os02g0276400 | |  | | - | |
| OS02T0277600-01 |  | OS02g0277600 | | Os02g0277600 | |  | | - | |
| OS02T0280500-01 |  | OS02g0280500 | | Os02g0280500 | |  | | - | |
| OS02T0283800-01 |  | OS02g0283800 | | Os02g0283800 | |  | | - | |
| OS02T0284500-01 |  | OS02g0284500 | | Os02g0284500 | |  | | - | |
| OS02T0285800-01 |  | OS02g0285800 | | Os02g0285800 | |  | | - | |
| OS02T0287000-01 |  | OS02g0287000 | | Os02g0287000 | |  | | - | |
| OS02T0288200-01 |  | OS02g0288200 | | Os02g0288200 | |  | | - | |
| OS02T0290600-02 |  | OS02g0290600 | | Os02g0290600 | |  | | - | |
| OS02T0294600-01 |  | OS02g0294600 | | Os02g0294600 | |  | | - | |
| OS02T0301000-01 |  | OS02g0301000 | | Os02g0301000 | |  | | - | |
| OS02T0302200-01 |  | OS02g0302200 | | Os02g0302200 | |  | | - | |
| OS02T0306401-00 |  | OS02g0306401 | | Os02g0306401 | | Down | | -Down | |
| OS02T0307000-01 |  | OS02g0307000 | | Os02g0307000 | |  | | - | |
| OS02T0307800-02 |  | OS02g0307800 | | Os02g0307800 | |  | | - | |
| OS02T0313400-01 |  | OS02g0313400 | | Os02g0313400 | |  | | - | |
| OS02T0319100-01 |  | OS02g0319100 | | Os02g0319100 | |  | | - | |
| OS02T0319300-01 |  | OS02g0319300 | | Os02g0319300 | |  | | - | |
| OS02T0320300-01 |  | OS02g0320300 | | Os02g0320300 | |  | | - | |
| OS02T0321500-01 |  | OS02g0321500 | | Os02g0321500 | |  | | - | |
| OS02T0321900-02 |  | OS02g0321900 | | Os02g0321900 | |  | | - | |
| OS02T0325100-02 |  | OS02g0325100 | | Os02g0325100 | |  | | - | |
| OS02T0328300-02 |  | OS02g0328300 | | Os02g0328300 | |  | | - | |
| OS02T0332200-01 |  | OS02g0332200 | | Os02g0332200 | |  | | - | |
| OS02T0435000-02 |  | OS02g0435000 | | Os02g0435000 | |  | | - | |
| OS02T0436400-01 |  | OS02g0436400 | | Os02g0436400 | |  | | - | |
| OS02T0437800-02 |  | OS02g0437800 | | Os02g0437800 | |  | | - | |
| OS02T0439700-01 |  | OS02g0439700 | | Os02g0439700 | |  | | - | |
| OS02T0440000-01 |  | OS02g0440000 | | Os02g0440000 | |  | | - | |
| OS02T0460200-02 |  | OS02g0460200 | | Os02g0460200 | | Up | | -Up | |
| OS02T0462900-01 | Up | OS02g0462900 | | Os02g0462900 | |  | | Up- | |
| OS02T0469600-01 |  | OS02g0469600 | | Os02g0469600 | |  | | - | |
| OS02T0469900-01 |  | OS02g0469900 | | Os02g0469900 | |  | | - | |
| OS02T0473200-01 |  | OS02g0473200 | | Os02g0473200 | | Up | | -Up | |
| OS02T0474700-01 |  | OS02g0474700 | | Os02g0474700 | |  | | - | |
| OS02T0478900-00 |  | OS02g0478900 | | Os02g0478900 | |  | | - | |
| OS02T0480900-01 |  | OS02g0480900 | | Os02g0480900 | |  | | - | |
| OS02T0485000-01 |  | OS02g0485000 | | Os02g0485000 | |  | | - | |
| OS02T0489400-01 |  | OS02g0489400 | | Os02g0489400 | |  | | - | |
| OS02T0495900-01 |  | OS02g0495900 | | Os02g0495900 | |  | | - | |
| OS02T0496900-01 |  | OS02g0496900 | | Os02g0496900 | |  | | - | |
| OS02T0497700-01 |  | OS02g0497700 | | Os02g0497700 | |  | | - | |
| OS02T0504500-02 |  | OS02g0504500 | | Os02g0504500 | |  | | - | |
| OS02T0506500-02 |  | OS02g0506500 | | Os02g0506500 | |  | | - | |
| OS02T0507400-02 |  | OS02g0507400 | | Os02g0507400 | |  | | - | |
| OS02T0508000-01 |  | OS02g0508000 | | Os02g0508000 | |  | | - | |
| OS02T0510100-02 |  | OS02g0510100 | | Os02g0510100 | |  | | - | |
| OS02T0511500-01 |  | OS02g0511500 | | Os02g0511500 | |  | | - | |
| OS02T0516800-00 |  | OS02g0516800 | | Os02g0516800 | |  | | - | |
| OS02T0519300-01 |  | OS02g0519300 | | Os02g0519300 | |  | | - | |
| OS02T0519900-01 |  | OS02g0519900 | | Os02g0519900 | |  | | - | |
| OS02T0520800-01 |  | OS02g0520800 | | Os02g0520800 | |  | | - | |
| OS02T0521300-01 |  | OS02g0521300 | | Os02g0521300 | |  | | - | |
| OS02T0521700-01 |  | OS02g0521700 | | Os02g0521700 | |  | | - | |
| OS02T0523500-01 |  | OS02g0523500 | | Os02g0523500 | |  | | - | |
| OS02T0524600-01 |  | OS02g0524600 | | Os02g0524600 | |  | | - | |
| OS02T0525900-02 |  | OS02g0525900 | | Os02g0525900 | |  | | - | |
| OS02T0526400-01 |  | OS02g0526400 | | Os02g0526400 | | Up | | -Up | |
| OS02T0528200-01 |  | OS02g0528200 | | Os02g0528200 | |  | | - | |
| OS02T0529400-02 |  | OS02g0529400 | | Os02g0529400 | |  | | - | |
| OS02T0530100-01 |  | OS02g0530100 | | Os02g0530100 | | Down | | -Down | |
| OS02T0532500-01 |  | OS02g0532500 | | Os02g0532500 | |  | | - | |
| OS02T0533000-00 |  | OS02g0533000 | | Os02g0533000 | |  | | - | |
| OS02T0533200-00 | Up | OS02g0533200 | | Os02g0533200 | |  | | Up- | |
| OS02T0533300-01 |  | OS02g0533300 | | Os02g0533300 | |  | | - | |
| OS02T0533800-01 |  | OS02g0533800 | | Os02g0533800 | |  | | - | |
| OS02T0533900-02 |  | OS02g0533900 | | Os02g0533900 | |  | | - | |
| OS02T0534400-01 | Up | OS02g0534400 | | Os02g0534400 | |  | | Up- | |
| OS02T0535700-01 |  | OS02g0535700 | | Os02g0535700 | |  | | - | |
| OS02T0537700-01 |  | OS02g0537700 | | Os02g0537700 | |  | | - | |
| OS02T0537900-01 |  | OS02g0537900 | | Os02g0537900 | |  | | - | |
| OS02T0538000-01 |  | OS02g0538000 | | Os02g0538000 | |  | | - | |
| OS02T0538700-02 |  | OS02g0538700 | | Os02g0538700 | |  | | - | |
| OS02T0539500-01 |  | OS02g0539500 | | Os02g0539500 | |  | | - | |
| OS02T0539600-01 |  | OS02g0539600 | | Os02g0539600 | |  | | - | |
| OS02T0541700-01 |  | OS02g0541700 | | Os02g0541700 | |  | | - | |
| OS02T0542400-01 |  | OS02g0542400 | | Os02g0542400 | |  | | - | |
| OS02T0543300-00 |  | OS02g0543300 | | Os02g0543300 | |  | | - | |
| OS02T0550700-01 |  | OS02g0550700 | | Os02g0550700 | |  | | - | |
| OS02T0550800-01 |  | OS02g0550800 | | Os02g0550800 | |  | | - | |
| OS02T0551100-01 |  | OS02g0551100 | | Os02g0551100 | |  | | - | |
| OS02T0551400-01 |  | OS02g0551400 | | Os02g0551400 | |  | | - | |
| OS02T0551900-01 |  | OS02g0551900 | | Os02g0551900 | |  | | - | |
| OS02T0553200-01 | Down | OS02g0553200 | | Os02g0553200 | | Down | | Down-Down | |
| OS02T0553600-01 |  | OS02g0553600 | | Os02g0553600 | |  | | - | |
| OS02T0554100-01 |  | OS02g0554100 | | Os02g0554100 | |  | | - | |
| OS02T0554300-01 | Down | OS02g0554300 | | Os02g0554300 | |  | | Down- | |
| OS02T0556100-01 | Up | OS02g0556100 | | Os02g0556100 | |  | | Up- | |
| OS02T0556600-01 |  | OS02g0556600 | | Os02g0556600 | |  | | - | |
| OS02T0556800-01 |  | OS02g0556800 | | Os02g0556800 | | Up | | -Up | |
| OS02T0557600-01 |  | OS02g0557600 | | Os02g0557600 | |  | | - | |
| OS02T0557700-01 |  | OS02g0557700 | | Os02g0557700 | |  | | - | |
| OS02T0558300-01 |  | OS02g0558300 | | Os02g0558300 | |  | | - | |
| OS02T0560450-00 |  | OS02g0560450 | | Os02g0560450 | |  | | - | |
| OS02T0562300-01 |  | OS02g0562300 | | Os02g0562300 | |  | | - | |
| OS02T0562700-02 |  | OS02g0562700 | | Os02g0562700 | |  | | - | |
| OS02T0564000-01 |  | OS02g0564000 | | Os02g0564000 | |  | | - | |
| OS02T0564200-02 |  | OS02g0564200 | | Os02g0564200 | |  | | - | |
| OS02T0564400-01 | Down | OS02g0564400 | | Os02g0564400 | |  | | Down- | |
| OS02T0567100-01 |  | OS02g0567100 | | Os02g0567100 | |  | | - | |
| OS02T0567800-01 |  | OS02g0567800 | | Os02g0567800 | |  | | - | |
| OS02T0570700-01 |  | OS02g0570700 | | Os02g0570700 | |  | | - | |
| OS02T0573200-01 |  | OS02g0573200 | | Os02g0573200 | |  | | - | |
| OS02T0575500-01 |  | OS02g0575500 | | Os02g0575500 | |  | | - | |
| OS02T0576700-01 |  | OS02g0576700 | | Os02g0576700 | |  | | - | |
| OS02T0578400-01 |  | OS02g0578400 | | Os02g0578400 | | Down | | -Down | |
| OS02T0580300-03 |  | OS02g0580300 | | Os02g0580300 | |  | | - | |
| OS02T0580900-01 |  | OS02g0580900 | | Os02g0580900 | | Up | | -Up | |
| OS02T0581100-01 | Down | OS02g0581100 | | Os02g0581100 | | Down | | Down-Down | |
| OS02T0582400-01 |  | OS02g0582400 | | Os02g0582400 | |  | | - | |
| OS02T0586400-01 |  | OS02g0586400 | | Os02g0586400 | |  | | - | |
| OS02T0586500-02 |  | OS02g0586500 | | Os02g0586500 | |  | | - | |
| OS02T0588500-02 |  | OS02g0588500 | | Os02g0588500 | |  | | - | |
| OS02T0589400-01 |  | OS02g0589400 | | Os02g0589400 | |  | | - | |
| OS02T0591700-01 |  | OS02g0591700 | | Os02g0591700 | |  | | - | |
| OS02T0591800-02 |  | OS02g0591800 | | Os02g0591800 | |  | | - | |
| OS02T0591900-02 |  | OS02g0591900 | | Os02g0591900 | |  | | - | |
| OS02T0592500-01 |  | OS02g0592500 | | Os02g0592500 | |  | | - | |
| OS02T0593500-01 |  | OS02g0593500 | | Os02g0593500 | |  | | - | |
| OS02T0593700-04 |  | OS02g0593700 | | Os02g0593700 | |  | | - | |
| OS02T0595500-01 |  | OS02g0595500 | | Os02g0595500 | |  | | - | |
| OS02T0595700-04 |  | OS02g0595700 | | Os02g0595700 | |  | | - | |
| OS02T0595800-01 |  | OS02g0595800 | | Os02g0595800 | | Up | | -Up | |
| OS02T0596000-01 |  | OS02g0596000 | | Os02g0596000 | | Down | | -Down | |
| OS02T0596500-01 |  | OS02g0596500 | | Os02g0596500 | |  | | - | |
| OS02T0600200-01 |  | OS02g0600200 | | Os02g0600200 | |  | | - | |
| OS02T0601100-01 |  | OS02g0601100 | | Os02g0601100 | |  | | - | |
| OS02T0601300-01 |  | OS02g0601300 | | Os02g0601300 | |  | | - | |
| OS02T0601800-03 |  | OS02g0601800 | | Os02g0601800 | |  | | - | |
| OS02T0602300-02 |  | OS02g0602300 | | Os02g0602300 | |  | | - | |
| OS02T0602500-02 |  | OS02g0602500 | | Os02g0602500 | |  | | - | |
| OS02T0603800-01 |  | OS02g0603800 | | Os02g0603800 | |  | | - | |
| OS02T0605600-01 |  | OS02g0605600 | | Os02g0605600 | |  | | - | |
| OS02T0605900-01 |  | OS02g0605900 | | Os02g0605900 | | Down | | -Down | |
| OS02T0606100-00 |  | OS02g0606100 | | Os02g0606100 | |  | | - | |
| OS02T0608400-01 |  | OS02g0608400 | | Os02g0608400 | |  | | - | |
| OS02T0608500-02 |  | OS02g0608500 | | Os02g0608500 | |  | | - | |
| OS02T0608900-02 |  | OS02g0608900 | | Os02g0608900 | |  | | - | |
| OS02T0609400-02 |  | OS02g0609400 | | Os02g0609400 | |  | | - | |
| OS02T0610700-01 |  | OS02g0610700 | | Os02g0610700 | | Up | | -Up | |
| OS02T0610800-01 |  | OS02g0610800 | | Os02g0610800 | |  | | - | |
| OS02T0611400-01 |  | OS02g0611400 | | Os02g0611400 | |  | | - | |
| OS02T0611800-01 |  | OS02g0611800 | | Os02g0611800 | | Up | | -Up | |
| OS02T0612000-01 |  | OS02g0612000 | | Os02g0612000 | |  | | - | |
| OS02T0612800-01 |  | OS02g0612800 | | Os02g0612800 | |  | | - | |
| OS02T0612900-01 |  | OS02g0612900 | | Os02g0612900 | |  | | - | |
| OS02T0618100-01 |  | OS02g0618100 | | Os02g0618100 | |  | | - | |
| OS02T0618250-01 |  | OS02g0618250 | | Os02g0618250 | |  | | - | |
| OS02T0621700-01 |  | OS02g0621700 | | Os02g0621700 | |  | | - | |
| OS02T0622400-01 |  | OS02g0622400 | | Os02g0622400 | |  | | - | |
| OS02T0623500-02 |  | OS02g0623500 | | Os02g0623500 | |  | | - | |
| OS02T0625500-02 |  | OS02g0625500 | | Os02g0625500 | |  | | - | |
| OS02T0626100-01 | Down | OS02g0626100 | | Os02g0626100 | |  | | Down- | |
| OS02T0626400-01 |  | OS02g0626400 | | Os02g0626400 | |  | | - | |
| OS02T0629200-02 |  | OS02g0629200 | | Os02g0629200 | |  | | - | |
| OS02T0633200-01 |  | OS02g0633200 | | Os02g0633200 | |  | | - | |
| OS02T0633300-01 |  | OS02g0633300 | | Os02g0633300 | |  | | - | |
| OS02T0633400-01 |  | OS02g0633400 | | Os02g0633400 | |  | | - | |
| OS02T0634500-01 |  | OS02g0634500 | | Os02g0634500 | |  | | - | |
| OS02T0634600-02 |  | OS02g0634600 | | Os02g0634600 | |  | | - | |
| OS02T0634700-02 | Up | OS02g0634700 | | Os02g0634700 | |  | | Up- | |
| OS02T0634800-01 |  | OS02g0634800 | | Os02g0634800 | |  | | - | |
| OS02T0635000-01 |  | OS02g0635000 | | Os02g0635000 | | Down | | -Down | |
| OS02T0635200-01 |  | OS02g0635200 | | Os02g0635200 | |  | | - | |
| OS02T0637900-01 |  | OS02g0637900 | | Os02g0637900 | |  | | - | |
| OS02T0638300-01 |  | OS02g0638300 | | Os02g0638300 | |  | | - | |
| OS02T0639000-01 |  | OS02g0639000 | | Os02g0639000 | |  | | - | |
| OS02T0639900-01 |  | OS02g0639900 | | Os02g0639900 | |  | | - | |
| OS02T0643000-01 |  | OS02g0643000 | | Os02g0643000 | | Up | | -Up | |
| OS02T0643500-01 |  | OS02g0643500 | | Os02g0643500 | |  | | - | |
| OS02T0644100-01 |  | OS02g0644100 | | Os02g0644100 | |  | | - | |
| OS02T0649700-01 |  | OS02g0649700 | | Os02g0649700 | |  | | - | |
| OS02T0649800-01 |  | OS02g0649800 | | Os02g0649800 | |  | | - | |
| OS02T0651300-02 |  | OS02g0651300 | | Os02g0651300 | |  | | - | |
| OS02T0652600-02 |  | OS02g0652600 | | Os02g0652600 | | Down | | -Down | |
| OS02T0653800-01 |  | OS02g0653800 | | Os02g0653800 | |  | | - | |
| OS02T0654100-01 |  | OS02g0654100 | | Os02g0654100 | |  | | - | |
| OS02T0654400-01 |  | OS02g0654400 | | Os02g0654400 | |  | | - | |
| OS02T0654500-01 |  | OS02g0654500 | | Os02g0654500 | |  | | - | |
| OS02T0655300-03 |  | OS02g0655300 | | Os02g0655300 | |  | | - | |
| OS02T0656500-01 |  | OS02g0656500 | | Os02g0656500 | |  | | - | |
| OS02T0661000-00 |  | OS02g0661000 | | Os02g0661000 | |  | | - | |
| OS02T0664000-01 |  | OS02g0664000 | | Os02g0664000 | |  | | - | |
| OS02T0664100-00 |  | OS02g0664100 | | Os02g0664100 | |  | | - | |
| OS02T0664300-01 |  | OS02g0664300 | | Os02g0664300 | |  | | - | |
| OS02T0666200-02 |  | OS02g0666200 | | Os02g0666200 | |  | | - | |
| OS02T0668100-03 |  | OS02g0668100 | | Os02g0668100 | |  | | - | |
| OS02T0668400-00 |  | OS02g0668400 | | Os02g0668400 | |  | | - | |
| OS02T0669100-01 |  | OS02g0669100 | | Os02g0669100 | |  | | - | |
| OS02T0670500-01 |  | OS02g0670500 | | Os02g0670500 | |  | | - | |
| OS02T0672100-01 |  | OS02g0672100 | | Os02g0672100 | |  | | - | |
| OS02T0672200-01 |  | OS02g0672200 | | Os02g0672200 | |  | | - | |
| OS02T0672600-01 |  | OS02g0672600 | | Os02g0672600 | |  | | - | |
| OS02T0677600-01 | Down | OS02g0677600 | | Os02g0677600 | | Down | | Down-Down | |
| OS02T0678400-01 |  | OS02g0678400 | | Os02g0678400 | |  | | - | |
| OS02T0680400-02 |  | OS02g0680400 | | Os02g0680400 | |  | | - | |
| OS02T0680600-01 |  | OS02g0680600 | | Os02g0680600 | |  | | - | |
| OS02T0680700-01 |  | OS02g0680700 | | Os02g0680700 | | Up | | -Up | |
| OS02T0686400-02 |  | OS02g0686400 | | Os02g0686400 | |  | | - | |
| OS02T0686600-01 |  | OS02g0686600 | | Os02g0686600 | |  | | - | |
| OS02T0687900-01 |  | OS02g0687900 | | Os02g0687900 | |  | | - | |
| OS02T0690700-01 |  | OS02g0690700 | | Os02g0690700 | |  | | - | |
| OS02T0690800-02 |  | OS02g0690800 | | Os02g0690800 | |  | | - | |
| OS02T0697200-01 |  | OS02g0697200 | | Os02g0697200 | |  | | - | |
| OS02T0697300-01 |  | OS02g0697300 | | Os02g0697300 | |  | | - | |
| OS02T0697600-01 |  | OS02g0697600 | | Os02g0697600 | |  | | - | |
| OS02T0697700-01 |  | OS02g0697700 | | Os02g0697700 | |  | | - | |
| OS02T0698000-03 |  | OS02g0698000 | | Os02g0698000 | |  | | - | |
| OS02T0700400-01 |  | OS02g0700400 | | Os02g0700400 | |  | | - | |
| OS02T0701600-01 |  | OS02g0701600 | | Os02g0701600 | |  | | - | |
| OS02T0704000-01 |  | OS02g0704000 | | Os02g0704000 | |  | | - | |
| OS02T0704300-01 | Down | OS02g0704300 | | Os02g0704300 | | Up | | Down-Up | |
| OS02T0704800-01 |  | OS02g0704800 | | Os02g0704800 | |  | | - | |
| OS02T0704900-02 |  | OS02g0704900 | | Os02g0704900 | |  | | - | |
| OS02T0706500-01 |  | OS02g0706500 | | Os02g0706500 | |  | | - | |
| OS02T0707100-01 |  | OS02g0707100 | | Os02g0707100 | |  | | - | |
| OS02T0707900-02 |  | OS02g0707900 | | Os02g0707900 | |  | | - | |
| OS02T0708100-02 |  | OS02g0708100 | | Os02g0708100 | |  | | - | |
| OS02T0708200-01 |  | OS02g0708200 | | Os02g0708200 | |  | | - | |
| OS02T0709200-01 |  | OS02g0709200 | | Os02g0709200 | |  | | - | |
| OS02T0709800-01 |  | OS02g0709800 | | Os02g0709800 | |  | | - | |
| OS02T0710900-01 |  | OS02g0710900 | | Os02g0710900 | |  | | - | |
| OS02T0711200-01 |  | OS02g0711200 | | Os02g0711200 | |  | | - | |
| OS02T0712000-01 |  | OS02g0712000 | | Os02g0712000 | |  | | - | |
| OS02T0713400-01 |  | OS02g0713400 | | Os02g0713400 | |  | | - | |
| OS02T0714200-01 |  | OS02g0714200 | | Os02g0714200 | | Down | | -Down | |
| OS02T0714600-02 |  | OS02g0714600 | | Os02g0714600 | |  | | - | |
| OS02T0717300-01 |  | OS02g0717300 | | Os02g0717300 | |  | | - | |
| OS02T0717400-01 |  | OS02g0717400 | | Os02g0717400 | |  | | - | |
| OS02T0717500-01 | Up | OS02g0717500 | | Os02g0717500 | | Up | | Up-Up | |
| OS02T0718900-02 | Up | OS02g0718900 | | Os02g0718900 | |  | | Up- | |
| OS02T0719800-01 |  | OS02g0719800 | | Os02g0719800 | |  | | - | |
| OS02T0720000-01 |  | OS02g0720000 | | Os02g0720000 | |  | | - | |
| OS02T0720600-01 |  | OS02g0720600 | | Os02g0720600 | |  | | - | |
| OS02T0720900-04 |  | OS02g0720900 | | Os02g0720900 | |  | | - | |
| OS02T0722500-01 |  | OS02g0722500 | | Os02g0722500 | |  | | - | |
| OS02T0722800-01 |  | OS02g0722800 | | Os02g0722800 | |  | | - | |
| OS02T0725100-01 |  | OS02g0725100 | | Os02g0725100 | |  | | - | |
| OS02T0725200-01 |  | OS02g0725200 | | Os02g0725200 | |  | | - | |
| OS02T0725600-01 |  | OS02g0725600 | | Os02g0725600 | |  | | - | |
| OS02T0726400-02 |  | OS02g0726400 | | Os02g0726400 | |  | | - | |
| OS02T0726600-01 |  | OS02g0726600 | | Os02g0726600 | |  | | - | |
| OS02T0728100-02 | Down | OS02g0728100 | | Os02g0728100 | |  | | Down- | |
| OS02T0729400-01 |  | OS02g0729400 | | Os02g0729400 | |  | | - | |
| OS02T0730800-01 |  | OS02g0730800 | | Os02g0730800 | |  | | - | |
| OS02T0731600-01 |  | OS02g0731600 | | Os02g0731600 | |  | | - | |
| OS02T0733800-01 |  | OS02g0733800 | | Os02g0733800 | |  | | - | |
| OS02T0734600-02 |  | OS02g0734600 | | Os02g0734600 | |  | | - | |
| OS02T0735100-01 | Up | OS02g0735100 | | Os02g0735100 | |  | | Up- | |
| OS02T0735200-03 |  | OS02g0735200 | | Os02g0735200 | | Up | | -Up | |
| OS02T0736400-01 |  | OS02g0736400 | | Os02g0736400 | |  | | - | |
| OS02T0738900-01 |  | OS02g0738900 | | Os02g0738900 | |  | | - | |
| OS02T0739000-01 |  | OS02g0739000 | | Os02g0739000 | |  | | - | |
| OS02T0739600-01 |  | OS02g0739600 | | Os02g0739600 | |  | | - | |
| OS02T0740300-01 |  | OS02g0740300 | | Os02g0740300 | |  | | - | |
| OS02T0740400-02 |  | OS02g0740400 | | Os02g0740400 | |  | | - | |
| OS02T0741400-01 |  | OS02g0741400 | | Os02g0741400 | |  | | - | |
| OS02T0741900-01 |  | OS02g0741900 | | Os02g0741900 | |  | | - | |
| OS02T0742000-01 |  | OS02g0742000 | | Os02g0742000 | |  | | - | |
| OS02T0744100-01 |  | OS02g0744100 | | Os02g0744100 | |  | | - | |
| OS02T0744200-01 |  | OS02g0744200 | | Os02g0744200 | |  | | - | |
| OS02T0744700-01 | Down | OS02g0744700 | | Os02g0744700 | |  | | Down- | |
| OS02T0744900-01 |  | OS02g0744900 | | Os02g0744900 | |  | | - | |
| OS02T0745000-01 | Up | OS02g0745000 | | Os02g0745000 | |  | | Up- | |
| OS02T0749150-01 |  | OS02g0749150 | | Os02g0749150 | |  | | - | |
| OS02T0749800-01 |  | OS02g0749800 | | Os02g0749800 | |  | | - | |
| OS02T0750100-00 |  | OS02g0750100 | | Os02g0750100 | |  | | - | |
| OS02T0750600-01 |  | OS02g0750600 | | Os02g0750600 | |  | | - | |
| OS02T0751600-02 | Down | OS02g0751600 | | Os02g0751600 | |  | | Down- | |
| OS02T0752000-02 |  | OS02g0752000 | | Os02g0752000 | |  | | - | |
| OS02T0752200-01 | Down | OS02g0752200 | | Os02g0752200 | |  | | Down- | |
| OS02T0753200-01 |  | OS02g0753200 | | Os02g0753200 | |  | | - | |
| OS02T0753300-01 |  | OS02g0753300 | | Os02g0753300 | |  | | - | |
| OS02T0753800-01 |  | OS02g0753800 | | Os02g0753800 | |  | | - | |
| OS02T0754300-01 | Down | OS02g0754300 | | Os02g0754300 | | Down | | Down-Down | |
| OS02T0754700-02 |  | OS02g0754700 | | Os02g0754700 | | Down | | -Down | |
| OS02T0756800-01 |  | OS02g0756800 | | Os02g0756800 | |  | | - | |
| OS02T0758000-01 |  | OS02g0758000 | | Os02g0758000 | | Down | | -Down | |
| OS02T0759800-01 |  | OS02g0759800 | | Os02g0759800 | |  | | - | |
| OS02T0760300-01 |  | OS02g0760300 | | Os02g0760300 | |  | | - | |
| OS02T0760500-01 |  | OS02g0760500 | | Os02g0760500 | |  | | - | |
| OS02T0761400-01 |  | OS02g0761400 | | Os02g0761400 | |  | | - | |
| OS02T0761700-01 |  | OS02g0761700 | | Os02g0761700 | |  | | - | |
| OS02T0761900-02 |  | OS02g0761900 | | Os02g0761900 | |  | | - | |
| OS02T0762100-02 |  | OS02g0762100 | | Os02g0762100 | |  | | - | |
| OS02T0766000-01 |  | OS02g0766000 | | Os02g0766000 | |  | | - | |
| OS02T0767100-01 |  | OS02g0767100 | | Os02g0767100 | |  | | - | |
| OS02T0767500-01 |  | OS02g0767500 | | Os02g0767500 | |  | | - | |
| OS02T0767700-01 |  | OS02g0767700 | | Os02g0767700 | |  | | - | |
| OS02T0768600-02 | Down | OS02g0768600 | | Os02g0768600 | |  | | Down- | |
| OS02T0768700-01 |  | OS02g0768700 | | Os02g0768700 | |  | | - | |
| OS02T0770000-02 |  | OS02g0770000 | | Os02g0770000 | |  | | - | |
| OS02T0771100-02 |  | OS02g0771100 | | Os02g0771100 | |  | | - | |
| OS02T0771200-01 |  | OS02g0771200 | | Os02g0771200 | |  | | - | |
| OS02T0771400-00 |  | OS02g0771400 | | Os02g0771400 | |  | | - | |
| OS02T0771600-02 |  | OS02g0771600 | | Os02g0771600 | |  | | - | |
| OS02T0771700-01 | Down | OS02g0771700 | | Os02g0771700 | |  | | Down- | |
| OS02T0772000-00 |  | OS02g0772000 | | Os02g0772000 | |  | | - | |
| OS02T0773300-01 | Up | OS02g0773300 | | Os02g0773300 | |  | | Up- | |
| OS02T0774100-00 |  | OS02g0774100 | | Os02g0774100 | |  | | - | |
| OS02T0774300-01 |  | OS02g0774300 | | Os02g0774300 | |  | | - | |
| OS02T0778200-01 | Down | OS02g0778200 | | Os02g0778200 | |  | | Down- | |
| OS02T0779200-02 |  | OS02g0779200 | | Os02g0779200 | |  | | - | |
| OS02T0782200-02 |  | OS02g0782200 | | Os02g0782200 | |  | | - | |
| OS02T0782500-02 |  | OS02g0782500 | | Os02g0782500 | |  | | - | |
| OS02T0785800-02 |  | OS02g0785800 | | Os02g0785800 | |  | | - | |
| OS02T0786000-01 |  | OS02g0786000 | | Os02g0786000 | |  | | - | |
| OS02T0786200-01 |  | OS02g0786200 | | Os02g0786200 | |  | | - | |
| OS02T0787300-01 |  | OS02g0787300 | | Os02g0787300 | |  | | - | |
| OS02T0787800-01 |  | OS02g0787800 | | Os02g0787800 | |  | | - | |
| OS02T0788400-01 |  | OS02g0788400 | | Os02g0788400 | |  | | - | |
| OS02T0788500-01 |  | OS02g0788500 | | Os02g0788500 | | Down | | -Down | |
| OS02T0789400-02 |  | OS02g0789400 | | Os02g0789400 | |  | | - | |
| OS02T0789600-01 |  | OS02g0789600 | | Os02g0789600 | |  | | - | |
| OS02T0792800-02 | Up | OS02g0792800 | | Os02g0792800 | |  | | Up- | |
| OS02T0793100-01 |  | OS02g0793100 | | Os02g0793100 | |  | | - | |
| OS02T0793700-01 |  | OS02g0793700 | | Os02g0793700 | |  | | - | |
| OS02T0794400-01 |  | OS02g0794400 | | Os02g0794400 | |  | | - | |
| OS02T0794700-02 |  | OS02g0794700 | | Os02g0794700 | |  | | - | |
| OS02T0794900-00 |  | OS02g0794900 | | Os02g0794900 | |  | | - | |
| OS02T0795300-02 |  | OS02g0795300 | | Os02g0795300 | |  | | - | |
| OS02T0797500-02 |  | OS02g0797500 | | Os02g0797500 | |  | | - | |
| OS02T0797700-01 |  | OS02g0797700 | | Os02g0797700 | |  | | - | |
| OS02T0800000-01 |  | OS02g0800000 | | Os02g0800000 | |  | | - | |
| OS02T0803200-01 |  | OS02g0803200 | | Os02g0803200 | |  | | - | |
| OS02T0803700-01 |  | OS02g0803700 | | Os02g0803700 | |  | | - | |
| OS02T0804500-01 |  | OS02g0804500 | | Os02g0804500 | |  | | - | |
| OS02T0805200-01 |  | OS02g0805200 | | Os02g0805200 | |  | | - | |
| OS02T0805600-02 |  | OS02g0805600 | | Os02g0805600 | |  | | - | |
| OS02T0805900-01 |  | OS02g0805900 | | Os02g0805900 | |  | | - | |
| OS02T0806600-03 |  | OS02g0806600 | | Os02g0806600 | |  | | - | |
| OS02T0806700-01 |  | OS02g0806700 | | Os02g0806700 | |  | | - | |
| OS02T0810300-01 |  | OS02g0810300 | | Os02g0810300 | |  | | - | |
| OS02T0810450-01 |  | OS02g0810450 | | Os02g0810450 | |  | | - | |
| OS02T0812400-01 |  | OS02g0812400 | | Os02g0812400 | |  | | - | |
| OS02T0813350-01 |  | OS02g0813350 | | Os02g0813350 | |  | | - | |
| OS02T0813500-01 | Up | OS02g0813500 | | Os02g0813500 | |  | | Up- | |
| OS02T0814800-01 |  | OS02g0814800 | | Os02g0814800 | |  | | - | |
| OS02T0815200-01 |  | OS02g0815200 | | Os02g0815200 | |  | | - | |
| OS02T0815400-01 |  | OS02g0815400 | | Os02g0815400 | |  | | - | |
| OS02T0815500-01 |  | OS02g0815500 | | Os02g0815500 | |  | | - | |
| OS02T0815700-01 |  | OS02g0815700 | | Os02g0815700 | |  | | - | |
| OS02T0816100-02 |  | OS02g0816100 | | Os02g0816100 | |  | | - | |
| OS02T0816500-01 |  | OS02g0816500 | | Os02g0816500 | |  | | - | |
| OS02T0816600-02 |  | OS02g0816600 | | Os02g0816600 | | Up | | -Up | |
| OS02T0816800-01 |  | OS02g0816800 | | Os02g0816800 | |  | | - | |
| OS02T0817200-02 |  | OS02g0817200 | | Os02g0817200 | |  | | - | |
| OS02T0817500-02 |  | OS02g0817500 | | Os02g0817500 | |  | | - | |
| OS02T0817700-02 |  | OS02g0817700 | | Os02g0817700 | |  | | - | |
| OS02T0817900-01 |  | OS02g0817900 | | Os02g0817900 | |  | | - | |
| OS02T0818000-01 |  | OS02g0818000 | | Os02g0818000 | | Down | | -Down | |
| OS02T0819200-01 |  | OS02g0819200 | | Os02g0819200 | |  | | - | |
| OS02T0819300-01 |  | OS02g0819300 | | Os02g0819300 | |  | | - | |
| OS02T0820000-01 |  | OS02g0820000 | | Os02g0820000 | |  | | - | |
| OS02T0820400-02 |  | OS02g0820400 | | Os02g0820400 | |  | | - | |
| OS02T0821200-01 |  | OS02g0821200 | | Os02g0821200 | |  | | - | |
| OS02T0821800-02 |  | OS02g0821800 | | Os02g0821800 | |  | | - | |
| OS02T0821900-01 |  | OS02g0821900 | | Os02g0821900 | |  | | - | |
| OS02T0822200-02 |  | OS02g0822200 | | Os02g0822200 | |  | | - | |
| OS02T0822300-00 |  | OS02g0822300 | | Os02g0822300 | |  | | - | |
| OS02T0822600-01 | Down | OS02g0822600 | | Os02g0822600 | | Down | | Down-Down | |
| OS02T0823000-01 |  | OS02g0823000 | | Os02g0823000 | |  | | - | |
| OS02T0823100-01 | Up | OS02g0823100 | | Os02g0823100 | | Up | | Up-Up | |
| OS02T0823400-01 |  | OS02g0823400 | | Os02g0823400 | | Down | | -Down | |
| OS02T0826500-01 |  | OS02g0826500 | | Os02g0826500 | |  | | - | |
| OS02T0827300-01 |  | OS02g0827300 | | Os02g0827300 | |  | | - | |
| OS02T0827600-01 |  | OS02g0827600 | | Os02g0827600 | | Down | | -Down | |
| OS02T0828200-01 |  | OS02g0828200 | | Os02g0828200 | |  | | - | |
| OS02T0829500-02 |  | OS02g0829500 | | Os02g0829500 | |  | | - | |
| OS02T0829800-01 |  | OS02g0829800 | | Os02g0829800 | |  | | - | |
| OS02T0831100-01 |  | OS02g0831100 | | Os02g0831100 | |  | | - | |
| OS02T0831500-01 |  | OS02g0831500 | | Os02g0831500 | |  | | - | |
| OS02T0831600-01 |  | OS02g0831600 | | Os02g0831600 | |  | | - | |
| OS02T0831800-01 |  | OS02g0831800 | | Os02g0831800 | |  | | - | |
| OS02T0832400-02 |  | OS02g0832400 | | Os02g0832400 | |  | | - | |
| OS02T0832600-02 |  | OS02g0832600 | | Os02g0832600 | |  | | - | |
| OS02T0832800-01 |  | OS02g0832800 | | Os02g0832800 | |  | | - | |
| OS02T0833400-01 |  | OS02g0833400 | | Os02g0833400 | |  | | - | |
| OS02T0833900-03 |  | OS02g0833900 | | Os02g0833900 | |  | | - | |
| OS02T0834700-01 |  | OS02g0834700 | | Os02g0834700 | |  | | - | |
| OS03T0100040-01 |  | OS03g0100040 | | Os03g0100040 | |  | | - | |
| OS03T0100200-01 |  | OS03g0100200 | | Os03g0100200 | |  | | - | |
| OS03T0101100-01 |  | OS03g0101100 | | Os03g0101100 | |  | | - | |
| OS03T0102000-01 |  | OS03g0102000 | | Os03g0102000 | |  | | - | |
| OS03T0102100-01 |  | OS03g0102100 | | Os03g0102100 | |  | | - | |
| OS03T0102400-01 |  | OS03g0102400 | | Os03g0102400 | |  | | - | |
| OS03T0103200-01 |  | OS03g0103200 | | Os03g0103200 | |  | | - | |
| OS03T0103300-00 |  | OS03g0103300 | | Os03g0103300 | |  | | - | |
| OS03T0104400-02 |  | OS03g0104400 | | Os03g0104400 | |  | | - | |
| OS03T0105300-02 |  | OS03g0105300 | | Os03g0105300 | |  | | - | |
| OS03T0107800-02 |  | OS03g0107800 | | Os03g0107800 | |  | | - | |
| OS03T0109600-02 |  | OS03g0109600 | | Os03g0109600 | |  | | - | |
| OS03T0109700-02 |  | OS03g0109700 | | Os03g0109700 | |  | | - | |
| OS03T0109800-01 |  | OS03g0109800 | | Os03g0109800 | |  | | - | |
| OS03T0110400-02 |  | OS03g0110400 | | Os03g0110400 | |  | | - | |
| OS03T0110900-01 |  | OS03g0110900 | | Os03g0110900 | |  | | - | |
| OS03T0111100-01 |  | OS03g0111100 | | Os03g0111100 | |  | | - | |
| OS03T0111400-01 |  | OS03g0111400 | | Os03g0111400 | |  | | - | |
| OS03T0111800-01 |  | OS03g0111800 | | Os03g0111800 | |  | | - | |
| OS03T0112101-00 |  | OS03g0112101 | | Os03g0112101 | |  | | - | |
| OS03T0113700-01 | Up | OS03g0113700 | | Os03g0113700 | |  | | Up- | |
| OS03T0114900-01 |  | OS03g0114900 | | Os03g0114900 | |  | | - | |
| OS03T0115100-01 |  | OS03g0115100 | | Os03g0115100 | |  | | - | |
| OS03T0115500-01 |  | OS03g0115500 | | Os03g0115500 | |  | | - | |
| OS03T0116500-01 |  | OS03g0116500 | | Os03g0116500 | |  | | - | |
| OS03T0117100-02 |  | OS03g0117100 | | Os03g0117100 | |  | | - | |
| OS03T0117200-01 |  | OS03g0117200 | | Os03g0117200 | |  | | - | |
| OS03T0117800-01 |  | OS03g0117800 | | Os03g0117800 | |  | | - | |
| OS03T0118400-01 |  | OS03g0118400 | | Os03g0118400 | |  | | - | |
| OS03T0118400-03 |  | OS03g0118400 | | Os03g0118400 | |  | | - | |
| OS03T0118600-01 |  | OS03g0118600 | | Os03g0118600 | | Down | | -Down | |
| OS03T0121700-01 |  | OS03g0121700 | | Os03g0121700 | |  | | - | |
| OS03T0122200-01 | Down | OS03g0122200 | | Os03g0122200 | | Down | | Down-Down | |
| OS03T0123600-02 |  | OS03g0123600 | | Os03g0123600 | |  | | - | |
| OS03T0125000-02 | Down | OS03g0125000 | | Os03g0125000 | | Down | | Down-Down | |
| OS03T0126000-01 |  | OS03g0126000 | | Os03g0126000 | |  | | - | |
| OS03T0126100-01 |  | OS03g0126100 | | Os03g0126100 | |  | | - | |
| OS03T0126300-01 |  | OS03g0126300 | | Os03g0126300 | |  | | - | |
| OS03T0128800-03 |  | OS03g0128800 | | Os03g0128800 | |  | | - | |
| OS03T0129200-00 |  | OS03g0129200 | | Os03g0129200 | |  | | - | |
| OS03T0129300-01 |  | OS03g0129300 | | Os03g0129300 | |  | | - | |
| OS03T0129900-01 |  | OS03g0129900 | | Os03g0129900 | |  | | - | |
| OS03T0130500-01 |  | OS03g0130500 | | Os03g0130500 | |  | | - | |
| OS03T0131200-02 |  | OS03g0131200 | | Os03g0131200 | |  | | - | |
| OS03T0131300-02 |  | OS03g0131300 | | Os03g0131300 | |  | | - | |
| OS03T0131500-01 |  | OS03g0131500 | | Os03g0131500 | |  | | - | |
| OS03T0131900-01 |  | OS03g0131900 | | Os03g0131900 | |  | | - | |
| OS03T0132000-01 |  | OS03g0132000 | | Os03g0132000 | | Up | | -Up | |
| OS03T0132800-01 |  | OS03g0132800 | | Os03g0132800 | |  | | - | |
| OS03T0132900-01 |  | OS03g0132900 | | Os03g0132900 | | Down | | -Down | |
| OS03T0133400-01 | Up | OS03g0133400 | | Os03g0133400 | |  | | Up- | |
| OS03T0134300-01 |  | OS03g0134300 | | Os03g0134300 | |  | | - | |
| OS03T0135100-01 |  | OS03g0135100 | | Os03g0135100 | |  | | - | |
| OS03T0135300-01 | Up | OS03g0135300 | | Os03g0135300 | |  | | Up- | |
| OS03T0135600-01 |  | OS03g0135600 | | Os03g0135600 | |  | | - | |
| OS03T0136500-01 |  | OS03g0136500 | | Os03g0136500 | |  | | - | |
| OS03T0136900-01 |  | OS03g0136900 | | Os03g0136900 | |  | | - | |
| OS03T0137500-03 |  | OS03g0137500 | | Os03g0137500 | |  | | - | |
| OS03T0137600-02 |  | OS03g0137600 | | Os03g0137600 | | Down | | -Down | |
| OS03T0140700-01 |  | OS03g0140700 | | Os03g0140700 | |  | | - | |
| OS03T0143300-01 |  | OS03g0143300 | | Os03g0143300 | |  | | - | |
| OS03T0143400-01 | Up | OS03g0143400 | | Os03g0143400 | |  | | Up- | |
| OS03T0146000-01 |  | OS03g0146000 | | Os03g0146000 | |  | | - | |
| OS03T0146100-03 |  | OS03g0146100 | | Os03g0146100 | |  | | - | |
| OS03T0146400-02 |  | OS03g0146400 | | Os03g0146400 | | Up | | -Up | |
| OS03T0146500-02 |  | OS03g0146500 | | Os03g0146500 | |  | | - | |
| OS03T0147700-02 |  | OS03g0147700 | | Os03g0147700 | |  | | - | |
| OS03T0150600-01 |  | OS03g0150600 | | Os03g0150600 | | Down | | -Down | |
| OS03T0151700-01 |  | OS03g0151700 | | Os03g0151700 | |  | | - | |
| OS03T0151800-02 |  | OS03g0151800 | | Os03g0151800 | |  | | - | |
| OS03T0151900-02 |  | OS03g0151900 | | Os03g0151900 | |  | | - | |
| OS03T0152400-01 |  | OS03g0152400 | | Os03g0152400 | |  | | - | |
| OS03T0152700-01 |  | OS03g0152700 | | Os03g0152700 | |  | | - | |
| OS03T0152800-02 |  | OS03g0152800 | | Os03g0152800 | |  | | - | |
| OS03T0157400-01 |  | OS03g0157400 | | Os03g0157400 | |  | | - | |
| OS03T0157800-02 |  | OS03g0157800 | | Os03g0157800 | |  | | - | |
| OS03T0157900-01 |  | OS03g0157900 | | Os03g0157900 | |  | | - | |
| OS03T0158200-01 | Up | OS03g0158200 | | Os03g0158200 | |  | | Up- | |
| OS03T0158300-01 |  | OS03g0158300 | | Os03g0158300 | |  | | - | |
| OS03T0158500-03 |  | OS03g0158500 | | Os03g0158500 | |  | | - | |
| OS03T0159200-01 |  | OS03g0159200 | | Os03g0159200 | |  | | - | |
| OS03T0161200-01 |  | OS03g0161200 | | Os03g0161200 | |  | | - | |
| OS03T0161800-01 |  | OS03g0161800 | | Os03g0161800 | |  | | - | |
| OS03T0163100-01 |  | OS03g0163100 | | Os03g0163100 | |  | | - | |
| OS03T0163300-01 |  | OS03g0163300 | | Os03g0163300 | |  | | - | |
| OS03T0164700-01 |  | OS03g0164700 | | Os03g0164700 | |  | | - | |
| OS03T0165300-02 |  | OS03g0165300 | | Os03g0165300 | |  | | - | |
| OS03T0168200-01 |  | OS03g0168200 | | Os03g0168200 | |  | | - | |
| OS03T0168700-00 |  | OS03g0168700 | | Os03g0168700 | |  | | - | |
| OS03T0169100-01 |  | OS03g0169100 | | Os03g0169100 | |  | | - | |
| OS03T0170300-01 |  | OS03g0170300 | | Os03g0170300 | |  | | - | |
| OS03T0170400-01 |  | OS03g0170400 | | Os03g0170400 | |  | | - | |
| OS03T0170500-00 |  | OS03g0170500 | | Os03g0170500 | |  | | - | |
| OS03T0170900-01 |  | OS03g0170900 | | Os03g0170900 | | Up | | -Up | |
| OS03T0171900-02 |  | OS03g0171900 | | Os03g0171900 | |  | | - | |
| OS03T0174100-02 |  | OS03g0174100 | | Os03g0174100 | |  | | - | |
| OS03T0174500-01 |  | OS03g0174500 | | Os03g0174500 | |  | | - | |
| OS03T0175600-01 |  | OS03g0175600 | | Os03g0175600 | |  | | - | |
| OS03T0176700-00 |  | OS03g0176700 | | Os03g0176700 | |  | | - | |
| OS03T0179000-01 |  | OS03g0179000 | | Os03g0179000 | |  | | - | |
| OS03T0179900-01 |  | OS03g0179900 | | Os03g0179900 | |  | | - | |
| OS03T0180300-02 |  | OS03g0180300 | | Os03g0180300 | |  | | - | |
| OS03T0180400-01 |  | OS03g0180400 | | Os03g0180400 | |  | | - | |
| OS03T0182400-01 |  | OS03g0182400 | | Os03g0182400 | |  | | - | |
| OS03T0182600-02 |  | OS03g0182600 | | Os03g0182600 | |  | | - | |
| OS03T0182700-02 |  | OS03g0182700 | | Os03g0182700 | |  | | - | |
| OS03T0183100-02 |  | OS03g0183100 | | Os03g0183100 | |  | | - | |
| OS03T0184000-00 |  | OS03g0184000 | | Os03g0184000 | |  | | - | |
| OS03T0185700-01 |  | OS03g0185700 | | Os03g0185700 | |  | | - | |
| OS03T0186100-01 |  | OS03g0186100 | | Os03g0186100 | |  | | - | |
| OS03T0186900-01 |  | OS03g0186900 | | Os03g0186900 | |  | | - | |
| OS03T0186950-01 |  | OS03g0186950 | | Os03g0186950 | |  | | - | |
| OS03T0187000-01 |  | OS03g0187000 | | Os03g0187000 | |  | | - | |
| OS03T0187600-01 |  | OS03g0187600 | | Os03g0187600 | |  | | - | |
| OS03T0189400-01 | Up | OS03g0189400 | | Os03g0189400 | |  | | Up- | |
| OS03T0191000-01 |  | OS03g0191000 | | Os03g0191000 | |  | | - | |
| OS03T0192400-01 |  | OS03g0192400 | | Os03g0192400 | |  | | - | |
| OS03T0192700-01 |  | OS03g0192700 | | Os03g0192700 | |  | | - | |
| OS03T0194100-01 |  | OS03g0194100 | | Os03g0194100 | |  | | - | |
| OS03T0194500-01 |  | OS03g0194500 | | Os03g0194500 | |  | | - | |
| OS03T0196400-01 |  | OS03g0196400 | | Os03g0196400 | | Up | | -Up | |
| OS03T0196800-01 | Down | OS03g0196800 | | Os03g0196800 | |  | | Down- | |
| OS03T0197000-01 |  | OS03g0197000 | | Os03g0197000 | | Up | | -Up | |
| OS03T0197100-01 |  | OS03g0197100 | | Os03g0197100 | | Up | | -Up | |
| OS03T0197400-02 |  | OS03g0197400 | | Os03g0197400 | |  | | - | |
| OS03T0198300-02 |  | OS03g0198300 | | Os03g0198300 | |  | | - | |
| OS03T0199000-01 |  | OS03g0199000 | | Os03g0199000 | |  | | - | |
| OS03T0200500-02 |  | OS03g0200500 | | Os03g0200500 | |  | | - | |
| OS03T0200700-02 |  | OS03g0200700 | | Os03g0200700 | |  | | - | |
| OS03T0200800-01 | Up | OS03g0200800 | | Os03g0200800 | |  | | Up- | |
| OS03T0201100-01 |  | OS03g0201100 | | Os03g0201100 | |  | | - | |
| OS03T0201600-02 |  | OS03g0201600 | | Os03g0201600 | |  | | - | |
| OS03T0202200-01 |  | OS03g0202200 | | Os03g0202200 | |  | | - | |
| OS03T0203200-01 |  | OS03g0203200 | | Os03g0203200 | |  | | - | |
| OS03T0205000-01 |  | OS03g0205000 | | Os03g0205000 | |  | | - | |
| OS03T0206300-01 |  | OS03g0206300 | | Os03g0206300 | |  | | - | |
| OS03T0206700-00 |  | OS03g0206700 | | Os03g0206700 | |  | | - | |
| OS03T0207250-01 |  | OS03g0207250 | | Os03g0207250 | |  | | - | |
| OS03T0207900-02 |  | OS03g0207900 | | Os03g0207900 | |  | | - | |
| OS03T0208900-01 |  | OS03g0208900 | | Os03g0208900 | |  | | - | |
| OS03T0209000-01 |  | OS03g0209000 | | Os03g0209000 | |  | | - | |
| OS03T0209600-01 |  | OS03g0209600 | | Os03g0209600 | |  | | - | |
| OS03T0210200-01 |  | OS03g0210200 | | Os03g0210200 | |  | | - | |
| OS03T0210400-01 |  | OS03g0210400 | | Os03g0210400 | |  | | - | |
| OS03T0210600-01 |  | OS03g0210600 | | Os03g0210600 | |  | | - | |
| OS03T0212700-01 |  | OS03g0212700 | | Os03g0212700 | |  | | - | |
| OS03T0213300-01 |  | OS03g0213300 | | Os03g0213300 | |  | | - | |
| OS03T0213400-01 |  | OS03g0213400 | | Os03g0213400 | |  | | - | |
| OS03T0214600-02 | Up | OS03g0214600 | | Os03g0214600 | |  | | Up- | |
| OS03T0215900-00 |  | OS03g0215900 | | Os03g0215900 | |  | | - | |
| OS03T0216600-01 | Down | OS03g0216600 | | Os03g0216600 | |  | | Down- | |
| OS03T0218400-01 |  | OS03g0218400 | | Os03g0218400 | |  | | - | |
| OS03T0218500-01 |  | OS03g0218500 | | Os03g0218500 | |  | | - | |
| OS03T0219200-01 |  | OS03g0219200 | | Os03g0219200 | |  | | - | |
| OS03T0219300-01 |  | OS03g0219300 | | Os03g0219300 | |  | | - | |
| OS03T0219700-01 |  | OS03g0219700 | | Os03g0219700 | |  | | - | |
| OS03T0219900-01 | Down | OS03g0219900 | | Os03g0219900 | | Down | | Down-Down | |
| OS03T0222600-01 |  | OS03g0222600 | | Os03g0222600 | |  | | - | |
| OS03T0223400-01 |  | OS03g0223400 | | Os03g0223400 | |  | | - | |
| OS03T0223700-01 |  | OS03g0223700 | | Os03g0223700 | |  | | - | |
| OS03T0224200-03 |  | OS03g0224200 | | Os03g0224200 | |  | | - | |
| OS03T0225500-01 |  | OS03g0225500 | | Os03g0225500 | |  | | - | |
| OS03T0225700-01 |  | OS03g0225700 | | Os03g0225700 | |  | | - | |
| OS03T0226700-01 |  | OS03g0226700 | | Os03g0226700 | |  | | - | |
| OS03T0227000-01 |  | OS03g0227000 | | Os03g0227000 | |  | | - | |
| OS03T0227400-01 |  | OS03g0227400 | | Os03g0227400 | |  | | - | |
| OS03T0232800-01 |  | OS03g0232800 | | Os03g0232800 | |  | | - | |
| OS03T0234600-00 |  | OS03g0234600 | | Os03g0234600 | |  | | - | |
| OS03T0234900-01 |  | OS03g0234900 | | Os03g0234900 | |  | | - | |
| OS03T0235000-01 |  | OS03g0235000 | | Os03g0235000 | |  | | - | |
| OS03T0235100-02 |  | OS03g0235100 | | Os03g0235100 | |  | | - | |
| OS03T0237000-01 |  | OS03g0237000 | | Os03g0237000 | |  | | - | |
| OS03T0237100-01 |  | OS03g0237100 | | Os03g0237100 | |  | | - | |
| OS03T0238600-01 |  | OS03g0238600 | | Os03g0238600 | | Up | | -Up | |
| OS03T0239200-04 |  | OS03g0239200 | | Os03g0239200 | |  | | - | |
| OS03T0240500-02 |  | OS03g0240500 | | Os03g0240500 | |  | | - | |
| OS03T0240800-02 |  | OS03g0240800 | | Os03g0240800 | |  | | - | |
| OS03T0241200-00 |  | OS03g0241200 | | Os03g0241200 | |  | | - | |
| OS03T0242900-01 |  | OS03g0242900 | | Os03g0242900 | |  | | - | |
| OS03T0243300-01 |  | OS03g0243300 | | Os03g0243300 | |  | | - | |
| OS03T0246800-02 |  | OS03g0246800 | | Os03g0246800 | |  | | - | |
| OS03T0246800-04 |  | OS03g0246800 | | Os03g0246800 | |  | | - | |
| OS03T0247000-01 |  | OS03g0247000 | | Os03g0247000 | |  | | - | |
| OS03T0248600-02 | Up | OS03g0248600 | | Os03g0248600 | | Up | | Up-Up | |
| OS03T0249200-01 |  | OS03g0249200 | | Os03g0249200 | |  | | - | |
| OS03T0249300-01 |  | OS03g0249300 | | Os03g0249300 | |  | | - | |
| OS03T0249400-01 |  | OS03g0249400 | | Os03g0249400 | | Down | | -Down | |
| OS03T0250000-02 |  | OS03g0250000 | | Os03g0250000 | |  | | - | |
| OS03T0251350-00 |  | OS03g0251350 | | Os03g0251350 | | Down | | -Down | |
| OS03T0251500-01 | Up | OS03g0251500 | | Os03g0251500 | |  | | Up- | |
| OS03T0252100-01 |  | OS03g0252100 | | Os03g0252100 | |  | | - | |
| OS03T0253100-01 |  | OS03g0253100 | | Os03g0253100 | |  | | - | |
| OS03T0253500-00 |  | OS03g0253500 | | Os03g0253500 | |  | | - | |
| OS03T0253800-03 |  | OS03g0253800 | | Os03g0253800 | |  | | - | |
| OS03T0254700-01 |  | OS03g0254700 | | Os03g0254700 | |  | | - | |
| OS03T0254800-01 |  | OS03g0254800 | | Os03g0254800 | |  | | - | |
| OS03T0255100-01 |  | OS03g0255100 | | Os03g0255100 | | Up | | -Up | |
| OS03T0255500-01 |  | OS03g0255500 | | Os03g0255500 | |  | | - | |
| OS03T0256400-01 |  | OS03g0256400 | | Os03g0256400 | |  | | - | |
| OS03T0259300-01 |  | OS03g0259300 | | Os03g0259300 | |  | | - | |
| OS03T0261500-02 |  | OS03g0261500 | | Os03g0261500 | |  | | - | |
| OS03T0262100-01 |  | OS03g0262100 | | Os03g0262100 | |  | | - | |
| OS03T0262400-01 |  | OS03g0262400 | | Os03g0262400 | |  | | - | |
| OS03T0263500-01 |  | OS03g0263500 | | Os03g0263500 | |  | | - | |
| OS03T0263900-01 |  | OS03g0263900 | | Os03g0263900 | |  | | - | |
| OS03T0264700-01 |  | OS03g0264700 | | Os03g0264700 | |  | | - | |
| OS03T0265100-01 |  | OS03g0265100 | | Os03g0265100 | |  | | - | |
| OS03T0265400-02 | Down | OS03g0265400 | | Os03g0265400 | | Down | | Down-Down | |
| OS03T0265500-02 |  | OS03g0265500 | | Os03g0265500 | |  | | - | |
| OS03T0265900-01 | Down | OS03g0265900 | | Os03g0265900 | |  | | Down- | |
| OS03T0266000-01 |  | OS03g0266000 | | Os03g0266000 | |  | | - | |
| OS03T0266100-01 |  | OS03g0266100 | | Os03g0266100 | |  | | - | |
| OS03T0266200-01 |  | OS03g0266200 | | Os03g0266200 | |  | | - | |
| OS03T0266700-01 |  | OS03g0266700 | | Os03g0266700 | |  | | - | |
| OS03T0266900-02 |  | OS03g0266900 | | Os03g0266900 | |  | | - | |
| OS03T0267300-01 |  | OS03g0267300 | | Os03g0267300 | |  | | - | |
| OS03T0268000-01 |  | OS03g0268000 | | Os03g0268000 | |  | | - | |
| OS03T0268400-01 |  | OS03g0268400 | | Os03g0268400 | |  | | - | |
| OS03T0268900-01 |  | OS03g0268900 | | Os03g0268900 | |  | | - | |
| OS03T0269000-02 |  | OS03g0269000 | | Os03g0269000 | |  | | - | |
| OS03T0269100-01 |  | OS03g0269100 | | Os03g0269100 | |  | | - | |
| OS03T0271200-01 |  | OS03g0271200 | | Os03g0271200 | |  | | - | |
| OS03T0274000-01 |  | OS03g0274000 | | Os03g0274000 | |  | | - | |
| OS03T0275500-01 |  | OS03g0275500 | | Os03g0275500 | |  | | - | |
| OS03T0276500-01 |  | OS03g0276500 | | Os03g0276500 | |  | | - | |
| OS03T0277600-01 | Up | OS03g0277600 | | Os03g0277600 | |  | | Up- | |
| OS03T0277700-01 |  | OS03g0277700 | | Os03g0277700 | |  | | - | |
| OS03T0278000-02 | Down | OS03g0278000 | | Os03g0278000 | |  | | Down- | |
| OS03T0278200-01 | Down | OS03g0278200 | | Os03g0278200 | |  | | Down- | |
| OS03T0278300-02 |  | OS03g0278300 | | Os03g0278300 | |  | | - | |
| OS03T0278400-01 |  | OS03g0278400 | | Os03g0278400 | |  | | - | |
| OS03T0278900-01 |  | OS03g0278900 | | Os03g0278900 | |  | | - | |
| OS03T0279000-00 |  | OS03g0279000 | | Os03g0279000 | |  | | - | |
| OS03T0279400-02 | Up | OS03g0279400 | | Os03g0279400 | |  | | Up- | |
| OS03T0279600-02 |  | OS03g0279600 | | Os03g0279600 | |  | | - | |
| OS03T0279900-01 |  | OS03g0279900 | | Os03g0279900 | |  | | - | |
| OS03T0279950-01 |  | OS03g0279950 | | Os03g0279950 | | Down | | -Down | |
| OS03T0280000-01 |  | OS03g0280000 | | Os03g0280000 | |  | | - | |
| OS03T0281600-01 |  | OS03g0281600 | | Os03g0281600 | |  | | - | |
| OS03T0281600-02 |  | OS03g0281600 | | Os03g0281600 | |  | | - | |
| OS03T0282900-02 |  | OS03g0282900 | | Os03g0282900 | |  | | - | |
| OS03T0283100-02 | Up | OS03g0283100 | | Os03g0283100 | |  | | Up- | |
| OS03T0283300-01 |  | OS03g0283300 | | Os03g0283300 | |  | | - | |
| OS03T0283600-02 |  | OS03g0283600 | | Os03g0283600 | |  | | - | |
| OS03T0284400-01 |  | OS03g0284400 | | Os03g0284400 | | Down | | -Down | |
| OS03T0284500-01 |  | OS03g0284500 | | Os03g0284500 | |  | | - | |
| OS03T0284600-01 |  | OS03g0284600 | | Os03g0284600 | |  | | - | |
| OS03T0284800-01 |  | OS03g0284800 | | Os03g0284800 | |  | | - | |
| OS03T0285100-01 |  | OS03g0285100 | | Os03g0285100 | |  | | - | |
| OS03T0285700-01 |  | OS03g0285700 | | Os03g0285700 | |  | | - | |
| OS03T0285800-02 |  | OS03g0285800 | | Os03g0285800 | |  | | - | |
| OS03T0285900-01 |  | OS03g0285900 | | Os03g0285900 | |  | | - | |
| OS03T0286200-01 |  | OS03g0286200 | | Os03g0286200 | |  | | - | |
| OS03T0287900-01 |  | OS03g0287900 | | Os03g0287900 | |  | | - | |
| OS03T0288400-01 |  | OS03g0288400 | | Os03g0288400 | |  | | - | |
| OS03T0289400-00 |  | OS03g0289400 | | Os03g0289400 | | Down | | -Down | |
| OS03T0290300-01 |  | OS03g0290300 | | Os03g0290300 | | Up | | -Up | |
| OS03T0292200-01 |  | OS03g0292200 | | Os03g0292200 | |  | | - | |
| OS03T0293100-01 | Down | OS03g0293100 | | Os03g0293100 | |  | | Down- | |
| OS03T0293500-02 |  | OS03g0293500 | | Os03g0293500 | |  | | - | |
| OS03T0295400-01 |  | OS03g0295400 | | Os03g0295400 | |  | | - | |
| OS03T0295500-01 |  | OS03g0295500 | | Os03g0295500 | |  | | - | |
| OS03T0295800-02 | Up | OS03g0295800 | | Os03g0295800 | |  | | Up- | |
| OS03T0296400-03 |  | OS03g0296400 | | Os03g0296400 | |  | | - | |
| OS03T0297100-01 |  | OS03g0297100 | | Os03g0297100 | |  | | - | |
| OS03T0297400-01 |  | OS03g0297400 | | Os03g0297400 | |  | | - | |
| OS03T0298400-01 |  | OS03g0298400 | | Os03g0298400 | |  | | - | |
| OS03T0298800-01 |  | OS03g0298800 | | Os03g0298800 | |  | | - | |
| OS03T0299900-01 |  | OS03g0299900 | | Os03g0299900 | |  | | - | |
| OS03T0300300-01 |  | OS03g0300300 | | Os03g0300300 | |  | | - | |
| OS03T0300400-01 |  | OS03g0300400 | | Os03g0300400 | | Up | | -Up | |
| OS03T0305000-01 |  | OS03g0305000 | | Os03g0305000 | |  | | - | |
| OS03T0305100-01 |  | OS03g0305100 | | Os03g0305100 | |  | | - | |
| OS03T0305500-02 | Up | OS03g0305500 | | Os03g0305500 | |  | | Up- | |
| OS03T0305600-01 |  | OS03g0305600 | | Os03g0305600 | | Up | | -Up | |
| OS03T0305700-01 |  | OS03g0305700 | | Os03g0305700 | |  | | - | |
| OS03T0306800-01 |  | OS03g0306800 | | Os03g0306800 | |  | | - | |
| OS03T0307100-01 |  | OS03g0307100 | | Os03g0307100 | |  | | - | |
| OS03T0307200-01 |  | OS03g0307200 | | Os03g0307200 | |  | | - | |
| OS03T0307400-01 |  | OS03g0307400 | | Os03g0307400 | |  | | - | |
| OS03T0308100-03 |  | OS03g0308100 | | Os03g0308100 | |  | | - | |
| OS03T0308500-01 |  | OS03g0308500 | | Os03g0308500 | |  | | - | |
| OS03T0308800-03 |  | OS03g0308800 | | Os03g0308800 | |  | | - | |
| OS03T0309000-01 |  | OS03g0309000 | | Os03g0309000 | |  | | - | |
| OS03T0309200-01 |  | OS03g0309200 | | Os03g0309200 | |  | | - | |
| OS03T0309800-02 |  | OS03g0309800 | | Os03g0309800 | |  | | - | |
| OS03T0310400-01 |  | OS03g0310400 | | Os03g0310400 | |  | | - | |
| OS03T0310500-01 |  | OS03g0310500 | | Os03g0310500 | |  | | - | |
| OS03T0310800-01 |  | OS03g0310800 | | Os03g0310800 | |  | | - | |
| OS03T0311300-02 |  | OS03g0311300 | | Os03g0311300 | |  | | - | |
| OS03T0311300-03 |  | OS03g0311300 | | Os03g0311300 | |  | | - | |
| OS03T0313000-01 |  | OS03g0313000 | | Os03g0313000 | |  | | - | |
| OS03T0313600-01 | Up | OS03g0313600 | | Os03g0313600 | |  | | Up- | |
| OS03T0314100-01 |  | OS03g0314100 | | Os03g0314100 | |  | | - | |
| OS03T0314700-01 |  | OS03g0314700 | | Os03g0314700 | |  | | - | |
| OS03T0315800-01 | Down | OS03g0315800 | | Os03g0315800 | |  | | Down- | |
| OS03T0316900-01 |  | OS03g0316900 | | Os03g0316900 | |  | | - | |
| OS03T0317000-01 |  | OS03g0317000 | | Os03g0317000 | |  | | - | |
| OS03T0318500-01 |  | OS03g0318500 | | Os03g0318500 | |  | | - | |
| OS03T0320900-01 |  | OS03g0320900 | | Os03g0320900 | |  | | - | |
| OS03T0322500-01 |  | OS03g0322500 | | Os03g0322500 | |  | | - | |
| OS03T0322600-01 |  | OS03g0322600 | | Os03g0322600 | |  | | - | |
| OS03T0323200-02 |  | OS03g0323200 | | Os03g0323200 | |  | | - | |
| OS03T0323800-01 |  | OS03g0323800 | | Os03g0323800 | |  | | - | |
| OS03T0324800-01 |  | OS03g0324800 | | Os03g0324800 | |  | | - | |
| OS03T0326500-01 |  | OS03g0326500 | | Os03g0326500 | |  | | - | |
| OS03T0327600-01 | Down | OS03g0327600 | | Os03g0327600 | |  | | Down- | |
| OS03T0329700-01 |  | OS03g0329700 | | Os03g0329700 | |  | | - | |
| OS03T0331600-01 |  | OS03g0331600 | | Os03g0331600 | |  | | - | |
| OS03T0332400-01 | Up | OS03g0332400 | | Os03g0332400 | |  | | Up- | |
| OS03T0332700-01 | Up | OS03g0332700 | | Os03g0332700 | |  | | Up- | |
| OS03T0333200-01 |  | OS03g0333200 | | Os03g0333200 | |  | | - | |
| OS03T0333300-01 |  | OS03g0333300 | | Os03g0333300 | |  | | - | |
| OS03T0333400-01 |  | OS03g0333400 | | Os03g0333400 | | Down | | -Down | |
| OS03T0336000-01 |  | OS03g0336000 | | Os03g0336000 | |  | | - | |
| OS03T0336300-01 |  | OS03g0336300 | | Os03g0336300 | |  | | - | |
| OS03T0336500-01 |  | OS03g0336500 | | Os03g0336500 | |  | | - | |
| OS03T0337600-01 |  | OS03g0337600 | | Os03g0337600 | |  | | - | |
| OS03T0337800-01 |  | OS03g0337800 | | Os03g0337800 | |  | | - | |
| OS03T0337900-01 |  | OS03g0337900 | | Os03g0337900 | |  | | - | |
| OS03T0339100-01 |  | OS03g0339100 | | Os03g0339100 | |  | | - | |
| OS03T0340500-01 | Up | OS03g0340500 | | Os03g0340500 | |  | | Up- | |
| OS03T0340700-01 |  | OS03g0340700 | | Os03g0340700 | |  | | - | |
| OS03T0340900-01 |  | OS03g0340900 | | Os03g0340900 | |  | | - | |
| OS03T0341100-01 |  | OS03g0341100 | | Os03g0341100 | |  | | - | |
| OS03T0342900-04 |  | OS03g0342900 | | Os03g0342900 | | Down | | -Down | |
| OS03T0343400-01 |  | OS03g0343400 | | Os03g0343400 | |  | | - | |
| OS03T0343500-03 | Down | OS03g0343500 | | Os03g0343500 | |  | | Down- | |
| OS03T0344900-00 |  | OS03g0344900 | | Os03g0344900 | |  | | - | |
| OS03T0347200-01 |  | OS03g0347200 | | Os03g0347200 | |  | | - | |
| OS03T0349000-01 |  | OS03g0349000 | | Os03g0349000 | |  | | - | |
| OS03T0350100-01 |  | OS03g0350100 | | Os03g0350100 | |  | | - | |
| OS03T0351200-00 |  | OS03g0351200 | | Os03g0351200 | |  | | - | |
| OS03T0351500-01 | Up | OS03g0351500 | | Os03g0351500 | |  | | Up- | |
| OS03T0352300-01 | Down | OS03g0352300 | | Os03g0352300 | |  | | Down- | |
| OS03T0352450-00 |  | OS03g0352450 | | Os03g0352450 | |  | | - | |
| OS03T0352800-01 |  | OS03g0352800 | | Os03g0352800 | |  | | - | |
| OS03T0355100-01 |  | OS03g0355100 | | Os03g0355100 | |  | | - | |
| OS03T0355600-01 |  | OS03g0355600 | | Os03g0355600 | |  | | - | |
| OS03T0356484-01 |  | OS03g0356484 | | Os03g0356484 | |  | | - | |
| OS03T0356700-01 |  | OS03g0356700 | | Os03g0356700 | |  | | - | |
| OS03T0358000-00 |  | OS03g0358000 | | Os03g0358000 | |  | | - | |
| OS03T0358100-01 | Up | OS03g0358100 | | Os03g0358100 | |  | | Up- | |
| OS03T0360600-00 |  | OS03g0360600 | | Os03g0360600 | |  | | - | |
| OS03T0360700-01 | Up | OS03g0360700 | | Os03g0360700 | |  | | Up- | |
| OS03T0363400-01 |  | OS03g0363400 | | Os03g0363400 | |  | | - | |
| OS03T0363800-01 |  | OS03g0363800 | | Os03g0363800 | |  | | - | |
| OS03T0364000-01 |  | OS03g0364000 | | Os03g0364000 | |  | | - | |
| OS03T0364400-02 |  | OS03g0364400 | | Os03g0364400 | |  | | - | |
| OS03T0366000-01 |  | OS03g0366000 | | Os03g0366000 | |  | | - | |
| OS03T0367000-01 |  | OS03g0367000 | | Os03g0367000 | |  | | - | |
| OS03T0370250-01 |  | OS03g0370250 | | Os03g0370250 | |  | | - | |
| OS03T0375200-00 |  | OS03g0375200 | | Os03g0375200 | |  | | - | |
| OS03T0376100-02 |  | OS03g0376100 | | Os03g0376100 | |  | | - | |
| OS03T0376600-01 |  | OS03g0376600 | | Os03g0376600 | | Down | | -Down | |
| OS03T0376800-05 |  | OS03g0376800 | | Os03g0376800 | |  | | - | |
| OS03T0381500-01 |  | OS03g0381500 | | Os03g0381500 | |  | | - | |
| OS03T0383800-02 |  | OS03g0383800 | | Os03g0383800 | |  | | - | |
| OS03T0385400-01 |  | OS03g0385400 | | Os03g0385400 | |  | | - | |
| OS03T0385900-03 |  | OS03g0385900 | | Os03g0385900 | |  | | - | |
| OS03T0387000-01 |  | OS03g0387000 | | Os03g0387000 | |  | | - | |
| OS03T0387100-01 |  | OS03g0387100 | | Os03g0387100 | |  | | - | |
| OS03T0387800-01 |  | OS03g0387800 | | Os03g0387800 | |  | | - | |
| OS03T0388100-02 |  | OS03g0388100 | | Os03g0388100 | |  | | - | |
| OS03T0388900-01 |  | OS03g0388900 | | Os03g0388900 | |  | | - | |
| OS03T0389700-01 |  | OS03g0389700 | | Os03g0389700 | |  | | - | |
| OS03T0390000-02 |  | OS03g0390000 | | Os03g0390000 | |  | | - | |
| OS03T0390400-01 |  | OS03g0390400 | | Os03g0390400 | |  | | - | |
| OS03T0390600-02 |  | OS03g0390600 | | Os03g0390600 | |  | | - | |
| OS03T0390700-01 |  | OS03g0390700 | | Os03g0390700 | |  | | - | |
| OS03T0391000-01 |  | OS03g0391000 | | Os03g0391000 | |  | | - | |
| OS03T0393700-01 |  | OS03g0393700 | | Os03g0393700 | |  | | - | |
| OS03T0395300-01 |  | OS03g0395300 | | Os03g0395300 | |  | | - | |
| OS03T0395600-01 |  | OS03g0395600 | | Os03g0395600 | |  | | - | |
| OS03T0397500-01 |  | OS03g0397500 | | Os03g0397500 | |  | | - | |
| OS03T0397600-00 |  | OS03g0397600 | | Os03g0397600 | |  | | - | |
| OS03T0399800-01 |  | OS03g0399800 | | Os03g0399800 | |  | | - | |
| OS03T0401100-01 |  | OS03g0401100 | | Os03g0401100 | |  | | - | |
| OS03T0405000-01 |  | OS03g0405000 | | Os03g0405000 | |  | | - | |
| OS03T0405100-01 | Up | OS03g0405100 | | Os03g0405100 | |  | | Up- | |
| OS03T0405500-01 |  | OS03g0405500 | | Os03g0405500 | |  | | - | |
| OS03T0405900-01 |  | OS03g0405900 | | Os03g0405900 | |  | | - | |
| OS03T0406200-02 |  | OS03g0406200 | | Os03g0406200 | |  | | - | |
| OS03T0407000-01 |  | OS03g0407000 | | Os03g0407000 | |  | | - | |
| OS03T0408300-00 |  | OS03g0408300 | | Os03g0408300 | |  | | - | |
| OS03T0410700-01 |  | OS03g0410700 | | Os03g0410700 | | Down | | -Down | |
| OS03T0411000-01 |  | OS03g0411000 | | Os03g0411000 | |  | | - | |
| OS03T0412200-02 |  | OS03g0412200 | | Os03g0412200 | |  | | - | |
| OS03T0412800-01 |  | OS03g0412800 | | Os03g0412800 | |  | | - | |
| OS03T0413400-01 |  | OS03g0413400 | | Os03g0413400 | |  | | - | |
| OS03T0415700-00 |  | OS03g0415700 | | Os03g0415700 | |  | | - | |
| OS03T0417800-01 |  | OS03g0417800 | | Os03g0417800 | |  | | - | |
| OS03T0418800-01 |  | OS03g0418800 | | Os03g0418800 | |  | | - | |
| OS03T0419100-02 |  | OS03g0419100 | | Os03g0419100 | |  | | - | |
| OS03T0421800-01 |  | OS03g0421800 | | Os03g0421800 | |  | | - | |
| OS03T0423800-01 |  | OS03g0423800 | | Os03g0423800 | |  | | - | |
| OS03T0425200-02 |  | OS03g0425200 | | Os03g0425200 | |  | | - | |
| OS03T0426900-01 |  | OS03g0426900 | | Os03g0426900 | |  | | - | |
| OS03T0427900-01 |  | OS03g0427900 | | Os03g0427900 | |  | | - | |
| OS03T0429000-01 |  | OS03g0429000 | | Os03g0429000 | | Down | | -Down | |
| OS03T0429800-03 |  | OS03g0429800 | | Os03g0429800 | |  | | - | |
| OS03T0430000-01 |  | OS03g0430000 | | Os03g0430000 | |  | | - | |
| OS03T0438100-02 |  | OS03g0438100 | | Os03g0438100 | |  | | - | |
| OS03T0441500-01 | Down | OS03g0441500 | | Os03g0441500 | |  | | Down- | |
| OS03T0448700-01 |  | OS03g0448700 | | Os03g0448700 | |  | | - | |
| OS03T0452300-01 | Down | OS03g0452300 | | Os03g0452300 | |  | | Down- | |
| OS03T0563300-01 |  | OS03g0563300 | | Os03g0563300 | | Down | | -Down | |
| OS03T0565100-01 |  | OS03g0565100 | | Os03g0565100 | |  | | - | |
| OS03T0565200-01 |  | OS03g0565200 | | Os03g0565200 | |  | | - | |
| OS03T0565500-01 |  | OS03g0565500 | | Os03g0565500 | |  | | - | |
| OS03T0566800-01 |  | OS03g0566800 | | Os03g0566800 | |  | | - | |
| OS03T0568900-01 |  | OS03g0568900 | | Os03g0568900 | |  | | - | |
| OS03T0569900-01 |  | OS03g0569900 | | Os03g0569900 | |  | | - | |
| OS03T0572300-01 |  | OS03g0572300 | | Os03g0572300 | |  | | - | |
| OS03T0572900-01 |  | OS03g0572900 | | Os03g0572900 | | Up | | -Up | |
| OS03T0577000-01 |  | OS03g0577000 | | Os03g0577000 | |  | | - | |
| OS03T0579000-02 |  | OS03g0579000 | | Os03g0579000 | |  | | - | |
| OS03T0579300-00 |  | OS03g0579300 | | Os03g0579300 | |  | | - | |
| OS03T0581800-01 |  | OS03g0581800 | | Os03g0581800 | |  | | - | |
| OS03T0582200-02 |  | OS03g0582200 | | Os03g0582200 | |  | | - | |
| OS03T0586500-01 | Down | OS03g0586500 | | Os03g0586500 | |  | | Down- | |
| OS03T0586700-01 |  | OS03g0586700 | | Os03g0586700 | |  | | - | |
| OS03T0586800-02 |  | OS03g0586800 | | Os03g0586800 | |  | | - | |
| OS03T0586900-01 |  | OS03g0586900 | | Os03g0586900 | |  | | - | |
| OS03T0587000-01 | Up | OS03g0587000 | | Os03g0587000 | |  | | Up- | |
| OS03T0592500-01 |  | OS03g0592500 | | Os03g0592500 | |  | | - | |
| OS03T0596900-01 |  | OS03g0596900 | | Os03g0596900 | |  | | - | |
| OS03T0598900-01 |  | OS03g0598900 | | Os03g0598900 | |  | | - | |
| OS03T0599800-01 |  | OS03g0599800 | | Os03g0599800 | |  | | - | |
| OS03T0602600-01 |  | OS03g0602600 | | Os03g0602600 | |  | | - | |
| OS03T0603600-01 | Up | OS03g0603600 | | Os03g0603600 | |  | | Up- | |
| OS03T0605300-01 |  | OS03g0605300 | | Os03g0605300 | |  | | - | |
| OS03T0606200-01 | Up | OS03g0606200 | | Os03g0606200 | |  | | Up- | |
| OS03T0610650-01 |  | OS03g0610650 | | Os03g0610650 | |  | | - | |
| OS03T0611200-01 |  | OS03g0611200 | | Os03g0611200 | |  | | - | |
| OS03T0617900-01 |  | OS03g0617900 | | Os03g0617900 | |  | | - | |
| OS03T0618300-01 |  | OS03g0618300 | | Os03g0618300 | |  | | - | |
| OS03T0619400-01 |  | OS03g0619400 | | Os03g0619400 | |  | | - | |
| OS03T0620800-01 |  | OS03g0620800 | | Os03g0620800 | |  | | - | |
| OS03T0625900-01 |  | OS03g0625900 | | Os03g0625900 | |  | | - | |
| OS03T0627500-01 |  | OS03g0627500 | | Os03g0627500 | |  | | - | |
| OS03T0628900-01 | Up | OS03g0628900 | | Os03g0628900 | |  | | Up- | |
| OS03T0638800-01 |  | OS03g0638800 | | Os03g0638800 | |  | | - | |
| OS03T0639700-01 |  | OS03g0639700 | | Os03g0639700 | |  | | - | |
| OS03T0640100-01 |  | OS03g0640100 | | Os03g0640100 | |  | | - | |
| OS03T0643300-02 |  | OS03g0643300 | | Os03g0643300 | |  | | - | |
| OS03T0646800-01 |  | OS03g0646800 | | Os03g0646800 | |  | | - | |
| OS03T0647400-01 |  | OS03g0647400 | | Os03g0647400 | |  | | - | |
| OS03T0648400-03 | Down | OS03g0648400 | | Os03g0648400 | |  | | Down- | |
| OS03T0651000-01 |  | OS03g0651000 | | Os03g0651000 | |  | | - | |
| OS03T0652100-01 |  | OS03g0652100 | | Os03g0652100 | |  | | - | |
| OS03T0654600-01 |  | OS03g0654600 | | Os03g0654600 | |  | | - | |
| OS03T0655400-01 |  | OS03g0655400 | | Os03g0655400 | |  | | - | |
| OS03T0655700-01 |  | OS03g0655700 | | Os03g0655700 | |  | | - | |
| OS03T0656100-01 |  | OS03g0656100 | | Os03g0656100 | |  | | - | |
| OS03T0656800-01 |  | OS03g0656800 | | Os03g0656800 | |  | | - | |
| OS03T0657000-01 |  | OS03g0657000 | | Os03g0657000 | |  | | - | |
| OS03T0659200-03 |  | OS03g0659200 | | Os03g0659200 | | Down | | -Down | |
| OS03T0659300-01 |  | OS03g0659300 | | Os03g0659300 | |  | | - | |
| OS03T0661600-01 | Up | OS03g0661600 | | Os03g0661600 | | Down | | Up-Down | |
| OS03T0663400-02 |  | OS03g0663400 | | Os03g0663400 | | Down | | -Down | |
| OS03T0663500-00 |  | OS03g0663500 | | Os03g0663500 | |  | | - | |
| OS03T0666200-01 |  | OS03g0666200 | | Os03g0666200 | |  | | - | |
| OS03T0667700-01 |  | OS03g0667700 | | Os03g0667700 | |  | | - | |
| OS03T0669200-01 |  | OS03g0669200 | | Os03g0669200 | |  | | - | |
| OS03T0669300-02 |  | OS03g0669300 | | Os03g0669300 | |  | | - | |
| OS03T0670100-01 |  | OS03g0670100 | | Os03g0670100 | |  | | - | |
| OS03T0670700-04 |  | OS03g0670700 | | Os03g0670700 | |  | | - | |
| OS03T0676300-01 |  | OS03g0676300 | | Os03g0676300 | |  | | - | |
| OS03T0679700-03 |  | OS03g0679700 | | Os03g0679700 | |  | | - | |
| OS03T0680800-04 |  | OS03g0680800 | | Os03g0680800 | |  | | - | |
| OS03T0681700-01 |  | OS03g0681700 | | Os03g0681700 | |  | | - | |
| OS03T0681900-01 |  | OS03g0681900 | | Os03g0681900 | |  | | - | |
| OS03T0684100-01 |  | OS03g0684100 | | Os03g0684100 | |  | | - | |
| OS03T0685000-01 |  | OS03g0685000 | | Os03g0685000 | |  | | - | |
| OS03T0685500-01 | Down | OS03g0685500 | | Os03g0685500 | |  | | Down- | |
| OS03T0685600-01 |  | OS03g0685600 | | Os03g0685600 | |  | | - | |
| OS03T0685900-01 |  | OS03g0685900 | | Os03g0685900 | |  | | - | |
| OS03T0686300-00 |  | OS03g0686300 | | Os03g0686300 | |  | | - | |
| OS03T0687000-01 |  | OS03g0687000 | | Os03g0687000 | |  | | - | |
| OS03T0688200-01 |  | OS03g0688200 | | Os03g0688200 | |  | | - | |
| OS03T0689100-01 |  | OS03g0689100 | | Os03g0689100 | |  | | - | |
| OS03T0690500-00 |  | OS03g0690500 | | Os03g0690500 | |  | | - | |
| OS03T0691400-01 |  | OS03g0691400 | | Os03g0691400 | |  | | - | |
| OS03T0691500-00 |  | OS03g0691500 | | Os03g0691500 | |  | | - | |
| OS03T0692700-05 |  | OS03g0692700 | | Os03g0692700 | |  | | - | |
| OS03T0693400-01 |  | OS03g0693400 | | Os03g0693400 | |  | | - | |
| OS03T0694900-01 |  | OS03g0694900 | | Os03g0694900 | |  | | - | |
| OS03T0695500-01 |  | OS03g0695500 | | Os03g0695500 | |  | | - | |
| OS03T0695600-03 |  | OS03g0695600 | | Os03g0695600 | |  | | - | |
| OS03T0699200-01 |  | OS03g0699200 | | Os03g0699200 | |  | | - | |
| OS03T0699300-01 |  | OS03g0699300 | | Os03g0699300 | | Down | | -Down | |
| OS03T0700700-02 |  | OS03g0700700 | | Os03g0700700 | |  | | - | |
| OS03T0701000-01 |  | OS03g0701000 | | Os03g0701000 | |  | | - | |
| OS03T0701000-02 |  | OS03g0701000 | | Os03g0701000 | |  | | - | |
| OS03T0701900-01 |  | OS03g0701900 | | Os03g0701900 | |  | | - | |
| OS03T0702800-02 |  | OS03g0702800 | | Os03g0702800 | |  | | - | |
| OS03T0703000-01 | Down | OS03g0703000 | | Os03g0703000 | |  | | Down- | |
| OS03T0703100-02 |  | OS03g0703100 | | Os03g0703100 | |  | | - | |
| OS03T0704000-00 | Down | OS03g0704000 | | Os03g0704000 | |  | | Down- | |
| OS03T0704100-01 | Up | OS03g0704100 | | Os03g0704100 | |  | | Up- | |
| OS03T0707300-01 |  | OS03g0707300 | | Os03g0707300 | |  | | - | |
| OS03T0707600-01 |  | OS03g0707600 | | Os03g0707600 | |  | | - | |
| OS03T0707900-01 |  | OS03g0707900 | | Os03g0707900 | |  | | - | |
| OS03T0708500-00 | Down | OS03g0708500 | | Os03g0708500 | |  | | Down- | |
| OS03T0708600-02 |  | OS03g0708600 | | Os03g0708600 | |  | | - | |
| OS03T0708900-01 |  | OS03g0708900 | | Os03g0708900 | |  | | - | |
| OS03T0709300-01 |  | OS03g0709300 | | Os03g0709300 | |  | | - | |
| OS03T0710800-01 | Up | OS03g0710800 | | Os03g0710800 | |  | | Up- | |
| OS03T0710900-00 |  | OS03g0710900 | | Os03g0710900 | |  | | - | |
| OS03T0711400-01 |  | OS03g0711400 | | Os03g0711400 | |  | | - | |
| OS03T0711800-01 |  | OS03g0711800 | | Os03g0711800 | |  | | - | |
| OS03T0712300-01 |  | OS03g0712300 | | Os03g0712300 | |  | | - | |
| OS03T0712400-01 |  | OS03g0712400 | | Os03g0712400 | |  | | - | |
| OS03T0712700-01 |  | OS03g0712700 | | Os03g0712700 | |  | | - | |
| OS03T0713200-01 |  | OS03g0713200 | | Os03g0713200 | | Down | | -Down | |
| OS03T0713400-01 |  | OS03g0713400 | | Os03g0713400 | |  | | - | |
| OS03T0717600-01 |  | OS03g0717600 | | Os03g0717600 | |  | | - | |
| OS03T0718100-01 |  | OS03g0718100 | | Os03g0718100 | |  | | - | |
| OS03T0718800-01 |  | OS03g0718800 | | Os03g0718800 | |  | | - | |
| OS03T0719300-01 |  | OS03g0719300 | | Os03g0719300 | |  | | - | |
| OS03T0719500-01 |  | OS03g0719500 | | Os03g0719500 | |  | | - | |
| OS03T0719500-02 |  | OS03g0719500 | | Os03g0719500 | |  | | - | |
| OS03T0719900-04 |  | OS03g0719900 | | Os03g0719900 | |  | | - | |
| OS03T0720300-01 |  | OS03g0720300 | | Os03g0720300 | |  | | - | |
| OS03T0721200-01 |  | OS03g0721200 | | Os03g0721200 | |  | | - | |
| OS03T0721400-02 |  | OS03g0721400 | | Os03g0721400 | |  | | - | |
| OS03T0722100-01 |  | OS03g0722100 | | Os03g0722100 | |  | | - | |
| OS03T0722600-01 |  | OS03g0722600 | | Os03g0722600 | |  | | - | |
| OS03T0725300-01 |  | OS03g0725300 | | Os03g0725300 | |  | | - | |
| OS03T0725400-03 |  | OS03g0725400 | | Os03g0725400 | |  | | - | |
| OS03T0729000-01 |  | OS03g0729000 | | Os03g0729000 | |  | | - | |
| OS03T0729200-01 |  | OS03g0729200 | | Os03g0729200 | |  | | - | |
| OS03T0729300-01 |  | OS03g0729300 | | Os03g0729300 | |  | | - | |
| OS03T0729700-01 |  | OS03g0729700 | | Os03g0729700 | |  | | - | |
| OS03T0730000-01 |  | OS03g0730000 | | Os03g0730000 | |  | | - | |
| OS03T0731900-01 |  | OS03g0731900 | | Os03g0731900 | |  | | - | |
| OS03T0732100-01 |  | OS03g0732100 | | Os03g0732100 | |  | | - | |
| OS03T0733400-01 |  | OS03g0733400 | | Os03g0733400 | |  | | - | |
| OS03T0733800-00 |  | OS03g0733800 | | Os03g0733800 | |  | | - | |
| OS03T0734300-01 |  | OS03g0734300 | | Os03g0734300 | |  | | - | |
| OS03T0734900-00 |  | OS03g0734900 | | Os03g0734900 | |  | | - | |
| OS03T0735300-01 | Up | OS03g0735300 | | Os03g0735300 | |  | | Up- | |
| OS03T0736400-02 |  | OS03g0736400 | | Os03g0736400 | |  | | - | |
| OS03T0737000-01 |  | OS03g0737000 | | Os03g0737000 | |  | | - | |
| OS03T0737800-01 |  | OS03g0737800 | | Os03g0737800 | |  | | - | |
| OS03T0737900-01 |  | OS03g0737900 | | Os03g0737900 | |  | | - | |
| OS03T0738400-01 |  | OS03g0738400 | | Os03g0738400 | |  | | - | |
| OS03T0738900-00 |  | OS03g0738900 | | Os03g0738900 | |  | | - | |
| OS03T0740700-01 |  | OS03g0740700 | | Os03g0740700 | |  | | - | |
| OS03T0741600-00 |  | OS03g0741600 | | Os03g0741600 | |  | | - | |
| OS03T0743400-01 |  | OS03g0743400 | | Os03g0743400 | |  | | - | |
| OS03T0743800-00 |  | OS03g0743800 | | Os03g0743800 | |  | | - | |
| OS03T0743900-01 |  | OS03g0743900 | | Os03g0743900 | |  | | - | |
| OS03T0744600-01 |  | OS03g0744600 | | Os03g0744600 | |  | | - | |
| OS03T0745000-01 |  | OS03g0745000 | | Os03g0745000 | |  | | - | |
| OS03T0746800-01 |  | OS03g0746800 | | Os03g0746800 | |  | | - | |
| OS03T0747200-01 |  | OS03g0747200 | | Os03g0747200 | |  | | - | |
| OS03T0748000-01 |  | OS03g0748000 | | Os03g0748000 | |  | | - | |
| OS03T0748200-01 |  | OS03g0748200 | | Os03g0748200 | |  | | - | |
| OS03T0748300-01 |  | OS03g0748300 | | Os03g0748300 | |  | | - | |
| OS03T0749300-02 |  | OS03g0749300 | | Os03g0749300 | | Down | | -Down | |
| OS03T0749500-01 | Down | OS03g0749500 | | Os03g0749500 | | Down | | Down-Down | |
| OS03T0749900-01 |  | OS03g0749900 | | Os03g0749900 | |  | | - | |
| OS03T0750100-00 |  | OS03g0750100 | | Os03g0750100 | |  | | - | |
| OS03T0750700-01 |  | OS03g0750700 | | Os03g0750700 | |  | | - | |
| OS03T0750800-01 |  | OS03g0750800 | | Os03g0750800 | |  | | - | |
| OS03T0751100-02 |  | OS03g0751100 | | Os03g0751100 | |  | | - | |
| OS03T0751400-01 | Down | OS03g0751400 | | Os03g0751400 | | Down | | Down-Down | |
| OS03T0751600-02 |  | OS03g0751600 | | Os03g0751600 | |  | | - | |
| OS03T0752100-01 |  | OS03g0752100 | | Os03g0752100 | |  | | - | |
| OS03T0752500-01 |  | OS03g0752500 | | Os03g0752500 | |  | | - | |
| OS03T0754900-01 |  | OS03g0754900 | | Os03g0754900 | |  | | - | |
| OS03T0755100-01 |  | OS03g0755100 | | Os03g0755100 | |  | | - | |
| OS03T0756000-01 | Down | OS03g0756000 | | Os03g0756000 | |  | | Down- | |
| OS03T0757200-01 |  | OS03g0757200 | | Os03g0757200 | |  | | - | |
| OS03T0757900-01 |  | OS03g0757900 | | Os03g0757900 | |  | | - | |
| OS03T0758100-01 | Up | OS03g0758100 | | Os03g0758100 | | Up | | Up-Up | |
| OS03T0758400-01 |  | OS03g0758400 | | Os03g0758400 | |  | | - | |
| OS03T0758800-01 |  | OS03g0758800 | | Os03g0758800 | |  | | - | |
| OS03T0758900-01 | Down | OS03g0758900 | | Os03g0758900 | | Down | | Down-Down | |
| OS03T0760000-01 |  | OS03g0760000 | | Os03g0760000 | | Up | | -Up | |
| OS03T0760600-01 |  | OS03g0760600 | | Os03g0760600 | |  | | - | |
| OS03T0760700-01 |  | OS03g0760700 | | Os03g0760700 | |  | | - | |
| OS03T0761500-02 |  | OS03g0761500 | | Os03g0761500 | |  | | - | |
| OS03T0762300-02 |  | OS03g0762300 | | Os03g0762300 | |  | | - | |
| OS03T0764450-00 |  | OS03g0764450 | | Os03g0764450 | |  | | - | |
| OS03T0764800-02 |  | OS03g0764800 | | Os03g0764800 | |  | | - | |
| OS03T0765800-00 |  | OS03g0765800 | | Os03g0765800 | |  | | - | |
| OS03T0766900-02 |  | OS03g0766900 | | Os03g0766900 | |  | | - | |
| OS03T0767500-01 |  | OS03g0767500 | | Os03g0767500 | |  | | - | |
| OS03T0769100-01 | Down | OS03g0769100 | | Os03g0769100 | |  | | Down- | |
| OS03T0772800-02 | Up | OS03g0772800 | | Os03g0772800 | |  | | Up- | |
| OS03T0773800-03 |  | OS03g0773800 | | Os03g0773800 | |  | | - | |
| OS03T0774200-02 |  | OS03g0774200 | | Os03g0774200 | |  | | - | |
| OS03T0774300-02 |  | OS03g0774300 | | Os03g0774300 | |  | | - | |
| OS03T0774400-01 |  | OS03g0774400 | | Os03g0774400 | | Down | | -Down | |
| OS03T0775500-01 |  | OS03g0775500 | | Os03g0775500 | |  | | - | |
| OS03T0776000-01 |  | OS03g0776000 | | Os03g0776000 | |  | | - | |
| OS03T0777600-01 |  | OS03g0777600 | | Os03g0777600 | |  | | - | |
| OS03T0778100-03 |  | OS03g0778100 | | Os03g0778100 | |  | | - | |
| OS03T0780500-01 |  | OS03g0780500 | | Os03g0780500 | |  | | - | |
| OS03T0780500-02 |  | OS03g0780500 | | Os03g0780500 | |  | | - | |
| OS03T0780600-01 |  | OS03g0780600 | | Os03g0780600 | |  | | - | |
| OS03T0783000-01 |  | OS03g0783000 | | Os03g0783000 | |  | | - | |
| OS03T0784000-02 |  | OS03g0784000 | | Os03g0784000 | |  | | - | |
| OS03T0784400-01 |  | OS03g0784400 | | Os03g0784400 | |  | | - | |
| OS03T0784700-02 | Up | OS03g0784700 | | Os03g0784700 | |  | | Up- | |
| OS03T0784900-02 |  | OS03g0784900 | | Os03g0784900 | |  | | - | |
| OS03T0785200-01 |  | OS03g0785200 | | Os03g0785200 | |  | | - | |
| OS03T0785300-01 |  | OS03g0785300 | | Os03g0785300 | |  | | - | |
| OS03T0786100-02 |  | OS03g0786100 | | Os03g0786100 | |  | | - | |
| OS03T0786700-01 |  | OS03g0786700 | | Os03g0786700 | |  | | - | |
| OS03T0786800-01 |  | OS03g0786800 | | Os03g0786800 | |  | | - | |
| OS03T0787000-01 |  | OS03g0787000 | | Os03g0787000 | |  | | - | |
| OS03T0787100-01 |  | OS03g0787100 | | Os03g0787100 | |  | | - | |
| OS03T0787300-01 |  | OS03g0787300 | | Os03g0787300 | |  | | - | |
| OS03T0788200-01 |  | OS03g0788200 | | Os03g0788200 | |  | | - | |
| OS03T0788250-00 |  | OS03g0788250 | | Os03g0788250 | |  | | - | |
| OS03T0791500-01 |  | OS03g0791500 | | Os03g0791500 | |  | | - | |
| OS03T0792400-01 |  | OS03g0792400 | | Os03g0792400 | |  | | - | |
| OS03T0793800-01 |  | OS03g0793800 | | Os03g0793800 | |  | | - | |
| OS03T0794500-01 |  | OS03g0794500 | | Os03g0794500 | |  | | - | |
| OS03T0795500-01 |  | OS03g0795500 | | Os03g0795500 | |  | | - | |
| OS03T0796501-00 | Down | OS03g0796501 | | Os03g0796501 | |  | | Down- | |
| OS03T0797600-01 |  | OS03g0797600 | | Os03g0797600 | |  | | - | |
| OS03T0798600-01 |  | OS03g0798600 | | Os03g0798600 | |  | | - | |
| OS03T0799000-01 |  | OS03g0799000 | | Os03g0799000 | |  | | - | |
| OS03T0799600-02 |  | OS03g0799600 | | Os03g0799600 | |  | | - | |
| OS03T0799700-01 |  | OS03g0799700 | | Os03g0799700 | |  | | - | |
| OS03T0800000-01 |  | OS03g0800000 | | Os03g0800000 | |  | | - | |
| OS03T0801600-02 |  | OS03g0801600 | | Os03g0801600 | |  | | - | |
| OS03T0801700-01 |  | OS03g0801700 | | Os03g0801700 | |  | | - | |
| OS03T0802300-03 |  | OS03g0802300 | | Os03g0802300 | |  | | - | |
| OS03T0804400-01 |  | OS03g0804400 | | Os03g0804400 | |  | | - | |
| OS03T0804500-01 | Up | OS03g0804500 | | Os03g0804500 | | Down | | Up-Down | |
| OS03T0804800-01 |  | OS03g0804800 | | Os03g0804800 | |  | | - | |
| OS03T0805200-01 |  | OS03g0805200 | | Os03g0805200 | |  | | - | |
| OS03T0805300-01 |  | OS03g0805300 | | Os03g0805300 | |  | | - | |
| OS03T0805600-00 |  | OS03g0805600 | | Os03g0805600 | |  | | - | |
| OS03T0806900-01 |  | OS03g0806900 | | Os03g0806900 | |  | | - | |
| OS03T0807800-01 |  | OS03g0807800 | | Os03g0807800 | |  | | - | |
| OS03T0807900-02 |  | OS03g0807900 | | Os03g0807900 | | Up | | -Up | |
| OS03T0808300-01 |  | OS03g0808300 | | Os03g0808300 | |  | | - | |
| OS03T0808600-01 |  | OS03g0808600 | | Os03g0808600 | |  | | - | |
| OS03T0809300-01 |  | OS03g0809300 | | Os03g0809300 | |  | | - | |
| OS03T0810600-01 |  | OS03g0810600 | | Os03g0810600 | |  | | - | |
| OS03T0811100-01 | Down | OS03g0811100 | | Os03g0811100 | | Down | | Down-Down | |
| OS03T0811600-01 |  | OS03g0811600 | | Os03g0811600 | |  | | - | |
| OS03T0812000-01 |  | OS03g0812000 | | Os03g0812000 | |  | | - | |
| OS03T0815200-01 | Up | OS03g0815200 | | Os03g0815200 | |  | | Up- | |
| OS03T0815400-01 |  | OS03g0815400 | | Os03g0815400 | | Down | | -Down | |
| OS03T0815900-01 |  | OS03g0815900 | | Os03g0815900 | |  | | - | |
| OS03T0818100-01 |  | OS03g0818100 | | Os03g0818100 | |  | | - | |
| OS03T0818200-01 |  | OS03g0818200 | | Os03g0818200 | |  | | - | |
| OS03T0818400-02 |  | OS03g0818400 | | Os03g0818400 | |  | | - | |
| OS03T0819100-01 |  | OS03g0819100 | | Os03g0819100 | |  | | - | |
| OS03T0819600-02 |  | OS03g0819600 | | Os03g0819600 | |  | | - | |
| OS03T0819900-01 |  | OS03g0819900 | | Os03g0819900 | |  | | - | |
| OS03T0820100-01 |  | OS03g0820100 | | Os03g0820100 | |  | | - | |
| OS03T0820500-01 |  | OS03g0820500 | | Os03g0820500 | | Up | | -Up | |
| OS03T0820600-01 |  | OS03g0820600 | | Os03g0820600 | |  | | - | |
| OS03T0821100-01 |  | OS03g0821100 | | Os03g0821100 | |  | | - | |
| OS03T0822200-01 |  | OS03g0822200 | | Os03g0822200 | |  | | - | |
| OS03T0822700-01 |  | OS03g0822700 | | Os03g0822700 | |  | | - | |
| OS03T0823100-01 |  | OS03g0823100 | | Os03g0823100 | |  | | - | |
| OS03T0823400-01 |  | OS03g0823400 | | Os03g0823400 | | Down | | -Down | |
| OS03T0824100-01 |  | OS03g0824100 | | Os03g0824100 | |  | | - | |
| OS03T0825300-01 |  | OS03g0825300 | | Os03g0825300 | |  | | - | |
| OS03T0825400-01 |  | OS03g0825400 | | Os03g0825400 | |  | | - | |
| OS03T0825500-01 |  | OS03g0825500 | | Os03g0825500 | |  | | - | |
| OS03T0825600-01 |  | OS03g0825600 | | Os03g0825600 | |  | | - | |
| OS03T0826400-01 |  | OS03g0826400 | | Os03g0826400 | |  | | - | |
| OS03T0826600-02 |  | OS03g0826600 | | Os03g0826600 | | Up | | -Up | |
| OS03T0826800-01 |  | OS03g0826800 | | Os03g0826800 | |  | | - | |
| OS03T0827700-01 |  | OS03g0827700 | | Os03g0827700 | |  | | - | |
| OS03T0828100-02 | Down | OS03g0828100 | | Os03g0828100 | | Down | | Down-Down | |
| OS03T0829000-03 |  | OS03g0829000 | | Os03g0829000 | |  | | - | |
| OS03T0831100-01 |  | OS03g0831100 | | Os03g0831100 | |  | | - | |
| OS03T0831500-01 |  | OS03g0831500 | | Os03g0831500 | |  | | - | |
| OS03T0831800-01 |  | OS03g0831800 | | Os03g0831800 | |  | | - | |
| OS03T0831900-01 |  | OS03g0831900 | | Os03g0831900 | |  | | - | |
| OS03T0832600-01 | Up | OS03g0832600 | | Os03g0832600 | |  | | Up- | |
| OS03T0833800-01 |  | OS03g0833800 | | Os03g0833800 | |  | | - | |
| OS03T0835100-00 | Down | OS03g0835100 | | Os03g0835100 | |  | | Down- | |
| OS03T0835400-01 |  | OS03g0835400 | | Os03g0835400 | |  | | - | |
| OS03T0835600-01 |  | OS03g0835600 | | Os03g0835600 | |  | | - | |
| OS03T0836000-01 |  | OS03g0836000 | | Os03g0836000 | |  | | - | |
| OS03T0836200-03 |  | OS03g0836200 | | Os03g0836200 | |  | | - | |
| OS03T0836300-01 |  | OS03g0836300 | | Os03g0836300 | |  | | - | |
| OS03T0837300-02 |  | OS03g0837300 | | Os03g0837300 | |  | | - | |
| OS03T0837900-01 |  | OS03g0837900 | | Os03g0837900 | |  | | - | |
| OS03T0838100-04 |  | OS03g0838100 | | Os03g0838100 | |  | | - | |
| OS03T0838400-01 |  | OS03g0838400 | | Os03g0838400 | |  | | - | |
| OS03T0838900-01 |  | OS03g0838900 | | Os03g0838900 | |  | | - | |
| OS03T0839100-01 |  | OS03g0839100 | | Os03g0839100 | |  | | - | |
| OS03T0840200-02 |  | OS03g0840200 | | Os03g0840200 | |  | | - | |
| OS03T0840900-01 |  | OS03g0840900 | | Os03g0840900 | |  | | - | |
| OS03T0841700-02 | Up | OS03g0841700 | | Os03g0841700 | |  | | Up- | |
| OS03T0841900-00 |  | OS03g0841900 | | Os03g0841900 | |  | | - | |
| OS03T0843100-02 |  | OS03g0843100 | | Os03g0843100 | |  | | - | |
| OS03T0843300-02 |  | OS03g0843300 | | Os03g0843300 | |  | | - | |
| OS03T0843400-01 | Down | OS03g0843400 | | Os03g0843400 | | Down | | Down-Down | |
| OS03T0843500-02 |  | OS03g0843500 | | Os03g0843500 | |  | | - | |
| OS03T0844100-01 |  | OS03g0844100 | | Os03g0844100 | |  | | - | |
| OS03T0844700-01 |  | OS03g0844700 | | Os03g0844700 | |  | | - | |
| OS03T0844900-01 | Down | OS03g0844900 | | Os03g0844900 | | Down | | Down-Down | |
| OS03T0845000-01 |  | OS03g0845000 | | Os03g0845000 | |  | | - | |
| OS03T0845500-01 |  | OS03g0845500 | | Os03g0845500 | |  | | - | |
| OS03T0849600-01 |  | OS03g0849600 | | Os03g0849600 | |  | | - | |
| OS03T0850000-01 |  | OS03g0850000 | | Os03g0850000 | |  | | - | |
| OS03T0850600-01 |  | OS03g0850600 | | Os03g0850600 | |  | | - | |
| OS03T0850800-01 |  | OS03g0850800 | | Os03g0850800 | |  | | - | |
| OS03T0850900-01 |  | OS03g0850900 | | Os03g0850900 | |  | | - | |
| OS03T0851100-01 |  | OS03g0851100 | | Os03g0851100 | |  | | - | |
| OS03T0851200-01 |  | OS03g0851200 | | Os03g0851200 | |  | | - | |
| OS03T0851300-03 | Up | OS03g0851300 | | Os03g0851300 | |  | | Up- | |
| OS03T0851700-01 |  | OS03g0851700 | | Os03g0851700 | |  | | - | |
| OS03T0852500-01 |  | OS03g0852500 | | Os03g0852500 | |  | | - | |
| OS03T0852900-01 |  | OS03g0852900 | | Os03g0852900 | |  | | - | |
| OS03T0853700-01 |  | OS03g0853700 | | Os03g0853700 | |  | | - | |
| OS03T0854500-02 |  | OS03g0854500 | | Os03g0854500 | |  | | - | |
| OS03T0855600-02 |  | OS03g0855600 | | Os03g0855600 | |  | | - | |
| OS03T0856100-01 |  | OS03g0856100 | | Os03g0856100 | |  | | - | |
| OS03T0856400-02 |  | OS03g0856400 | | Os03g0856400 | |  | | - | |
| OS03T0856500-02 | Down | OS03g0856500 | | Os03g0856500 | | Down | | Down-Down | |
| OS03T0857400-01 | Down | OS03g0857400 | | Os03g0857400 | |  | | Down- | |
| OS03T0858100-01 |  | OS03g0858100 | | Os03g0858100 | |  | | - | |
| OS03T0858400-01 |  | OS03g0858400 | | Os03g0858400 | |  | | - | |
| OS03T0858700-01 |  | OS03g0858700 | | Os03g0858700 | |  | | - | |
| OS03T0859600-01 |  | OS03g0859600 | | Os03g0859600 | |  | | - | |
| OS03T0860600-01 |  | OS03g0860600 | | Os03g0860600 | |  | | - | |
| OS03T0860900-01 |  | OS03g0860900 | | Os03g0860900 | |  | | - | |
| OS03T0862100-01 |  | OS03g0862100 | | Os03g0862100 | |  | | - | |
| OS04T0100300-00 |  | OS04g0100300 | | Os04g0100300 | |  | | - | |
| OS04T0102500-01 |  | OS04g0102500 | | Os04g0102500 | |  | | - | |
| OS04T0102700-01 |  | OS04g0102700 | | Os04g0102700 | |  | | - | |
| OS04T0103200-02 |  | OS04g0103200 | | Os04g0103200 | |  | | - | |
| OS04T0105700-01 | Up | OS04g0105700 | | Os04g0105700 | |  | | Up- | |
| OS04T0106000-01 |  | OS04g0106000 | | Os04g0106000 | |  | | - | |
| OS04T0106300-01 | Up | OS04g0106300 | | Os04g0106300 | |  | | Up- | |
| OS04T0110600-01 |  | OS04g0110600 | | Os04g0110600 | | Up | | -Up | |
| OS04T0111200-01 |  | OS04g0111200 | | Os04g0111200 | |  | | - | |
| OS04T0118100-01 | Up | OS04g0118100 | | Os04g0118100 | |  | | Up- | |
| OS04T0118400-01 |  | OS04g0118400 | | Os04g0118400 | |  | | - | |
| OS04T0118900-01 |  | OS04g0118900 | | Os04g0118900 | |  | | - | |
| OS04T0119400-01 |  | OS04g0119400 | | Os04g0119400 | |  | | - | |
| OS04T0121100-01 |  | OS04g0121100 | | Os04g0121100 | | Down | | -Down | |
| OS04T0126600-00 |  | OS04g0126600 | | Os04g0126600 | |  | | - | |
| OS04T0127600-00 |  | OS04g0127600 | | Os04g0127600 | |  | | - | |
| OS04T0128900-01 |  | OS04g0128900 | | Os04g0128900 | |  | | - | |
| OS04T0129500-02 |  | OS04g0129500 | | Os04g0129500 | |  | | - | |
| OS04T0129600-01 |  | OS04g0129600 | | Os04g0129600 | |  | | - | |
| OS04T0131900-01 |  | OS04g0131900 | | Os04g0131900 | |  | | - | |
| OS04T0136700-01 |  | OS04g0136700 | | Os04g0136700 | | Up | | -Up | |
| OS04T0137500-00 | Up | OS04g0137500 | | Os04g0137500 | |  | | Up- | |
| OS04T0151800-02 |  | OS04g0151800 | | Os04g0151800 | |  | | - | |
| OS04T0156200-00 |  | OS04g0156200 | | Os04g0156200 | |  | | - | |
| OS04T0164900-01 |  | OS04g0164900 | | Os04g0164900 | |  | | - | |
| OS04T0165400-01 |  | OS04g0165400 | | Os04g0165400 | |  | | - | |
| OS04T0165500-01 |  | OS04g0165500 | | Os04g0165500 | |  | | - | |
| OS04T0165700-01 |  | OS04g0165700 | | Os04g0165700 | |  | | - | |
| OS04T0167800-01 | Up | OS04g0167800 | | Os04g0167800 | |  | | Up- | |
| OS04T0174800-01 |  | OS04g0174800 | | Os04g0174800 | |  | | - | |
| OS04T0175600-01 |  | OS04g0175600 | | Os04g0175600 | |  | | - | |
| OS04T0177600-02 |  | OS04g0177600 | | Os04g0177600 | |  | | - | |
| OS04T0181100-01 |  | OS04g0181100 | | Os04g0181100 | |  | | - | |
| OS04T0182800-01 |  | OS04g0182800 | | Os04g0182800 | |  | | - | |
| OS04T0183500-01 |  | OS04g0183500 | | Os04g0183500 | |  | | - | |
| OS04T0184100-01 |  | OS04g0184100 | | Os04g0184100 | |  | | - | |
| OS04T0186400-02 | Up | OS04g0186400 | | Os04g0186400 | | Up | | Up-Up | |
| OS04T0206500-01 |  | OS04g0206500 | | Os04g0206500 | |  | | - | |
| OS04T0208200-01 |  | OS04g0208200 | | Os04g0208200 | |  | | - | |
| OS04T0209300-01 |  | OS04g0209300 | | Os04g0209300 | |  | | - | |
| OS04T0218600-02 |  | OS04g0218600 | | Os04g0218600 | |  | | - | |
| OS04T0223300-00 |  | OS04g0223300 | | Os04g0223300 | |  | | - | |
| OS04T0224600-01 |  | OS04g0224600 | | Os04g0224600 | |  | | - | |
| OS04T0224900-01 |  | OS04g0224900 | | Os04g0224900 | |  | | - | |
| OS04T0227500-01 | Up | OS04g0227500 | | Os04g0227500 | |  | | Up- | |
| OS04T0233400-02 |  | OS04g0233400 | | Os04g0233400 | |  | | - | |
| OS04T0234600-03 | Down | OS04g0234600 | | Os04g0234600 | |  | | Down- | |
| OS04T0244400-02 | Up | OS04g0244400 | | Os04g0244400 | |  | | Up- | |
| OS04T0244800-01 |  | OS04g0244800 | | Os04g0244800 | |  | | - | |
| OS04T0250700-01 |  | OS04g0250700 | | Os04g0250700 | |  | | - | |
| OS04T0253000-01 |  | OS04g0253000 | | Os04g0253000 | |  | | - | |
| OS04T0254000-01 |  | OS04g0254000 | | Os04g0254000 | |  | | - | |
| OS04T0266900-01 |  | OS04g0266900 | | Os04g0266900 | | Up | | -Up | |
| OS04T0269600-02 | Up | OS04g0269600 | | Os04g0269600 | |  | | Up- | |
| OS04T0269600-03 |  | OS04g0269600 | | Os04g0269600 | |  | | - | |
| OS04T0269600-04 |  | OS04g0269600 | | Os04g0269600 | |  | | - | |
| OS04T0270200-01 |  | OS04g0270200 | | Os04g0270200 | |  | | - | |
| OS04T0281900-01 |  | OS04g0281900 | | Os04g0281900 | |  | | - | |
| OS04T0282400-01 |  | OS04g0282400 | | Os04g0282400 | |  | | - | |
| OS04T0290800-00 |  | OS04g0290800 | | Os04g0290800 | |  | | - | |
| OS04T0303900-01 |  | OS04g0303900 | | Os04g0303900 | |  | | - | |
| OS04T0304200-01 |  | OS04g0304200 | | Os04g0304200 | |  | | - | |
| OS04T0306400-01 |  | OS04g0306400 | | Os04g0306400 | |  | | - | |
| OS04T0306750-01 | Down | OS04g0306750 | | Os04g0306750 | |  | | Down- | |
| OS04T0306800-02 |  | OS04g0306800 | | Os04g0306800 | |  | | - | |
| OS04T0307200-01 |  | OS04g0307200 | | Os04g0307200 | |  | | - | |
| OS04T0309600-01 |  | OS04g0309600 | | Os04g0309600 | |  | | - | |
| OS04T0310500-02 |  | OS04g0310500 | | Os04g0310500 | |  | | - | |
| OS04T0319600-01 |  | OS04g0319600 | | Os04g0319600 | |  | | - | |
| OS04T0320100-01 |  | OS04g0320100 | | Os04g0320100 | |  | | - | |
| OS04T0320200-01 |  | OS04g0320200 | | Os04g0320200 | |  | | - | |
| OS04T0320700-01 |  | OS04g0320700 | | Os04g0320700 | | Up | | -Up | |
| OS04T0321600-01 |  | OS04g0321600 | | Os04g0321600 | |  | | - | |
| OS04T0321700-02 | Up | OS04g0321700 | | Os04g0321700 | |  | | Up- | |
| OS04T0322100-01 |  | OS04g0322100 | | Os04g0322100 | |  | | - | |
| OS04T0337201-00 |  | OS04g0337201 | | Os04g0337201 | |  | | - | |
| OS04T0337500-02 |  | OS04g0337500 | | Os04g0337500 | |  | | - | |
| OS04T0346100-01 |  | OS04g0346100 | | Os04g0346100 | |  | | - | |
| OS04T0348300-01 |  | OS04g0348300 | | Os04g0348300 | |  | | - | |
| OS04T0369000-01 |  | OS04g0369000 | | Os04g0369000 | |  | | - | |
| OS04T0372700-01 | Up | OS04g0372700 | | Os04g0372700 | |  | | Up- | |
| OS04T0372800-01 |  | OS04g0372800 | | Os04g0372800 | |  | | - | |
| OS04T0376000-01 |  | OS04g0376000 | | Os04g0376000 | |  | | - | |
| OS04T0376500-01 |  | OS04g0376500 | | Os04g0376500 | |  | | - | |
| OS04T0377932-00 |  | OS04g0377932 | | Os04g0377932 | |  | | - | |
| OS04T0378200-01 |  | OS04g0378200 | | Os04g0378200 | |  | | - | |
| OS04T0379300-02 |  | OS04g0379300 | | Os04g0379300 | |  | | - | |
| OS04T0379700-01 |  | OS04g0379700 | | Os04g0379700 | |  | | - | |
| OS04T0379900-01 |  | OS04g0379900 | | Os04g0379900 | |  | | - | |
| OS04T0381600-00 |  | OS04g0381600 | | Os04g0381600 | |  | | - | |
| OS04T0382100-01 |  | OS04g0382100 | | Os04g0382100 | |  | | - | |
| OS04T0382200-01 |  | OS04g0382200 | | Os04g0382200 | |  | | - | |
| OS04T0382300-03 |  | OS04g0382300 | | Os04g0382300 | |  | | - | |
| OS04T0385700-01 |  | OS04g0385700 | | Os04g0385700 | |  | | - | |
| OS04T0386500-01 |  | OS04g0386500 | | Os04g0386500 | |  | | - | |
| OS04T0386600-02 |  | OS04g0386600 | | Os04g0386600 | |  | | - | |
| OS04T0388500-01 |  | OS04g0388500 | | Os04g0388500 | |  | | - | |
| OS04T0388800-01 |  | OS04g0388800 | | Os04g0388800 | |  | | - | |
| OS04T0389800-01 |  | OS04g0389800 | | Os04g0389800 | |  | | - | |
| OS04T0390000-02 |  | OS04g0390000 | | Os04g0390000 | |  | | - | |
| OS04T0391900-01 |  | OS04g0391900 | | Os04g0391900 | |  | | - | |
| OS04T0394200-03 |  | OS04g0394200 | | Os04g0394200 | |  | | - | |
| OS04T0394300-01 |  | OS04g0394300 | | Os04g0394300 | |  | | - | |
| OS04T0397100-01 |  | OS04g0397100 | | Os04g0397100 | |  | | - | |
| OS04T0398300-01 |  | OS04g0398300 | | Os04g0398300 | |  | | - | |
| OS04T0398500-01 |  | OS04g0398500 | | Os04g0398500 | |  | | - | |
| OS04T0402100-01 |  | OS04g0402100 | | Os04g0402100 | |  | | - | |
| OS04T0403500-02 |  | OS04g0403500 | | Os04g0403500 | |  | | - | |
| OS04T0404800-01 |  | OS04g0404800 | | Os04g0404800 | | Up | | -Up | |
| OS04T0405100-01 |  | OS04g0405100 | | Os04g0405100 | |  | | - | |
| OS04T0405600-02 |  | OS04g0405600 | | Os04g0405600 | |  | | - | |
| OS04T0409200-00 |  | OS04g0409200 | | Os04g0409200 | | Up | | -Up | |
| OS04T0409500-01 |  | OS04g0409500 | | Os04g0409500 | |  | | - | |
| OS04T0412200-02 |  | OS04g0412200 | | Os04g0412200 | | Down | | -Down | |
| OS04T0412500-01 |  | OS04g0412500 | | Os04g0412500 | |  | | - | |
| OS04T0414700-01 |  | OS04g0414700 | | Os04g0414700 | | Down | | -Down | |
| OS04T0415800-00 |  | OS04g0415800 | | Os04g0415800 | |  | | - | |
| OS04T0416000-01 |  | OS04g0416000 | | Os04g0416000 | |  | | - | |
| OS04T0416700-01 |  | OS04g0416700 | | Os04g0416700 | | Down | | -Down | |
| OS04T0418000-01 | Up | OS04g0418000 | | Os04g0418000 | |  | | Up- | |
| OS04T0421800-01 |  | OS04g0421800 | | Os04g0421800 | |  | | - | |
| OS04T0421900-01 |  | OS04g0421900 | | Os04g0421900 | |  | | - | |
| OS04T0422000-03 |  | OS04g0422000 | | Os04g0422000 | |  | | - | |
| OS04T0423600-03 |  | OS04g0423600 | | Os04g0423600 | |  | | - | |
| OS04T0428900-01 |  | OS04g0428900 | | Os04g0428900 | |  | | - | |
| OS04T0429800-01 |  | OS04g0429800 | | Os04g0429800 | |  | | - | |
| OS04T0430100-01 |  | OS04g0430100 | | Os04g0430100 | |  | | - | |
| OS04T0430700-01 |  | OS04g0430700 | | Os04g0430700 | |  | | - | |
| OS04T0430800-02 |  | OS04g0430800 | | Os04g0430800 | |  | | - | |
| OS04T0431100-01 |  | OS04g0431100 | | Os04g0431100 | |  | | - | |
| OS04T0431300-01 |  | OS04g0431300 | | Os04g0431300 | |  | | - | |
| OS04T0432400-01 |  | OS04g0432400 | | Os04g0432400 | |  | | - | |
| OS04T0432500-01 |  | OS04g0432500 | | Os04g0432500 | |  | | - | |
| OS04T0434800-02 | Up | OS04g0434800 | | Os04g0434800 | | Up | | Up-Up | |
| OS04T0434900-00 |  | OS04g0434900 | | Os04g0434900 | | Down | | -Down | |
| OS04T0435700-01 |  | OS04g0435700 | | Os04g0435700 | |  | | - | |
| OS04T0436100-01 |  | OS04g0436100 | | Os04g0436100 | |  | | - | |
| OS04T0437600-01 |  | OS04g0437600 | | Os04g0437600 | |  | | - | |
| OS04T0438300-01 |  | OS04g0438300 | | Os04g0438300 | | Down | | -Down | |
| OS04T0439900-03 |  | OS04g0439900 | | Os04g0439900 | |  | | - | |
| OS04T0440100-02 |  | OS04g0440100 | | Os04g0440100 | |  | | - | |
| OS04T0441900-02 |  | OS04g0441900 | | Os04g0441900 | |  | | - | |
| OS04T0442200-01 |  | OS04g0442200 | | Os04g0442200 | |  | | - | |
| OS04T0443200-01 |  | OS04g0443200 | | Os04g0443200 | | Up | | -Up | |
| OS04T0443500-01 |  | OS04g0443500 | | Os04g0443500 | |  | | - | |
| OS04T0443900-01 |  | OS04g0443900 | | Os04g0443900 | |  | | - | |
| OS04T0444200-01 |  | OS04g0444200 | | Os04g0444200 | |  | | - | |
| OS04T0444600-01 |  | OS04g0444600 | | Os04g0444600 | |  | | - | |
| OS04T0444800-02 |  | OS04g0444800 | | Os04g0444800 | | Up | | -Up | |
| OS04T0445200-01 |  | OS04g0445200 | | Os04g0445200 | |  | | - | |
| OS04T0445700-02 |  | OS04g0445700 | | Os04g0445700 | | Down | | -Down | |
| OS04T0446200-01 |  | OS04g0446200 | | Os04g0446200 | | Down | | -Down | |
| OS04T0446300-01 |  | OS04g0446300 | | Os04g0446300 | |  | | - | |
| OS04T0446500-01 |  | OS04g0446500 | | Os04g0446500 | |  | | - | |
| OS04T0447100-01 |  | OS04g0447100 | | Os04g0447100 | |  | | - | |
| OS04T0447500-01 |  | OS04g0447500 | | Os04g0447500 | |  | | - | |
| OS04T0447700-01 |  | OS04g0447700 | | Os04g0447700 | | Up | | -Up | |
| OS04T0447800-01 |  | OS04g0447800 | | Os04g0447800 | |  | | - | |
| OS04T0448600-02 |  | OS04g0448600 | | Os04g0448600 | |  | | - | |
| OS04T0448900-00 | Down | OS04g0448900 | | Os04g0448900 | |  | | Down- | |
| OS04T0449900-01 |  | OS04g0449900 | | Os04g0449900 | |  | | - | |
| OS04T0450100-02 |  | OS04g0450100 | | Os04g0450100 | | Up | | -Up | |
| OS04T0452000-01 | Up | OS04g0452000 | | Os04g0452000 | |  | | Up- | |
| OS04T0455800-01 |  | OS04g0455800 | | Os04g0455800 | |  | | - | |
| OS04T0456700-01 | Up | OS04g0456700 | | Os04g0456700 | | Up | | Up-Up | |
| OS04T0457000-01 |  | OS04g0457000 | | Os04g0457000 | |  | | - | |
| OS04T0457500-01 |  | OS04g0457500 | | Os04g0457500 | |  | | - | |
| OS04T0458300-02 | Up | OS04g0458300 | | Os04g0458300 | | Down | | Up-Down | |
| OS04T0459500-02 | Down | OS04g0459500 | | Os04g0459500 | |  | | Down- | |
| OS04T0459900-01 |  | OS04g0459900 | | Os04g0459900 | |  | | - | |
| OS04T0461100-02 |  | OS04g0461100 | | Os04g0461100 | | Down | | -Down | |
| OS04T0462500-05 |  | OS04g0462500 | | Os04g0462500 | |  | | - | |
| OS04T0462900-06 | Up | OS04g0462900 | | Os04g0462900 | |  | | Up- | |
| OS04T0464200-01 |  | OS04g0464200 | | Os04g0464200 | |  | | - | |
| OS04T0464500-01 |  | OS04g0464500 | | Os04g0464500 | |  | | - | |
| OS04T0465500-02 |  | OS04g0465500 | | Os04g0465500 | |  | | - | |
| OS04T0466600-01 |  | OS04g0466600 | | Os04g0466600 | |  | | - | |
| OS04T0469500-01 |  | OS04g0469500 | | Os04g0469500 | |  | | - | |
| OS04T0470100-01 |  | OS04g0470100 | | Os04g0470100 | |  | | - | |
| OS04T0470700-01 |  | OS04g0470700 | | Os04g0470700 | |  | | - | |
| OS04T0472300-01 |  | OS04g0472300 | | Os04g0472300 | |  | | - | |
| OS04T0473150-00 |  | OS04g0473150 | | Os04g0473150 | |  | | - | |
| OS04T0473400-01 |  | OS04g0473400 | | Os04g0473400 | |  | | - | |
| OS04T0474800-01 |  | OS04g0474800 | | Os04g0474800 | |  | | - | |
| OS04T0474800-03 | Up | OS04g0474800 | | Os04g0474800 | |  | | Up- | |
| OS04T0475600-01 |  | OS04g0475600 | | Os04g0475600 | | Up | | -Up | |
| OS04T0476700-01 |  | OS04g0476700 | | Os04g0476700 | |  | | - | |
| OS04T0479100-02 |  | OS04g0479100 | | Os04g0479100 | |  | | - | |
| OS04T0479200-01 |  | OS04g0479200 | | Os04g0479200 | |  | | - | |
| OS04T0480100-01 |  | OS04g0480100 | | Os04g0480100 | |  | | - | |
| OS04T0480400-01 |  | OS04g0480400 | | Os04g0480400 | |  | | - | |
| OS04T0481300-01 |  | OS04g0481300 | | Os04g0481300 | |  | | - | |
| OS04T0481400-01 |  | OS04g0481400 | | Os04g0481400 | |  | | - | |
| OS04T0482000-01 |  | OS04g0482000 | | Os04g0482000 | |  | | - | |
| OS04T0482800-01 |  | OS04g0482800 | | Os04g0482800 | |  | | - | |
| OS04T0484900-01 |  | OS04g0484900 | | Os04g0484900 | |  | | - | |
| OS04T0485000-01 |  | OS04g0485000 | | Os04g0485000 | |  | | - | |
| OS04T0485200-01 |  | OS04g0485200 | | Os04g0485200 | |  | | - | |
| OS04T0485300-02 |  | OS04g0485300 | | Os04g0485300 | |  | | - | |
| OS04T0485400-01 |  | OS04g0485400 | | Os04g0485400 | |  | | - | |
| OS04T0486600-01 |  | OS04g0486600 | | Os04g0486600 | |  | | - | |
| OS04T0486950-01 | Up | OS04g0486950 | | Os04g0486950 | | Up | | Up-Up | |
| OS04T0487200-01 |  | OS04g0487200 | | Os04g0487200 | |  | | - | |
| OS04T0490400-01 |  | OS04g0490400 | | Os04g0490400 | |  | | - | |
| OS04T0490500-01 |  | OS04g0490500 | | Os04g0490500 | |  | | - | |
| OS04T0490800-01 |  | OS04g0490800 | | Os04g0490800 | |  | | - | |
| OS04T0492100-01 |  | OS04g0492100 | | Os04g0492100 | |  | | - | |
| OS04T0492300-01 |  | OS04g0492300 | | Os04g0492300 | |  | | - | |
| OS04T0493400-01 |  | OS04g0493400 | | Os04g0493400 | | Down | | -Down | |
| OS04T0496400-01 |  | OS04g0496400 | | Os04g0496400 | |  | | - | |
| OS04T0496800-01 |  | OS04g0496800 | | Os04g0496800 | |  | | - | |
| OS04T0497000-02 |  | OS04g0497000 | | Os04g0497000 | | Down | | -Down | |
| OS04T0497900-01 |  | OS04g0497900 | | Os04g0497900 | |  | | - | |
| OS04T0498200-01 |  | OS04g0498200 | | Os04g0498200 | |  | | - | |
| OS04T0499300-01 |  | OS04g0499300 | | Os04g0499300 | |  | | - | |
| OS04T0500700-02 |  | OS04g0500700 | | Os04g0500700 | |  | | - | |
| OS04T0501000-00 |  | OS04g0501000 | | Os04g0501000 | |  | | - | |
| OS04T0501600-01 |  | OS04g0501600 | | Os04g0501600 | |  | | - | |
| OS04T0502900-01 |  | OS04g0502900 | | Os04g0502900 | |  | | - | |
| OS04T0504000-01 |  | OS04g0504000 | | Os04g0504000 | |  | | - | |
| OS04T0504600-01 |  | OS04g0504600 | | Os04g0504600 | |  | | - | |
| OS04T0504800-01 |  | OS04g0504800 | | Os04g0504800 | |  | | - | |
| OS04T0505200-03 | Down | OS04g0505200 | | Os04g0505200 | | Down | | Down-Down | |
| OS04T0507100-02 |  | OS04g0507100 | | Os04g0507100 | |  | | - | |
| OS04T0508200-01 |  | OS04g0508200 | | Os04g0508200 | |  | | - | |
| OS04T0508300-01 |  | OS04g0508300 | | Os04g0508300 | |  | | - | |
| OS04T0509600-02 |  | OS04g0509600 | | Os04g0509600 | |  | | - | |
| OS04T0513000-01 |  | OS04g0513000 | | Os04g0513000 | |  | | - | |
| OS04T0514400-01 |  | OS04g0514400 | | Os04g0514400 | |  | | - | |
| OS04T0514800-01 |  | OS04g0514800 | | Os04g0514800 | |  | | - | |
| OS04T0515300-02 |  | OS04g0515300 | | Os04g0515300 | |  | | - | |
| OS04T0516600-01 |  | OS04g0516600 | | Os04g0516600 | |  | | - | |
| OS04T0517300-01 |  | OS04g0517300 | | Os04g0517300 | |  | | - | |
| OS04T0518000-01 |  | OS04g0518000 | | Os04g0518000 | |  | | - | |
| OS04T0521800-01 |  | OS04g0521800 | | Os04g0521800 | |  | | - | |
| OS04T0523000-00 |  | OS04g0523000 | | Os04g0523000 | |  | | - | |
| OS04T0523200-01 |  | OS04g0523200 | | Os04g0523200 | |  | | - | |
| OS04T0524400-01 |  | OS04g0524400 | | Os04g0524400 | |  | | - | |
| OS04T0525000-01 |  | OS04g0525000 | | Os04g0525000 | |  | | - | |
| OS04T0525600-02 |  | OS04g0525600 | | Os04g0525600 | |  | | - | |
| OS04T0527700-01 |  | OS04g0527700 | | Os04g0527700 | |  | | - | |
| OS04T0527800-01 |  | OS04g0527800 | | Os04g0527800 | |  | | - | |
| OS04T0528800-02 |  | OS04g0528800 | | Os04g0528800 | |  | | - | |
| OS04T0530300-01 |  | OS04g0530300 | | Os04g0530300 | |  | | - | |
| OS04T0531900-01 |  | OS04g0531900 | | Os04g0531900 | | Up | | -Up | |
| OS04T0532400-01 |  | OS04g0532400 | | Os04g0532400 | | Up | | -Up | |
| OS04T0533300-01 |  | OS04g0533300 | | Os04g0533300 | |  | | - | |
| OS04T0534400-01 |  | OS04g0534400 | | Os04g0534400 | |  | | - | |
| OS04T0535600-01 |  | OS04g0535600 | | Os04g0535600 | |  | | - | |
| OS04T0537800-01 |  | OS04g0537800 | | Os04g0537800 | |  | | - | |
| OS04T0537900-01 |  | OS04g0537900 | | Os04g0537900 | |  | | - | |
| OS04T0538000-01 |  | OS04g0538000 | | Os04g0538000 | |  | | - | |
| OS04T0538100-01 |  | OS04g0538100 | | Os04g0538100 | |  | | - | |
| OS04T0539000-01 | Down | OS04g0539000 | | Os04g0539000 | |  | | Down- | |
| OS04T0539800-01 |  | OS04g0539800 | | Os04g0539800 | |  | | - | |
| OS04T0542900-01 |  | OS04g0542900 | | Os04g0542900 | |  | | - | |
| OS04T0543200-01 |  | OS04g0543200 | | Os04g0543200 | |  | | - | |
| OS04T0543900-02 | Up | OS04g0543900 | | Os04g0543900 | |  | | Up- | |
| OS04T0544400-00 |  | OS04g0544400 | | Os04g0544400 | | Down | | -Down | |
| OS04T0545200-00 |  | OS04g0545200 | | Os04g0545200 | |  | | - | |
| OS04T0550500-05 |  | OS04g0550500 | | Os04g0550500 | |  | | - | |
| OS04T0551200-01 |  | OS04g0551200 | | Os04g0551200 | |  | | - | |
| OS04T0551700-01 |  | OS04g0551700 | | Os04g0551700 | |  | | - | |
| OS04T0551800-01 |  | OS04g0551800 | | Os04g0551800 | |  | | - | |
| OS04T0555400-01 |  | OS04g0555400 | | Os04g0555400 | |  | | - | |
| OS04T0556300-01 |  | OS04g0556300 | | Os04g0556300 | |  | | - | |
| OS04T0558400-01 |  | OS04g0558400 | | Os04g0558400 | |  | | - | |
| OS04T0559700-01 |  | OS04g0559700 | | Os04g0559700 | |  | | - | |
| OS04T0560200-01 |  | OS04g0560200 | | Os04g0560200 | |  | | - | |
| OS04T0560400-01 |  | OS04g0560400 | | Os04g0560400 | |  | | - | |
| OS04T0560500-00 |  | OS04g0560500 | | Os04g0560500 | |  | | - | |
| OS04T0560600-01 |  | OS04g0560600 | | Os04g0560600 | |  | | - | |
| OS04T0561500-02 |  | OS04g0561500 | | Os04g0561500 | | Down | | -Down | |
| OS04T0564600-01 |  | OS04g0564600 | | Os04g0564600 | |  | | - | |
| OS04T0565400-01 |  | OS04g0565400 | | Os04g0565400 | |  | | - | |
| OS04T0566000-01 |  | OS04g0566000 | | Os04g0566000 | |  | | - | |
| OS04T0566400-02 |  | OS04g0566400 | | Os04g0566400 | |  | | - | |
| OS04T0566500-04 |  | OS04g0566500 | | Os04g0566500 | |  | | - | |
| OS04T0568600-00 |  | OS04g0568600 | | Os04g0568600 | |  | | - | |
| OS04T0574200-01 |  | OS04g0574200 | | Os04g0574200 | |  | | - | |
| OS04T0577000-01 |  | OS04g0577000 | | Os04g0577000 | |  | | - | |
| OS04T0578200-01 |  | OS04g0578200 | | Os04g0578200 | | Up | | -Up | |
| OS04T0581300-01 |  | OS04g0581300 | | Os04g0581300 | |  | | - | |
| OS04T0584600-01 |  | OS04g0584600 | | Os04g0584600 | |  | | - | |
| OS04T0584800-01 |  | OS04g0584800 | | Os04g0584800 | |  | | - | |
| OS04T0589500-01 |  | OS04g0589500 | | Os04g0589500 | |  | | - | |
| OS04T0589600-01 |  | OS04g0589600 | | Os04g0589600 | |  | | - | |
| OS04T0589900-03 |  | OS04g0589900 | | Os04g0589900 | |  | | - | |
| OS04T0591000-01 | Down | OS04g0591000 | | Os04g0591000 | | Down | | Down-Down | |
| OS04T0593400-00 |  | OS04g0593400 | | Os04g0593400 | |  | | - | |
| OS04T0594400-01 |  | OS04g0594400 | | Os04g0594400 | |  | | - | |
| OS04T0596300-01 |  | OS04g0596300 | | Os04g0596300 | |  | | - | |
| OS04T0596900-00 | Down | OS04g0596900 | | Os04g0596900 | |  | | Down- | |
| OS04T0598000-01 |  | OS04g0598000 | | Os04g0598000 | |  | | - | |
| OS04T0598200-00 |  | OS04g0598200 | | Os04g0598200 | |  | | - | |
| OS04T0599900-03 |  | OS04g0599900 | | Os04g0599900 | |  | | - | |
| OS04T0600300-01 |  | OS04g0600300 | | Os04g0600300 | |  | | - | |
| OS04T0601400-01 |  | OS04g0601400 | | Os04g0601400 | |  | | - | |
| OS04T0601700-02 |  | OS04g0601700 | | Os04g0601700 | |  | | - | |
| OS04T0602100-01 | Up | OS04g0602100 | | Os04g0602100 | | Down | | Up-Down | |
| OS04T0602500-02 |  | OS04g0602500 | | Os04g0602500 | |  | | - | |
| OS04T0603000-00 |  | OS04g0603000 | | Os04g0603000 | |  | | - | |
| OS04T0605500-02 |  | OS04g0605500 | | Os04g0605500 | |  | | - | |
| OS04T0605900-01 |  | OS04g0605900 | | Os04g0605900 | |  | | - | |
| OS04T0606400-02 |  | OS04g0606400 | | Os04g0606400 | |  | | - | |
| OS04T0607000-02 |  | OS04g0607000 | | Os04g0607000 | |  | | - | |
| OS04T0608400-03 |  | OS04g0608400 | | Os04g0608400 | |  | | - | |
| OS04T0609100-01 |  | OS04g0609100 | | Os04g0609100 | |  | | - | |
| OS04T0609600-01 |  | OS04g0609600 | | Os04g0609600 | |  | | - | |
| OS04T0609700-01 |  | OS04g0609700 | | Os04g0609700 | |  | | - | |
| OS04T0610500-01 |  | OS04g0610500 | | Os04g0610500 | |  | | - | |
| OS04T0610800-01 |  | OS04g0610800 | | Os04g0610800 | | Down | | -Down | |
| OS04T0611200-00 |  | OS04g0611200 | | Os04g0611200 | |  | | - | |
| OS04T0612500-01 |  | OS04g0612500 | | Os04g0612500 | | Down | | -Down | |
| OS04T0613300-01 |  | OS04g0613300 | | Os04g0613300 | | Up | | -Up | |
| OS04T0614000-01 |  | OS04g0614000 | | Os04g0614000 | |  | | - | |
| OS04T0614600-01 |  | OS04g0614600 | | Os04g0614600 | |  | | - | |
| OS04T0614650-00 |  | OS04g0614650 | | Os04g0614650 | |  | | - | |
| OS04T0615700-01 | Down | OS04g0615700 | | Os04g0615700 | | Down | | Down-Down | |
| OS04T0616000-01 |  | OS04g0616000 | | Os04g0616000 | |  | | - | |
| OS04T0616300-01 |  | OS04g0616300 | | Os04g0616300 | |  | | - | |
| OS04T0618200-02 |  | OS04g0618200 | | Os04g0618200 | |  | | - | |
| OS04T0618500-01 |  | OS04g0618500 | | Os04g0618500 | |  | | - | |
| OS04T0619400-01 |  | OS04g0619400 | | Os04g0619400 | |  | | - | |
| OS04T0619500-01 |  | OS04g0619500 | | Os04g0619500 | |  | | - | |
| OS04T0620000-01 |  | OS04g0620000 | | Os04g0620000 | |  | | - | |
| OS04T0620700-01 |  | OS04g0620700 | | Os04g0620700 | |  | | - | |
| OS04T0623100-01 |  | OS04g0623100 | | Os04g0623100 | |  | | - | |
| OS04T0623800-01 | Down | OS04g0623800 | | Os04g0623800 | |  | | Down- | |
| OS04T0624600-01 |  | OS04g0624600 | | Os04g0624600 | | Up | | -Up | |
| OS04T0625800-01 |  | OS04g0625800 | | Os04g0625800 | |  | | - | |
| OS04T0626000-01 |  | OS04g0626000 | | Os04g0626000 | |  | | - | |
| OS04T0626100-01 |  | OS04g0626100 | | Os04g0626100 | |  | | - | |
| OS04T0626400-03 |  | OS04g0626400 | | Os04g0626400 | |  | | - | |
| OS04T0626900-01 |  | OS04g0626900 | | Os04g0626900 | |  | | - | |
| OS04T0628900-00 |  | OS04g0628900 | | Os04g0628900 | |  | | - | |
| OS04T0629500-02 | Up | OS04g0629500 | | Os04g0629500 | |  | | Up- | |
| OS04T0630400-01 |  | OS04g0630400 | | Os04g0630400 | |  | | - | |
| OS04T0634700-01 |  | OS04g0634700 | | Os04g0634700 | |  | | - | |
| OS04T0636600-02 |  | OS04g0636600 | | Os04g0636600 | |  | | - | |
| OS04T0636700-01 |  | OS04g0636700 | | Os04g0636700 | |  | | - | |
| OS04T0636900-02 |  | OS04g0636900 | | Os04g0636900 | |  | | - | |
| OS04T0637400-01 |  | OS04g0637400 | | Os04g0637400 | |  | | - | |
| OS04T0639300-01 | Up | OS04g0639300 | | Os04g0639300 | |  | | Up- | |
| OS04T0640500-01 |  | OS04g0640500 | | Os04g0640500 | |  | | - | |
| OS04T0640700-01 |  | OS04g0640700 | | Os04g0640700 | |  | | - | |
| OS04T0641400-03 |  | OS04g0641400 | | Os04g0641400 | |  | | - | |
| OS04T0643100-02 | Up | OS04g0643100 | | Os04g0643100 | |  | | Up- | |
| OS04T0643200-00 |  | OS04g0643200 | | Os04g0643200 | |  | | - | |
| OS04T0643300-01 |  | OS04g0643300 | | Os04g0643300 | |  | | - | |
| OS04T0644400-01 |  | OS04g0644400 | | Os04g0644400 | |  | | - | |
| OS04T0644600-02 | Down | OS04g0644600 | | Os04g0644600 | |  | | Down- | |
| OS04T0645100-01 |  | OS04g0645100 | | Os04g0645100 | |  | | - | |
| OS04T0647800-02 | Up | OS04g0647800 | | Os04g0647800 | |  | | Up- | |
| OS04T0650000-03 | Up | OS04g0650000 | | Os04g0650000 | |  | | Up- | |
| OS04T0650700-02 |  | OS04g0650700 | | Os04g0650700 | | Up | | -Up | |
| OS04T0650800-01 |  | OS04g0650800 | | Os04g0650800 | |  | | - | |
| OS04T0652700-01 | Up | OS04g0652700 | | Os04g0652700 | | Up | | Up-Up | |
| OS04T0653100-01 |  | OS04g0653100 | | Os04g0653100 | |  | | - | |
| OS04T0653400-01 |  | OS04g0653400 | | Os04g0653400 | |  | | - | |
| OS04T0654700-02 |  | OS04g0654700 | | Os04g0654700 | |  | | - | |
| OS04T0655600-01 | Down | OS04g0655600 | | Os04g0655600 | |  | | Down- | |
| OS04T0656100-01 |  | OS04g0656100 | | Os04g0656100 | |  | | - | |
| OS04T0657500-01 | Up | OS04g0657500 | | Os04g0657500 | |  | | Up- | |
| OS04T0658000-02 |  | OS04g0658000 | | Os04g0658000 | |  | | - | |
| OS04T0658300-01 |  | OS04g0658300 | | Os04g0658300 | |  | | - | |
| OS04T0659100-03 |  | OS04g0659100 | | Os04g0659100 | |  | | - | |
| OS04T0659400-01 |  | OS04g0659400 | | Os04g0659400 | |  | | - | |
| OS04T0659500-02 |  | OS04g0659500 | | Os04g0659500 | |  | | - | |
| OS04T0660500-01 |  | OS04g0660500 | | Os04g0660500 | |  | | - | |
| OS04T0661200-01 |  | OS04g0661200 | | Os04g0661200 | |  | | - | |
| OS04T0661300-02 |  | OS04g0661300 | | Os04g0661300 | |  | | - | |
| OS04T0661600-03 |  | OS04g0661600 | | Os04g0661600 | |  | | - | |
| OS04T0661900-02 |  | OS04g0661900 | | Os04g0661900 | |  | | - | |
| OS04T0662800-01 |  | OS04g0662800 | | Os04g0662800 | |  | | - | |
| OS04T0662900-02 |  | OS04g0662900 | | Os04g0662900 | |  | | - | |
| OS04T0665000-01 |  | OS04g0665000 | | Os04g0665000 | |  | | - | |
| OS04T0665400-02 |  | OS04g0665400 | | Os04g0665400 | |  | | - | |
| OS04T0665500-01 |  | OS04g0665500 | | Os04g0665500 | |  | | - | |
| OS04T0665700-01 |  | OS04g0665700 | | Os04g0665700 | |  | | - | |
| OS04T0665800-02 |  | OS04g0665800 | | Os04g0665800 | |  | | - | |
| OS04T0667600-01 |  | OS04g0667600 | | Os04g0667600 | | Down | | -Down | |
| OS04T0667800-02 |  | OS04g0667800 | | Os04g0667800 | |  | | - | |
| OS04T0668800-01 |  | OS04g0668800 | | Os04g0668800 | | Up | | -Up | |
| OS04T0669500-01 |  | OS04g0669500 | | Os04g0669500 | |  | | - | |
| OS04T0669800-01 |  | OS04g0669800 | | Os04g0669800 | |  | | - | |
| OS04T0670200-01 |  | OS04g0670200 | | Os04g0670200 | |  | | - | |
| OS04T0670800-02 |  | OS04g0670800 | | Os04g0670800 | |  | | - | |
| OS04T0671100-01 |  | OS04g0671100 | | Os04g0671100 | | Up | | -Up | |
| OS04T0671700-01 |  | OS04g0671700 | | Os04g0671700 | |  | | - | |
| OS04T0671800-01 |  | OS04g0671800 | | Os04g0671800 | |  | | - | |
| OS04T0672800-01 |  | OS04g0672800 | | Os04g0672800 | |  | | - | |
| OS04T0673300-01 |  | OS04g0673300 | | Os04g0673300 | |  | | - | |
| OS04T0674400-01 |  | OS04g0674400 | | Os04g0674400 | |  | | - | |
| OS04T0674700-01 |  | OS04g0674700 | | Os04g0674700 | |  | | - | |
| OS04T0675101-00 |  | OS04g0675101 | | Os04g0675101 | |  | | - | |
| OS04T0675400-01 |  | OS04g0675400 | | Os04g0675400 | |  | | - | |
| OS04T0675500-03 |  | OS04g0675500 | | Os04g0675500 | |  | | - | |
| OS04T0676100-01 |  | OS04g0676100 | | Os04g0676100 | |  | | - | |
| OS04T0676300-01 |  | OS04g0676300 | | Os04g0676300 | |  | | - | |
| OS04T0677500-02 |  | OS04g0677500 | | Os04g0677500 | |  | | - | |
| OS04T0678200-01 |  | OS04g0678200 | | Os04g0678200 | |  | | - | |
| OS04T0678700-01 |  | OS04g0678700 | | Os04g0678700 | | Down | | -Down | |
| OS04T0679100-01 |  | OS04g0679100 | | Os04g0679100 | |  | | - | |
| OS04T0679400-02 |  | OS04g0679400 | | Os04g0679400 | |  | | - | |
| OS04T0680400-01 |  | OS04g0680400 | | Os04g0680400 | |  | | - | |
| OS04T0680700-02 |  | OS04g0680700 | | Os04g0680700 | |  | | - | |
| OS04T0681900-02 |  | OS04g0681900 | | Os04g0681900 | |  | | - | |
| OS04T0682100-01 |  | OS04g0682100 | | Os04g0682100 | |  | | - | |
| OS04T0682300-02 |  | OS04g0682300 | | Os04g0682300 | |  | | - | |
| OS04T0682800-01 |  | OS04g0682800 | | Os04g0682800 | |  | | - | |
| OS04T0683100-02 |  | OS04g0683100 | | Os04g0683100 | |  | | - | |
| OS04T0683700-01 | Up | OS04g0683700 | | Os04g0683700 | | Up | | Up-Up | |
| OS04T0683900-03 |  | OS04g0683900 | | Os04g0683900 | |  | | - | |
| OS04T0684500-01 |  | OS04g0684500 | | Os04g0684500 | |  | | - | |
| OS04T0685300-02 |  | OS04g0685300 | | Os04g0685300 | |  | | - | |
| OS04T0685600-01 |  | OS04g0685600 | | Os04g0685600 | |  | | - | |
| OS04T0685800-01 |  | OS04g0685800 | | Os04g0685800 | |  | | - | |
| OS04T0687100-01 |  | OS04g0687100 | | Os04g0687100 | |  | | - | |
| OS04T0688100-02 |  | OS04g0688100 | | Os04g0688100 | |  | | - | |
| OS04T0688300-01 |  | OS04g0688300 | | Os04g0688300 | |  | | - | |
| OS04T0688500-01 |  | OS04g0688500 | | Os04g0688500 | |  | | - | |
| OS04T0691200-00 |  | OS04g0691200 | | Os04g0691200 | |  | | - | |
| OS04T0691500-01 |  | OS04g0691500 | | Os04g0691500 | |  | | - | |
| OS04T0691600-02 | Down | OS04g0691600 | | Os04g0691600 | | Down | | Down-Down | |
| OS04T0691800-01 |  | OS04g0691800 | | Os04g0691800 | |  | | - | |
| OS04T0692200-01 |  | OS04g0692200 | | Os04g0692200 | |  | | - | |
| OS04T0693000-01 |  | OS04g0693000 | | Os04g0693000 | |  | | - | |
| OS05T0100800-01 |  | OS05g0100800 | | Os05g0100800 | |  | | - | |
| OS05T0101200-01 | Down | OS05g0101200 | | Os05g0101200 | |  | | Down- | |
| OS05T0101400-02 |  | OS05g0101400 | | Os05g0101400 | |  | | - | |
| OS05T0102900-02 |  | OS05g0102900 | | Os05g0102900 | |  | | - | |
| OS05T0103100-01 |  | OS05g0103100 | | Os05g0103100 | |  | | - | |
| OS05T0103200-02 | Down | OS05g0103200 | | Os05g0103200 | | Down | | Down-Down | |
| OS05T0103500-01 |  | OS05g0103500 | | Os05g0103500 | |  | | - | |
| OS05T0103600-01 |  | OS05g0103600 | | Os05g0103600 | |  | | - | |
| OS05T0104650-00 |  | OS05g0104650 | | Os05g0104650 | |  | | - | |
| OS05T0104800-01 |  | OS05g0104800 | | Os05g0104800 | |  | | - | |
| OS05T0105000-01 |  | OS05g0105000 | | Os05g0105000 | |  | | - | |
| OS05T0105800-01 |  | OS05g0105800 | | Os05g0105800 | |  | | - | |
| OS05T0108800-01 |  | OS05g0108800 | | Os05g0108800 | |  | | - | |
| OS05T0110100-02 |  | OS05g0110100 | | Os05g0110100 | |  | | - | |
| OS05T0110300-02 |  | OS05g0110300 | | Os05g0110300 | | Down | | -Down | |
| OS05T0111200-01 |  | OS05g0111200 | | Os05g0111200 | | Up | | -Up | |
| OS05T0112800-01 | Up | OS05g0112800 | | Os05g0112800 | |  | | Up- | |
| OS05T0113900-01 |  | OS05g0113900 | | Os05g0113900 | |  | | - | |
| OS05T0115800-02 |  | OS05g0115800 | | Os05g0115800 | |  | | - | |
| OS05T0115900-01 | Up | OS05g0115900 | | Os05g0115900 | |  | | Up- | |
| OS05T0116000-02 |  | OS05g0116000 | | Os05g0116000 | |  | | - | |
| OS05T0116100-01 |  | OS05g0116100 | | Os05g0116100 | |  | | - | |
| OS05T0117600-00 |  | OS05g0117600 | | Os05g0117600 | |  | | - | |
| OS05T0117864-01 |  | OS05g0117864 | | Os05g0117864 | |  | | - | |
| OS05T0122800-03 |  | OS05g0122800 | | Os05g0122800 | |  | | - | |
| OS05T0122900-01 |  | OS05g0122900 | | Os05g0122900 | |  | | - | |
| OS05T0125000-01 |  | OS05g0125000 | | Os05g0125000 | |  | | - | |
| OS05T0125500-01 | Up | OS05g0125500 | | Os05g0125500 | |  | | Up- | |
| OS05T0129000-01 |  | OS05g0129000 | | Os05g0129000 | |  | | - | |
| OS05T0129100-00 |  | OS05g0129100 | | Os05g0129100 | |  | | - | |
| OS05T0129900-02 |  | OS05g0129900 | | Os05g0129900 | |  | | - | |
| OS05T0131100-01 |  | OS05g0131100 | | Os05g0131100 | |  | | - | |
| OS05T0133100-01 |  | OS05g0133100 | | Os05g0133100 | |  | | - | |
| OS05T0134400-02 |  | OS05g0134400 | | Os05g0134400 | |  | | - | |
| OS05T0135500-01 |  | OS05g0135500 | | Os05g0135500 | | Up | | -Up | |
| OS05T0135800-01 |  | OS05g0135800 | | Os05g0135800 | |  | | - | |
| OS05T0135900-01 |  | OS05g0135900 | | Os05g0135900 | |  | | - | |
| OS05T0136900-01 |  | OS05g0136900 | | Os05g0136900 | | Up | | -Up | |
| OS05T0137400-01 |  | OS05g0137400 | | Os05g0137400 | |  | | - | |
| OS05T0140500-01 |  | OS05g0140500 | | Os05g0140500 | |  | | - | |
| OS05T0141400-01 |  | OS05g0141400 | | Os05g0141400 | |  | | - | |
| OS05T0143500-02 |  | OS05g0143500 | | Os05g0143500 | |  | | - | |
| OS05T0144300-01 |  | OS05g0144300 | | Os05g0144300 | |  | | - | |
| OS05T0144400-01 |  | OS05g0144400 | | Os05g0144400 | |  | | - | |
| OS05T0145600-01 |  | OS05g0145600 | | Os05g0145600 | |  | | - | |
| OS05T0147400-01 |  | OS05g0147400 | | Os05g0147400 | |  | | - | |
| OS05T0147500-01 |  | OS05g0147500 | | Os05g0147500 | |  | | - | |
| OS05T0148700-02 |  | OS05g0148700 | | Os05g0148700 | |  | | - | |
| OS05T0149600-02 |  | OS05g0149600 | | Os05g0149600 | |  | | - | |
| OS05T0150000-01 |  | OS05g0150000 | | Os05g0150000 | |  | | - | |
| OS05T0150300-01 |  | OS05g0150300 | | Os05g0150300 | |  | | - | |
| OS05T0150800-01 |  | OS05g0150800 | | Os05g0150800 | |  | | - | |
| OS05T0150900-02 |  | OS05g0150900 | | Os05g0150900 | |  | | - | |
| OS05T0151000-00 |  | OS05g0151000 | | Os05g0151000 | |  | | - | |
| OS05T0151300-01 | Up | OS05g0151300 | | Os05g0151300 | |  | | Up- | |
| OS05T0151400-03 |  | OS05g0151400 | | Os05g0151400 | |  | | - | |
| OS05T0154800-01 |  | OS05g0154800 | | Os05g0154800 | |  | | - | |
| OS05T0155100-01 | Down | OS05g0155100 | | Os05g0155100 | |  | | Down- | |
| OS05T0155300-01 |  | OS05g0155300 | | Os05g0155300 | |  | | - | |
| OS05T0156300-03 |  | OS05g0156300 | | Os05g0156300 | |  | | - | |
| OS05T0156500-01 |  | OS05g0156500 | | Os05g0156500 | |  | | - | |
| OS05T0157200-01 | Up | OS05g0157200 | | Os05g0157200 | |  | | Up- | |
| OS05T0157300-02 |  | OS05g0157300 | | Os05g0157300 | |  | | - | |
| OS05T0158500-01 | Up | OS05g0158500 | | Os05g0158500 | |  | | Up- | |
| OS05T0160000-01 |  | OS05g0160000 | | Os05g0160000 | |  | | - | |
| OS05T0160100-01 |  | OS05g0160100 | | Os05g0160100 | |  | | - | |
| OS05T0160200-01 | Down | OS05g0160200 | | Os05g0160200 | |  | | Down- | |
| OS05T0161500-01 | Down | OS05g0161500 | | Os05g0161500 | |  | | Down- | |
| OS05T0162200-02 |  | OS05g0162200 | | Os05g0162200 | |  | | - | |
| OS05T0163000-01 |  | OS05g0163000 | | Os05g0163000 | |  | | - | |
| OS05T0163700-01 |  | OS05g0163700 | | Os05g0163700 | | Up | | -Up | |
| OS05T0164100-02 | Down | OS05g0164100 | | Os05g0164100 | |  | | Down- | |
| OS05T0169000-01 |  | OS05g0169000 | | Os05g0169000 | |  | | - | |
| OS05T0169100-01 |  | OS05g0169100 | | Os05g0169100 | |  | | - | |
| OS05T0169400-01 |  | OS05g0169400 | | Os05g0169400 | |  | | - | |
| OS05T0171200-01 |  | OS05g0171200 | | Os05g0171200 | |  | | - | |
| OS05T0177500-01 |  | OS05g0177500 | | Os05g0177500 | | Up | | -Up | |
| OS05T0178900-02 |  | OS05g0178900 | | Os05g0178900 | |  | | - | |
| OS05T0179800-01 |  | OS05g0179800 | | Os05g0179800 | |  | | - | |
| OS05T0179950-00 |  | OS05g0179950 | | Os05g0179950 | | Down | | -Down | |
| OS05T0180300-01 |  | OS05g0180300 | | Os05g0180300 | |  | | - | |
| OS05T0181000-01 |  | OS05g0181000 | | Os05g0181000 | |  | | - | |
| OS05T0182000-01 |  | OS05g0182000 | | Os05g0182000 | |  | | - | |
| OS05T0182100-01 |  | OS05g0182100 | | Os05g0182100 | |  | | - | |
| OS05T0182700-01 |  | OS05g0182700 | | Os05g0182700 | |  | | - | |
| OS05T0182800-01 |  | OS05g0182800 | | Os05g0182800 | |  | | - | |
| OS05T0185700-01 |  | OS05g0185700 | | Os05g0185700 | |  | | - | |
| OS05T0186000-03 |  | OS05g0186000 | | Os05g0186000 | |  | | - | |
| OS05T0186300-01 |  | OS05g0186300 | | Os05g0186300 | |  | | - | |
| OS05T0187000-01 |  | OS05g0187000 | | Os05g0187000 | |  | | - | |
| OS05T0188100-01 |  | OS05g0188100 | | Os05g0188100 | | Down | | -Down | |
| OS05T0188500-02 |  | OS05g0188500 | | Os05g0188500 | |  | | - | |
| OS05T0189900-01 |  | OS05g0189900 | | Os05g0189900 | |  | | - | |
| OS05T0194600-01 |  | OS05g0194600 | | Os05g0194600 | |  | | - | |
| OS05T0196100-02 |  | OS05g0196100 | | Os05g0196100 | |  | | - | |
| OS05T0200100-02 |  | OS05g0200100 | | Os05g0200100 | |  | | - | |
| OS05T0200280-01 |  | OS05g0200280 | | Os05g0200280 | |  | | - | |
| OS05T0202300-01 |  | OS05g0202300 | | Os05g0202300 | |  | | - | |
| OS05T0202800-00 |  | OS05g0202800 | | Os05g0202800 | | Up | | -Up | |
| OS05T0204900-02 |  | OS05g0204900 | | Os05g0204900 | |  | | - | |
| OS05T0207300-01 |  | OS05g0207300 | | Os05g0207300 | |  | | - | |
| OS05T0207900-02 |  | OS05g0207900 | | Os05g0207900 | |  | | - | |
| OS05T0208000-03 | Up | OS05g0208000 | | Os05g0208000 | |  | | Up- | |
| OS05T0209100-01 |  | OS05g0209100 | | Os05g0209100 | |  | | - | |
| OS05T0209400-01 |  | OS05g0209400 | | Os05g0209400 | |  | | - | |
| OS05T0209600-01 |  | OS05g0209600 | | Os05g0209600 | |  | | - | |
| OS05T0215700-02 |  | OS05g0215700 | | Os05g0215700 | | Down | | -Down | |
| OS05T0218400-01 |  | OS05g0218400 | | Os05g0218400 | |  | | - | |
| OS05T0223000-01 |  | OS05g0223000 | | Os05g0223000 | |  | | - | |
| OS05T0227700-02 |  | OS05g0227700 | | Os05g0227700 | |  | | - | |
| OS05T0230600-01 |  | OS05g0230600 | | Os05g0230600 | |  | | - | |
| OS05T0230900-01 |  | OS05g0230900 | | Os05g0230900 | |  | | - | |
| OS05T0232200-01 |  | OS05g0232200 | | Os05g0232200 | |  | | - | |
| OS05T0243300-01 |  | OS05g0243300 | | Os05g0243300 | |  | | - | |
| OS05T0244500-01 |  | OS05g0244500 | | Os05g0244500 | |  | | - | |
| OS05T0244600-01 | Down | OS05g0244600 | | Os05g0244600 | |  | | Down- | |
| OS05T0247100-02 |  | OS05g0247100 | | Os05g0247100 | |  | | - | |
| OS05T0268500-01 |  | OS05g0268500 | | Os05g0268500 | |  | | - | |
| OS05T0270200-01 |  | OS05g0270200 | | Os05g0270200 | |  | | - | |
| OS05T0272800-01 |  | OS05g0272800 | | Os05g0272800 | |  | | - | |
| OS05T0272900-01 |  | OS05g0272900 | | Os05g0272900 | |  | | - | |
| OS05T0273800-01 |  | OS05g0273800 | | Os05g0273800 | |  | | - | |
| OS05T0274900-00 |  | OS05g0274900 | | Os05g0274900 | |  | | - | |
| OS05T0278500-01 |  | OS05g0278500 | | Os05g0278500 | |  | | - | |
| OS05T0279400-02 |  | OS05g0279400 | | Os05g0279400 | |  | | - | |
| OS05T0289400-01 |  | OS05g0289400 | | Os05g0289400 | |  | | - | |
| OS05T0291700-03 | Down | OS05g0291700 | | Os05g0291700 | | Down | | Down-Down | |
| OS05T0292200-01 |  | OS05g0292200 | | Os05g0292200 | |  | | - | |
| OS05T0292800-01 |  | OS05g0292800 | | Os05g0292800 | |  | | - | |
| OS05T0295300-01 |  | OS05g0295300 | | Os05g0295300 | |  | | - | |
| OS05T0295800-01 |  | OS05g0295800 | | Os05g0295800 | |  | | - | |
| OS05T0301500-01 |  | OS05g0301500 | | Os05g0301500 | |  | | - | |
| OS05T0301600-01 |  | OS05g0301600 | | Os05g0301600 | |  | | - | |
| OS05T0301700-01 |  | OS05g0301700 | | Os05g0301700 | |  | | - | |
| OS05T0302700-01 | Up | OS05g0302700 | | Os05g0302700 | |  | | Up- | |
| OS05T0303000-01 |  | OS05g0303000 | | Os05g0303000 | |  | | - | |
| OS05T0304400-01 |  | OS05g0304400 | | Os05g0304400 | |  | | - | |
| OS05T0304600-02 |  | OS05g0304600 | | Os05g0304600 | |  | | - | |
| OS05T0306000-01 |  | OS05g0306000 | | Os05g0306000 | |  | | - | |
| OS05T0310800-01 |  | OS05g0310800 | | Os05g0310800 | |  | | - | |
| OS05T0313500-01 |  | OS05g0313500 | | Os05g0313500 | |  | | - | |
| OS05T0314100-02 |  | OS05g0314100 | | Os05g0314100 | |  | | - | |
| OS05T0316200-02 |  | OS05g0316200 | | Os05g0316200 | |  | | - | |
| OS05T0317200-01 |  | OS05g0317200 | | Os05g0317200 | |  | | - | |
| OS05T0318300-01 |  | OS05g0318300 | | Os05g0318300 | |  | | - | |
| OS05T0318700-01 |  | OS05g0318700 | | Os05g0318700 | |  | | - | |
| OS05T0320700-02 |  | OS05g0320700 | | Os05g0320700 | |  | | - | |
| OS05T0323800-01 | Up | OS05g0323800 | | Os05g0323800 | | Up | | Up-Up | |
| OS05T0323900-01 | Up | OS05g0323900 | | Os05g0323900 | |  | | Up- | |
| OS05T0334400-03 |  | OS05g0334400 | | Os05g0334400 | |  | | - | |
| OS05T0346100-01 |  | OS05g0346100 | | Os05g0346100 | |  | | - | |
| OS05T0346300-01 |  | OS05g0346300 | | Os05g0346300 | |  | | - | |
| OS05T0347000-01 |  | OS05g0347000 | | Os05g0347000 | |  | | - | |
| OS05T0349200-01 |  | OS05g0349200 | | Os05g0349200 | |  | | - | |
| OS05T0349500-01 |  | OS05g0349500 | | Os05g0349500 | |  | | - | |
| OS05T0350500-01 |  | OS05g0350500 | | Os05g0350500 | |  | | - | |
| OS05T0350600-01 |  | OS05g0350600 | | Os05g0350600 | |  | | - | |
| OS05T0352800-01 |  | OS05g0352800 | | Os05g0352800 | |  | | - | |
| OS05T0353400-01 |  | OS05g0353400 | | Os05g0353400 | |  | | - | |
| OS05T0355400-01 |  | OS05g0355400 | | Os05g0355400 | |  | | - | |
| OS05T0357600-02 |  | OS05g0357600 | | Os05g0357600 | |  | | - | |
| OS05T0358400-01 |  | OS05g0358400 | | Os05g0358400 | |  | | - | |
| OS05T0361200-01 |  | OS05g0361200 | | Os05g0361200 | |  | | - | |
| OS05T0361900-01 |  | OS05g0361900 | | Os05g0361900 | | Down | | -Down | |
| OS05T0362500-01 |  | OS05g0362500 | | Os05g0362500 | |  | | - | |
| OS05T0363200-01 |  | OS05g0363200 | | Os05g0363200 | |  | | - | |
| OS05T0365500-01 |  | OS05g0365500 | | Os05g0365500 | |  | | - | |
| OS05T0365600-01 |  | OS05g0365600 | | Os05g0365600 | |  | | - | |
| OS05T0366600-01 |  | OS05g0366600 | | Os05g0366600 | | Up | | -Up | |
| OS05T0367100-01 |  | OS05g0367100 | | Os05g0367100 | |  | | - | |
| OS05T0367400-01 |  | OS05g0367400 | | Os05g0367400 | |  | | - | |
| OS05T0368300-01 |  | OS05g0368300 | | Os05g0368300 | |  | | - | |
| OS05T0370600-02 |  | OS05g0370600 | | Os05g0370600 | |  | | - | |
| OS05T0371200-01 |  | OS05g0371200 | | Os05g0371200 | |  | | - | |
| OS05T0371500-01 |  | OS05g0371500 | | Os05g0371500 | |  | | - | |
| OS05T0373300-01 |  | OS05g0373300 | | Os05g0373300 | |  | | - | |
| OS05T0373700-01 |  | OS05g0373700 | | Os05g0373700 | |  | | - | |
| OS05T0375400-01 |  | OS05g0375400 | | Os05g0375400 | |  | | - | |
| OS05T0377000-01 |  | OS05g0377000 | | Os05g0377000 | |  | | - | |
| OS05T0378900-01 |  | OS05g0378900 | | Os05g0378900 | |  | | - | |
| OS05T0381500-02 |  | OS05g0381500 | | Os05g0381500 | |  | | - | |
| OS05T0383000-02 |  | OS05g0383000 | | Os05g0383000 | |  | | - | |
| OS05T0387200-01 | Up | OS05g0387200 | | Os05g0387200 | |  | | Up- | |
| OS05T0387700-02 |  | OS05g0387700 | | Os05g0387700 | |  | | - | |
| OS05T0388400-01 |  | OS05g0388400 | | Os05g0388400 | |  | | - | |
| OS05T0388500-01 |  | OS05g0388500 | | Os05g0388500 | |  | | - | |
| OS05T0388600-01 | Up | OS05g0388600 | | Os05g0388600 | |  | | Up- | |
| OS05T0389500-02 |  | OS05g0389500 | | Os05g0389500 | |  | | - | |
| OS05T0390100-01 |  | OS05g0390100 | | Os05g0390100 | |  | | - | |
| OS05T0390500-01 |  | OS05g0390500 | | Os05g0390500 | |  | | - | |
| OS05T0391500-01 |  | OS05g0391500 | | Os05g0391500 | |  | | - | |
| OS05T0392100-01 |  | OS05g0392100 | | Os05g0392100 | |  | | - | |
| OS05T0392200-01 |  | OS05g0392200 | | Os05g0392200 | |  | | - | |
| OS05T0392300-01 |  | OS05g0392300 | | Os05g0392300 | |  | | - | |
| OS05T0392700-03 |  | OS05g0392700 | | Os05g0392700 | |  | | - | |
| OS05T0393100-01 |  | OS05g0393100 | | Os05g0393100 | |  | | - | |
| OS05T0395300-01 |  | OS05g0395300 | | Os05g0395300 | |  | | - | |
| OS05T0399100-02 | Up | OS05g0399100 | | Os05g0399100 | |  | | Up- | |
| OS05T0400400-01 |  | OS05g0400400 | | Os05g0400400 | |  | | - | |
| OS05T0400700-01 |  | OS05g0400700 | | Os05g0400700 | |  | | - | |
| OS05T0400800-02 |  | OS05g0400800 | | Os05g0400800 | |  | | - | |
| OS05T0401000-01 |  | OS05g0401000 | | Os05g0401000 | |  | | - | |
| OS05T0401100-02 |  | OS05g0401100 | | Os05g0401100 | | Down | | -Down | |
| OS05T0401200-01 |  | OS05g0401200 | | Os05g0401200 | |  | | - | |
| OS05T0401300-01 |  | OS05g0401300 | | Os05g0401300 | |  | | - | |
| OS05T0402700-01 | Up | OS05g0402700 | | Os05g0402700 | |  | | Up- | |
| OS05T0402800-01 |  | OS05g0402800 | | Os05g0402800 | |  | | - | |
| OS05T0403400-01 |  | OS05g0403400 | | Os05g0403400 | |  | | - | |
| OS05T0404200-02 |  | OS05g0404200 | | Os05g0404200 | |  | | - | |
| OS05T0404300-01 |  | OS05g0404300 | | Os05g0404300 | |  | | - | |
| OS05T0405000-01 |  | OS05g0405000 | | Os05g0405000 | | Up | | -Up | |
| OS05T0406000-01 |  | OS05g0406000 | | Os05g0406000 | |  | | - | |
| OS05T0408900-04 |  | OS05g0408900 | | Os05g0408900 | |  | | - | |
| OS05T0409000-01 |  | OS05g0409000 | | Os05g0409000 | |  | | - | |
| OS05T0410200-01 | Down | OS05g0410200 | | Os05g0410200 | |  | | Down- | |
| OS05T0411200-01 |  | OS05g0411200 | | Os05g0411200 | | Down | | -Down | |
| OS05T0413200-01 | Down | OS05g0413200 | | Os05g0413200 | | Down | | Down-Down | |
| OS05T0414700-01 |  | OS05g0414700 | | Os05g0414700 | |  | | - | |
| OS05T0415700-01 |  | OS05g0415700 | | Os05g0415700 | |  | | - | |
| OS05T0418000-01 |  | OS05g0418000 | | Os05g0418000 | |  | | - | |
| OS05T0419100-01 |  | OS05g0419100 | | Os05g0419100 | |  | | - | |
| OS05T0420600-01 |  | OS05g0420600 | | Os05g0420600 | |  | | - | |
| OS05T0429400-01 |  | OS05g0429400 | | Os05g0429400 | |  | | - | |
| OS05T0429500-02 |  | OS05g0429500 | | Os05g0429500 | |  | | - | |
| OS05T0430400-01 |  | OS05g0430400 | | Os05g0430400 | |  | | - | |
| OS05T0430800-01 |  | OS05g0430800 | | Os05g0430800 | |  | | - | |
| OS05T0432400-03 |  | OS05g0432400 | | Os05g0432400 | |  | | - | |
| OS05T0432700-01 |  | OS05g0432700 | | Os05g0432700 | |  | | - | |
| OS05T0435400-01 |  | OS05g0435400 | | Os05g0435400 | |  | | - | |
| OS05T0435700-01 |  | OS05g0435700 | | Os05g0435700 | |  | | - | |
| OS05T0435800-01 |  | OS05g0435800 | | Os05g0435800 | |  | | - | |
| OS05T0437300-01 |  | OS05g0437300 | | Os05g0437300 | |  | | - | |
| OS05T0438800-01 |  | OS05g0438800 | | Os05g0438800 | |  | | - | |
| OS05T0440250-01 |  | OS05g0440250 | | Os05g0440250 | |  | | - | |
| OS05T0443300-01 |  | OS05g0443300 | | Os05g0443300 | |  | | - | |
| OS05T0443500-01 |  | OS05g0443500 | | Os05g0443500 | |  | | - | |
| OS05T0445500-02 |  | OS05g0445500 | | Os05g0445500 | |  | | - | |
| OS05T0446800-03 |  | OS05g0446800 | | Os05g0446800 | |  | | - | |
| OS05T0449600-01 |  | OS05g0449600 | | Os05g0449600 | |  | | - | |
| OS05T0450600-01 |  | OS05g0450600 | | Os05g0450600 | |  | | - | |
| OS05T0455500-02 |  | OS05g0455500 | | Os05g0455500 | |  | | - | |
| OS05T0455700-02 |  | OS05g0455700 | | Os05g0455700 | |  | | - | |
| OS05T0456300-02 |  | OS05g0456300 | | Os05g0456300 | |  | | - | |
| OS05T0457700-01 |  | OS05g0457700 | | Os05g0457700 | |  | | - | |
| OS05T0458400-01 |  | OS05g0458400 | | Os05g0458400 | |  | | - | |
| OS05T0459900-01 |  | OS05g0459900 | | Os05g0459900 | |  | | - | |
| OS05T0460000-01 |  | OS05g0460000 | | Os05g0460000 | | Up | | -Up | |
| OS05T0460600-01 |  | OS05g0460600 | | Os05g0460600 | | Down | | -Down | |
| OS05T0461600-01 |  | OS05g0461600 | | Os05g0461600 | |  | | - | |
| OS05T0462000-00 |  | OS05g0462000 | | Os05g0462000 | | Down | | -Down | |
| OS05T0463500-01 |  | OS05g0463500 | | Os05g0463500 | |  | | - | |
| OS05T0465800-01 |  | OS05g0465800 | | Os05g0465800 | |  | | - | |
| OS05T0468600-00 |  | OS05g0468600 | | Os05g0468600 | |  | | - | |
| OS05T0468700-01 |  | OS05g0468700 | | Os05g0468700 | |  | | - | |
| OS05T0468800-01 |  | OS05g0468800 | | Os05g0468800 | |  | | - | |
| OS05T0470800-01 |  | OS05g0470800 | | Os05g0470800 | |  | | - | |
| OS05T0473500-02 |  | OS05g0473500 | | Os05g0473500 | |  | | - | |
| OS05T0474400-01 |  | OS05g0474400 | | Os05g0474400 | |  | | - | |
| OS05T0474500-01 |  | OS05g0474500 | | Os05g0474500 | |  | | - | |
| OS05T0475300-01 |  | OS05g0475300 | | Os05g0475300 | |  | | - | |
| OS05T0475400-01 |  | OS05g0475400 | | Os05g0475400 | |  | | - | |
| OS05T0477300-03 |  | OS05g0477300 | | Os05g0477300 | |  | | - | |
| OS05T0477900-01 |  | OS05g0477900 | | Os05g0477900 | | Down | | -Down | |
| OS05T0480000-01 | Down | OS05g0480000 | | Os05g0480000 | |  | | Down- | |
| OS05T0481000-01 |  | OS05g0481000 | | Os05g0481000 | |  | | - | |
| OS05T0481100-04 |  | OS05g0481100 | | Os05g0481100 | |  | | - | |
| OS05T0481600-01 |  | OS05g0481600 | | Os05g0481600 | |  | | - | |
| OS05T0482700-01 |  | OS05g0482700 | | Os05g0482700 | |  | | - | |
| OS05T0486700-01 | Down | OS05g0486700 | | Os05g0486700 | |  | | Down- | |
| OS05T0488000-01 |  | OS05g0488000 | | Os05g0488000 | |  | | - | |
| OS05T0488900-01 |  | OS05g0488900 | | Os05g0488900 | |  | | - | |
| OS05T0489600-03 |  | OS05g0489600 | | Os05g0489600 | |  | | - | |
| OS05T0490100-01 |  | OS05g0490100 | | Os05g0490100 | |  | | - | |
| OS05T0490200-02 |  | OS05g0490200 | | Os05g0490200 | |  | | - | |
| OS05T0490700-01 |  | OS05g0490700 | | Os05g0490700 | |  | | - | |
| OS05T0490800-01 |  | OS05g0490800 | | Os05g0490800 | | Down | | -Down | |
| OS05T0494000-01 |  | OS05g0494000 | | Os05g0494000 | |  | | - | |
| OS05T0494200-02 |  | OS05g0494200 | | Os05g0494200 | |  | | - | |
| OS05T0495100-01 |  | OS05g0495100 | | Os05g0495100 | |  | | - | |
| OS05T0496200-01 |  | OS05g0496200 | | Os05g0496200 | | Down | | -Down | |
| OS05T0499500-01 |  | OS05g0499500 | | Os05g0499500 | |  | | - | |
| OS05T0499600-01 |  | OS05g0499600 | | Os05g0499600 | | Up | | -Up | |
| OS05T0500000-00 |  | OS05g0500000 | | Os05g0500000 | |  | | - | |
| OS05T0500400-01 |  | OS05g0500400 | | Os05g0500400 | |  | | - | |
| OS05T0500450-00 |  | OS05g0500450 | | Os05g0500450 | |  | | - | |
| OS05T0501300-01 |  | OS05g0501300 | | Os05g0501300 | |  | | - | |
| OS05T0501700-01 |  | OS05g0501700 | | Os05g0501700 | |  | | - | |
| OS05T0502500-01 |  | OS05g0502500 | | Os05g0502500 | |  | | - | |
| OS05T0503300-03 |  | OS05g0503300 | | Os05g0503300 | |  | | - | |
| OS05T0506200-01 |  | OS05g0506200 | | Os05g0506200 | |  | | - | |
| OS05T0507300-01 |  | OS05g0507300 | | Os05g0507300 | |  | | - | |
| OS05T0508300-01 | Up | OS05g0508300 | | Os05g0508300 | |  | | Up- | |
| OS05T0508500-02 |  | OS05g0508500 | | Os05g0508500 | |  | | - | |
| OS05T0508700-02 |  | OS05g0508700 | | Os05g0508700 | |  | | - | |
| OS05T0509200-01 |  | OS05g0509200 | | Os05g0509200 | |  | | - | |
| OS05T0509300-01 |  | OS05g0509300 | | Os05g0509300 | |  | | - | |
| OS05T0509400-01 |  | OS05g0509400 | | Os05g0509400 | |  | | - | |
| OS05T0510700-01 |  | OS05g0510700 | | Os05g0510700 | |  | | - | |
| OS05T0511300-01 |  | OS05g0511300 | | Os05g0511300 | |  | | - | |
| OS05T0511400-01 |  | OS05g0511400 | | Os05g0511400 | |  | | - | |
| OS05T0511700-01 |  | OS05g0511700 | | Os05g0511700 | |  | | - | |
| OS05T0512200-01 |  | OS05g0512200 | | Os05g0512200 | |  | | - | |
| OS05T0512400-01 |  | OS05g0512400 | | Os05g0512400 | |  | | - | |
| OS05T0513300-00 |  | OS05g0513300 | | Os05g0513300 | |  | | - | |
| OS05T0513900-02 |  | OS05g0513900 | | Os05g0513900 | |  | | - | |
| OS05T0515700-01 |  | OS05g0515700 | | Os05g0515700 | |  | | - | |
| OS05T0516600-01 |  | OS05g0516600 | | Os05g0516600 | |  | | - | |
| OS05T0517500-01 |  | OS05g0517500 | | Os05g0517500 | |  | | - | |
| OS05T0519400-01 |  | OS05g0519400 | | Os05g0519400 | |  | | - | |
| OS05T0519700-02 |  | OS05g0519700 | | Os05g0519700 | | Up | | -Up | |
| OS05T0519800-01 |  | OS05g0519800 | | Os05g0519800 | |  | | - | |
| OS05T0519900-03 |  | OS05g0519900 | | Os05g0519900 | |  | | - | |
| OS05T0520100-00 | Down | OS05g0520100 | | Os05g0520100 | |  | | Down- | |
| OS05T0520200-02 |  | OS05g0520200 | | Os05g0520200 | |  | | - | |
| OS05T0522500-01 |  | OS05g0522500 | | Os05g0522500 | |  | | - | |
| OS05T0522600-01 |  | OS05g0522600 | | Os05g0522600 | | Up | | -Up | |
| OS05T0524300-01 |  | OS05g0524300 | | Os05g0524300 | |  | | - | |
| OS05T0524400-02 |  | OS05g0524400 | | Os05g0524400 | |  | | - | |
| OS05T0526200-01 |  | OS05g0526200 | | Os05g0526200 | |  | | - | |
| OS05T0526800-01 |  | OS05g0526800 | | Os05g0526800 | | Up | | -Up | |
| OS05T0528900-01 |  | OS05g0528900 | | Os05g0528900 | |  | | - | |
| OS05T0529000-01 |  | OS05g0529000 | | Os05g0529000 | |  | | - | |
| OS05T0529200-02 |  | OS05g0529200 | | Os05g0529200 | |  | | - | |
| OS05T0529400-01 |  | OS05g0529400 | | Os05g0529400 | |  | | - | |
| OS05T0530500-01 |  | OS05g0530500 | | Os05g0530500 | |  | | - | |
| OS05T0533100-01 |  | OS05g0533100 | | Os05g0533100 | |  | | - | |
| OS05T0533500-01 |  | OS05g0533500 | | Os05g0533500 | |  | | - | |
| OS05T0533600-01 |  | OS05g0533600 | | Os05g0533600 | |  | | - | |
| OS05T0534600-02 |  | OS05g0534600 | | Os05g0534600 | |  | | - | |
| OS05T0535800-00 |  | OS05g0535800 | | Os05g0535800 | |  | | - | |
| OS05T0536200-01 |  | OS05g0536200 | | Os05g0536200 | |  | | - | |
| OS05T0536400-01 |  | OS05g0536400 | | Os05g0536400 | |  | | - | |
| OS05T0539400-01 |  | OS05g0539400 | | Os05g0539400 | |  | | - | |
| OS05T0539700-01 |  | OS05g0539700 | | Os05g0539700 | |  | | - | |
| OS05T0540000-01 |  | OS05g0540000 | | Os05g0540000 | |  | | - | |
| OS05T0540300-01 |  | OS05g0540300 | | Os05g0540300 | |  | | - | |
| OS05T0540800-01 |  | OS05g0540800 | | Os05g0540800 | |  | | - | |
| OS05T0541900-01 | Down | OS05g0541900 | | Os05g0541900 | |  | | Down- | |
| OS05T0542100-01 |  | OS05g0542100 | | Os05g0542100 | |  | | - | |
| OS05T0542500-02 |  | OS05g0542500 | | Os05g0542500 | |  | | - | |
| OS05T0542600-01 |  | OS05g0542600 | | Os05g0542600 | |  | | - | |
| OS05T0542900-01 |  | OS05g0542900 | | Os05g0542900 | |  | | - | |
| OS05T0543200-01 |  | OS05g0543200 | | Os05g0543200 | |  | | - | |
| OS05T0543700-02 |  | OS05g0543700 | | Os05g0543700 | |  | | - | |
| OS05T0545600-00 |  | OS05g0545600 | | Os05g0545600 | |  | | - | |
| OS05T0548100-02 |  | OS05g0548100 | | Os05g0548100 | |  | | - | |
| OS05T0548500-00 |  | OS05g0548500 | | Os05g0548500 | |  | | - | |
| OS05T0549000-02 |  | OS05g0549000 | | Os05g0549000 | |  | | - | |
| OS05T0549100-02 |  | OS05g0549100 | | Os05g0549100 | |  | | - | |
| OS05T0549700-02 |  | OS05g0549700 | | Os05g0549700 | |  | | - | |
| OS05T0550250-00 |  | OS05g0550250 | | Os05g0550250 | |  | | - | |
| OS05T0550300-01 |  | OS05g0550300 | | Os05g0550300 | | Up | | -Up | |
| OS05T0550600-02 |  | OS05g0550600 | | Os05g0550600 | |  | | - | |
| OS05T0551900-00 |  | OS05g0551900 | | Os05g0551900 | |  | | - | |
| OS05T0552300-02 |  | OS05g0552300 | | Os05g0552300 | |  | | - | |
| OS05T0552500-01 |  | OS05g0552500 | | Os05g0552500 | |  | | - | |
| OS05T0553000-02 |  | OS05g0553000 | | Os05g0553000 | |  | | - | |
| OS05T0553700-01 |  | OS05g0553700 | | Os05g0553700 | |  | | - | |
| OS05T0554100-01 |  | OS05g0554100 | | Os05g0554100 | |  | | - | |
| OS05T0555300-02 |  | OS05g0555300 | | Os05g0555300 | |  | | - | |
| OS05T0555600-02 |  | OS05g0555600 | | Os05g0555600 | | Down | | -Down | |
| OS05T0556000-02 |  | OS05g0556000 | | Os05g0556000 | | Up | | -Up | |
| OS05T0556100-01 |  | OS05g0556100 | | Os05g0556100 | |  | | - | |
| OS05T0557000-01 |  | OS05g0557000 | | Os05g0557000 | |  | | - | |
| OS05T0557200-02 |  | OS05g0557200 | | Os05g0557200 | |  | | - | |
| OS05T0557800-02 |  | OS05g0557800 | | Os05g0557800 | |  | | - | |
| OS05T0558000-01 |  | OS05g0558000 | | Os05g0558000 | |  | | - | |
| OS05T0558400-01 |  | OS05g0558400 | | Os05g0558400 | |  | | - | |
| OS05T0558800-03 |  | OS05g0558800 | | Os05g0558800 | |  | | - | |
| OS05T0558900-01 |  | OS05g0558900 | | Os05g0558900 | |  | | - | |
| OS05T0559900-01 |  | OS05g0559900 | | Os05g0559900 | |  | | - | |
| OS05T0560000-01 |  | OS05g0560000 | | Os05g0560000 | |  | | - | |
| OS05T0561500-01 |  | OS05g0561500 | | Os05g0561500 | |  | | - | |
| OS05T0562200-01 |  | OS05g0562200 | | Os05g0562200 | |  | | - | |
| OS05T0562300-01 |  | OS05g0562300 | | Os05g0562300 | |  | | - | |
| OS05T0562400-01 |  | OS05g0562400 | | Os05g0562400 | |  | | - | |
| OS05T0563050-01 |  | OS05g0563050 | | Os05g0563050 | |  | | - | |
| OS05T0563600-01 |  | OS05g0563600 | | Os05g0563600 | | Down | | -Down | |
| OS05T0564200-01 |  | OS05g0564200 | | Os05g0564200 | |  | | - | |
| OS05T0565000-01 |  | OS05g0565000 | | Os05g0565000 | |  | | - | |
| OS05T0565200-01 |  | OS05g0565200 | | Os05g0565200 | |  | | - | |
| OS05T0566500-02 |  | OS05g0566500 | | Os05g0566500 | |  | | - | |
| OS05T0566600-01 |  | OS05g0566600 | | Os05g0566600 | |  | | - | |
| OS05T0566900-01 |  | OS05g0566900 | | Os05g0566900 | |  | | - | |
| OS05T0567100-02 | Down | OS05g0567100 | | Os05g0567100 | |  | | Down- | |
| OS05T0567300-01 |  | OS05g0567300 | | Os05g0567300 | |  | | - | |
| OS05T0567400-01 |  | OS05g0567400 | | Os05g0567400 | |  | | - | |
| OS05T0568300-00 |  | OS05g0568300 | | Os05g0568300 | | Down | | -Down | |
| OS05T0568900-01 |  | OS05g0568900 | | Os05g0568900 | |  | | - | |
| OS05T0569200-01 |  | OS05g0569200 | | Os05g0569200 | |  | | - | |
| OS05T0571100-02 |  | OS05g0571100 | | Os05g0571100 | |  | | - | |
| OS05T0573700-01 |  | OS05g0573700 | | Os05g0573700 | |  | | - | |
| OS05T0574300-00 |  | OS05g0574300 | | Os05g0574300 | |  | | - | |
| OS05T0574400-01 |  | OS05g0574400 | | Os05g0574400 | |  | | - | |
| OS05T0574500-01 |  | OS05g0574500 | | Os05g0574500 | |  | | - | |
| OS05T0575300-01 |  | OS05g0575300 | | Os05g0575300 | |  | | - | |
| OS05T0575300-02 | Down | OS05g0575300 | | Os05g0575300 | |  | | Down- | |
| OS05T0578000-01 |  | OS05g0578000 | | Os05g0578000 | |  | | - | |
| OS05T0579300-01 |  | OS05g0579300 | | Os05g0579300 | | Down | | -Down | |
| OS05T0580000-01 |  | OS05g0580000 | | Os05g0580000 | | Up | | -Up | |
| OS05T0581800-02 |  | OS05g0581800 | | Os05g0581800 | |  | | - | |
| OS05T0582800-02 | Up | OS05g0582800 | | Os05g0582800 | |  | | Up- | |
| OS05T0583075-00 |  | OS05g0583075 | | Os05g0583075 | |  | | - | |
| OS05T0583100-02 |  | OS05g0583100 | | Os05g0583100 | |  | | - | |
| OS05T0584200-02 |  | OS05g0584200 | | Os05g0584200 | |  | | - | |
| OS05T0585900-01 |  | OS05g0585900 | | Os05g0585900 | |  | | - | |
| OS05T0587100-01 |  | OS05g0587100 | | Os05g0587100 | |  | | - | |
| OS05T0589600-01 |  | OS05g0589600 | | Os05g0589600 | |  | | - | |
| OS05T0590000-02 |  | OS05g0590000 | | Os05g0590000 | |  | | - | |
| OS05T0591900-06 |  | OS05g0591900 | | Os05g0591900 | |  | | - | |
| OS05T0592000-00 |  | OS05g0592000 | | Os05g0592000 | |  | | - | |
| OS05T0592100-01 |  | OS05g0592100 | | Os05g0592100 | |  | | - | |
| OS05T0592400-01 |  | OS05g0592400 | | Os05g0592400 | |  | | - | |
| OS05T0592500-01 |  | OS05g0592500 | | Os05g0592500 | |  | | - | |
| OS05T0592600-01 |  | OS05g0592600 | | Os05g0592600 | |  | | - | |
| OS05T0592800-03 |  | OS05g0592800 | | Os05g0592800 | |  | | - | |
| OS05T0593100-01 |  | OS05g0593100 | | Os05g0593100 | |  | | - | |
| OS05T0593200-01 |  | OS05g0593200 | | Os05g0593200 | |  | | - | |
| OS05T0594500-02 |  | OS05g0594500 | | Os05g0594500 | |  | | - | |
| OS05T0594800-01 |  | OS05g0594800 | | Os05g0594800 | |  | | - | |
| OS05T0595400-02 |  | OS05g0595400 | | Os05g0595400 | |  | | - | |
| OS05T0597100-01 |  | OS05g0597100 | | Os05g0597100 | |  | | - | |
| OS06T0101300-01 |  | OS06g0101300 | | Os06g0101300 | |  | | - | |
| OS06T0101600-01 |  | OS06g0101600 | | Os06g0101600 | |  | | - | |
| OS06T0102700-01 |  | OS06g0102700 | | Os06g0102700 | |  | | - | |
| OS06T0103300-02 |  | OS06g0103300 | | Os06g0103300 | |  | | - | |
| OS06T0103400-02 |  | OS06g0103400 | | Os06g0103400 | |  | | - | |
| OS06T0103500-02 |  | OS06g0103500 | | Os06g0103500 | |  | | - | |
| OS06T0103600-01 | Down | OS06g0103600 | | Os06g0103600 | |  | | Down- | |
| OS06T0103800-01 |  | OS06g0103800 | | Os06g0103800 | |  | | - | |
| OS06T0104000-01 |  | OS06g0104000 | | Os06g0104000 | |  | | - | |
| OS06T0104300-01 |  | OS06g0104300 | | Os06g0104300 | |  | | - | |
| OS06T0104400-01 | Up | OS06g0104400 | | Os06g0104400 | |  | | Up- | |
| OS06T0105400-02 |  | OS06g0105400 | | Os06g0105400 | |  | | - | |
| OS06T0105900-01 |  | OS06g0105900 | | Os06g0105900 | |  | | - | |
| OS06T0106000-02 | Up | OS06g0106000 | | Os06g0106000 | |  | | Up- | |
| OS06T0106100-02 |  | OS06g0106100 | | Os06g0106100 | |  | | - | |
| OS06T0107700-01 | Down | OS06g0107700 | | Os06g0107700 | |  | | Down- | |
| OS06T0108900-02 |  | OS06g0108900 | | Os06g0108900 | |  | | - | |
| OS06T0110100-01 |  | OS06g0110100 | | Os06g0110100 | |  | | - | |
| OS06T0111400-02 |  | OS06g0111400 | | Os06g0111400 | |  | | - | |
| OS06T0111500-01 |  | OS06g0111500 | | Os06g0111500 | |  | | - | |
| OS06T0112400-01 |  | OS06g0112400 | | Os06g0112400 | |  | | - | |
| OS06T0113900-03 |  | OS06g0113900 | | Os06g0113900 | |  | | - | |
| OS06T0114000-03 |  | OS06g0114000 | | Os06g0114000 | | Down | | -Down | |
| OS06T0114500-02 |  | OS06g0114500 | | Os06g0114500 | |  | | - | |
| OS06T0115100-01 | Up | OS06g0115100 | | Os06g0115100 | |  | | Up- | |
| OS06T0115200-01 |  | OS06g0115200 | | Os06g0115200 | |  | | - | |
| OS06T0115300-01 |  | OS06g0115300 | | Os06g0115300 | |  | | - | |
| OS06T0115400-01 |  | OS06g0115400 | | Os06g0115400 | |  | | - | |
| OS06T0115500-01 |  | OS06g0115500 | | Os06g0115500 | |  | | - | |
| OS06T0116800-02 |  | OS06g0116800 | | Os06g0116800 | |  | | - | |
| OS06T0118700-02 | Down | OS06g0118700 | | Os06g0118700 | |  | | Down- | |
| OS06T0118800-00 |  | OS06g0118800 | | Os06g0118800 | |  | | - | |
| OS06T0119600-01 | Down | OS06g0119600 | | Os06g0119600 | |  | | Down- | |
| OS06T0120732-00 |  | OS06g0120732 | | Os06g0120732 | |  | | - | |
| OS06T0128000-00 |  | OS06g0128000 | | Os06g0128000 | |  | | - | |
| OS06T0128300-02 |  | OS06g0128300 | | Os06g0128300 | |  | | - | |
| OS06T0128500-01 |  | OS06g0128500 | | Os06g0128500 | |  | | - | |
| OS06T0129700-01 |  | OS06g0129700 | | Os06g0129700 | |  | | - | |
| OS06T0130000-03 |  | OS06g0130000 | | Os06g0130000 | |  | | - | |
| OS06T0130400-01 |  | OS06g0130400 | | Os06g0130400 | |  | | - | |
| OS06T0130500-01 |  | OS06g0130500 | | Os06g0130500 | |  | | - | |
| OS06T0130800-01 |  | OS06g0130800 | | Os06g0130800 | |  | | - | |
| OS06T0133800-01 |  | OS06g0133800 | | Os06g0133800 | |  | | - | |
| OS06T0133900-01 | Up | OS06g0133900 | | Os06g0133900 | |  | | Up- | |
| OS06T0134000-01 |  | OS06g0134000 | | Os06g0134000 | |  | | - | |
| OS06T0134050-01 |  | OS06g0134050 | | Os06g0134050 | |  | | - | |
| OS06T0134800-01 |  | OS06g0134800 | | Os06g0134800 | |  | | - | |
| OS06T0136000-02 |  | OS06g0136000 | | Os06g0136000 | |  | | - | |
| OS06T0136600-01 | Down | OS06g0136600 | | Os06g0136600 | | Down | | Down-Down | |
| OS06T0136800-01 |  | OS06g0136800 | | Os06g0136800 | |  | | - | |
| OS06T0137300-01 |  | OS06g0137300 | | Os06g0137300 | |  | | - | |
| OS06T0137600-02 |  | OS06g0137600 | | Os06g0137600 | |  | | - | |
| OS06T0137700-03 |  | OS06g0137700 | | Os06g0137700 | |  | | - | |
| OS06T0138100-01 |  | OS06g0138100 | | Os06g0138100 | |  | | - | |
| OS06T0138200-01 |  | OS06g0138200 | | Os06g0138200 | |  | | - | |
| OS06T0139900-01 |  | OS06g0139900 | | Os06g0139900 | |  | | - | |
| OS06T0140100-01 |  | OS06g0140100 | | Os06g0140100 | |  | | - | |
| OS06T0141166-00 |  | OS06g0141166 | | Os06g0141166 | |  | | - | |
| OS06T0142700-01 |  | OS06g0142700 | | Os06g0142700 | |  | | - | |
| OS06T0143900-01 |  | OS06g0143900 | | Os06g0143900 | |  | | - | |
| OS06T0144000-01 |  | OS06g0144000 | | Os06g0144000 | |  | | - | |
| OS06T0144800-01 |  | OS06g0144800 | | Os06g0144800 | |  | | - | |
| OS06T0145800-00 |  | OS06g0145800 | | Os06g0145800 | | Down | | -Down | |
| OS06T0146300-01 |  | OS06g0146300 | | Os06g0146300 | |  | | - | |
| OS06T0147800-01 |  | OS06g0147800 | | Os06g0147800 | |  | | - | |
| OS06T0149400-01 |  | OS06g0149400 | | Os06g0149400 | |  | | - | |
| OS06T0149900-01 |  | OS06g0149900 | | Os06g0149900 | |  | | - | |
| OS06T0150100-01 |  | OS06g0150100 | | Os06g0150100 | |  | | - | |
| OS06T0150400-01 |  | OS06g0150400 | | Os06g0150400 | |  | | - | |
| OS06T0151100-00 |  | OS06g0151100 | | Os06g0151100 | |  | | - | |
| OS06T0151900-01 |  | OS06g0151900 | | Os06g0151900 | |  | | - | |
| OS06T0152100-01 |  | OS06g0152100 | | Os06g0152100 | |  | | - | |
| OS06T0152300-01 |  | OS06g0152300 | | Os06g0152300 | |  | | - | |
| OS06T0153800-01 |  | OS06g0153800 | | Os06g0153800 | |  | | - | |
| OS06T0154500-01 |  | OS06g0154500 | | Os06g0154500 | |  | | - | |
| OS06T0154600-00 |  | OS06g0154600 | | Os06g0154600 | |  | | - | |
| OS06T0155600-01 |  | OS06g0155600 | | Os06g0155600 | |  | | - | |
| OS06T0156600-01 |  | OS06g0156600 | | Os06g0156600 | |  | | - | |
| OS06T0157000-01 |  | OS06g0157000 | | Os06g0157000 | |  | | - | |
| OS06T0157800-01 |  | OS06g0157800 | | Os06g0157800 | |  | | - | |
| OS06T0158000-01 |  | OS06g0158000 | | Os06g0158000 | |  | | - | |
| OS06T0158900-00 |  | OS06g0158900 | | Os06g0158900 | |  | | - | |
| OS06T0159725-00 |  | OS06g0159725 | | Os06g0159725 | |  | | - | |
| OS06T0160400-01 |  | OS06g0160400 | | Os06g0160400 | |  | | - | |
| OS06T0160700-01 |  | OS06g0160700 | | Os06g0160700 | | Up | | -Up | |
| OS06T0163200-01 |  | OS06g0163200 | | Os06g0163200 | |  | | - | |
| OS06T0163600-01 |  | OS06g0163600 | | Os06g0163600 | |  | | - | |
| OS06T0164100-02 |  | OS06g0164100 | | Os06g0164100 | |  | | - | |
| OS06T0165800-01 |  | OS06g0165800 | | Os06g0165800 | |  | | - | |
| OS06T0166100-01 |  | OS06g0166100 | | Os06g0166100 | |  | | - | |
| OS06T0167000-01 |  | OS06g0167000 | | Os06g0167000 | |  | | - | |
| OS06T0167000-02 |  | OS06g0167000 | | Os06g0167000 | |  | | - | |
| OS06T0167100-01 |  | OS06g0167100 | | Os06g0167100 | |  | | - | |
| OS06T0167600-01 |  | OS06g0167600 | | Os06g0167600 | |  | | - | |
| OS06T0168000-01 |  | OS06g0168000 | | Os06g0168000 | |  | | - | |
| OS06T0168500-01 |  | OS06g0168500 | | Os06g0168500 | |  | | - | |
| OS06T0170500-01 |  | OS06g0170500 | | Os06g0170500 | |  | | - | |
| OS06T0172600-00 |  | OS06g0172600 | | Os06g0172600 | |  | | - | |
| OS06T0172800-00 | Up | OS06g0172800 | | Os06g0172800 | |  | | Up- | |
| OS06T0173100-01 |  | OS06g0173100 | | Os06g0173100 | |  | | - | |
| OS06T0174300-01 |  | OS06g0174300 | | Os06g0174300 | | Up | | -Up | |
| OS06T0174400-01 |  | OS06g0174400 | | Os06g0174400 | |  | | - | |
| OS06T0175500-01 |  | OS06g0175500 | | Os06g0175500 | |  | | - | |
| OS06T0176900-00 |  | OS06g0176900 | | Os06g0176900 | |  | | - | |
| OS06T0179000-01 |  | OS06g0179000 | | Os06g0179000 | | Down | | -Down | |
| OS06T0181100-01 |  | OS06g0181100 | | Os06g0181100 | |  | | - | |
| OS06T0182400-02 |  | OS06g0182400 | | Os06g0182400 | |  | | - | |
| OS06T0183900-01 |  | OS06g0183900 | | Os06g0183900 | |  | | - | |
| OS06T0185900-01 | Down | OS06g0185900 | | Os06g0185900 | |  | | Down- | |
| OS06T0186400-01 |  | OS06g0186400 | | Os06g0186400 | |  | | - | |
| OS06T0186900-01 | Up | OS06g0186900 | | Os06g0186900 | |  | | Up- | |
| OS06T0187900-02 |  | OS06g0187900 | | Os06g0187900 | |  | | - | |
| OS06T0189600-01 |  | OS06g0189600 | | Os06g0189600 | |  | | - | |
| OS06T0189600-05 |  | OS06g0189600 | | Os06g0189600 | |  | | - | |
| OS06T0191300-02 |  | OS06g0191300 | | Os06g0191300 | |  | | - | |
| OS06T0192600-01 |  | OS06g0192600 | | Os06g0192600 | |  | | - | |
| OS06T0194900-03 | Up | OS06g0194900 | | Os06g0194900 | |  | | Up- | |
| OS06T0196300-02 |  | OS06g0196300 | | Os06g0196300 | |  | | - | |
| OS06T0196600-01 |  | OS06g0196600 | | Os06g0196600 | |  | | - | |
| OS06T0196900-03 |  | OS06g0196900 | | Os06g0196900 | |  | | - | |
| OS06T0199200-06 |  | OS06g0199200 | | Os06g0199200 | |  | | - | |
| OS06T0199500-02 |  | OS06g0199500 | | Os06g0199500 | |  | | - | |
| OS06T0206100-02 |  | OS06g0206100 | | Os06g0206100 | |  | | - | |
| OS06T0208300-01 |  | OS06g0208300 | | Os06g0208300 | |  | | - | |
| OS06T0210500-01 |  | OS06g0210500 | | Os06g0210500 | |  | | - | |
| OS06T0211300-01 |  | OS06g0211300 | | Os06g0211300 | |  | | - | |
| OS06T0213200-01 |  | OS06g0213200 | | Os06g0213200 | |  | | - | |
| OS06T0214300-01 |  | OS06g0214300 | | Os06g0214300 | |  | | - | |
| OS06T0214800-02 |  | OS06g0214800 | | Os06g0214800 | | Up | | -Up | |
| OS06T0214850-00 | Up | OS06g0214850 | | Os06g0214850 | |  | | Up- | |
| OS06T0215300-01 |  | OS06g0215300 | | Os06g0215300 | |  | | - | |
| OS06T0215400-01 |  | OS06g0215400 | | Os06g0215400 | |  | | - | |
| OS06T0215900-01 |  | OS06g0215900 | | Os06g0215900 | | Up | | -Up | |
| OS06T0216700-01 |  | OS06g0216700 | | Os06g0216700 | | Up | | -Up | |
| OS06T0217500-01 |  | OS06g0217500 | | Os06g0217500 | |  | | - | |
| OS06T0217600-01 |  | OS06g0217600 | | Os06g0217600 | |  | | - | |
| OS06T0219600-01 |  | OS06g0219600 | | Os06g0219600 | |  | | - | |
| OS06T0220000-01 |  | OS06g0220000 | | Os06g0220000 | |  | | - | |
| OS06T0221200-01 |  | OS06g0221200 | | Os06g0221200 | |  | | - | |
| OS06T0225900-01 |  | OS06g0225900 | | Os06g0225900 | |  | | - | |
| OS06T0226600-02 |  | OS06g0226600 | | Os06g0226600 | |  | | - | |
| OS06T0227200-02 |  | OS06g0227200 | | Os06g0227200 | |  | | - | |
| OS06T0229300-01 |  | OS06g0229300 | | Os06g0229300 | |  | | - | |
| OS06T0231300-01 |  | OS06g0231300 | | Os06g0231300 | |  | | - | |
| OS06T0232000-03 |  | OS06g0232000 | | Os06g0232000 | |  | | - | |
| OS06T0232600-01 | Up | OS06g0232600 | | Os06g0232600 | |  | | Up- | |
| OS06T0234100-01 |  | OS06g0234100 | | Os06g0234100 | |  | | - | |
| OS06T0237300-01 |  | OS06g0237300 | | Os06g0237300 | |  | | - | |
| OS06T0237502-00 |  | OS06g0237502 | | Os06g0237502 | |  | | - | |
| OS06T0238300-01 |  | OS06g0238300 | | Os06g0238300 | |  | | - | |
| OS06T0245700-00 |  | OS06g0245700 | | Os06g0245700 | |  | | - | |
| OS06T0245800-01 | Down | OS06g0245800 | | Os06g0245800 | |  | | Down- | |
| OS06T0246000-01 | Up | OS06g0246000 | | Os06g0246000 | |  | | Up- | |
| OS06T0246500-01 |  | OS06g0246500 | | Os06g0246500 | | Up | | -Up | |
| OS06T0247500-01 |  | OS06g0247500 | | Os06g0247500 | |  | | - | |
| OS06T0247800-01 |  | OS06g0247800 | | Os06g0247800 | |  | | - | |
| OS06T0253100-01 |  | OS06g0253100 | | Os06g0253100 | |  | | - | |
| OS06T0254300-02 | Up | OS06g0254300 | | Os06g0254300 | |  | | Up- | |
| OS06T0254700-01 |  | OS06g0254700 | | Os06g0254700 | |  | | - | |
| OS06T0255100-01 |  | OS06g0255100 | | Os06g0255100 | |  | | - | |
| OS06T0255200-01 |  | OS06g0255200 | | Os06g0255200 | |  | | - | |
| OS06T0256200-01 | Up | OS06g0256200 | | Os06g0256200 | |  | | Up- | |
| OS06T0256500-01 | Down | OS06g0256500 | | Os06g0256500 | |  | | Down- | |
| OS06T0257200-01 |  | OS06g0257200 | | Os06g0257200 | |  | | - | |
| OS06T0258900-01 |  | OS06g0258900 | | Os06g0258900 | |  | | - | |
| OS06T0264300-02 |  | OS06g0264300 | | Os06g0264300 | |  | | - | |
| OS06T0264700-01 |  | OS06g0264700 | | Os06g0264700 | |  | | - | |
| OS06T0264800-01 |  | OS06g0264800 | | Os06g0264800 | |  | | - | |
| OS06T0265000-01 |  | OS06g0265000 | | Os06g0265000 | |  | | - | |
| OS06T0270900-01 |  | OS06g0270900 | | Os06g0270900 | |  | | - | |
| OS06T0273800-02 |  | OS06g0273800 | | Os06g0273800 | |  | | - | |
| OS06T0274000-01 |  | OS06g0274000 | | Os06g0274000 | |  | | - | |
| OS06T0281400-01 |  | OS06g0281400 | | Os06g0281400 | |  | | - | |
| OS06T0286351-01 |  | OS06g0286351 | | Os06g0286351 | |  | | - | |
| OS06T0288100-01 |  | OS06g0288100 | | Os06g0288100 | |  | | - | |
| OS06T0288300-01 |  | OS06g0288300 | | Os06g0288300 | |  | | - | |
| OS06T0298500-01 |  | OS06g0298500 | | Os06g0298500 | |  | | - | |
| OS06T0300600-01 |  | OS06g0300600 | | Os06g0300600 | |  | | - | |
| OS06T0300700-01 |  | OS06g0300700 | | Os06g0300700 | |  | | - | |
| OS06T0300800-01 |  | OS06g0300800 | | Os06g0300800 | |  | | - | |
| OS06T0301000-01 |  | OS06g0301000 | | Os06g0301000 | |  | | - | |
| OS06T0301300-00 |  | OS06g0301300 | | Os06g0301300 | |  | | - | |
| OS06T0303400-00 |  | OS06g0303400 | | Os06g0303400 | |  | | - | |
| OS06T0304600-02 |  | OS06g0304600 | | Os06g0304600 | |  | | - | |
| OS06T0305200-01 |  | OS06g0305200 | | Os06g0305200 | |  | | - | |
| OS06T0306300-01 |  | OS06g0306300 | | Os06g0306300 | | Down | | -Down | |
| OS06T0306600-02 |  | OS06g0306600 | | Os06g0306600 | |  | | - | |
| OS06T0308000-01 |  | OS06g0308000 | | Os06g0308000 | |  | | - | |
| OS06T0308100-01 |  | OS06g0308100 | | Os06g0308100 | |  | | - | |
| OS06T0308800-01 |  | OS06g0308800 | | Os06g0308800 | |  | | - | |
| OS06T0318200-01 |  | OS06g0318200 | | Os06g0318200 | |  | | - | |
| OS06T0318600-01 |  | OS06g0318600 | | Os06g0318600 | | Up | | -Up | |
| OS06T0319700-01 |  | OS06g0319700 | | Os06g0319700 | |  | | - | |
| OS06T0319800-00 |  | OS06g0319800 | | Os06g0319800 | |  | | - | |
| OS06T0320000-01 |  | OS06g0320000 | | Os06g0320000 | |  | | - | |
| OS06T0320500-01 |  | OS06g0320500 | | Os06g0320500 | |  | | - | |
| OS06T0320700-01 |  | OS06g0320700 | | Os06g0320700 | |  | | - | |
| OS06T0325500-02 | Up | OS06g0325500 | | Os06g0325500 | |  | | Up- | |
| OS06T0326400-01 | Up | OS06g0326400 | | Os06g0326400 | |  | | Up- | |
| OS06T0326500-01 |  | OS06g0326500 | | Os06g0326500 | |  | | - | |
| OS06T0332400-02 |  | OS06g0332400 | | Os06g0332400 | |  | | - | |
| OS06T0332800-01 |  | OS06g0332800 | | Os06g0332800 | |  | | - | |
| OS06T0334400-01 |  | OS06g0334400 | | Os06g0334400 | |  | | - | |
| OS06T0336200-02 |  | OS06g0336200 | | Os06g0336200 | | Up | | -Up | |
| OS06T0338900-01 |  | OS06g0338900 | | Os06g0338900 | |  | | - | |
| OS06T0342100-01 |  | OS06g0342100 | | Os06g0342100 | |  | | - | |
| OS06T0342200-01 |  | OS06g0342200 | | Os06g0342200 | |  | | - | |
| OS06T0342500-01 |  | OS06g0342500 | | Os06g0342500 | |  | | - | |
| OS06T0343600-01 |  | OS06g0343600 | | Os06g0343600 | |  | | - | |
| OS06T0354500-01 |  | OS06g0354500 | | Os06g0354500 | |  | | - | |
| OS06T0355500-01 |  | OS06g0355500 | | Os06g0355500 | |  | | - | |
| OS06T0360300-02 | Up | OS06g0360300 | | Os06g0360300 | |  | | Up- | |
| OS06T0360600-02 |  | OS06g0360600 | | Os06g0360600 | |  | | - | |
| OS06T0367100-02 |  | OS06g0367100 | | Os06g0367100 | | Down | | -Down | |
| OS06T0472000-01 |  | OS06g0472000 | | Os06g0472000 | |  | | - | |
| OS06T0472200-02 |  | OS06g0472200 | | Os06g0472200 | |  | | - | |
| OS06T0472400-01 |  | OS06g0472400 | | Os06g0472400 | |  | | - | |
| OS06T0472700-02 |  | OS06g0472700 | | Os06g0472700 | |  | | - | |
| OS06T0476200-01 |  | OS06g0476200 | | Os06g0476200 | |  | | - | |
| OS06T0484500-01 |  | OS06g0484500 | | Os06g0484500 | | Down | | -Down | |
| OS06T0484600-01 |  | OS06g0484600 | | Os06g0484600 | |  | | - | |
| OS06T0485100-03 |  | OS06g0485100 | | Os06g0485100 | |  | | - | |
| OS06T0486800-01 |  | OS06g0486800 | | Os06g0486800 | |  | | - | |
| OS06T0489200-01 | Down | OS06g0489200 | | Os06g0489200 | |  | | Down- | |
| OS06T0496000-01 |  | OS06g0496000 | | Os06g0496000 | |  | | - | |
| OS06T0498400-01 |  | OS06g0498400 | | Os06g0498400 | |  | | - | |
| OS06T0498900-01 |  | OS06g0498900 | | Os06g0498900 | |  | | - | |
| OS06T0499000-01 |  | OS06g0499000 | | Os06g0499000 | |  | | - | |
| OS06T0499301-00 |  | OS06g0499301 | | Os06g0499301 | |  | | - | |
| OS06T0499900-01 |  | OS06g0499900 | | Os06g0499900 | |  | | - | |
| OS06T0503400-01 |  | OS06g0503400 | | Os06g0503400 | |  | | - | |
| OS06T0508700-01 |  | OS06g0508700 | | Os06g0508700 | |  | | - | |
| OS06T0528600-01 |  | OS06g0528600 | | Os06g0528600 | |  | | - | |
| OS06T0530200-02 |  | OS06g0530200 | | Os06g0530200 | |  | | - | |
| OS06T0531200-02 |  | OS06g0531200 | | Os06g0531200 | |  | | - | |
| OS06T0531900-01 |  | OS06g0531900 | | Os06g0531900 | |  | | - | |
| OS06T0538000-01 |  | OS06g0538000 | | Os06g0538000 | |  | | - | |
| OS06T0538200-01 |  | OS06g0538200 | | Os06g0538200 | |  | | - | |
| OS06T0547400-01 |  | OS06g0547400 | | Os06g0547400 | |  | | - | |
| OS06T0548000-01 |  | OS06g0548000 | | Os06g0548000 | |  | | - | |
| OS06T0548200-01 | Up | OS06g0548200 | | Os06g0548200 | |  | | Up- | |
| OS06T0551400-01 |  | OS06g0551400 | | Os06g0551400 | |  | | - | |
| OS06T0555400-01 | Down | OS06g0555400 | | Os06g0555400 | |  | | Down- | |
| OS06T0557100-02 |  | OS06g0557100 | | Os06g0557100 | |  | | - | |
| OS06T0559500-03 | Up | OS06g0559500 | | Os06g0559500 | |  | | Up- | |
| OS06T0562600-01 |  | OS06g0562600 | | Os06g0562600 | |  | | - | |
| OS06T0562700-01 |  | OS06g0562700 | | Os06g0562700 | |  | | - | |
| OS06T0563300-01 |  | OS06g0563300 | | Os06g0563300 | |  | | - | |
| OS06T0564500-01 |  | OS06g0564500 | | Os06g0564500 | |  | | - | |
| OS06T0566100-01 |  | OS06g0566100 | | Os06g0566100 | |  | | - | |
| OS06T0567900-01 |  | OS06g0567900 | | Os06g0567900 | |  | | - | |
| OS06T0568200-01 |  | OS06g0568200 | | Os06g0568200 | |  | | - | |
| OS06T0571100-01 |  | OS06g0571100 | | Os06g0571100 | |  | | - | |
| OS06T0571400-02 |  | OS06g0571400 | | Os06g0571400 | |  | | - | |
| OS06T0573600-01 |  | OS06g0573600 | | Os06g0573600 | | Down | | -Down | |
| OS06T0578700-01 |  | OS06g0578700 | | Os06g0578700 | |  | | - | |
| OS06T0581500-01 |  | OS06g0581500 | | Os06g0581500 | |  | | - | |
| OS06T0582600-01 |  | OS06g0582600 | | Os06g0582600 | |  | | - | |
| OS06T0583400-02 |  | OS06g0583400 | | Os06g0583400 | |  | | - | |
| OS06T0584200-03 |  | OS06g0584200 | | Os06g0584200 | |  | | - | |
| OS06T0590301-01 | Up | OS06g0590301 | | Os06g0590301 | |  | | Up- | |
| OS06T0590800-00 |  | OS06g0590800 | | Os06g0590800 | |  | | - | |
| OS06T0593800-01 | Up | OS06g0593800 | | Os06g0593800 | | Up | | Up-Up | |
| OS06T0594100-01 |  | OS06g0594100 | | Os06g0594100 | |  | | - | |
| OS06T0597600-01 |  | OS06g0597600 | | Os06g0597600 | |  | | - | |
| OS06T0597900-01 |  | OS06g0597900 | | Os06g0597900 | |  | | - | |
| OS06T0598900-01 |  | OS06g0598900 | | Os06g0598900 | |  | | - | |
| OS06T0600100-01 |  | OS06g0600100 | | Os06g0600100 | |  | | - | |
| OS06T0601000-01 |  | OS06g0601000 | | Os06g0601000 | |  | | - | |
| OS06T0601100-02 |  | OS06g0601100 | | Os06g0601100 | |  | | - | |
| OS06T0603000-01 |  | OS06g0603000 | | Os06g0603000 | | Up | | -Up | |
| OS06T0603600-02 |  | OS06g0603600 | | Os06g0603600 | | Up | | -Up | |
| OS06T0604200-02 |  | OS06g0604200 | | Os06g0604200 | |  | | - | |
| OS06T0604300-01 |  | OS06g0604300 | | Os06g0604300 | |  | | - | |
| OS06T0604400-01 |  | OS06g0604400 | | Os06g0604400 | |  | | - | |
| OS06T0604500-01 |  | OS06g0604500 | | Os06g0604500 | |  | | - | |
| OS06T0606900-01 |  | OS06g0606900 | | Os06g0606900 | |  | | - | |
| OS06T0607200-01 |  | OS06g0607200 | | Os06g0607200 | |  | | - | |
| OS06T0608300-01 |  | OS06g0608300 | | Os06g0608300 | |  | | - | |
| OS06T0608500-01 |  | OS06g0608500 | | Os06g0608500 | |  | | - | |
| OS06T0608700-02 |  | OS06g0608700 | | Os06g0608700 | |  | | - | |
| OS06T0608800-03 |  | OS06g0608800 | | Os06g0608800 | |  | | - | |
| OS06T0610800-01 |  | OS06g0610800 | | Os06g0610800 | |  | | - | |
| OS06T0611900-01 |  | OS06g0611900 | | Os06g0611900 | |  | | - | |
| OS06T0618100-01 |  | OS06g0618100 | | Os06g0618100 | |  | | - | |
| OS06T0620200-01 |  | OS06g0620200 | | Os06g0620200 | |  | | - | |
| OS06T0620550-00 |  | OS06g0620550 | | Os06g0620550 | |  | | - | |
| OS06T0622300-03 |  | OS06g0622300 | | Os06g0622300 | |  | | - | |
| OS06T0622900-01 |  | OS06g0622900 | | Os06g0622900 | |  | | - | |
| OS06T0623300-01 | Up | OS06g0623300 | | Os06g0623300 | |  | | Up- | |
| OS06T0625400-01 |  | OS06g0625400 | | Os06g0625400 | |  | | - | |
| OS06T0625500-03 | Up | OS06g0625500 | | Os06g0625500 | |  | | Up- | |
| OS06T0632200-01 | Up | OS06g0632200 | | Os06g0632200 | |  | | Up- | |
| OS06T0632700-01 |  | OS06g0632700 | | Os06g0632700 | |  | | - | |
| OS06T0634300-02 |  | OS06g0634300 | | Os06g0634300 | |  | | - | |
| OS06T0634600-01 |  | OS06g0634600 | | Os06g0634600 | |  | | - | |
| OS06T0635200-01 |  | OS06g0635200 | | Os06g0635200 | |  | | - | |
| OS06T0638200-01 |  | OS06g0638200 | | Os06g0638200 | |  | | - | |
| OS06T0639500-01 |  | OS06g0639500 | | Os06g0639500 | |  | | - | |
| OS06T0643100-01 |  | OS06g0643100 | | Os06g0643100 | |  | | - | |
| OS06T0643300-02 |  | OS06g0643300 | | Os06g0643300 | |  | | - | |
| OS06T0643600-01 |  | OS06g0643600 | | Os06g0643600 | |  | | - | |
| OS06T0643900-01 |  | OS06g0643900 | | Os06g0643900 | |  | | - | |
| OS06T0644100-01 |  | OS06g0644100 | | Os06g0644100 | |  | | - | |
| OS06T0644200-01 | Up | OS06g0644200 | | Os06g0644200 | |  | | Up- | |
| OS06T0644800-01 |  | OS06g0644800 | | Os06g0644800 | |  | | - | |
| OS06T0645400-01 |  | OS06g0645400 | | Os06g0645400 | |  | | - | |
| OS06T0645400-02 |  | OS06g0645400 | | Os06g0645400 | |  | | - | |
| OS06T0646000-02 | Up | OS06g0646000 | | Os06g0646000 | |  | | Up- | |
| OS06T0646500-01 | Up | OS06g0646500 | | Os06g0646500 | |  | | Up- | |
| OS06T0646600-02 |  | OS06g0646600 | | Os06g0646600 | |  | | - | |
| OS06T0647100-01 | Down | OS06g0647100 | | Os06g0647100 | |  | | Down- | |
| OS06T0647400-02 |  | OS06g0647400 | | Os06g0647400 | |  | | - | |
| OS06T0649500-01 |  | OS06g0649500 | | Os06g0649500 | |  | | - | |
| OS06T0649900-01 |  | OS06g0649900 | | Os06g0649900 | | Up | | -Up | |
| OS06T0651000-01 |  | OS06g0651000 | | Os06g0651000 | |  | | - | |
| OS06T0652400-02 | Up | OS06g0652400 | | Os06g0652400 | |  | | Up- | |
| OS06T0653800-01 |  | OS06g0653800 | | Os06g0653800 | |  | | - | |
| OS06T0655100-00 |  | OS06g0655100 | | Os06g0655100 | |  | | - | |
| OS06T0659300-01 |  | OS06g0659300 | | Os06g0659300 | |  | | - | |
| OS06T0661800-02 |  | OS06g0661800 | | Os06g0661800 | |  | | - | |
| OS06T0662000-01 |  | OS06g0662000 | | Os06g0662000 | |  | | - | |
| OS06T0663800-01 |  | OS06g0663800 | | Os06g0663800 | |  | | - | |
| OS06T0664100-01 |  | OS06g0664100 | | Os06g0664100 | |  | | - | |
| OS06T0664200-01 |  | OS06g0664200 | | Os06g0664200 | | Down | | -Down | |
| OS06T0664300-01 |  | OS06g0664300 | | Os06g0664300 | |  | | - | |
| OS06T0664400-01 |  | OS06g0664400 | | Os06g0664400 | |  | | - | |
| OS06T0665000-02 |  | OS06g0665000 | | Os06g0665000 | |  | | - | |
| OS06T0665100-02 |  | OS06g0665100 | | Os06g0665100 | |  | | - | |
| OS06T0665900-00 |  | OS06g0665900 | | Os06g0665900 | |  | | - | |
| OS06T0666400-01 |  | OS06g0666400 | | Os06g0666400 | |  | | - | |
| OS06T0667500-01 |  | OS06g0667500 | | Os06g0667500 | |  | | - | |
| OS06T0668200-01 | Up | OS06g0668200 | | Os06g0668200 | | Up | | Up-Up | |
| OS06T0669400-02 |  | OS06g0669400 | | Os06g0669400 | |  | | - | |
| OS06T0670500-01 |  | OS06g0670500 | | Os06g0670500 | |  | | - | |
| OS06T0671700-01 |  | OS06g0671700 | | Os06g0671700 | | Down | | -Down | |
| OS06T0671900-01 |  | OS06g0671900 | | Os06g0671900 | |  | | - | |
| OS06T0675700-01 |  | OS06g0675700 | | Os06g0675700 | | Down | | -Down | |
| OS06T0676700-02 |  | OS06g0676700 | | Os06g0676700 | |  | | - | |
| OS06T0677400-02 |  | OS06g0677400 | | Os06g0677400 | |  | | - | |
| OS06T0677700-00 |  | OS06g0677700 | | Os06g0677700 | |  | | - | |
| OS06T0681200-01 |  | OS06g0681200 | | Os06g0681200 | | Up | | -Up | |
| OS06T0681600-01 |  | OS06g0681600 | | Os06g0681600 | | Down | | -Down | |
| OS06T0682700-00 |  | OS06g0682700 | | Os06g0682700 | |  | | - | |
| OS06T0683200-01 |  | OS06g0683200 | | Os06g0683200 | | Down | | -Down | |
| OS06T0684000-02 |  | OS06g0684000 | | Os06g0684000 | |  | | - | |
| OS06T0686400-02 |  | OS06g0686400 | | Os06g0686400 | |  | | - | |
| OS06T0687700-01 |  | OS06g0687700 | | Os06g0687700 | |  | | - | |
| OS06T0687800-01 |  | OS06g0687800 | | Os06g0687800 | |  | | - | |
| OS06T0690700-01 |  | OS06g0690700 | | Os06g0690700 | |  | | - | |
| OS06T0694000-01 |  | OS06g0694000 | | Os06g0694000 | |  | | - | |
| OS06T0694500-01 |  | OS06g0694500 | | Os06g0694500 | |  | | - | |
| OS06T0694800-01 |  | OS06g0694800 | | Os06g0694800 | |  | | - | |
| OS06T0695500-01 |  | OS06g0695500 | | Os06g0695500 | |  | | - | |
| OS06T0696400-02 |  | OS06g0696400 | | Os06g0696400 | |  | | - | |
| OS06T0698859-01 |  | OS06g0698859 | | Os06g0698859 | |  | | - | |
| OS06T0698900-01 |  | OS06g0698900 | | Os06g0698900 | |  | | - | |
| OS06T0699400-01 |  | OS06g0699400 | | Os06g0699400 | |  | | - | |
| OS06T0699500-02 |  | OS06g0699500 | | Os06g0699500 | |  | | - | |
| OS06T0699900-01 |  | OS06g0699900 | | Os06g0699900 | |  | | - | |
| OS06T0701100-03 |  | OS06g0701100 | | Os06g0701100 | |  | | - | |
| OS06T0701200-01 |  | OS06g0701200 | | Os06g0701200 | |  | | - | |
| OS06T0701300-01 |  | OS06g0701300 | | Os06g0701300 | |  | | - | |
| OS06T0701400-01 | Down | OS06g0701400 | | Os06g0701400 | |  | | Down- | |
| OS06T0702500-01 |  | OS06g0702500 | | Os06g0702500 | |  | | - | |
| OS06T0704400-02 |  | OS06g0704400 | | Os06g0704400 | |  | | - | |
| OS06T0704600-02 |  | OS06g0704600 | | Os06g0704600 | |  | | - | |
| OS06T0704700-01 | Down | OS06g0704700 | | Os06g0704700 | |  | | Down- | |
| OS06T0705100-03 |  | OS06g0705100 | | Os06g0705100 | | Down | | -Down | |
| OS06T0708300-01 |  | OS06g0708300 | | Os06g0708300 | |  | | - | |
| OS06T0708500-02 | Up | OS06g0708500 | | Os06g0708500 | |  | | Up- | |
| OS06T0709000-01 |  | OS06g0709000 | | Os06g0709000 | |  | | - | |
| OS06T0710700-01 |  | OS06g0710700 | | Os06g0710700 | | Up | | -Up | |
| OS06T0711100-02 |  | OS06g0711100 | | Os06g0711100 | |  | | - | |
| OS06T0711600-02 |  | OS06g0711600 | | Os06g0711600 | |  | | - | |
| OS06T0712200-01 |  | OS06g0712200 | | Os06g0712200 | |  | | - | |
| OS06T0712400-01 |  | OS06g0712400 | | Os06g0712400 | |  | | - | |
| OS06T0714200-01 |  | OS06g0714200 | | Os06g0714200 | |  | | - | |
| OS06T0715200-01 |  | OS06g0715200 | | Os06g0715200 | |  | | - | |
| OS06T0715600-02 |  | OS06g0715600 | | Os06g0715600 | |  | | - | |
| OS06T0716000-02 |  | OS06g0716000 | | Os06g0716000 | |  | | - | |
| OS06T0716700-02 |  | OS06g0716700 | | Os06g0716700 | |  | | - | |
| OS06T0717100-01 |  | OS06g0717100 | | Os06g0717100 | |  | | - | |
| OS06T0717900-02 |  | OS06g0717900 | | Os06g0717900 | |  | | - | |
| OS06T0724100-02 |  | OS06g0724100 | | Os06g0724100 | |  | | - | |
| OS06T0724200-01 |  | OS06g0724200 | | Os06g0724200 | |  | | - | |
| OS06T0724400-01 |  | OS06g0724400 | | Os06g0724400 | | Up | | -Up | |
| OS06T0724600-01 |  | OS06g0724600 | | Os06g0724600 | |  | | - | |
| OS06T0724900-01 |  | OS06g0724900 | | Os06g0724900 | |  | | - | |
| OS06T0725900-01 |  | OS06g0725900 | | Os06g0725900 | |  | | - | |
| OS06T0726200-02 |  | OS06g0726200 | | Os06g0726200 | | Down | | -Down | |
| OS06T0726400-04 | Up | OS06g0726400 | | Os06g0726400 | |  | | Up- | |
| OS06T0727200-02 |  | OS06g0727200 | | Os06g0727200 | |  | | - | |
| OS06T0728000-02 |  | OS06g0728000 | | Os06g0728000 | |  | | - | |
| OS06T0728600-00 |  | OS06g0728600 | | Os06g0728600 | |  | | - | |
| OS06T0729650-00 | Down | OS06g0729650 | | Os06g0729650 | |  | | Down- | |
| OS06T0730600-01 |  | OS06g0730600 | | Os06g0730600 | |  | | - | |
| OS06T0730800-01 |  | OS06g0730800 | | Os06g0730800 | |  | | - | |
| OS06T0731800-01 |  | OS06g0731800 | | Os06g0731800 | |  | | - | |
| OS07T0100200-01 |  | OS07g0100200 | | Os07g0100200 | |  | | - | |
| OS07T0100300-03 |  | OS07g0100300 | | Os07g0100300 | |  | | - | |
| OS07T0102300-01 |  | OS07g0102300 | | Os07g0102300 | |  | | - | |
| OS07T0103200-01 |  | OS07g0103200 | | Os07g0103200 | |  | | - | |
| OS07T0105600-01 |  | OS07g0105600 | | Os07g0105600 | |  | | - | |
| OS07T0106200-02 | Up | OS07g0106200 | | Os07g0106200 | |  | | Up- | |
| OS07T0107300-01 |  | OS07g0107300 | | Os07g0107300 | |  | | - | |
| OS07T0108300-01 |  | OS07g0108300 | | Os07g0108300 | |  | | - | |
| OS07T0109500-01 |  | OS07g0109500 | | Os07g0109500 | |  | | - | |
| OS07T0110300-01 |  | OS07g0110300 | | Os07g0110300 | |  | | - | |
| OS07T0111600-01 |  | OS07g0111600 | | Os07g0111600 | |  | | - | |
| OS07T0112800-01 |  | OS07g0112800 | | Os07g0112800 | |  | | - | |
| OS07T0114400-01 |  | OS07g0114400 | | Os07g0114400 | |  | | - | |
| OS07T0115300-01 | Down | OS07g0115300 | | Os07g0115300 | |  | | Down- | |
| OS07T0122400-01 |  | OS07g0122400 | | Os07g0122400 | |  | | - | |
| OS07T0124500-01 |  | OS07g0124500 | | Os07g0124500 | |  | | - | |
| OS07T0133700-01 |  | OS07g0133700 | | Os07g0133700 | |  | | - | |
| OS07T0134800-01 |  | OS07g0134800 | | Os07g0134800 | |  | | - | |
| OS07T0137900-01 |  | OS07g0137900 | | Os07g0137900 | |  | | - | |
| OS07T0137900-02 |  | OS07g0137900 | | Os07g0137900 | |  | | - | |
| OS07T0141400-02 | Down | OS07g0141400 | | Os07g0141400 | |  | | Down- | |
| OS07T0142000-01 |  | OS07g0142000 | | Os07g0142000 | |  | | - | |
| OS07T0142900-01 |  | OS07g0142900 | | Os07g0142900 | |  | | - | |
| OS07T0143000-01 | Up | OS07g0143000 | | Os07g0143000 | |  | | Up- | |
| OS07T0143600-01 |  | OS07g0143600 | | Os07g0143600 | | Down | | -Down | |
| OS07T0143700-01 |  | OS07g0143700 | | Os07g0143700 | |  | | - | |
| OS07T0147500-01 |  | OS07g0147500 | | Os07g0147500 | | Down | | -Down | |
| OS07T0147900-01 | Up | OS07g0147900 | | Os07g0147900 | |  | | Up- | |
| OS07T0148900-01 |  | OS07g0148900 | | Os07g0148900 | |  | | - | |
| OS07T0150100-01 |  | OS07g0150100 | | Os07g0150100 | |  | | - | |
| OS07T0150500-01 |  | OS07g0150500 | | Os07g0150500 | |  | | - | |
| OS07T0151200-00 |  | OS07g0151200 | | Os07g0151200 | |  | | - | |
| OS07T0152900-04 |  | OS07g0152900 | | Os07g0152900 | |  | | - | |
| OS07T0158300-01 | Down | OS07g0158300 | | Os07g0158300 | |  | | Down- | |
| OS07T0159800-01 |  | OS07g0159800 | | Os07g0159800 | |  | | - | |
| OS07T0160400-01 |  | OS07g0160400 | | Os07g0160400 | |  | | - | |
| OS07T0162400-01 |  | OS07g0162400 | | Os07g0162400 | |  | | - | |
| OS07T0162500-02 |  | OS07g0162500 | | Os07g0162500 | |  | | - | |
| OS07T0162600-02 | Up | OS07g0162600 | | Os07g0162600 | |  | | Up- | |
| OS07T0162900-01 |  | OS07g0162900 | | Os07g0162900 | | Down | | -Down | |
| OS07T0163000-01 |  | OS07g0163000 | | Os07g0163000 | |  | | - | |
| OS07T0163500-01 |  | OS07g0163500 | | Os07g0163500 | |  | | - | |
| OS07T0163800-01 | Down | OS07g0163800 | | Os07g0163800 | |  | | Down- | |
| OS07T0164100-01 |  | OS07g0164100 | | Os07g0164100 | |  | | - | |
| OS07T0164200-02 |  | OS07g0164200 | | Os07g0164200 | |  | | - | |
| OS07T0164300-01 |  | OS07g0164300 | | Os07g0164300 | |  | | - | |
| OS07T0164900-01 |  | OS07g0164900 | | Os07g0164900 | |  | | - | |
| OS07T0165000-01 |  | OS07g0165000 | | Os07g0165000 | |  | | - | |
| OS07T0167000-02 |  | OS07g0167000 | | Os07g0167000 | |  | | - | |
| OS07T0167100-01 |  | OS07g0167100 | | Os07g0167100 | |  | | - | |
| OS07T0168000-01 |  | OS07g0168000 | | Os07g0168000 | |  | | - | |
| OS07T0168300-01 |  | OS07g0168300 | | Os07g0168300 | |  | | - | |
| OS07T0168600-02 |  | OS07g0168600 | | Os07g0168600 | |  | | - | |
| OS07T0169600-01 |  | OS07g0169600 | | Os07g0169600 | | Up | | -Up | |
| OS07T0170100-02 | Up | OS07g0170100 | | Os07g0170100 | |  | | Up- | |
| OS07T0171100-02 | Down | OS07g0171100 | | Os07g0171100 | |  | | Down- | |
| OS07T0171200-01 |  | OS07g0171200 | | Os07g0171200 | |  | | - | |
| OS07T0172500-01 |  | OS07g0172500 | | Os07g0172500 | |  | | - | |
| OS07T0173501-00 |  | OS07g0173501 | | Os07g0173501 | |  | | - | |
| OS07T0175600-01 | Up | OS07g0175600 | | Os07g0175600 | |  | | Up- | |
| OS07T0176500-01 |  | OS07g0176500 | | Os07g0176500 | |  | | - | |
| OS07T0176900-02 |  | OS07g0176900 | | Os07g0176900 | |  | | - | |
| OS07T0179200-01 |  | OS07g0179200 | | Os07g0179200 | |  | | - | |
| OS07T0179400-01 |  | OS07g0179400 | | Os07g0179400 | |  | | - | |
| OS07T0180900-01 |  | OS07g0180900 | | Os07g0180900 | |  | | - | |
| OS07T0181000-03 | Up | OS07g0181000 | | Os07g0181000 | |  | | Up- | |
| OS07T0182100-01 |  | OS07g0182100 | | Os07g0182100 | |  | | - | |
| OS07T0184800-01 |  | OS07g0184800 | | Os07g0184800 | |  | | - | |
| OS07T0185300-01 |  | OS07g0185300 | | Os07g0185300 | |  | | - | |
| OS07T0185800-01 |  | OS07g0185800 | | Os07g0185800 | |  | | - | |
| OS07T0186000-02 | Up | OS07g0186000 | | Os07g0186000 | |  | | Up- | |
| OS07T0186400-01 | Up | OS07g0186400 | | Os07g0186400 | |  | | Up- | |
| OS07T0187700-01 |  | OS07g0187700 | | Os07g0187700 | |  | | - | |
| OS07T0188800-01 |  | OS07g0188800 | | Os07g0188800 | |  | | - | |
| OS07T0189800-01 | Up | OS07g0189800 | | Os07g0189800 | |  | | Up- | |
| OS07T0190800-01 |  | OS07g0190800 | | Os07g0190800 | |  | | - | |
| OS07T0191000-01 |  | OS07g0191000 | | Os07g0191000 | |  | | - | |
| OS07T0191200-02 |  | OS07g0191200 | | Os07g0191200 | |  | | - | |
| OS07T0191700-01 |  | OS07g0191700 | | Os07g0191700 | |  | | - | |
| OS07T0192300-01 |  | OS07g0192300 | | Os07g0192300 | |  | | - | |
| OS07T0194000-01 |  | OS07g0194000 | | Os07g0194000 | |  | | - | |
| OS07T0195100-02 |  | OS07g0195100 | | Os07g0195100 | |  | | - | |
| OS07T0195400-01 |  | OS07g0195400 | | Os07g0195400 | |  | | - | |
| OS07T0196200-01 | Down | OS07g0196200 | | Os07g0196200 | |  | | Down- | |
| OS07T0198300-01 |  | OS07g0198300 | | Os07g0198300 | |  | | - | |
| OS07T0201100-01 |  | OS07g0201100 | | Os07g0201100 | |  | | - | |
| OS07T0202100-01 |  | OS07g0202100 | | Os07g0202100 | |  | | - | |
| OS07T0202900-01 |  | OS07g0202900 | | Os07g0202900 | |  | | - | |
| OS07T0204100-01 |  | OS07g0204100 | | Os07g0204100 | | Up | | -Up | |
| OS07T0204500-02 |  | OS07g0204500 | | Os07g0204500 | |  | | - | |
| OS07T0204900-01 |  | OS07g0204900 | | Os07g0204900 | |  | | - | |
| OS07T0205000-03 |  | OS07g0205000 | | Os07g0205000 | |  | | - | |
| OS07T0205700-01 |  | OS07g0205700 | | Os07g0205700 | |  | | - | |
| OS07T0206700-02 |  | OS07g0206700 | | Os07g0206700 | |  | | - | |
| OS07T0207400-01 |  | OS07g0207400 | | Os07g0207400 | |  | | - | |
| OS07T0208000-01 |  | OS07g0208000 | | Os07g0208000 | |  | | - | |
| OS07T0208600-01 |  | OS07g0208600 | | Os07g0208600 | |  | | - | |
| OS07T0209000-01 |  | OS07g0209000 | | Os07g0209000 | |  | | - | |
| OS07T0209100-01 |  | OS07g0209100 | | Os07g0209100 | | Up | | -Up | |
| OS07T0211800-02 |  | OS07g0211800 | | Os07g0211800 | |  | | - | |
| OS07T0212200-01 |  | OS07g0212200 | | Os07g0212200 | |  | | - | |
| OS07T0224900-03 |  | OS07g0224900 | | Os07g0224900 | |  | | - | |
| OS07T0227800-00 |  | OS07g0227800 | | Os07g0227800 | |  | | - | |
| OS07T0229900-01 |  | OS07g0229900 | | Os07g0229900 | |  | | - | |
| OS07T0232200-01 |  | OS07g0232200 | | Os07g0232200 | |  | | - | |
| OS07T0236800-01 |  | OS07g0236800 | | Os07g0236800 | |  | | - | |
| OS07T0237100-00 |  | OS07g0237100 | | Os07g0237100 | |  | | - | |
| OS07T0240300-01 |  | OS07g0240300 | | Os07g0240300 | |  | | - | |
| OS07T0243150-01 |  | OS07g0243150 | | Os07g0243150 | | Down | | -Down | |
| OS07T0245100-01 |  | OS07g0245100 | | Os07g0245100 | |  | | - | |
| OS07T0246200-02 |  | OS07g0246200 | | Os07g0246200 | |  | | - | |
| OS07T0247100-01 |  | OS07g0247100 | | Os07g0247100 | |  | | - | |
| OS07T0249700-01 |  | OS07g0249700 | | Os07g0249700 | |  | | - | |
| OS07T0252000-01 |  | OS07g0252000 | | Os07g0252000 | |  | | - | |
| OS07T0260300-01 |  | OS07g0260300 | | Os07g0260300 | |  | | - | |
| OS07T0262200-02 |  | OS07g0262200 | | Os07g0262200 | |  | | - | |
| OS07T0264100-01 |  | OS07g0264100 | | Os07g0264100 | |  | | - | |
| OS07T0271000-01 |  | OS07g0271000 | | Os07g0271000 | | Up | | -Up | |
| OS07T0274700-01 |  | OS07g0274700 | | Os07g0274700 | | Up | | -Up | |
| OS07T0274800-02 |  | OS07g0274800 | | Os07g0274800 | |  | | - | |
| OS07T0275100-00 |  | OS07g0275100 | | Os07g0275100 | | Down | | -Down | |
| OS07T0287100-01 |  | OS07g0287100 | | Os07g0287100 | |  | | - | |
| OS07T0290800-01 |  | OS07g0290800 | | Os07g0290800 | |  | | - | |
| OS07T0295400-02 |  | OS07g0295400 | | Os07g0295400 | |  | | - | |
| OS07T0295800-01 |  | OS07g0295800 | | Os07g0295800 | |  | | - | |
| OS07T0405100-01 |  | OS07g0405100 | | Os07g0405100 | |  | | - | |
| OS07T0406300-02 | Up | OS07g0406300 | | Os07g0406300 | |  | | Up- | |
| OS07T0408100-01 |  | OS07g0408100 | | Os07g0408100 | |  | | - | |
| OS07T0408700-02 |  | OS07g0408700 | | Os07g0408700 | |  | | - | |
| OS07T0409100-01 |  | OS07g0409100 | | Os07g0409100 | |  | | - | |
| OS07T0409200-01 |  | OS07g0409200 | | Os07g0409200 | |  | | - | |
| OS07T0409700-01 |  | OS07g0409700 | | Os07g0409700 | |  | | - | |
| OS07T0410100-01 |  | OS07g0410100 | | Os07g0410100 | |  | | - | |
| OS07T0412100-01 | Down | OS07g0412100 | | Os07g0412100 | | Up | | Down-Up | |
| OS07T0413800-04 |  | OS07g0413800 | | Os07g0413800 | |  | | - | |
| OS07T0418000-02 |  | OS07g0418000 | | Os07g0418000 | |  | | - | |
| OS07T0419200-01 |  | OS07g0419200 | | Os07g0419200 | |  | | - | |
| OS07T0420700-01 |  | OS07g0420700 | | Os07g0420700 | |  | | - | |
| OS07T0421866-00 |  | OS07g0421866 | | Os07g0421866 | |  | | - | |
| OS07T0435100-01 |  | OS07g0435100 | | Os07g0435100 | |  | | - | |
| OS07T0435300-01 |  | OS07g0435300 | | Os07g0435300 | |  | | - | |
| OS07T0438500-01 |  | OS07g0438500 | | Os07g0438500 | |  | | - | |
| OS07T0438800-02 |  | OS07g0438800 | | Os07g0438800 | |  | | - | |
| OS07T0447800-01 |  | OS07g0447800 | | Os07g0447800 | |  | | - | |
| OS07T0448800-01 |  | OS07g0448800 | | Os07g0448800 | | Up | | -Up | |
| OS07T0452700-03 |  | OS07g0452700 | | Os07g0452700 | |  | | - | |
| OS07T0456400-02 |  | OS07g0456400 | | Os07g0456400 | |  | | - | |
| OS07T0456700-01 |  | OS07g0456700 | | Os07g0456700 | |  | | - | |
| OS07T0461600-01 |  | OS07g0461600 | | Os07g0461600 | |  | | - | |
| OS07T0464200-00 |  | OS07g0464200 | | Os07g0464200 | |  | | - | |
| OS07T0464600-01 |  | OS07g0464600 | | Os07g0464600 | |  | | - | |
| OS07T0467200-01 | Up | OS07g0467200 | | Os07g0467200 | |  | | Up- | |
| OS07T0467500-01 |  | OS07g0467500 | | Os07g0467500 | |  | | - | |
| OS07T0468100-02 | Up | OS07g0468100 | | Os07g0468100 | |  | | Up- | |
| OS07T0469100-02 |  | OS07g0469100 | | Os07g0469100 | | Down | | -Down | |
| OS07T0470700-01 |  | OS07g0470700 | | Os07g0470700 | |  | | - | |
| OS07T0472100-00 |  | OS07g0472100 | | Os07g0472100 | |  | | - | |
| OS07T0472500-01 |  | OS07g0472500 | | Os07g0472500 | |  | | - | |
| OS07T0475000-01 |  | OS07g0475000 | | Os07g0475000 | |  | | - | |
| OS07T0476200-02 |  | OS07g0476200 | | Os07g0476200 | |  | | - | |
| OS07T0476500-01 |  | OS07g0476500 | | Os07g0476500 | |  | | - | |
| OS07T0476900-01 |  | OS07g0476900 | | Os07g0476900 | |  | | - | |
| OS07T0477500-01 |  | OS07g0477500 | | Os07g0477500 | |  | | - | |
| OS07T0479300-01 |  | OS07g0479300 | | Os07g0479300 | |  | | - | |
| OS07T0480900-01 |  | OS07g0480900 | | Os07g0480900 | |  | | - | |
| OS07T0483500-03 |  | OS07g0483500 | | Os07g0483500 | |  | | - | |
| OS07T0485100-01 |  | OS07g0485100 | | Os07g0485100 | | Up | | -Up | |
| OS07T0485400-01 | Down | OS07g0485400 | | Os07g0485400 | |  | | Down- | |
| OS07T0486500-02 |  | OS07g0486500 | | Os07g0486500 | |  | | - | |
| OS07T0489800-01 |  | OS07g0489800 | | Os07g0489800 | | Down | | -Down | |
| OS07T0490200-02 |  | OS07g0490200 | | Os07g0490200 | |  | | - | |
| OS07T0490300-01 |  | OS07g0490300 | | Os07g0490300 | |  | | - | |
| OS07T0490400-01 |  | OS07g0490400 | | Os07g0490400 | |  | | - | |
| OS07T0490600-02 |  | OS07g0490600 | | Os07g0490600 | |  | | - | |
| OS07T0490800-01 |  | OS07g0490800 | | Os07g0490800 | |  | | - | |
| OS07T0491900-00 |  | OS07g0491900 | | Os07g0491900 | | Up | | -Up | |
| OS07T0492000-01 |  | OS07g0492000 | | Os07g0492000 | |  | | - | |
| OS07T0495000-01 |  | OS07g0495000 | | Os07g0495000 | |  | | - | |
| OS07T0495200-01 |  | OS07g0495200 | | Os07g0495200 | |  | | - | |
| OS07T0495900-01 |  | OS07g0495900 | | Os07g0495900 | |  | | - | |
| OS07T0496000-01 |  | OS07g0496000 | | Os07g0496000 | |  | | - | |
| OS07T0496200-02 | Up | OS07g0496200 | | Os07g0496200 | |  | | Up- | |
| OS07T0496250-01 |  | OS07g0496250 | | Os07g0496250 | |  | | - | |
| OS07T0498800-01 |  | OS07g0498800 | | Os07g0498800 | |  | | - | |
| OS07T0503300-02 | Up | OS07g0503300 | | Os07g0503300 | | Up | | Up-Up | |
| OS07T0506700-01 |  | OS07g0506700 | | Os07g0506700 | |  | | - | |
| OS07T0507000-01 |  | OS07g0507000 | | Os07g0507000 | |  | | - | |
| OS07T0507300-02 |  | OS07g0507300 | | Os07g0507300 | |  | | - | |
| OS07T0508000-01 |  | OS07g0508000 | | Os07g0508000 | |  | | - | |
| OS07T0508200-01 |  | OS07g0508200 | | Os07g0508200 | |  | | - | |
| OS07T0509800-01 |  | OS07g0509800 | | Os07g0509800 | |  | | - | |
| OS07T0510500-01 |  | OS07g0510500 | | Os07g0510500 | |  | | - | |
| OS07T0513000-02 | Up | OS07g0513000 | | Os07g0513000 | |  | | Up- | |
| OS07T0515100-02 |  | OS07g0515100 | | Os07g0515100 | |  | | - | |
| OS07T0516200-01 |  | OS07g0516200 | | Os07g0516200 | |  | | - | |
| OS07T0516300-01 |  | OS07g0516300 | | Os07g0516300 | |  | | - | |
| OS07T0516900-01 |  | OS07g0516900 | | Os07g0516900 | |  | | - | |
| OS07T0516900-03 |  | OS07g0516900 | | Os07g0516900 | |  | | - | |
| OS07T0522500-01 |  | OS07g0522500 | | Os07g0522500 | |  | | - | |
| OS07T0529000-01 |  | OS07g0529000 | | Os07g0529000 | | Up | | -Up | |
| OS07T0529600-01 |  | OS07g0529600 | | Os07g0529600 | |  | | - | |
| OS07T0529800-01 |  | OS07g0529800 | | Os07g0529800 | |  | | - | |
| OS07T0530600-01 |  | OS07g0530600 | | Os07g0530600 | |  | | - | |
| OS07T0531500-01 |  | OS07g0531500 | | Os07g0531500 | |  | | - | |
| OS07T0531600-01 |  | OS07g0531600 | | Os07g0531600 | |  | | - | |
| OS07T0532800-00 | Up | OS07g0532800 | | Os07g0532800 | |  | | Up- | |
| OS07T0533000-00 |  | OS07g0533000 | | Os07g0533000 | |  | | - | |
| OS07T0534000-01 |  | OS07g0534000 | | Os07g0534000 | | Down | | -Down | |
| OS07T0537000-01 |  | OS07g0537000 | | Os07g0537000 | | Down | | -Down | |
| OS07T0539100-02 |  | OS07g0539100 | | Os07g0539100 | |  | | - | |
| OS07T0539300-01 |  | OS07g0539300 | | Os07g0539300 | |  | | - | |
| OS07T0539900-01 | Up | OS07g0539900 | | Os07g0539900 | | Down | | Up-Down | |
| OS07T0540100-01 |  | OS07g0540100 | | Os07g0540100 | |  | | - | |
| OS07T0541200-00 |  | OS07g0541200 | | Os07g0541200 | |  | | - | |
| OS07T0544800-01 |  | OS07g0544800 | | Os07g0544800 | | Down | | -Down | |
| OS07T0546000-01 |  | OS07g0546000 | | Os07g0546000 | |  | | - | |
| OS07T0546100-01 |  | OS07g0546100 | | Os07g0546100 | |  | | - | |
| OS07T0546600-01 |  | OS07g0546600 | | Os07g0546600 | |  | | - | |
| OS07T0548300-01 |  | OS07g0548300 | | Os07g0548300 | |  | | - | |
| OS07T0548900-01 |  | OS07g0548900 | | Os07g0548900 | |  | | - | |
| OS07T0549700-01 |  | OS07g0549700 | | Os07g0549700 | |  | | - | |
| OS07T0551400-02 |  | OS07g0551400 | | Os07g0551400 | |  | | - | |
| OS07T0555200-01 |  | OS07g0555200 | | Os07g0555200 | |  | | - | |
| OS07T0555200-02 |  | OS07g0555200 | | Os07g0555200 | |  | | - | |
| OS07T0556200-01 |  | OS07g0556200 | | Os07g0556200 | |  | | - | |
| OS07T0557500-01 |  | OS07g0557500 | | Os07g0557500 | |  | | - | |
| OS07T0558200-02 |  | OS07g0558200 | | Os07g0558200 | |  | | - | |
| OS07T0558300-01 |  | OS07g0558300 | | Os07g0558300 | |  | | - | |
| OS07T0558400-01 |  | OS07g0558400 | | Os07g0558400 | |  | | - | |
| OS07T0558500-01 |  | OS07g0558500 | | Os07g0558500 | |  | | - | |
| OS07T0558800-01 |  | OS07g0558800 | | Os07g0558800 | |  | | - | |
| OS07T0559400-00 |  | OS07g0559400 | | Os07g0559400 | |  | | - | |
| OS07T0561500-01 |  | OS07g0561500 | | Os07g0561500 | |  | | - | |
| OS07T0562700-02 |  | OS07g0562700 | | Os07g0562700 | |  | | - | |
| OS07T0563800-04 |  | OS07g0563800 | | Os07g0563800 | |  | | - | |
| OS07T0565600-02 | Down | OS07g0565600 | | Os07g0565600 | | Down | | Down-Down | |
| OS07T0565700-02 |  | OS07g0565700 | | Os07g0565700 | |  | | - | |
| OS07T0568600-01 |  | OS07g0568600 | | Os07g0568600 | |  | | - | |
| OS07T0568900-01 |  | OS07g0568900 | | Os07g0568900 | |  | | - | |
| OS07T0569100-01 |  | OS07g0569100 | | Os07g0569100 | |  | | - | |
| OS07T0569600-01 |  | OS07g0569600 | | Os07g0569600 | |  | | - | |
| OS07T0569700-01 |  | OS07g0569700 | | Os07g0569700 | |  | | - | |
| OS07T0570300-01 |  | OS07g0570300 | | Os07g0570300 | |  | | - | |
| OS07T0570700-01 |  | OS07g0570700 | | Os07g0570700 | |  | | - | |
| OS07T0571100-01 |  | OS07g0571100 | | Os07g0571100 | |  | | - | |
| OS07T0571100-02 |  | OS07g0571100 | | Os07g0571100 | |  | | - | |
| OS07T0571900-01 |  | OS07g0571900 | | Os07g0571900 | |  | | - | |
| OS07T0573100-01 |  | OS07g0573100 | | Os07g0573100 | |  | | - | |
| OS07T0573800-01 |  | OS07g0573800 | | Os07g0573800 | |  | | - | |
| OS07T0574800-02 |  | OS07g0574800 | | Os07g0574800 | |  | | - | |
| OS07T0575100-00 |  | OS07g0575100 | | Os07g0575100 | |  | | - | |
| OS07T0575500-00 |  | OS07g0575500 | | Os07g0575500 | |  | | - | |
| OS07T0577400-01 |  | OS07g0577400 | | Os07g0577400 | |  | | - | |
| OS07T0577600-01 |  | OS07g0577600 | | Os07g0577600 | |  | | - | |
| OS07T0577700-01 |  | OS07g0577700 | | Os07g0577700 | |  | | - | |
| OS07T0578600-03 |  | OS07g0578600 | | Os07g0578600 | |  | | - | |
| OS07T0582400-01 | Up | OS07g0582400 | | Os07g0582400 | | Up | | Up-Up | |
| OS07T0583600-03 |  | OS07g0583600 | | Os07g0583600 | |  | | - | |
| OS07T0584500-02 |  | OS07g0584500 | | Os07g0584500 | |  | | - | |
| OS07T0585000-01 |  | OS07g0585000 | | Os07g0585000 | |  | | - | |
| OS07T0585100-01 |  | OS07g0585100 | | Os07g0585100 | |  | | - | |
| OS07T0585800-01 |  | OS07g0585800 | | Os07g0585800 | |  | | - | |
| OS07T0586100-01 | Up | OS07g0586100 | | Os07g0586100 | | Up | | Up-Up | |
| OS07T0586200-02 |  | OS07g0586200 | | Os07g0586200 | |  | | - | |
| OS07T0586500-02 |  | OS07g0586500 | | Os07g0586500 | |  | | - | |
| OS07T0587100-01 |  | OS07g0587100 | | Os07g0587100 | |  | | - | |
| OS07T0587400-01 |  | OS07g0587400 | | Os07g0587400 | |  | | - | |
| OS07T0592100-01 |  | OS07g0592100 | | Os07g0592100 | |  | | - | |
| OS07T0592200-02 |  | OS07g0592200 | | Os07g0592200 | |  | | - | |
| OS07T0592300-01 |  | OS07g0592300 | | Os07g0592300 | |  | | - | |
| OS07T0595700-01 |  | OS07g0595700 | | Os07g0595700 | |  | | - | |
| OS07T0597000-01 |  | OS07g0597000 | | Os07g0597000 | |  | | - | |
| OS07T0599000-01 |  | OS07g0599000 | | Os07g0599000 | |  | | - | |
| OS07T0601000-01 |  | OS07g0601000 | | Os07g0601000 | |  | | - | |
| OS07T0602200-01 |  | OS07g0602200 | | Os07g0602200 | |  | | - | |
| OS07T0603200-01 |  | OS07g0603200 | | Os07g0603200 | |  | | - | |
| OS07T0603900-01 |  | OS07g0603900 | | Os07g0603900 | |  | | - | |
| OS07T0604500-01 |  | OS07g0604500 | | Os07g0604500 | |  | | - | |
| OS07T0604800-01 |  | OS07g0604800 | | Os07g0604800 | |  | | - | |
| OS07T0606800-01 |  | OS07g0606800 | | Os07g0606800 | |  | | - | |
| OS07T0607800-01 |  | OS07g0607800 | | Os07g0607800 | |  | | - | |
| OS07T0608700-02 |  | OS07g0608700 | | Os07g0608700 | |  | | - | |
| OS07T0609000-04 |  | OS07g0609000 | | Os07g0609000 | |  | | - | |
| OS07T0609766-00 |  | OS07g0609766 | | Os07g0609766 | |  | | - | |
| OS07T0611400-00 |  | OS07g0611400 | | Os07g0611400 | |  | | - | |
| OS07T0613200-00 |  | OS07g0613200 | | Os07g0613200 | |  | | - | |
| OS07T0614000-02 |  | OS07g0614000 | | Os07g0614000 | | Up | | -Up | |
| OS07T0614500-01 |  | OS07g0614500 | | Os07g0614500 | |  | | - | |
| OS07T0616200-01 |  | OS07g0616200 | | Os07g0616200 | | Down | | -Down | |
| OS07T0616500-01 |  | OS07g0616500 | | Os07g0616500 | |  | | - | |
| OS07T0616600-02 |  | OS07g0616600 | | Os07g0616600 | |  | | - | |
| OS07T0617100-01 |  | OS07g0617100 | | Os07g0617100 | |  | | - | |
| OS07T0617800-01 |  | OS07g0617800 | | Os07g0617800 | |  | | - | |
| OS07T0618500-02 | Up | OS07g0618500 | | Os07g0618500 | |  | | Up- | |
| OS07T0618600-02 |  | OS07g0618600 | | Os07g0618600 | |  | | - | |
| OS07T0618800-01 |  | OS07g0618800 | | Os07g0618800 | |  | | - | |
| OS07T0620200-01 |  | OS07g0620200 | | Os07g0620200 | |  | | - | |
| OS07T0620300-03 |  | OS07g0620300 | | Os07g0620300 | |  | | - | |
| OS07T0621300-02 |  | OS07g0621300 | | Os07g0621300 | |  | | - | |
| OS07T0622100-01 |  | OS07g0622100 | | Os07g0622100 | |  | | - | |
| OS07T0622200-01 |  | OS07g0622200 | | Os07g0622200 | |  | | - | |
| OS07T0623100-01 |  | OS07g0623100 | | Os07g0623100 | |  | | - | |
| OS07T0623200-03 |  | OS07g0623200 | | Os07g0623200 | |  | | - | |
| OS07T0623300-01 |  | OS07g0623300 | | Os07g0623300 | |  | | - | |
| OS07T0624700-01 |  | OS07g0624700 | | Os07g0624700 | |  | | - | |
| OS07T0626400-01 |  | OS07g0626400 | | Os07g0626400 | |  | | - | |
| OS07T0626800-01 |  | OS07g0626800 | | Os07g0626800 | | Down | | -Down | |
| OS07T0628700-01 |  | OS07g0628700 | | Os07g0628700 | |  | | - | |
| OS07T0628900-01 |  | OS07g0628900 | | Os07g0628900 | |  | | - | |
| OS07T0630800-04 |  | OS07g0630800 | | Os07g0630800 | |  | | - | |
| OS07T0631100-02 |  | OS07g0631100 | | Os07g0631100 | |  | | - | |
| OS07T0631900-01 | Down | OS07g0631900 | | Os07g0631900 | |  | | Down- | |
| OS07T0632600-02 |  | OS07g0632600 | | Os07g0632600 | |  | | - | |
| OS07T0632700-01 |  | OS07g0632700 | | Os07g0632700 | |  | | - | |
| OS07T0632800-01 |  | OS07g0632800 | | Os07g0632800 | |  | | - | |
| OS07T0633200-02 |  | OS07g0633200 | | Os07g0633200 | | Up | | -Up | |
| OS07T0633500-00 |  | OS07g0633500 | | Os07g0633500 | |  | | - | |
| OS07T0636000-01 |  | OS07g0636000 | | Os07g0636000 | |  | | - | |
| OS07T0637300-01 |  | OS07g0637300 | | Os07g0637300 | | Up | | -Up | |
| OS07T0638100-01 |  | OS07g0638100 | | Os07g0638100 | | Up | | -Up | |
| OS07T0639800-02 |  | OS07g0639800 | | Os07g0639800 | |  | | - | |
| OS07T0640200-01 |  | OS07g0640200 | | Os07g0640200 | |  | | - | |
| OS07T0641700-01 |  | OS07g0641700 | | Os07g0641700 | | Down | | -Down | |
| OS07T0643100-01 |  | OS07g0643100 | | Os07g0643100 | | Up | | -Up | |
| OS07T0644300-02 |  | OS07g0644300 | | Os07g0644300 | |  | | - | |
| OS07T0645100-01 |  | OS07g0645100 | | Os07g0645100 | |  | | - | |
| OS07T0645400-01 |  | OS07g0645400 | | Os07g0645400 | |  | | - | |
| OS07T0646100-00 |  | OS07g0646100 | | Os07g0646100 | | Up | | -Up | |
| OS07T0647500-01 |  | OS07g0647500 | | Os07g0647500 | |  | | - | |
| OS07T0647800-01 |  | OS07g0647800 | | Os07g0647800 | |  | | - | |
| OS07T0648266-00 |  | OS07g0648266 | | Os07g0648266 | |  | | - | |
| OS07T0656700-01 |  | OS07g0656700 | | Os07g0656700 | | Up | | -Up | |
| OS07T0656900-00 | Up | OS07g0656900 | | Os07g0656900 | |  | | Up- | |
| OS07T0657200-02 |  | OS07g0657200 | | Os07g0657200 | |  | | - | |
| OS07T0657900-01 |  | OS07g0657900 | | Os07g0657900 | |  | | - | |
| OS07T0658400-01 |  | OS07g0658400 | | Os07g0658400 | |  | | - | |
| OS07T0658400-02 |  | OS07g0658400 | | Os07g0658400 | |  | | - | |
| OS07T0658600-00 | Down | OS07g0658600 | | Os07g0658600 | |  | | Down- | |
| OS07T0660200-02 |  | OS07g0660200 | | Os07g0660200 | |  | | - | |
| OS07T0661300-01 |  | OS07g0661300 | | Os07g0661300 | |  | | - | |
| OS07T0662500-01 |  | OS07g0662500 | | Os07g0662500 | |  | | - | |
| OS07T0662900-01 | Down | OS07g0662900 | | Os07g0662900 | |  | | Down- | |
| OS07T0663700-01 | Up | OS07g0663700 | | Os07g0663700 | |  | | Up- | |
| OS07T0664000-00 |  | OS07g0664000 | | Os07g0664000 | |  | | - | |
| OS07T0665000-00 |  | OS07g0665000 | | Os07g0665000 | |  | | - | |
| OS07T0665200-01 | Up | OS07g0665200 | | Os07g0665200 | | Up | | Up-Up | |
| OS07T0665700-01 |  | OS07g0665700 | | Os07g0665700 | |  | | - | |
| OS07T0668200-02 |  | OS07g0668200 | | Os07g0668200 | |  | | - | |
| OS07T0669100-01 |  | OS07g0669100 | | Os07g0669100 | |  | | - | |
| OS07T0669200-00 |  | OS07g0669200 | | Os07g0669200 | |  | | - | |
| OS07T0669650-01 |  | OS07g0669650 | | Os07g0669650 | |  | | - | |
| OS07T0671700-01 |  | OS07g0671700 | | Os07g0671700 | |  | | - | |
| OS07T0671800-01 | Up | OS07g0671800 | | Os07g0671800 | |  | | Up- | |
| OS07T0672500-02 |  | OS07g0672500 | | Os07g0672500 | |  | | - | |
| OS07T0673400-01 |  | OS07g0673400 | | Os07g0673400 | |  | | - | |
| OS07T0674200-01 |  | OS07g0674200 | | Os07g0674200 | |  | | - | |
| OS07T0674400-01 |  | OS07g0674400 | | Os07g0674400 | |  | | - | |
| OS07T0675000-02 |  | OS07g0675000 | | Os07g0675000 | |  | | - | |
| OS07T0677100-01 |  | OS07g0677100 | | Os07g0677100 | |  | | - | |
| OS07T0677200-01 | Up | OS07g0677200 | | Os07g0677200 | | Down | | Up-Down | |
| OS07T0677400-00 |  | OS07g0677400 | | Os07g0677400 | | Down | | -Down | |
| OS07T0677500-00 |  | OS07g0677500 | | Os07g0677500 | | Down | | -Down | |
| OS07T0679300-01 | Up | OS07g0679300 | | Os07g0679300 | | Up | | Up-Up | |
| OS07T0679500-01 |  | OS07g0679500 | | Os07g0679500 | |  | | - | |
| OS07T0680300-01 |  | OS07g0680300 | | Os07g0680300 | |  | | - | |
| OS07T0681000-01 |  | OS07g0681000 | | Os07g0681000 | |  | | - | |
| OS07T0681400-01 |  | OS07g0681400 | | Os07g0681400 | |  | | - | |
| OS07T0682400-03 |  | OS07g0682400 | | Os07g0682400 | |  | | - | |
| OS07T0682700-01 |  | OS07g0682700 | | Os07g0682700 | |  | | - | |
| OS07T0682800-01 |  | OS07g0682800 | | Os07g0682800 | |  | | - | |
| OS07T0683600-00 |  | OS07g0683600 | | Os07g0683600 | | Up | | -Up | |
| OS07T0683900-01 |  | OS07g0683900 | | Os07g0683900 | |  | | - | |
| OS07T0684000-01 |  | OS07g0684000 | | Os07g0684000 | | Up | | -Up | |
| OS07T0687200-02 | Up | OS07g0687200 | | Os07g0687200 | |  | | Up- | |
| OS07T0687500-01 |  | OS07g0687500 | | Os07g0687500 | |  | | - | |
| OS07T0687700-01 |  | OS07g0687700 | | Os07g0687700 | |  | | - | |
| OS07T0687900-01 |  | OS07g0687900 | | Os07g0687900 | |  | | - | |
| OS07T0688800-01 |  | OS07g0688800 | | Os07g0688800 | |  | | - | |
| OS07T0691200-02 | Down | OS07g0691200 | | Os07g0691200 | |  | | Down- | |
| OS07T0691800-01 |  | OS07g0691800 | | Os07g0691800 | |  | | - | |
| OS07T0693500-01 |  | OS07g0693500 | | Os07g0693500 | | Up | | -Up | |
| OS07T0694000-01 |  | OS07g0694000 | | Os07g0694000 | |  | | - | |
| OS07T0694500-02 |  | OS07g0694500 | | Os07g0694500 | |  | | - | |
| OS07T0694600-01 |  | OS07g0694600 | | Os07g0694600 | |  | | - | |
| OS07T0694700-01 | Up | OS07g0694700 | | Os07g0694700 | |  | | Up- | |
| OS07T0694800-01 |  | OS07g0694800 | | Os07g0694800 | |  | | - | |
| OS07T0695800-01 |  | OS07g0695800 | | Os07g0695800 | |  | | - | |
| OS08T0102000-01 |  | OS08g0102000 | | Os08g0102000 | |  | | - | |
| OS08T0102100-01 |  | OS08g0102100 | | Os08g0102100 | |  | | - | |
| OS08T0104600-02 |  | OS08g0104600 | | Os08g0104600 | |  | | - | |
| OS08T0107500-01 |  | OS08g0107500 | | Os08g0107500 | |  | | - | |
| OS08T0108300-01 |  | OS08g0108300 | | Os08g0108300 | |  | | - | |
| OS08T0109200-01 |  | OS08g0109200 | | Os08g0109200 | |  | | - | |
| OS08T0109300-01 | Up | OS08g0109300 | | Os08g0109300 | |  | | Up- | |
| OS08T0109900-01 |  | OS08g0109900 | | Os08g0109900 | |  | | - | |
| OS08T0110000-02 |  | OS08g0110000 | | Os08g0110000 | |  | | - | |
| OS08T0110500-01 |  | OS08g0110500 | | Os08g0110500 | |  | | - | |
| OS08T0110800-03 |  | OS08g0110800 | | Os08g0110800 | |  | | - | |
| OS08T0110800-04 |  | OS08g0110800 | | Os08g0110800 | |  | | - | |
| OS08T0111200-01 | Down | OS08g0111200 | | Os08g0111200 | |  | | Down- | |
| OS08T0112600-01 |  | OS08g0112600 | | Os08g0112600 | |  | | - | |
| OS08T0112800-01 |  | OS08g0112800 | | Os08g0112800 | |  | | - | |
| OS08T0113000-01 |  | OS08g0113000 | | Os08g0113000 | |  | | - | |
| OS08T0113100-01 |  | OS08g0113100 | | Os08g0113100 | |  | | - | |
| OS08T0113200-01 |  | OS08g0113200 | | Os08g0113200 | |  | | - | |
| OS08T0114100-01 |  | OS08g0114100 | | Os08g0114100 | |  | | - | |
| OS08T0114300-01 |  | OS08g0114300 | | Os08g0114300 | |  | | - | |
| OS08T0116500-01 |  | OS08g0116500 | | Os08g0116500 | |  | | - | |
| OS08T0117100-01 |  | OS08g0117100 | | Os08g0117100 | |  | | - | |
| OS08T0117200-00 |  | OS08g0117200 | | Os08g0117200 | |  | | - | |
| OS08T0117300-01 | Down | OS08g0117300 | | Os08g0117300 | |  | | Down- | |
| OS08T0117400-02 | Up | OS08g0117400 | | Os08g0117400 | |  | | Up- | |
| OS08T0119000-02 |  | OS08g0119000 | | Os08g0119000 | |  | | - | |
| OS08T0119800-02 |  | OS08g0119800 | | Os08g0119800 | |  | | - | |
| OS08T0120000-04 |  | OS08g0120000 | | Os08g0120000 | |  | | - | |
| OS08T0120500-02 |  | OS08g0120500 | | Os08g0120500 | |  | | - | |
| OS08T0120600-01 |  | OS08g0120600 | | Os08g0120600 | |  | | - | |
| OS08T0122000-02 |  | OS08g0122000 | | Os08g0122000 | |  | | - | |
| OS08T0126300-02 |  | OS08g0126300 | | Os08g0126300 | |  | | - | |
| OS08T0126700-01 |  | OS08g0126700 | | Os08g0126700 | | Down | | -Down | |
| OS08T0127100-02 |  | OS08g0127100 | | Os08g0127100 | |  | | - | |
| OS08T0127600-01 |  | OS08g0127600 | | Os08g0127600 | |  | | - | |
| OS08T0127700-02 |  | OS08g0127700 | | Os08g0127700 | |  | | - | |
| OS08T0129200-02 |  | OS08g0129200 | | Os08g0129200 | |  | | - | |
| OS08T0130500-01 |  | OS08g0130500 | | Os08g0130500 | |  | | - | |
| OS08T0133000-01 |  | OS08g0133000 | | Os08g0133000 | |  | | - | |
| OS08T0135800-01 |  | OS08g0135800 | | Os08g0135800 | |  | | - | |
| OS08T0135900-01 |  | OS08g0135900 | | Os08g0135900 | |  | | - | |
| OS08T0137200-01 |  | OS08g0137200 | | Os08g0137200 | |  | | - | |
| OS08T0137400-02 |  | OS08g0137400 | | Os08g0137400 | | Down | | -Down | |
| OS08T0139000-01 |  | OS08g0139000 | | Os08g0139000 | |  | | - | |
| OS08T0139100-01 |  | OS08g0139100 | | Os08g0139100 | | Down | | -Down | |
| OS08T0139200-01 |  | OS08g0139200 | | Os08g0139200 | |  | | - | |
| OS08T0141300-01 |  | OS08g0141300 | | Os08g0141300 | |  | | - | |
| OS08T0143400-02 |  | OS08g0143400 | | Os08g0143400 | |  | | - | |
| OS08T0144000-01 |  | OS08g0144000 | | Os08g0144000 | | Up | | -Up | |
| OS08T0144400-02 |  | OS08g0144400 | | Os08g0144400 | |  | | - | |
| OS08T0148267-00 |  | OS08g0148267 | | Os08g0148267 | |  | | - | |
| OS08T0150800-01 |  | OS08g0150800 | | Os08g0150800 | |  | | - | |
| OS08T0151400-01 |  | OS08g0151400 | | Os08g0151400 | |  | | - | |
| OS08T0151800-01 | Up | OS08g0151800 | | Os08g0151800 | |  | | Up- | |
| OS08T0152400-00 |  | OS08g0152400 | | Os08g0152400 | |  | | - | |
| OS08T0152700-01 |  | OS08g0152700 | | Os08g0152700 | |  | | - | |
| OS08T0154600-01 |  | OS08g0154600 | | Os08g0154600 | |  | | - | |
| OS08T0154700-01 |  | OS08g0154700 | | Os08g0154700 | |  | | - | |
| OS08T0155000-02 |  | OS08g0155000 | | Os08g0155000 | |  | | - | |
| OS08T0155100-01 |  | OS08g0155100 | | Os08g0155100 | |  | | - | |
| OS08T0156700-01 |  | OS08g0156700 | | Os08g0156700 | |  | | - | |
| OS08T0157100-01 | Up | OS08g0157100 | | Os08g0157100 | |  | | Up- | |
| OS08T0157500-02 |  | OS08g0157500 | | Os08g0157500 | | Up | | -Up | |
| OS08T0157900-01 |  | OS08g0157900 | | Os08g0157900 | |  | | - | |
| OS08T0158200-02 |  | OS08g0158200 | | Os08g0158200 | |  | | - | |
| OS08T0158500-02 |  | OS08g0158500 | | Os08g0158500 | |  | | - | |
| OS08T0158900-01 |  | OS08g0158900 | | Os08g0158900 | |  | | - | |
| OS08T0159100-01 |  | OS08g0159100 | | Os08g0159100 | |  | | - | |
| OS08T0159900-01 |  | OS08g0159900 | | Os08g0159900 | |  | | - | |
| OS08T0161700-01 |  | OS08g0161700 | | Os08g0161700 | |  | | - | |
| OS08T0162100-01 |  | OS08g0162100 | | Os08g0162100 | |  | | - | |
| OS08T0162600-02 |  | OS08g0162600 | | Os08g0162600 | |  | | - | |
| OS08T0162800-01 |  | OS08g0162800 | | Os08g0162800 | |  | | - | |
| OS08T0167500-02 |  | OS08g0167500 | | Os08g0167500 | |  | | - | |
| OS08T0169800-01 |  | OS08g0169800 | | Os08g0169800 | |  | | - | |
| OS08T0170900-02 |  | OS08g0170900 | | Os08g0170900 | |  | | - | |
| OS08T0172200-01 |  | OS08g0172200 | | Os08g0172200 | |  | | - | |
| OS08T0174300-00 |  | OS08g0174300 | | Os08g0174300 | | Up | | -Up | |
| OS08T0174700-01 |  | OS08g0174700 | | Os08g0174700 | |  | | - | |
| OS08T0175200-02 |  | OS08g0175200 | | Os08g0175200 | |  | | - | |
| OS08T0176100-01 |  | OS08g0176100 | | Os08g0176100 | |  | | - | |
| OS08T0176800-01 |  | OS08g0176800 | | Os08g0176800 | |  | | - | |
| OS08T0177800-00 |  | OS08g0177800 | | Os08g0177800 | |  | | - | |
| OS08T0178300-01 |  | OS08g0178300 | | Os08g0178300 | |  | | - | |
| OS08T0178700-01 |  | OS08g0178700 | | Os08g0178700 | |  | | - | |
| OS08T0180500-00 |  | OS08g0180500 | | Os08g0180500 | |  | | - | |
| OS08T0187500-01 |  | OS08g0187500 | | Os08g0187500 | |  | | - | |
| OS08T0187700-01 |  | OS08g0187700 | | Os08g0187700 | |  | | - | |
| OS08T0189850-00 |  | OS08g0189850 | | Os08g0189850 | |  | | - | |
| OS08T0190200-00 |  | OS08g0190200 | | Os08g0190200 | |  | | - | |
| OS08T0190300-00 |  | OS08g0190300 | | Os08g0190300 | |  | | - | |
| OS08T0191200-01 |  | OS08g0191200 | | Os08g0191200 | |  | | - | |
| OS08T0191700-03 | Up | OS08g0191700 | | Os08g0191700 | |  | | Up- | |
| OS08T0192900-01 |  | OS08g0192900 | | Os08g0192900 | |  | | - | |
| OS08T0199300-01 |  | OS08g0199300 | | Os08g0199300 | |  | | - | |
| OS08T0200300-02 | Down | OS08g0200300 | | Os08g0200300 | |  | | Down- | |
| OS08T0203600-04 |  | OS08g0203600 | | Os08g0203600 | | Down | | -Down | |
| OS08T0205400-01 |  | OS08g0205400 | | Os08g0205400 | |  | | - | |
| OS08T0206600-02 |  | OS08g0206600 | | Os08g0206600 | |  | | - | |
| OS08T0206700-01 |  | OS08g0206700 | | Os08g0206700 | |  | | - | |
| OS08T0206900-01 |  | OS08g0206900 | | Os08g0206900 | |  | | - | |
| OS08T0218700-01 |  | OS08g0218700 | | Os08g0218700 | |  | | - | |
| OS08T0224200-01 |  | OS08g0224200 | | Os08g0224200 | |  | | - | |
| OS08T0224500-01 |  | OS08g0224500 | | Os08g0224500 | |  | | - | |
| OS08T0224700-02 |  | OS08g0224700 | | Os08g0224700 | |  | | - | |
| OS08T0225000-00 |  | OS08g0225000 | | Os08g0225000 | | Down | | -Down | |
| OS08T0231400-01 |  | OS08g0231400 | | Os08g0231400 | |  | | - | |
| OS08T0234000-01 |  | OS08g0234000 | | Os08g0234000 | |  | | - | |
| OS08T0237000-01 | Down | OS08g0237000 | | Os08g0237000 | |  | | Down- | |
| OS08T0238200-02 |  | OS08g0238200 | | Os08g0238200 | |  | | - | |
| OS08T0239000-01 |  | OS08g0239000 | | Os08g0239000 | |  | | - | |
| OS08T0240800-01 |  | OS08g0240800 | | Os08g0240800 | |  | | - | |
| OS08T0241600-01 |  | OS08g0241600 | | Os08g0241600 | |  | | - | |
| OS08T0242700-01 | Down | OS08g0242700 | | Os08g0242700 | |  | | Down- | |
| OS08T0242700-02 |  | OS08g0242700 | | Os08g0242700 | |  | | - | |
| OS08T0243500-02 |  | OS08g0243500 | | Os08g0243500 | | Up | | -Up | |
| OS08T0243600-01 |  | OS08g0243600 | | Os08g0243600 | |  | | - | |
| OS08T0243900-01 |  | OS08g0243900 | | Os08g0243900 | |  | | - | |
| OS08T0245400-01 |  | OS08g0245400 | | Os08g0245400 | |  | | - | |
| OS08T0248800-01 |  | OS08g0248800 | | Os08g0248800 | |  | | - | |
| OS08T0250200-01 |  | OS08g0250200 | | Os08g0250200 | |  | | - | |
| OS08T0254500-03 |  | OS08g0254500 | | Os08g0254500 | |  | | - | |
| OS08T0254900-01 |  | OS08g0254900 | | Os08g0254900 | |  | | - | |
| OS08T0266300-00 |  | OS08g0266300 | | Os08g0266300 | |  | | - | |
| OS08T0266600-01 |  | OS08g0266600 | | Os08g0266600 | |  | | - | |
| OS08T0276100-01 | Down | OS08g0276100 | | Os08g0276100 | | Down | | Down-Down | |
| OS08T0276200-01 |  | OS08g0276200 | | Os08g0276200 | |  | | - | |
| OS08T0277900-01 |  | OS08g0277900 | | Os08g0277900 | |  | | - | |
| OS08T0278750-01 |  | OS08g0278750 | | Os08g0278750 | |  | | - | |
| OS08T0278900-01 |  | OS08g0278900 | | Os08g0278900 | |  | | - | |
| OS08T0282000-01 |  | OS08g0282000 | | Os08g0282000 | |  | | - | |
| OS08T0282400-01 | Up | OS08g0282400 | | Os08g0282400 | |  | | Up- | |
| OS08T0288200-01 | Down | OS08g0288200 | | Os08g0288200 | |  | | Down- | |
| OS08T0292600-03 |  | OS08g0292600 | | Os08g0292600 | |  | | - | |
| OS08T0292900-01 |  | OS08g0292900 | | Os08g0292900 | |  | | - | |
| OS08T0295300-01 |  | OS08g0295300 | | Os08g0295300 | |  | | - | |
| OS08T0296900-01 |  | OS08g0296900 | | Os08g0296900 | |  | | - | |
| OS08T0299000-02 |  | OS08g0299000 | | Os08g0299000 | |  | | - | |
| OS08T0299200-01 |  | OS08g0299200 | | Os08g0299200 | |  | | - | |
| OS08T0300200-02 |  | OS08g0300200 | | Os08g0300200 | |  | | - | |
| OS08T0301500-01 |  | OS08g0301500 | | Os08g0301500 | |  | | - | |
| OS08T0308100-01 |  | OS08g0308100 | | Os08g0308100 | |  | | - | |
| OS08T0308700-00 |  | OS08g0308700 | | Os08g0308700 | |  | | - | |
| OS08T0310100-01 |  | OS08g0310100 | | Os08g0310100 | |  | | - | |
| OS08T0313200-01 | Up | OS08g0313200 | | Os08g0313200 | |  | | Up- | |
| OS08T0314800-02 |  | OS08g0314800 | | Os08g0314800 | |  | | - | |
| OS08T0318500-01 |  | OS08g0318500 | | Os08g0318500 | |  | | - | |
| OS08T0319900-01 |  | OS08g0319900 | | Os08g0319900 | |  | | - | |
| OS08T0320100-01 |  | OS08g0320100 | | Os08g0320100 | |  | | - | |
| OS08T0320400-01 |  | OS08g0320400 | | Os08g0320400 | |  | | - | |
| OS08T0321000-02 |  | OS08g0321000 | | Os08g0321000 | | Down | | -Down | |
| OS08T0323000-01 |  | OS08g0323000 | | Os08g0323000 | |  | | - | |
| OS08T0323400-01 |  | OS08g0323400 | | Os08g0323400 | |  | | - | |
| OS08T0326600-01 |  | OS08g0326600 | | Os08g0326600 | |  | | - | |
| OS08T0327400-01 |  | OS08g0327400 | | Os08g0327400 | |  | | - | |
| OS08T0328300-02 |  | OS08g0328300 | | Os08g0328300 | |  | | - | |
| OS08T0332700-03 |  | OS08g0332700 | | Os08g0332700 | |  | | - | |
| OS08T0335000-00 |  | OS08g0335000 | | Os08g0335000 | |  | | - | |
| OS08T0338700-01 |  | OS08g0338700 | | Os08g0338700 | |  | | - | |
| OS08T0342400-02 |  | OS08g0342400 | | Os08g0342400 | |  | | - | |
| OS08T0343000-02 |  | OS08g0343000 | | Os08g0343000 | |  | | - | |
| OS08T0343300-01 |  | OS08g0343300 | | Os08g0343300 | |  | | - | |
| OS08T0345400-01 |  | OS08g0345400 | | Os08g0345400 | |  | | - | |
| OS08T0345700-02 |  | OS08g0345700 | | Os08g0345700 | |  | | - | |
| OS08T0345800-02 |  | OS08g0345800 | | Os08g0345800 | |  | | - | |
| OS08T0357000-01 |  | OS08g0357000 | | Os08g0357000 | |  | | - | |
| OS08T0359000-01 |  | OS08g0359000 | | Os08g0359000 | |  | | - | |
| OS08T0359500-01 |  | OS08g0359500 | | Os08g0359500 | |  | | - | |
| OS08T0364500-00 |  | OS08g0364500 | | Os08g0364500 | | Down | | -Down | |
| OS08T0366000-02 | Up | OS08g0366000 | | Os08g0366000 | |  | | Up- | |
| OS08T0366100-01 |  | OS08g0366100 | | Os08g0366100 | |  | | - | |
| OS08T0369700-01 |  | OS08g0369700 | | Os08g0369700 | |  | | - | |
| OS08T0374000-01 |  | OS08g0374000 | | Os08g0374000 | |  | | - | |
| OS08T0374100-02 |  | OS08g0374100 | | Os08g0374100 | |  | | - | |
| OS08T0374200-02 |  | OS08g0374200 | | Os08g0374200 | |  | | - | |
| OS08T0375400-01 |  | OS08g0375400 | | Os08g0375400 | |  | | - | |
| OS08T0378900-02 |  | OS08g0378900 | | Os08g0378900 | |  | | - | |
| OS08T0379300-01 |  | OS08g0379300 | | Os08g0379300 | |  | | - | |
| OS08T0379400-03 |  | OS08g0379400 | | Os08g0379400 | |  | | - | |
| OS08T0382400-01 |  | OS08g0382400 | | Os08g0382400 | | Down | | -Down | |
| OS08T0383700-01 |  | OS08g0383700 | | Os08g0383700 | |  | | - | |
| OS08T0384100-01 | Down | OS08g0384100 | | Os08g0384100 | |  | | Down- | |
| OS08T0385000-01 |  | OS08g0385000 | | Os08g0385000 | |  | | - | |
| OS08T0385900-01 |  | OS08g0385900 | | Os08g0385900 | |  | | - | |
| OS08T0390000-02 |  | OS08g0390000 | | Os08g0390000 | |  | | - | |
| OS08T0392100-00 |  | OS08g0392100 | | Os08g0392100 | | Up | | -Up | |
| OS08T0398400-01 | Up | OS08g0398400 | | Os08g0398400 | |  | | Up- | |
| OS08T0398700-01 | Up | OS08g0398700 | | Os08g0398700 | |  | | Up- | |
| OS08T0399600-01 |  | OS08g0399600 | | Os08g0399600 | |  | | - | |
| OS08T0401800-00 |  | OS08g0401800 | | Os08g0401800 | |  | | - | |
| OS08T0404300-01 |  | OS08g0404300 | | Os08g0404300 | |  | | - | |
| OS08T0406400-01 |  | OS08g0406400 | | Os08g0406400 | |  | | - | |
| OS08T0407200-01 |  | OS08g0407200 | | Os08g0407200 | |  | | - | |
| OS08T0412800-01 |  | OS08g0412800 | | Os08g0412800 | | Up | | -Up | |
| OS08T0417100-01 |  | OS08g0417100 | | Os08g0417100 | |  | | - | |
| OS08T0422000-01 |  | OS08g0422000 | | Os08g0422000 | |  | | - | |
| OS08T0424200-01 |  | OS08g0424200 | | Os08g0424200 | |  | | - | |
| OS08T0424500-01 |  | OS08g0424500 | | Os08g0424500 | |  | | - | |
| OS08T0425800-02 |  | OS08g0425800 | | Os08g0425800 | |  | | - | |
| OS08T0427900-01 |  | OS08g0427900 | | Os08g0427900 | |  | | - | |
| OS08T0428800-00 |  | OS08g0428800 | | Os08g0428800 | |  | | - | |
| OS08T0430500-03 |  | OS08g0430500 | | Os08g0430500 | |  | | - | |
| OS08T0430700-02 |  | OS08g0430700 | | Os08g0430700 | |  | | - | |
| OS08T0431300-01 |  | OS08g0431300 | | Os08g0431300 | | Down | | -Down | |
| OS08T0431500-01 |  | OS08g0431500 | | Os08g0431500 | |  | | - | |
| OS08T0432500-01 |  | OS08g0432500 | | Os08g0432500 | | Up | | -Up | |
| OS08T0433600-01 |  | OS08g0433600 | | Os08g0433600 | |  | | - | |
| OS08T0434100-01 | Up | OS08g0434100 | | Os08g0434100 | | Up | | Up-Up | |
| OS08T0434300-01 |  | OS08g0434300 | | Os08g0434300 | | Up | | -Up | |
| OS08T0435900-03 |  | OS08g0435900 | | Os08g0435900 | |  | | - | |
| OS08T0436000-01 |  | OS08g0436000 | | Os08g0436000 | |  | | - | |
| OS08T0438400-01 |  | OS08g0438400 | | Os08g0438400 | |  | | - | |
| OS08T0439100-01 |  | OS08g0439100 | | Os08g0439100 | |  | | - | |
| OS08T0440100-01 | Up | OS08g0440100 | | Os08g0440100 | |  | | Up- | |
| OS08T0440500-01 |  | OS08g0440500 | | Os08g0440500 | |  | | - | |
| OS08T0440800-01 |  | OS08g0440800 | | Os08g0440800 | | Up | | -Up | |
| OS08T0444100-01 |  | OS08g0444100 | | Os08g0444100 | |  | | - | |
| OS08T0447000-01 | Down | OS08g0447000 | | Os08g0447000 | |  | | Down- | |
| OS08T0447300-00 |  | OS08g0447300 | | Os08g0447300 | |  | | - | |
| OS08T0451000-01 |  | OS08g0451000 | | Os08g0451000 | |  | | - | |
| OS08T0453716-00 |  | OS08g0453716 | | Os08g0453716 | |  | | - | |
| OS08T0455800-02 |  | OS08g0455800 | | Os08g0455800 | |  | | - | |
| OS08T0459300-02 |  | OS08g0459300 | | Os08g0459300 | |  | | - | |
| OS08T0459600-01 |  | OS08g0459600 | | Os08g0459600 | |  | | - | |
| OS08T0463900-01 |  | OS08g0463900 | | Os08g0463900 | |  | | - | |
| OS08T0464000-02 |  | OS08g0464000 | | Os08g0464000 | |  | | - | |
| OS08T0465800-02 |  | OS08g0465800 | | Os08g0465800 | |  | | - | |
| OS08T0467600-00 |  | OS08g0467600 | | Os08g0467600 | |  | | - | |
| OS08T0468100-01 |  | OS08g0468100 | | Os08g0468100 | | Up | | -Up | |
| OS08T0471950-00 |  | OS08g0471950 | | Os08g0471950 | |  | | - | |
| OS08T0474700-01 |  | OS08g0474700 | | Os08g0474700 | |  | | - | |
| OS08T0477800-01 |  | OS08g0477800 | | Os08g0477800 | |  | | - | |
| OS08T0478100-01 |  | OS08g0478100 | | Os08g0478100 | |  | | - | |
| OS08T0478200-01 | Up | OS08g0478200 | | Os08g0478200 | |  | | Up- | |
| OS08T0478500-01 |  | OS08g0478500 | | Os08g0478500 | |  | | - | |
| OS08T0480500-01 |  | OS08g0480500 | | Os08g0480500 | |  | | - | |
| OS08T0480800-01 |  | OS08g0480800 | | Os08g0480800 | |  | | - | |
| OS08T0481950-01 |  | OS08g0481950 | | Os08g0481950 | |  | | - | |
| OS08T0482100-01 |  | OS08g0482100 | | Os08g0482100 | |  | | - | |
| OS08T0483200-01 |  | OS08g0483200 | | Os08g0483200 | |  | | - | |
| OS08T0484500-01 | Down | OS08g0484500 | | Os08g0484500 | |  | | Down- | |
| OS08T0484600-01 |  | OS08g0484600 | | Os08g0484600 | |  | | - | |
| OS08T0485600-02 |  | OS08g0485600 | | Os08g0485600 | |  | | - | |
| OS08T0485900-01 |  | OS08g0485900 | | Os08g0485900 | | Down | | -Down | |
| OS08T0486200-02 |  | OS08g0486200 | | Os08g0486200 | |  | | - | |
| OS08T0487800-01 |  | OS08g0487800 | | Os08g0487800 | |  | | - | |
| OS08T0489100-00 |  | OS08g0489100 | | Os08g0489100 | |  | | - | |
| OS08T0490300-01 |  | OS08g0490300 | | Os08g0490300 | |  | | - | |
| OS08T0490900-00 |  | OS08g0490900 | | Os08g0490900 | |  | | - | |
| OS08T0492100-03 |  | OS08g0492100 | | Os08g0492100 | |  | | - | |
| OS08T0493900-02 |  | OS08g0493900 | | Os08g0493900 | |  | | - | |
| OS08T0495800-01 |  | OS08g0495800 | | Os08g0495800 | | Down | | -Down | |
| OS08T0496900-01 |  | OS08g0496900 | | Os08g0496900 | |  | | - | |
| OS08T0498100-01 |  | OS08g0498100 | | Os08g0498100 | | Up | | -Up | |
| OS08T0499300-02 |  | OS08g0499300 | | Os08g0499300 | | Down | | -Down | |
| OS08T0500100-01 |  | OS08g0500100 | | Os08g0500100 | |  | | - | |
| OS08T0500200-02 |  | OS08g0500200 | | Os08g0500200 | |  | | - | |
| OS08T0500700-01 | Up | OS08g0500700 | | Os08g0500700 | |  | | Up- | |
| OS08T0500900-01 |  | OS08g0500900 | | Os08g0500900 | |  | | - | |
| OS08T0502700-01 | Up | OS08g0502700 | | Os08g0502700 | |  | | Up- | |
| OS08T0502800-01 |  | OS08g0502800 | | Os08g0502800 | |  | | - | |
| OS08T0503200-01 |  | OS08g0503200 | | Os08g0503200 | |  | | - | |
| OS08T0504500-01 | Down | OS08g0504500 | | Os08g0504500 | | Down | | Down-Down | |
| OS08T0504600-01 |  | OS08g0504600 | | Os08g0504600 | |  | | - | |
| OS08T0506700-01 |  | OS08g0506700 | | Os08g0506700 | |  | | - | |
| OS08T0508800-01 |  | OS08g0508800 | | Os08g0508800 | |  | | - | |
| OS08T0509100-01 | Down | OS08g0509100 | | Os08g0509100 | | Down | | Down-Down | |
| OS08T0509200-02 |  | OS08g0509200 | | Os08g0509200 | | Down | | -Down | |
| OS08T0510400-01 |  | OS08g0510400 | | Os08g0510400 | |  | | - | |
| OS08T0511700-00 |  | OS08g0511700 | | Os08g0511700 | |  | | - | |
| OS08T0511900-01 |  | OS08g0511900 | | Os08g0511900 | | Up | | -Up | |
| OS08T0512400-02 |  | OS08g0512400 | | Os08g0512400 | |  | | - | |
| OS08T0512500-01 |  | OS08g0512500 | | Os08g0512500 | |  | | - | |
| OS08T0512900-01 |  | OS08g0512900 | | Os08g0512900 | |  | | - | |
| OS08T0513000-01 |  | OS08g0513000 | | Os08g0513000 | |  | | - | |
| OS08T0513300-02 |  | OS08g0513300 | | Os08g0513300 | |  | | - | |
| OS08T0516900-01 |  | OS08g0516900 | | Os08g0516900 | |  | | - | |
| OS08T0517300-01 |  | OS08g0517300 | | Os08g0517300 | |  | | - | |
| OS08T0517500-01 |  | OS08g0517500 | | Os08g0517500 | |  | | - | |
| OS08T0519400-01 |  | OS08g0519400 | | Os08g0519400 | |  | | - | |
| OS08T0519800-01 |  | OS08g0519800 | | Os08g0519800 | |  | | - | |
| OS08T0521400-01 |  | OS08g0521400 | | Os08g0521400 | |  | | - | |
| OS08T0522600-01 |  | OS08g0522600 | | Os08g0522600 | |  | | - | |
| OS08T0524200-01 |  | OS08g0524200 | | Os08g0524200 | |  | | - | |
| OS08T0524400-00 |  | OS08g0524400 | | Os08g0524400 | |  | | - | |
| OS08T0525600-01 |  | OS08g0525600 | | Os08g0525600 | |  | | - | |
| OS08T0525700-01 |  | OS08g0525700 | | Os08g0525700 | | Down | | -Down | |
| OS08T0526300-03 |  | OS08g0526300 | | Os08g0526300 | |  | | - | |
| OS08T0526400-01 |  | OS08g0526400 | | Os08g0526400 | |  | | - | |
| OS08T0528900-01 |  | OS08g0528900 | | Os08g0528900 | |  | | - | |
| OS08T0530000-01 |  | OS08g0530000 | | Os08g0530000 | |  | | - | |
| OS08T0530200-02 | Down | OS08g0530200 | | Os08g0530200 | |  | | Down- | |
| OS08T0530400-01 | Down | OS08g0530400 | | Os08g0530400 | |  | | Down- | |
| OS08T0531000-01 |  | OS08g0531000 | | Os08g0531000 | |  | | - | |
| OS08T0531300-01 |  | OS08g0531300 | | Os08g0531300 | | Up | | -Up | |
| OS08T0532200-03 |  | OS08g0532200 | | Os08g0532200 | |  | | - | |
| OS08T0532800-03 |  | OS08g0532800 | | Os08g0532800 | |  | | - | |
| OS08T0532900-02 |  | OS08g0532900 | | Os08g0532900 | |  | | - | |
| OS08T0534900-01 |  | OS08g0534900 | | Os08g0534900 | |  | | - | |
| OS08T0535600-01 | Up | OS08g0535600 | | Os08g0535600 | |  | | Up- | |
| OS08T0536000-01 | Up | OS08g0536000 | | Os08g0536000 | |  | | Up- | |
| OS08T0537800-01 |  | OS08g0537800 | | Os08g0537800 | |  | | - | |
| OS08T0538000-01 | Down | OS08g0538000 | | Os08g0538000 | |  | | Down- | |
| OS08T0538100-00 |  | OS08g0538100 | | Os08g0538100 | |  | | - | |
| OS08T0538300-01 |  | OS08g0538300 | | Os08g0538300 | |  | | - | |
| OS08T0538600-01 |  | OS08g0538600 | | Os08g0538600 | |  | | - | |
| OS08T0540100-02 |  | OS08g0540100 | | Os08g0540100 | |  | | - | |
| OS08T0540300-01 |  | OS08g0540300 | | Os08g0540300 | |  | | - | |
| OS08T0541400-02 |  | OS08g0541400 | | Os08g0541400 | | Down | | -Down | |
| OS08T0542100-01 |  | OS08g0542100 | | Os08g0542100 | |  | | - | |
| OS08T0542600-01 |  | OS08g0542600 | | Os08g0542600 | |  | | - | |
| OS08T0542900-01 |  | OS08g0542900 | | Os08g0542900 | |  | | - | |
| OS08T0543600-01 |  | OS08g0543600 | | Os08g0543600 | |  | | - | |
| OS08T0543900-00 |  | OS08g0543900 | | Os08g0543900 | |  | | - | |
| OS08T0545200-02 | Up | OS08g0545200 | | Os08g0545200 | |  | | Up- | |
| OS08T0547000-01 |  | OS08g0547000 | | Os08g0547000 | |  | | - | |
| OS08T0547800-01 |  | OS08g0547800 | | Os08g0547800 | |  | | - | |
| OS08T0547900-01 |  | OS08g0547900 | | Os08g0547900 | |  | | - | |
| OS08T0548900-01 |  | OS08g0548900 | | Os08g0548900 | |  | | - | |
| OS08T0549100-01 |  | OS08g0549100 | | Os08g0549100 | |  | | - | |
| OS08T0549300-01 |  | OS08g0549300 | | Os08g0549300 | |  | | - | |
| OS08T0550100-01 | Up | OS08g0550100 | | Os08g0550100 | |  | | Up- | |
| OS08T0550500-01 |  | OS08g0550500 | | Os08g0550500 | |  | | - | |
| OS08T0553800-05 |  | OS08g0553800 | | Os08g0553800 | |  | | - | |
| OS08T0554400-02 |  | OS08g0554400 | | Os08g0554400 | |  | | - | |
| OS08T0556900-02 |  | OS08g0556900 | | Os08g0556900 | | Down | | -Down | |
| OS08T0557000-01 | Up | OS08g0557000 | | Os08g0557000 | |  | | Up- | |
| OS08T0557100-01 |  | OS08g0557100 | | Os08g0557100 | |  | | - | |
| OS08T0557400-01 |  | OS08g0557400 | | Os08g0557400 | |  | | - | |
| OS08T0557500-02 |  | OS08g0557500 | | Os08g0557500 | |  | | - | |
| OS08T0557600-01 |  | OS08g0557600 | | Os08g0557600 | |  | | - | |
| OS08T0557900-01 |  | OS08g0557900 | | Os08g0557900 | |  | | - | |
| OS08T0558000-01 |  | OS08g0558000 | | Os08g0558000 | |  | | - | |
| OS08T0558200-01 |  | OS08g0558200 | | Os08g0558200 | |  | | - | |
| OS08T0558800-01 |  | OS08g0558800 | | Os08g0558800 | |  | | - | |
| OS08T0558900-02 |  | OS08g0558900 | | Os08g0558900 | |  | | - | |
| OS08T0559000-01 |  | OS08g0559000 | | Os08g0559000 | |  | | - | |
| OS08T0559200-01 | Down | OS08g0559200 | | Os08g0559200 | |  | | Down- | |
| OS08T0559400-01 |  | OS08g0559400 | | Os08g0559400 | |  | | - | |
| OS08T0559600-02 |  | OS08g0559600 | | Os08g0559600 | |  | | - | |
| OS08T0560500-01 |  | OS08g0560500 | | Os08g0560500 | |  | | - | |
| OS08T0560900-02 |  | OS08g0560900 | | Os08g0560900 | |  | | - | |
| OS08T0561600-01 |  | OS08g0561600 | | Os08g0561600 | |  | | - | |
| OS08T0561700-01 | Up | OS08g0561700 | | Os08g0561700 | |  | | Up- | |
| OS08T0562100-02 |  | OS08g0562100 | | Os08g0562100 | |  | | - | |
| OS08T0562600-03 | Down | OS08g0562600 | | Os08g0562600 | |  | | Down- | |
| OS08T0562700-01 |  | OS08g0562700 | | Os08g0562700 | |  | | - | |
| OS08T0564100-01 |  | OS08g0564100 | | Os08g0564100 | |  | | - | |
| OS08T0564800-01 |  | OS08g0564800 | | Os08g0564800 | |  | | - | |
| OS08T0566000-01 |  | OS08g0566000 | | Os08g0566000 | |  | | - | |
| OS08T0566400-01 |  | OS08g0566400 | | Os08g0566400 | |  | | - | |
| OS08T0567000-01 |  | OS08g0567000 | | Os08g0567000 | |  | | - | |
| OS08T0567200-02 |  | OS08g0567200 | | Os08g0567200 | |  | | - | |
| OS09T0104300-01 |  | OS09g0104300 | | Os09g0104300 | |  | | - | |
| OS09T0109600-01 |  | OS09g0109600 | | Os09g0109600 | | Up | | -Up | |
| OS09T0110300-02 |  | OS09g0110300 | | Os09g0110300 | | Up | | -Up | |
| OS09T0110400-03 |  | OS09g0110400 | | Os09g0110400 | |  | | - | |
| OS09T0115400-01 |  | OS09g0115400 | | Os09g0115400 | |  | | - | |
| OS09T0115500-02 |  | OS09g0115500 | | Os09g0115500 | |  | | - | |
| OS09T0115600-02 |  | OS09g0115600 | | Os09g0115600 | |  | | - | |
| OS09T0116400-02 |  | OS09g0116400 | | Os09g0116400 | |  | | - | |
| OS09T0121900-00 |  | OS09g0121900 | | Os09g0121900 | |  | | - | |
| OS09T0123100-01 |  | OS09g0123100 | | Os09g0123100 | |  | | - | |
| OS09T0123200-01 |  | OS09g0123200 | | Os09g0123200 | |  | | - | |
| OS09T0123300-01 |  | OS09g0123300 | | Os09g0123300 | |  | | - | |
| OS09T0127800-01 |  | OS09g0127800 | | Os09g0127800 | |  | | - | |
| OS09T0130800-02 |  | OS09g0130800 | | Os09g0130800 | |  | | - | |
| OS09T0132600-01 |  | OS09g0132600 | | Os09g0132600 | |  | | - | |
| OS09T0133200-02 |  | OS09g0133200 | | Os09g0133200 | |  | | - | |
| OS09T0133600-01 | Up | OS09g0133600 | | Os09g0133600 | |  | | Up- | |
| OS09T0135400-01 |  | OS09g0135400 | | Os09g0135400 | |  | | - | |
| OS09T0237600-01 |  | OS09g0237600 | | Os09g0237600 | |  | | - | |
| OS09T0244900-03 |  | OS09g0244900 | | Os09g0244900 | |  | | - | |
| OS09T0245500-01 |  | OS09g0245500 | | Os09g0245500 | |  | | - | |
| OS09T0248000-03 |  | OS09g0248000 | | Os09g0248000 | |  | | - | |
| OS09T0248100-03 |  | OS09g0248100 | | Os09g0248100 | |  | | - | |
| OS09T0249000-01 |  | OS09g0249000 | | Os09g0249000 | |  | | - | |
| OS09T0249600-02 |  | OS09g0249600 | | Os09g0249600 | |  | | - | |
| OS09T0249700-01 |  | OS09g0249700 | | Os09g0249700 | |  | | - | |
| OS09T0249900-01 |  | OS09g0249900 | | Os09g0249900 | |  | | - | |
| OS09T0250700-01 |  | OS09g0250700 | | Os09g0250700 | |  | | - | |
| OS09T0252100-01 |  | OS09g0252100 | | Os09g0252100 | |  | | - | |
| OS09T0253000-01 |  | OS09g0253000 | | Os09g0253000 | |  | | - | |
| OS09T0255000-01 | Up | OS09g0255000 | | Os09g0255000 | |  | | Up- | |
| OS09T0258000-01 |  | OS09g0258000 | | Os09g0258000 | |  | | - | |
| OS09T0258600-01 |  | OS09g0258600 | | Os09g0258600 | |  | | - | |
| OS09T0266000-03 |  | OS09g0266000 | | Os09g0266000 | |  | | - | |
| OS09T0267600-01 |  | OS09g0267600 | | Os09g0267600 | |  | | - | |
| OS09T0270900-02 |  | OS09g0270900 | | Os09g0270900 | |  | | - | |
| OS09T0272200-02 |  | OS09g0272200 | | Os09g0272200 | |  | | - | |
| OS09T0274900-01 |  | OS09g0274900 | | Os09g0274900 | |  | | - | |
| OS09T0277800-01 |  | OS09g0277800 | | Os09g0277800 | |  | | - | |
| OS09T0279000-01 |  | OS09g0279000 | | Os09g0279000 | |  | | - | |
| OS09T0279400-01 | Up | OS09g0279400 | | Os09g0279400 | |  | | Up- | |
| OS09T0279500-03 | Down | OS09g0279500 | | Os09g0279500 | | Down | | Down-Down | |
| OS09T0279600-01 |  | OS09g0279600 | | Os09g0279600 | |  | | - | |
| OS09T0281600-01 |  | OS09g0281600 | | Os09g0281600 | |  | | - | |
| OS09T0287300-01 |  | OS09g0287300 | | Os09g0287300 | |  | | - | |
| OS09T0294000-02 | Down | OS09g0294000 | | Os09g0294000 | | Down | | Down-Down | |
| OS09T0296800-01 |  | OS09g0296800 | | Os09g0296800 | |  | | - | |
| OS09T0297100-02 |  | OS09g0297100 | | Os09g0297100 | |  | | - | |
| OS09T0298200-02 |  | OS09g0298200 | | Os09g0298200 | | Up | | -Up | |
| OS09T0298400-01 |  | OS09g0298400 | | Os09g0298400 | |  | | - | |
| OS09T0298700-01 |  | OS09g0298700 | | Os09g0298700 | |  | | - | |
| OS09T0306700-01 |  | OS09g0306700 | | Os09g0306700 | |  | | - | |
| OS09T0308900-02 |  | OS09g0308900 | | Os09g0308900 | |  | | - | |
| OS09T0315700-01 | Up | OS09g0315700 | | Os09g0315700 | |  | | Up- | |
| OS09T0326900-01 |  | OS09g0326900 | | Os09g0326900 | |  | | - | |
| OS09T0327300-01 |  | OS09g0327300 | | Os09g0327300 | |  | | - | |
| OS09T0327400-01 | Up | OS09g0327400 | | Os09g0327400 | | Up | | Up-Up | |
| OS09T0327550-00 |  | OS09g0327550 | | Os09g0327550 | |  | | - | |
| OS09T0338400-02 |  | OS09g0338400 | | Os09g0338400 | |  | | - | |
| OS09T0339000-01 |  | OS09g0339000 | | Os09g0339000 | |  | | - | |
| OS09T0343200-01 |  | OS09g0343200 | | Os09g0343200 | | Down | | -Down | |
| OS09T0344500-01 |  | OS09g0344500 | | Os09g0344500 | | Up | | -Up | |
| OS09T0346400-01 |  | OS09g0346400 | | Os09g0346400 | |  | | - | |
| OS09T0346500-04 | Down | OS09g0346500 | | Os09g0346500 | |  | | Down- | |
| OS09T0347500-01 |  | OS09g0347500 | | Os09g0347500 | |  | | - | |
| OS09T0347800-01 |  | OS09g0347800 | | Os09g0347800 | |  | | - | |
| OS09T0352400-02 |  | OS09g0352400 | | Os09g0352400 | |  | | - | |
| OS09T0355400-01 |  | OS09g0355400 | | Os09g0355400 | |  | | - | |
| OS09T0359800-01 |  | OS09g0359800 | | Os09g0359800 | |  | | - | |
| OS09T0361400-01 |  | OS09g0361400 | | Os09g0361400 | |  | | - | |
| OS09T0362500-01 |  | OS09g0362500 | | Os09g0362500 | |  | | - | |
| OS09T0363600-01 |  | OS09g0363600 | | Os09g0363600 | |  | | - | |
| OS09T0363700-01 |  | OS09g0363700 | | Os09g0363700 | |  | | - | |
| OS09T0363800-00 | Up | OS09g0363800 | | Os09g0363800 | |  | | Up- | |
| OS09T0364000-01 |  | OS09g0364000 | | Os09g0364000 | |  | | - | |
| OS09T0370200-01 |  | OS09g0370200 | | Os09g0370200 | |  | | - | |
| OS09T0375100-01 |  | OS09g0375100 | | Os09g0375100 | |  | | - | |
| OS09T0375400-01 |  | OS09g0375400 | | Os09g0375400 | |  | | - | |
| OS09T0378300-01 |  | OS09g0378300 | | Os09g0378300 | |  | | - | |
| OS09T0379900-02 |  | OS09g0379900 | | Os09g0379900 | |  | | - | |
| OS09T0380000-02 |  | OS09g0380000 | | Os09g0380000 | |  | | - | |
| OS09T0380200-01 |  | OS09g0380200 | | Os09g0380200 | |  | | - | |
| OS09T0382400-01 |  | OS09g0382400 | | Os09g0382400 | | Down | | -Down | |
| OS09T0382500-01 |  | OS09g0382500 | | Os09g0382500 | |  | | - | |
| OS09T0386500-01 |  | OS09g0386500 | | Os09g0386500 | |  | | - | |
| OS09T0394900-01 |  | OS09g0394900 | | Os09g0394900 | |  | | - | |
| OS09T0396300-01 |  | OS09g0396300 | | Os09g0396300 | |  | | - | |
| OS09T0397700-02 |  | OS09g0397700 | | Os09g0397700 | |  | | - | |
| OS09T0400000-00 |  | OS09g0400000 | | Os09g0400000 | |  | | - | |
| OS09T0400500-01 |  | OS09g0400500 | | Os09g0400500 | |  | | - | |
| OS09T0402100-01 |  | OS09g0402100 | | Os09g0402100 | |  | | - | |
| OS09T0406300-00 |  | OS09g0406300 | | Os09g0406300 | |  | | - | |
| OS09T0407900-01 |  | OS09g0407900 | | Os09g0407900 | |  | | - | |
| OS09T0407950-01 |  | OS09g0407950 | | Os09g0407950 | |  | | - | |
| OS09T0408600-01 |  | OS09g0408600 | | Os09g0408600 | |  | | - | |
| OS09T0411650-01 |  | OS09g0411650 | | Os09g0411650 | |  | | - | |
| OS09T0411700-03 |  | OS09g0411700 | | Os09g0411700 | |  | | - | |
| OS09T0413500-01 |  | OS09g0413500 | | Os09g0413500 | |  | | - | |
| OS09T0413700-02 |  | OS09g0413700 | | Os09g0413700 | |  | | - | |
| OS09T0414600-02 |  | OS09g0414600 | | Os09g0414600 | |  | | - | |
| OS09T0415800-01 |  | OS09g0415800 | | Os09g0415800 | |  | | - | |
| OS09T0416200-01 |  | OS09g0416200 | | Os09g0416200 | |  | | - | |
| OS09T0419200-02 |  | OS09g0419200 | | Os09g0419200 | |  | | - | |
| OS09T0420600-01 |  | OS09g0420600 | | Os09g0420600 | |  | | - | |
| OS09T0420800-01 |  | OS09g0420800 | | Os09g0420800 | |  | | - | |
| OS09T0422000-01 |  | OS09g0422000 | | Os09g0422000 | |  | | - | |
| OS09T0423600-01 |  | OS09g0423600 | | Os09g0423600 | |  | | - | |
| OS09T0423700-01 |  | OS09g0423700 | | Os09g0423700 | |  | | - | |
| OS09T0425900-01 |  | OS09g0425900 | | Os09g0425900 | |  | | - | |
| OS09T0431500-02 |  | OS09g0431500 | | Os09g0431500 | |  | | - | |
| OS09T0436900-01 |  | OS09g0436900 | | Os09g0436900 | |  | | - | |
| OS09T0438700-01 |  | OS09g0438700 | | Os09g0438700 | |  | | - | |
| OS09T0439500-01 | Down | OS09g0439500 | | Os09g0439500 | | Down | | Down-Down | |
| OS09T0440300-02 |  | OS09g0440300 | | Os09g0440300 | | Up | | -Up | |
| OS09T0441625-00 |  | OS09g0441625 | | Os09g0441625 | |  | | - | |
| OS09T0442300-01 | Up | OS09g0442300 | | Os09g0442300 | | Up | | Up-Up | |
| OS09T0444200-01 |  | OS09g0444200 | | Os09g0444200 | |  | | - | |
| OS09T0446800-02 |  | OS09g0446800 | | Os09g0446800 | | Down | | -Down | |
| OS09T0451500-02 |  | OS09g0451500 | | Os09g0451500 | |  | | - | |
| OS09T0451700-00 |  | OS09g0451700 | | Os09g0451700 | |  | | - | |
| OS09T0453500-01 |  | OS09g0453500 | | Os09g0453500 | |  | | - | |
| OS09T0453800-02 |  | OS09g0453800 | | Os09g0453800 | |  | | - | |
| OS09T0454600-00 |  | OS09g0454600 | | Os09g0454600 | |  | | - | |
| OS09T0455400-01 |  | OS09g0455400 | | Os09g0455400 | |  | | - | |
| OS09T0458400-01 |  | OS09g0458400 | | Os09g0458400 | |  | | - | |
| OS09T0459800-02 |  | OS09g0459800 | | Os09g0459800 | |  | | - | |
| OS09T0460300-01 |  | OS09g0460300 | | Os09g0460300 | |  | | - | |
| OS09T0460400-01 |  | OS09g0460400 | | Os09g0460400 | |  | | - | |
| OS09T0460500-00 |  | OS09g0460500 | | Os09g0460500 | |  | | - | |
| OS09T0460700-01 |  | OS09g0460700 | | Os09g0460700 | |  | | - | |
| OS09T0461900-01 |  | OS09g0461900 | | Os09g0461900 | |  | | - | |
| OS09T0462200-01 |  | OS09g0462200 | | Os09g0462200 | |  | | - | |
| OS09T0463700-01 |  | OS09g0463700 | | Os09g0463700 | |  | | - | |
| OS09T0464000-02 |  | OS09g0464000 | | Os09g0464000 | | Up | | -Up | |
| OS09T0464400-00 |  | OS09g0464400 | | Os09g0464400 | |  | | - | |
| OS09T0465600-01 |  | OS09g0465600 | | Os09g0465600 | |  | | - | |
| OS09T0467100-01 |  | OS09g0467100 | | Os09g0467100 | |  | | - | |
| OS09T0467200-01 | Up | OS09g0467200 | | Os09g0467200 | |  | | Up- | |
| OS09T0469400-01 |  | OS09g0469400 | | Os09g0469400 | |  | | - | |
| OS09T0471100-01 |  | OS09g0471100 | | Os09g0471100 | | Down | | -Down | |
| OS09T0471900-01 |  | OS09g0471900 | | Os09g0471900 | |  | | - | |
| OS09T0474300-01 | Down | OS09g0474300 | | Os09g0474300 | |  | | Down- | |
| OS09T0476100-01 |  | OS09g0476100 | | Os09g0476100 | |  | | - | |
| OS09T0477900-01 |  | OS09g0477900 | | Os09g0477900 | |  | | - | |
| OS09T0478100-02 |  | OS09g0478100 | | Os09g0478100 | | Up | | -Up | |
| OS09T0478300-01 | Up | OS09g0478300 | | Os09g0478300 | | Up | | Up-Up | |
| OS09T0480700-01 |  | OS09g0480700 | | Os09g0480700 | |  | | - | |
| OS09T0482100-01 |  | OS09g0482100 | | Os09g0482100 | |  | | - | |
| OS09T0482660-01 |  | OS09g0482660 | | Os09g0482660 | |  | | - | |
| OS09T0482680-01 |  | OS09g0482680 | | Os09g0482680 | |  | | - | |
| OS09T0485201-00 |  | OS09g0485201 | | Os09g0485201 | |  | | - | |
| OS09T0485900-01 | Down | OS09g0485900 | | Os09g0485900 | |  | | Down- | |
| OS09T0487500-01 |  | OS09g0487500 | | Os09g0487500 | |  | | - | |
| OS09T0487600-01 |  | OS09g0487600 | | Os09g0487600 | |  | | - | |
| OS09T0487700-01 |  | OS09g0487700 | | Os09g0487700 | |  | | - | |
| OS09T0491644-01 |  | OS09g0491644 | | Os09g0491644 | |  | | - | |
| OS09T0491676-02 |  | OS09g0491676 | | Os09g0491676 | |  | | - | |
| OS09T0491708-02 | Up | OS09g0491708 | | Os09g0491708 | |  | | Up- | |
| OS09T0491820-01 |  | OS09g0491820 | | Os09g0491820 | |  | | - | |
| OS09T0491852-01 | Up | OS09g0491852 | | Os09g0491852 | |  | | Up- | |
| OS09T0494300-01 |  | OS09g0494300 | | Os09g0494300 | |  | | - | |
| OS09T0497400-02 |  | OS09g0497400 | | Os09g0497400 | |  | | - | |
| OS09T0498500-00 |  | OS09g0498500 | | Os09g0498500 | | Up | | -Up | |
| OS09T0500200-01 |  | OS09g0500200 | | Os09g0500200 | |  | | - | |
| OS09T0501200-01 |  | OS09g0501200 | | Os09g0501200 | |  | | - | |
| OS09T0501700-00 |  | OS09g0501700 | | Os09g0501700 | |  | | - | |
| OS09T0501850-01 |  | OS09g0501850 | | Os09g0501850 | |  | | - | |
| OS09T0502200-01 |  | OS09g0502200 | | Os09g0502200 | |  | | - | |
| OS09T0502500-01 |  | OS09g0502500 | | Os09g0502500 | |  | | - | |
| OS09T0503100-01 |  | OS09g0503100 | | Os09g0503100 | |  | | - | |
| OS09T0503400-01 | Down | OS09g0503400 | | Os09g0503400 | |  | | Down- | |
| OS09T0505600-01 |  | OS09g0505600 | | Os09g0505600 | |  | | - | |
| OS09T0505700-01 |  | OS09g0505700 | | Os09g0505700 | |  | | - | |
| OS09T0506000-02 |  | OS09g0506000 | | Os09g0506000 | | Up | | -Up | |
| OS09T0507800-01 |  | OS09g0507800 | | Os09g0507800 | |  | | - | |
| OS09T0508900-01 |  | OS09g0508900 | | Os09g0508900 | |  | | - | |
| OS09T0509000-01 |  | OS09g0509000 | | Os09g0509000 | |  | | - | |
| OS09T0509200-02 |  | OS09g0509200 | | Os09g0509200 | |  | | - | |
| OS09T0509300-01 |  | OS09g0509300 | | Os09g0509300 | |  | | - | |
| OS09T0511600-04 |  | OS09g0511600 | | Os09g0511600 | | Up | | -Up | |
| OS09T0512800-00 |  | OS09g0512800 | | Os09g0512800 | |  | | - | |
| OS09T0512900-03 |  | OS09g0512900 | | Os09g0512900 | |  | | - | |
| OS09T0513000-01 |  | OS09g0513000 | | Os09g0513000 | |  | | - | |
| OS09T0513600-01 |  | OS09g0513600 | | Os09g0513600 | |  | | - | |
| OS09T0514100-02 |  | OS09g0514100 | | Os09g0514100 | |  | | - | |
| OS09T0514600-00 |  | OS09g0514600 | | Os09g0514600 | |  | | - | |
| OS09T0515100-01 |  | OS09g0515100 | | Os09g0515100 | |  | | - | |
| OS09T0515200-02 |  | OS09g0515200 | | Os09g0515200 | |  | | - | |
| OS09T0515500-01 |  | OS09g0515500 | | Os09g0515500 | |  | | - | |
| OS09T0516200-01 |  | OS09g0516200 | | Os09g0516200 | |  | | - | |
| OS09T0516300-01 |  | OS09g0516300 | | Os09g0516300 | |  | | - | |
| OS09T0516600-02 |  | OS09g0516600 | | Os09g0516600 | |  | | - | |
| OS09T0517000-01 |  | OS09g0517000 | | Os09g0517000 | |  | | - | |
| OS09T0517700-01 | Up | OS09g0517700 | | Os09g0517700 | |  | | Up- | |
| OS09T0518700-02 |  | OS09g0518700 | | Os09g0518700 | |  | | - | |
| OS09T0520800-01 |  | OS09g0520800 | | Os09g0520800 | |  | | - | |
| OS09T0521500-01 |  | OS09g0521500 | | Os09g0521500 | |  | | - | |
| OS09T0525900-01 |  | OS09g0525900 | | Os09g0525900 | |  | | - | |
| OS09T0526700-02 |  | OS09g0526700 | | Os09g0526700 | |  | | - | |
| OS09T0528300-01 |  | OS09g0528300 | | Os09g0528300 | |  | | - | |
| OS09T0529100-01 |  | OS09g0529100 | | Os09g0529100 | |  | | - | |
| OS09T0529900-01 |  | OS09g0529900 | | Os09g0529900 | |  | | - | |
| OS09T0530000-02 |  | OS09g0530000 | | Os09g0530000 | |  | | - | |
| OS09T0530300-01 | Up | OS09g0530300 | | Os09g0530300 | | Up | | Up-Up | |
| OS09T0530700-01 |  | OS09g0530700 | | Os09g0530700 | |  | | - | |
| OS09T0531100-01 |  | OS09g0531100 | | Os09g0531100 | |  | | - | |
| OS09T0532700-01 | Down | OS09g0532700 | | Os09g0532700 | |  | | Down- | |
| OS09T0533100-01 |  | OS09g0533100 | | Os09g0533100 | |  | | - | |
| OS09T0533300-01 |  | OS09g0533300 | | Os09g0533300 | |  | | - | |
| OS09T0533400-01 |  | OS09g0533400 | | Os09g0533400 | |  | | - | |
| OS09T0534800-01 |  | OS09g0534800 | | Os09g0534800 | |  | | - | |
| OS09T0535000-02 |  | OS09g0535000 | | Os09g0535000 | |  | | - | |
| OS09T0535300-01 |  | OS09g0535300 | | Os09g0535300 | |  | | - | |
| OS09T0535900-02 | Up | OS09g0535900 | | Os09g0535900 | |  | | Up- | |
| OS09T0537700-02 |  | OS09g0537700 | | Os09g0537700 | | Down | | -Down | |
| OS09T0538000-01 |  | OS09g0538000 | | Os09g0538000 | |  | | - | |
| OS09T0538200-01 |  | OS09g0538200 | | Os09g0538200 | |  | | - | |
| OS09T0539100-01 |  | OS09g0539100 | | Os09g0539100 | |  | | - | |
| OS09T0540500-01 |  | OS09g0540500 | | Os09g0540500 | |  | | - | |
| OS09T0540600-01 |  | OS09g0540600 | | Os09g0540600 | |  | | - | |
| OS09T0541000-02 |  | OS09g0541000 | | Os09g0541000 | |  | | - | |
| OS09T0541700-02 | Up | OS09g0541700 | | Os09g0541700 | |  | | Up- | |
| OS09T0541900-02 |  | OS09g0541900 | | Os09g0541900 | |  | | - | |
| OS09T0542000-01 |  | OS09g0542000 | | Os09g0542000 | |  | | - | |
| OS09T0542100-01 | Down | OS09g0542100 | | Os09g0542100 | |  | | Down- | |
| OS09T0542200-02 |  | OS09g0542200 | | Os09g0542200 | |  | | - | |
| OS09T0543100-02 |  | OS09g0543100 | | Os09g0543100 | |  | | - | |
| OS09T0544900-01 |  | OS09g0544900 | | Os09g0544900 | |  | | - | |
| OS09T0548200-01 |  | OS09g0548200 | | Os09g0548200 | |  | | - | |
| OS09T0550000-01 |  | OS09g0550000 | | Os09g0550000 | |  | | - | |
| OS09T0551300-01 |  | OS09g0551300 | | Os09g0551300 | |  | | - | |
| OS09T0551600-01 |  | OS09g0551600 | | Os09g0551600 | |  | | - | |
| OS09T0553200-01 |  | OS09g0553200 | | Os09g0553200 | |  | | - | |
| OS09T0553300-01 |  | OS09g0553300 | | Os09g0553300 | |  | | - | |
| OS09T0553600-01 |  | OS09g0553600 | | Os09g0553600 | |  | | - | |
| OS09T0553900-01 |  | OS09g0553900 | | Os09g0553900 | | Up | | -Up | |
| OS09T0556000-01 |  | OS09g0556000 | | Os09g0556000 | |  | | - | |
| OS09T0556200-00 |  | OS09g0556200 | | Os09g0556200 | |  | | - | |
| OS09T0557800-01 |  | OS09g0557800 | | Os09g0557800 | |  | | - | |
| OS09T0558000-01 |  | OS09g0558000 | | Os09g0558000 | |  | | - | |
| OS09T0560000-01 |  | OS09g0560000 | | Os09g0560000 | |  | | - | |
| OS09T0560400-01 |  | OS09g0560400 | | Os09g0560400 | |  | | - | |
| OS09T0560700-02 |  | OS09g0560700 | | Os09g0560700 | |  | | - | |
| OS09T0563200-01 |  | OS09g0563200 | | Os09g0563200 | |  | | - | |
| OS09T0565200-02 | Down | OS09g0565200 | | Os09g0565200 | | Down | | Down-Down | |
| OS09T0565400-01 |  | OS09g0565400 | | Os09g0565400 | |  | | - | |
| OS09T0565450-00 |  | OS09g0565450 | | Os09g0565450 | |  | | - | |
| OS09T0566100-02 |  | OS09g0566100 | | Os09g0566100 | |  | | - | |
| OS09T0567000-01 |  | OS09g0567000 | | Os09g0567000 | |  | | - | |
| OS09T0567300-01 |  | OS09g0567300 | | Os09g0567300 | |  | | - | |
| OS09T0567400-02 |  | OS09g0567400 | | Os09g0567400 | |  | | - | |
| OS09T0567700-01 |  | OS09g0567700 | | Os09g0567700 | |  | | - | |
| OS09T0568400-02 |  | OS09g0568400 | | Os09g0568400 | |  | | - | |
| OS09T0568900-01 | Up | OS09g0568900 | | Os09g0568900 | |  | | Up- | |
| OS09T0569400-01 |  | OS09g0569400 | | Os09g0569400 | |  | | - | |
| OS09T0570300-02 |  | OS09g0570300 | | Os09g0570300 | |  | | - | |
| OS09T0570400-01 |  | OS09g0570400 | | Os09g0570400 | |  | | - | |
| OS09T0571200-01 |  | OS09g0571200 | | Os09g0571200 | |  | | - | |
| OS09T0571400-01 |  | OS09g0571400 | | Os09g0571400 | |  | | - | |
| OS09T0572400-01 |  | OS09g0572400 | | Os09g0572400 | | Up | | -Up | |
| OS09T0572700-02 |  | OS09g0572700 | | Os09g0572700 | |  | | - | |
| OS09T0572900-01 |  | OS09g0572900 | | Os09g0572900 | |  | | - | |
| OS09T0573200-03 |  | OS09g0573200 | | Os09g0573200 | |  | | - | |
| OS10T0100300-01 | Down | OS10g0100300 | | Os10g0100300 | | Down | | Down-Down | |
| OS10T0100700-01 |  | OS10g0100700 | | Os10g0100700 | |  | | - | |
| OS10T0101200-01 | Up | OS10g0101200 | | Os10g0101200 | |  | | Up- | |
| OS10T0104700-01 |  | OS10g0104700 | | Os10g0104700 | |  | | - | |
| OS10T0113900-02 | Up | OS10g0113900 | | Os10g0113900 | |  | | Up- | |
| OS10T0114300-01 |  | OS10g0114300 | | Os10g0114300 | | Up | | -Up | |
| OS10T0115600-02 |  | OS10g0115600 | | Os10g0115600 | |  | | - | |
| OS10T0135600-01 |  | OS10g0135600 | | Os10g0135600 | |  | | - | |
| OS10T0136400-00 |  | OS10g0136400 | | Os10g0136400 | |  | | - | |
| OS10T0140200-01 |  | OS10g0140200 | | Os10g0140200 | |  | | - | |
| OS10T0147900-02 |  | OS10g0147900 | | Os10g0147900 | |  | | - | |
| OS10T0154000-03 |  | OS10g0154000 | | Os10g0154000 | |  | | - | |
| OS10T0155500-01 |  | OS10g0155500 | | Os10g0155500 | |  | | - | |
| OS10T0159600-01 |  | OS10g0159600 | | Os10g0159600 | |  | | - | |
| OS10T0159800-04 |  | OS10g0159800 | | Os10g0159800 | |  | | - | |
| OS10T0163340-01 | Up | OS10g0163340 | | Os10g0163340 | |  | | Up- | |
| OS10T0167300-02 |  | OS10g0167300 | | Os10g0167300 | |  | | - | |
| OS10T0170000-01 |  | OS10g0170000 | | Os10g0170000 | |  | | - | |
| OS10T0177200-02 |  | OS10g0177200 | | Os10g0177200 | |  | | - | |
| OS10T0178500-01 | Up | OS10g0178500 | | Os10g0178500 | | Up | | Up-Up | |
| OS10T0181200-01 |  | OS10g0181200 | | Os10g0181200 | |  | | - | |
| OS10T0181600-02 |  | OS10g0181600 | | Os10g0181600 | |  | | - | |
| OS10T0182000-01 |  | OS10g0182000 | | Os10g0182000 | |  | | - | |
| OS10T0183900-02 |  | OS10g0183900 | | Os10g0183900 | |  | | - | |
| OS10T0188100-03 |  | OS10g0188100 | | Os10g0188100 | |  | | - | |
| OS10T0188900-01 |  | OS10g0188900 | | Os10g0188900 | |  | | - | |
| OS10T0189100-01 |  | OS10g0189100 | | Os10g0189100 | |  | | - | |
| OS10T0190000-02 |  | OS10g0190000 | | Os10g0190000 | |  | | - | |
| OS10T0190800-01 |  | OS10g0190800 | | Os10g0190800 | |  | | - | |
| OS10T0194200-01 |  | OS10g0194200 | | Os10g0194200 | |  | | - | |
| OS10T0205200-02 |  | OS10g0205200 | | Os10g0205200 | |  | | - | |
| OS10T0214132-00 |  | OS10g0214132 | | Os10g0214132 | |  | | - | |
| OS10T0320100-01 |  | OS10g0320100 | | Os10g0320100 | | Up | | -Up | |
| OS10T0320400-01 | Up | OS10g0320400 | | Os10g0320400 | |  | | Up- | |
| OS10T0321700-01 |  | OS10g0321700 | | Os10g0321700 | |  | | - | |
| OS10T0329300-03 |  | OS10g0329300 | | Os10g0329300 | |  | | - | |
| OS10T0330000-01 | Down | OS10g0330000 | | Os10g0330000 | |  | | Down- | |
| OS10T0339600-01 |  | OS10g0339600 | | Os10g0339600 | |  | | - | |
| OS10T0341700-02 |  | OS10g0341700 | | Os10g0341700 | |  | | - | |
| OS10T0346600-00 |  | OS10g0346600 | | Os10g0346600 | |  | | - | |
| OS10T0351700-01 |  | OS10g0351700 | | Os10g0351700 | |  | | - | |
| OS10T0355800-00 |  | OS10g0355800 | | Os10g0355800 | |  | | - | |
| OS10T0356000-00 |  | OS10g0356000 | | Os10g0356000 | |  | | - | |
| OS10T0365200-00 |  | OS10g0365200 | | Os10g0365200 | |  | | - | |
| OS10T0365800-01 |  | OS10g0365800 | | Os10g0365800 | |  | | - | |
| OS10T0369000-01 |  | OS10g0369000 | | Os10g0369000 | |  | | - | |
| OS10T0369600-01 |  | OS10g0369600 | | Os10g0369600 | |  | | - | |
| OS10T0370000-02 |  | OS10g0370000 | | Os10g0370000 | |  | | - | |
| OS10T0370500-01 |  | OS10g0370500 | | Os10g0370500 | |  | | - | |
| OS10T0375600-01 |  | OS10g0375600 | | Os10g0375600 | |  | | - | |
| OS10T0377400-02 |  | OS10g0377400 | | Os10g0377400 | |  | | - | |
| OS10T0377800-03 |  | OS10g0377800 | | Os10g0377800 | |  | | - | |
| OS10T0389200-01 |  | OS10g0389200 | | Os10g0389200 | | Up | | -Up | |
| OS10T0389300-01 |  | OS10g0389300 | | Os10g0389300 | | Up | | -Up | |
| OS10T0390500-01 |  | OS10g0390500 | | Os10g0390500 | |  | | - | |
| OS10T0390500-02 |  | OS10g0390500 | | Os10g0390500 | |  | | - | |
| OS10T0392700-02 |  | OS10g0392700 | | Os10g0392700 | |  | | - | |
| OS10T0394100-01 |  | OS10g0394100 | | Os10g0394100 | |  | | - | |
| OS10T0395200-01 |  | OS10g0395200 | | Os10g0395200 | |  | | - | |
| OS10T0397200-02 |  | OS10g0397200 | | Os10g0397200 | |  | | - | |
| OS10T0400100-01 |  | OS10g0400100 | | Os10g0400100 | |  | | - | |
| OS10T0400800-01 |  | OS10g0400800 | | Os10g0400800 | |  | | - | |
| OS10T0403700-00 |  | OS10g0403700 | | Os10g0403700 | |  | | - | |
| OS10T0405600-01 |  | OS10g0405600 | | Os10g0405600 | |  | | - | |
| OS10T0406600-01 |  | OS10g0406600 | | Os10g0406600 | |  | | - | |
| OS10T0407000-01 |  | OS10g0407000 | | Os10g0407000 | |  | | - | |
| OS10T0410700-01 |  | OS10g0410700 | | Os10g0410700 | |  | | - | |
| OS10T0411800-01 |  | OS10g0411800 | | Os10g0411800 | |  | | - | |
| OS10T0412100-03 |  | OS10g0412100 | | Os10g0412100 | |  | | - | |
| OS10T0414500-01 |  | OS10g0414500 | | Os10g0414500 | |  | | - | |
| OS10T0415600-03 |  | OS10g0415600 | | Os10g0415600 | |  | | - | |
| OS10T0415900-01 |  | OS10g0415900 | | Os10g0415900 | |  | | - | |
| OS10T0416100-02 |  | OS10g0416100 | | Os10g0416100 | |  | | - | |
| OS10T0416800-03 |  | OS10g0416800 | | Os10g0416800 | |  | | - | |
| OS10T0417600-01 | Up | OS10g0417600 | | Os10g0417600 | |  | | Up- | |
| OS10T0419100-01 |  | OS10g0419100 | | Os10g0419100 | |  | | - | |
| OS10T0419300-00 |  | OS10g0419300 | | Os10g0419300 | |  | | - | |
| OS10T0419600-00 |  | OS10g0419600 | | Os10g0419600 | |  | | - | |
| OS10T0430200-03 | Down | OS10g0430200 | | Os10g0430200 | |  | | Down- | |
| OS10T0431000-03 |  | OS10g0431000 | | Os10g0431000 | |  | | - | |
| OS10T0431900-02 |  | OS10g0431900 | | Os10g0431900 | |  | | - | |
| OS10T0436800-01 | Up | OS10g0436800 | | Os10g0436800 | |  | | Up- | |
| OS10T0437600-00 |  | OS10g0437600 | | Os10g0437600 | |  | | - | |
| OS10T0438000-00 |  | OS10g0438000 | | Os10g0438000 | |  | | - | |
| OS10T0439600-01 |  | OS10g0439600 | | Os10g0439600 | |  | | - | |
| OS10T0442600-01 |  | OS10g0442600 | | Os10g0442600 | |  | | - | |
| OS10T0443200-01 |  | OS10g0443200 | | Os10g0443200 | |  | | - | |
| OS10T0444700-02 |  | OS10g0444700 | | Os10g0444700 | |  | | - | |
| OS10T0445500-01 |  | OS10g0445500 | | Os10g0445500 | |  | | - | |
| OS10T0445600-01 |  | OS10g0445600 | | Os10g0445600 | |  | | - | |
| OS10T0447100-02 |  | OS10g0447100 | | Os10g0447100 | |  | | - | |
| OS10T0447600-01 |  | OS10g0447600 | | Os10g0447600 | |  | | - | |
| OS10T0456100-00 |  | OS10g0456100 | | Os10g0456100 | | Up | | -Up | |
| OS10T0456200-01 |  | OS10g0456200 | | Os10g0456200 | |  | | - | |
| OS10T0456500-02 |  | OS10g0456500 | | Os10g0456500 | |  | | - | |
| OS10T0457000-01 |  | OS10g0457000 | | Os10g0457000 | |  | | - | |
| OS10T0457500-01 |  | OS10g0457500 | | Os10g0457500 | |  | | - | |
| OS10T0457600-01 |  | OS10g0457600 | | Os10g0457600 | |  | | - | |
| OS10T0462900-02 | Up | OS10g0462900 | | Os10g0462900 | |  | | Up- | |
| OS10T0463800-01 | Up | OS10g0463800 | | Os10g0463800 | | Up | | Up-Up | |
| OS10T0464100-01 |  | OS10g0464100 | | Os10g0464100 | |  | | - | |
| OS10T0464400-01 |  | OS10g0464400 | | Os10g0464400 | |  | | - | |
| OS10T0465800-00 |  | OS10g0465800 | | Os10g0465800 | |  | | - | |
| OS10T0465900-01 |  | OS10g0465900 | | Os10g0465900 | |  | | - | |
| OS10T0466300-02 |  | OS10g0466300 | | Os10g0466300 | |  | | - | |
| OS10T0466700-01 |  | OS10g0466700 | | Os10g0466700 | |  | | - | |
| OS10T0467600-01 |  | OS10g0467600 | | Os10g0467600 | |  | | - | |
| OS10T0470900-01 |  | OS10g0470900 | | Os10g0470900 | |  | | - | |
| OS10T0471300-01 | Up | OS10g0471300 | | Os10g0471300 | |  | | Up- | |
| OS10T0471350-01 |  | OS10g0471350 | | Os10g0471350 | |  | | - | |
| OS10T0476000-02 |  | OS10g0476000 | | Os10g0476000 | |  | | - | |
| OS10T0476600-02 |  | OS10g0476600 | | Os10g0476600 | |  | | - | |
| OS10T0477800-01 |  | OS10g0477800 | | Os10g0477800 | |  | | - | |
| OS10T0478200-01 |  | OS10g0478200 | | Os10g0478200 | |  | | - | |
| OS10T0479600-01 |  | OS10g0479600 | | Os10g0479600 | |  | | - | |
| OS10T0481400-01 |  | OS10g0481400 | | Os10g0481400 | |  | | - | |
| OS10T0483000-01 |  | OS10g0483000 | | Os10g0483000 | |  | | - | |
| OS10T0484900-00 |  | OS10g0484900 | | Os10g0484900 | |  | | - | |
| OS10T0485000-01 |  | OS10g0485000 | | Os10g0485000 | |  | | - | |
| OS10T0485600-00 |  | OS10g0485600 | | Os10g0485600 | |  | | - | |
| OS10T0486900-01 |  | OS10g0486900 | | Os10g0486900 | |  | | - | |
| OS10T0488100-01 |  | OS10g0488100 | | Os10g0488100 | |  | | - | |
| OS10T0489200-01 |  | OS10g0489200 | | Os10g0489200 | |  | | - | |
| OS10T0491000-01 | Up | OS10g0491000 | | Os10g0491000 | | Down | | Up-Down | |
| OS10T0492000-01 |  | OS10g0492000 | | Os10g0492000 | |  | | - | |
| OS10T0492200-01 |  | OS10g0492200 | | Os10g0492200 | |  | | - | |
| OS10T0492800-01 |  | OS10g0492800 | | Os10g0492800 | |  | | - | |
| OS10T0492900-01 |  | OS10g0492900 | | Os10g0492900 | | Up | | -Up | |
| OS10T0493600-03 |  | OS10g0493600 | | Os10g0493600 | | Down | | -Down | |
| OS10T0494500-01 |  | OS10g0494500 | | Os10g0494500 | |  | | - | |
| OS10T0495000-01 |  | OS10g0495000 | | Os10g0495000 | |  | | - | |
| OS10T0495300-01 |  | OS10g0495300 | | Os10g0495300 | |  | | - | |
| OS10T0498000-01 |  | OS10g0498000 | | Os10g0498000 | | Up | | -Up | |
| OS10T0498200-01 |  | OS10g0498200 | | Os10g0498200 | |  | | - | |
| OS10T0498300-01 |  | OS10g0498300 | | Os10g0498300 | |  | | - | |
| OS10T0498600-01 |  | OS10g0498600 | | Os10g0498600 | |  | | - | |
| OS10T0500500-01 |  | OS10g0500500 | | Os10g0500500 | |  | | - | |
| OS10T0500600-01 |  | OS10g0500600 | | Os10g0500600 | |  | | - | |
| OS10T0502000-01 |  | OS10g0502000 | | Os10g0502000 | | Down | | -Down | |
| OS10T0502400-01 |  | OS10g0502400 | | Os10g0502400 | |  | | - | |
| OS10T0502500-04 |  | OS10g0502500 | | Os10g0502500 | |  | | - | |
| OS10T0502600-02 |  | OS10g0502600 | | Os10g0502600 | |  | | - | |
| OS10T0503500-01 | Up | OS10g0503500 | | Os10g0503500 | |  | | Up- | |
| OS10T0503700-01 |  | OS10g0503700 | | Os10g0503700 | |  | | - | |
| OS10T0503800-01 |  | OS10g0503800 | | Os10g0503800 | |  | | - | |
| OS10T0505700-01 |  | OS10g0505700 | | Os10g0505700 | |  | | - | |
| OS10T0505900-01 |  | OS10g0505900 | | Os10g0505900 | |  | | - | |
| OS10T0507800-01 |  | OS10g0507800 | | Os10g0507800 | |  | | - | |
| OS10T0508700-01 |  | OS10g0508700 | | Os10g0508700 | | Down | | -Down | |
| OS10T0509000-01 |  | OS10g0509000 | | Os10g0509000 | |  | | - | |
| OS10T0509200-01 | Up | OS10g0509200 | | Os10g0509200 | |  | | Up- | |
| OS10T0510000-01 |  | OS10g0510000 | | Os10g0510000 | |  | | - | |
| OS10T0511400-01 | Up | OS10g0511400 | | Os10g0511400 | |  | | Up- | |
| OS10T0511600-01 |  | OS10g0511600 | | Os10g0511600 | |  | | - | |
| OS10T0515200-01 |  | OS10g0515200 | | Os10g0515200 | |  | | - | |
| OS10T0516100-02 |  | OS10g0516100 | | Os10g0516100 | | Down | | -Down | |
| OS10T0517400-01 |  | OS10g0517400 | | Os10g0517400 | |  | | - | |
| OS10T0518800-01 |  | OS10g0518800 | | Os10g0518800 | |  | | - | |
| OS10T0520600-02 |  | OS10g0520600 | | Os10g0520600 | |  | | - | |
| OS10T0522500-02 |  | OS10g0522500 | | Os10g0522500 | |  | | - | |
| OS10T0525000-02 |  | OS10g0525000 | | Os10g0525000 | | Down | | -Down | |
| OS10T0525400-03 |  | OS10g0525400 | | Os10g0525400 | |  | | - | |
| OS10T0527800-01 |  | OS10g0527800 | | Os10g0527800 | |  | | - | |
| OS10T0528400-01 |  | OS10g0528400 | | Os10g0528400 | |  | | - | |
| OS10T0530400-01 | Up | OS10g0530400 | | Os10g0530400 | |  | | Up- | |
| OS10T0530900-01 |  | OS10g0530900 | | Os10g0530900 | |  | | - | |
| OS10T0531400-01 |  | OS10g0531400 | | Os10g0531400 | |  | | - | |
| OS10T0533200-01 | Up | OS10g0533200 | | Os10g0533200 | |  | | Up- | |
| OS10T0533600-01 |  | OS10g0533600 | | Os10g0533600 | |  | | - | |
| OS10T0534500-02 |  | OS10g0534500 | | Os10g0534500 | |  | | - | |
| OS10T0536400-01 | Up | OS10g0536400 | | Os10g0536400 | |  | | Up- | |
| OS10T0537300-01 |  | OS10g0537300 | | Os10g0537300 | |  | | - | |
| OS10T0538200-01 |  | OS10g0538200 | | Os10g0538200 | |  | | - | |
| OS10T0539500-01 |  | OS10g0539500 | | Os10g0539500 | |  | | - | |
| OS10T0539700-01 |  | OS10g0539700 | | Os10g0539700 | |  | | - | |
| OS10T0539900-02 |  | OS10g0539900 | | Os10g0539900 | |  | | - | |
| OS10T0542200-01 |  | OS10g0542200 | | Os10g0542200 | |  | | - | |
| OS10T0542900-01 | Up | OS10g0542900 | | Os10g0542900 | | Down | | Up-Down | |
| OS10T0543500-01 |  | OS10g0543500 | | Os10g0543500 | |  | | - | |
| OS10T0543800-00 |  | OS10g0543800 | | Os10g0543800 | |  | | - | |
| OS10T0545300-01 |  | OS10g0545300 | | Os10g0545300 | |  | | - | |
| OS10T0545700-03 |  | OS10g0545700 | | Os10g0545700 | |  | | - | |
| OS10T0546600-01 |  | OS10g0546600 | | Os10g0546600 | | Down | | -Down | |
| OS10T0548400-00 |  | OS10g0548400 | | Os10g0548400 | |  | | - | |
| OS10T0548800-01 |  | OS10g0548800 | | Os10g0548800 | |  | | - | |
| OS10T0548900-01 |  | OS10g0548900 | | Os10g0548900 | | Down | | -Down | |
| OS10T0551600-01 |  | OS10g0551600 | | Os10g0551600 | |  | | - | |
| OS10T0552900-01 |  | OS10g0552900 | | Os10g0552900 | |  | | - | |
| OS10T0553800-01 |  | OS10g0553800 | | Os10g0553800 | | Up | | -Up | |
| OS10T0555900-02 |  | OS10g0555900 | | Os10g0555900 | | Down | | -Down | |
| OS10T0558700-02 | Up | OS10g0558700 | | Os10g0558700 | |  | | Up- | |
| OS10T0559600-01 |  | OS10g0559600 | | Os10g0559600 | |  | | - | |
| OS10T0559700-02 |  | OS10g0559700 | | Os10g0559700 | |  | | - | |
| OS10T0561100-01 |  | OS10g0561100 | | Os10g0561100 | |  | | - | |
| OS10T0561400-01 |  | OS10g0561400 | | Os10g0561400 | |  | | - | |
| OS10T0563000-01 |  | OS10g0563000 | | Os10g0563000 | |  | | - | |
| OS10T0563400-01 |  | OS10g0563400 | | Os10g0563400 | |  | | - | |
| OS10T0563600-01 |  | OS10g0563600 | | Os10g0563600 | |  | | - | |
| OS10T0563700-01 |  | OS10g0563700 | | Os10g0563700 | | Down | | -Down | |
| OS10T0564000-01 |  | OS10g0564000 | | Os10g0564000 | |  | | - | |
| OS10T0564300-01 |  | OS10g0564300 | | Os10g0564300 | |  | | - | |
| OS10T0564500-01 |  | OS10g0564500 | | Os10g0564500 | |  | | - | |
| OS10T0565300-01 |  | OS10g0565300 | | Os10g0565300 | |  | | - | |
| OS10T0567000-01 |  | OS10g0567000 | | Os10g0567000 | | Up | | -Up | |
| OS10T0567800-01 |  | OS10g0567800 | | Os10g0567800 | |  | | - | |
| OS10T0568900-01 | Up | OS10g0568900 | | Os10g0568900 | |  | | Up- | |
| OS10T0569200-01 |  | OS10g0569200 | | Os10g0569200 | |  | | - | |
| OS10T0571100-01 |  | OS10g0571100 | | Os10g0571100 | |  | | - | |
| OS10T0572300-01 |  | OS10g0572300 | | Os10g0572300 | |  | | - | |
| OS10T0573400-01 |  | OS10g0573400 | | Os10g0573400 | |  | | - | |
| OS10T0573700-02 | Down | OS10g0573700 | | Os10g0573700 | |  | | Down- | |
| OS10T0573900-01 |  | OS10g0573900 | | Os10g0573900 | |  | | - | |
| OS10T0574800-01 |  | OS10g0574800 | | Os10g0574800 | |  | | - | |
| OS10T0575000-01 |  | OS10g0575000 | | Os10g0575000 | |  | | - | |
| OS10T0575200-01 |  | OS10g0575200 | | Os10g0575200 | |  | | - | |
| OS10T0575700-00 |  | OS10g0575700 | | Os10g0575700 | |  | | - | |
| OS10T0576100-01 |  | OS10g0576100 | | Os10g0576100 | |  | | - | |
| OS10T0577700-01 |  | OS10g0577700 | | Os10g0577700 | |  | | - | |
| OS10T0577900-01 |  | OS10g0577900 | | Os10g0577900 | |  | | - | |
| OS10T0579300-01 |  | OS10g0579300 | | Os10g0579300 | |  | | - | |
| OS10T0580300-01 |  | OS10g0580300 | | Os10g0580300 | |  | | - | |
| OS10T0580700-01 |  | OS10g0580700 | | Os10g0580700 | |  | | - | |
| OS10T0580800-01 |  | OS10g0580800 | | Os10g0580800 | |  | | - | |
| OS11T0102700-01 |  | OS11g0102700 | | Os11g0102700 | |  | | - | |
| OS11T0104050-01 |  | OS11g0104050 | | Os11g0104050 | |  | | - | |
| OS11T0105750-01 | Up | OS11g0105750 | | Os11g0105750 | |  | | Up- | |
| OS11T0106200-01 |  | OS11g0106200 | | Os11g0106200 | |  | | - | |
| OS11T0106400-01 |  | OS11g0106400 | | Os11g0106400 | |  | | - | |
| OS11T0109933-01 |  | OS11g0109933 | | Os11g0109933 | |  | | - | |
| OS11T0110100-01 |  | OS11g0110100 | | Os11g0110100 | |  | | - | |
| OS11T0115350-01 |  | OS11g0115350 | | Os11g0115350 | |  | | - | |
| OS11T0118300-01 |  | OS11g0118300 | | Os11g0118300 | |  | | - | |
| OS11T0128800-01 |  | OS11g0128800 | | Os11g0128800 | |  | | - | |
| OS11T0131900-01 |  | OS11g0131900 | | Os11g0131900 | |  | | - | |
| OS11T0132400-01 |  | OS11g0132400 | | Os11g0132400 | |  | | - | |
| OS11T0132600-02 |  | OS11g0132600 | | Os11g0132600 | |  | | - | |
| OS11T0133600-01 |  | OS11g0133600 | | Os11g0133600 | |  | | - | |
| OS11T0134500-01 |  | OS11g0134500 | | Os11g0134500 | |  | | - | |
| OS11T0137300-01 |  | OS11g0137300 | | Os11g0137300 | |  | | - | |
| OS11T0141000-01 |  | OS11g0141000 | | Os11g0141000 | |  | | - | |
| OS11T0142500-00 |  | OS11g0142500 | | Os11g0142500 | |  | | - | |
| OS11T0143500-01 |  | OS11g0143500 | | Os11g0143500 | |  | | - | |
| OS11T0145400-01 |  | OS11g0145400 | | Os11g0145400 | |  | | - | |
| OS11T0146700-02 |  | OS11g0146700 | | Os11g0146700 | |  | | - | |
| OS11T0147000-01 |  | OS11g0147000 | | Os11g0147000 | |  | | - | |
| OS11T0147800-01 |  | OS11g0147800 | | Os11g0147800 | |  | | - | |
| OS11T0148500-02 |  | OS11g0148500 | | Os11g0148500 | |  | | - | |
| OS11T0150100-02 |  | OS11g0150100 | | Os11g0150100 | |  | | - | |
| OS11T0151002-00 |  | OS11g0151002 | | Os11g0151002 | |  | | - | |
| OS11T0153800-01 |  | OS11g0153800 | | Os11g0153800 | |  | | - | |
| OS11T0158600-00 |  | OS11g0158600 | | Os11g0158600 | |  | | - | |
| OS11T0160500-01 |  | OS11g0160500 | | Os11g0160500 | |  | | - | |
| OS11T0162200-01 |  | OS11g0162200 | | Os11g0162200 | |  | | - | |
| OS11T0163100-01 |  | OS11g0163100 | | Os11g0163100 | |  | | - | |
| OS11T0163800-01 |  | OS11g0163800 | | Os11g0163800 | |  | | - | |
| OS11T0167800-01 |  | OS11g0167800 | | Os11g0167800 | |  | | - | |
| OS11T0168200-02 |  | OS11g0168200 | | Os11g0168200 | |  | | - | |
| OS11T0169600-01 |  | OS11g0169600 | | Os11g0169600 | |  | | - | |
| OS11T0171300-01 |  | OS11g0171300 | | Os11g0171300 | |  | | - | |
| OS11T0171500-01 |  | OS11g0171500 | | Os11g0171500 | |  | | - | |
| OS11T0174000-03 |  | OS11g0174000 | | Os11g0174000 | |  | | - | |
| OS11T0175400-01 |  | OS11g0175400 | | Os11g0175400 | | Down | | -Down | |
| OS11T0175900-01 |  | OS11g0175900 | | Os11g0175900 | |  | | - | |
| OS11T0176200-01 |  | OS11g0176200 | | Os11g0176200 | |  | | - | |
| OS11T0178800-01 |  | OS11g0178800 | | Os11g0178800 | |  | | - | |
| OS11T0180000-01 |  | OS11g0180000 | | Os11g0180000 | |  | | - | |
| OS11T0181100-01 |  | OS11g0181100 | | Os11g0181100 | |  | | - | |
| OS11T0181500-01 |  | OS11g0181500 | | Os11g0181500 | | Up | | -Up | |
| OS11T0181700-02 |  | OS11g0181700 | | Os11g0181700 | |  | | - | |
| OS11T0182200-02 |  | OS11g0182200 | | Os11g0182200 | |  | | - | |
| OS11T0183700-01 |  | OS11g0183700 | | Os11g0183700 | |  | | - | |
| OS11T0183900-02 | Up | OS11g0183900 | | Os11g0183900 | |  | | Up- | |
| OS11T0186200-01 | Up | OS11g0186200 | | Os11g0186200 | |  | | Up- | |
| OS11T0186400-00 |  | OS11g0186400 | | Os11g0186400 | |  | | - | |
| OS11T0191400-02 |  | OS11g0191400 | | Os11g0191400 | |  | | - | |
| OS11T0194800-01 |  | OS11g0194800 | | Os11g0194800 | |  | | - | |
| OS11T0199200-01 |  | OS11g0199200 | | Os11g0199200 | |  | | - | |
| OS11T0199600-02 |  | OS11g0199600 | | Os11g0199600 | |  | | - | |
| OS11T0199700-02 |  | OS11g0199700 | | Os11g0199700 | |  | | - | |
| OS11T0205500-02 |  | OS11g0205500 | | Os11g0205500 | |  | | - | |
| OS11T0207000-01 |  | OS11g0207000 | | Os11g0207000 | |  | | - | |
| OS11T0210300-01 |  | OS11g0210300 | | Os11g0210300 | |  | | - | |
| OS11T0210600-01 |  | OS11g0210600 | | Os11g0210600 | | Down | | -Down | |
| OS11T0213600-01 |  | OS11g0213600 | | Os11g0213600 | |  | | - | |
| OS11T0215100-01 |  | OS11g0215100 | | Os11g0215100 | |  | | - | |
| OS11T0216000-02 |  | OS11g0216000 | | Os11g0216000 | |  | | - | |
| OS11T0216100-01 |  | OS11g0216100 | | Os11g0216100 | |  | | - | |
| OS11T0216300-01 | Up | OS11g0216300 | | Os11g0216300 | | Up | | Up-Up | |
| OS11T0216400-01 |  | OS11g0216400 | | Os11g0216400 | |  | | - | |
| OS11T0216900-03 | Down | OS11g0216900 | | Os11g0216900 | |  | | Down- | |
| OS11T0217300-01 |  | OS11g0217300 | | Os11g0217300 | |  | | - | |
| OS11T0218000-01 |  | OS11g0218000 | | Os11g0218000 | |  | | - | |
| OS11T0220300-01 |  | OS11g0220300 | | Os11g0220300 | |  | | - | |
| OS11T0220800-01 |  | OS11g0220800 | | Os11g0220800 | |  | | - | |
| OS11T0222000-01 |  | OS11g0222000 | | Os11g0222000 | |  | | - | |
| OS11T0226933-01 |  | OS11g0226933 | | Os11g0226933 | | Down | | -Down | |
| OS11T0227800-01 |  | OS11g0227800 | | Os11g0227800 | |  | | - | |
| OS11T0229500-01 |  | OS11g0229500 | | Os11g0229500 | |  | | - | |
| OS11T0234200-01 |  | OS11g0234200 | | Os11g0234200 | |  | | - | |
| OS11T0236100-01 |  | OS11g0236100 | | Os11g0236100 | |  | | - | |
| OS11T0240600-02 |  | OS11g0240600 | | Os11g0240600 | | Up | | -Up | |
| OS11T0242100-02 |  | OS11g0242100 | | Os11g0242100 | |  | | - | |
| OS11T0242400-01 |  | OS11g0242400 | | Os11g0242400 | |  | | - | |
| OS11T0242700-01 |  | OS11g0242700 | | Os11g0242700 | |  | | - | |
| OS11T0242800-01 |  | OS11g0242800 | | Os11g0242800 | | Down | | -Down | |
| OS11T0247300-01 |  | OS11g0247300 | | Os11g0247300 | |  | | - | |
| OS11T0248200-01 |  | OS11g0248200 | | Os11g0248200 | |  | | - | |
| OS11T0249900-02 |  | OS11g0249900 | | Os11g0249900 | |  | | - | |
| OS11T0250000-01 |  | OS11g0250000 | | Os11g0250000 | |  | | - | |
| OS11T0256000-01 |  | OS11g0256000 | | Os11g0256000 | |  | | - | |
| OS11T0256200-01 |  | OS11g0256200 | | Os11g0256200 | |  | | - | |
| OS11T0264600-01 |  | OS11g0264600 | | Os11g0264600 | |  | | - | |
| OS11T0265400-01 |  | OS11g0265400 | | Os11g0265400 | |  | | - | |
| OS11T0267000-03 |  | OS11g0267000 | | Os11g0267000 | |  | | - | |
| OS11T0269000-00 |  | OS11g0269000 | | Os11g0269000 | |  | | - | |
| OS11T0275000-00 |  | OS11g0275000 | | Os11g0275000 | |  | | - | |
| OS11T0276300-01 |  | OS11g0276300 | | Os11g0276300 | |  | | - | |
| OS11T0293900-02 |  | OS11g0293900 | | Os11g0293900 | |  | | - | |
| OS11T0303500-01 | Up | OS11g0303500 | | Os11g0303500 | |  | | Up- | |
| OS11T0311300-04 |  | OS11g0311300 | | Os11g0311300 | |  | | - | |
| OS11T0312400-01 |  | OS11g0312400 | | Os11g0312400 | |  | | - | |
| OS11T0414000-01 |  | OS11g0414000 | | Os11g0414000 | |  | | - | |
| OS11T0425300-02 |  | OS11g0425300 | | Os11g0425300 | |  | | - | |
| OS11T0425600-01 |  | OS11g0425600 | | Os11g0425600 | |  | | - | |
| OS11T0432400-02 |  | OS11g0432400 | | Os11g0432400 | |  | | - | |
| OS11T0432900-01 |  | OS11g0432900 | | Os11g0432900 | | Up | | -Up | |
| OS11T0433500-01 |  | OS11g0433500 | | Os11g0433500 | |  | | - | |
| OS11T0433600-01 |  | OS11g0433600 | | Os11g0433600 | |  | | - | |
| OS11T0433900-01 |  | OS11g0433900 | | Os11g0433900 | |  | | - | |
| OS11T0443700-01 |  | OS11g0443700 | | Os11g0443700 | |  | | - | |
| OS11T0444900-01 |  | OS11g0444900 | | Os11g0444900 | |  | | - | |
| OS11T0448700-01 |  | OS11g0448700 | | Os11g0448700 | |  | | - | |
| OS11T0455500-01 | Up | OS11g0455500 | | Os11g0455500 | |  | | Up- | |
| OS11T0455800-01 |  | OS11g0455800 | | Os11g0455800 | |  | | - | |
| OS11T0456300-01 |  | OS11g0456300 | | Os11g0456300 | |  | | - | |
| OS11T0472000-01 |  | OS11g0472000 | | Os11g0472000 | |  | | - | |
| OS11T0473100-01 |  | OS11g0473100 | | Os11g0473100 | |  | | - | |
| OS11T0473200-01 |  | OS11g0473200 | | Os11g0473200 | |  | | - | |
| OS11T0482100-01 |  | OS11g0482100 | | Os11g0482100 | |  | | - | |
| OS11T0482400-01 |  | OS11g0482400 | | Os11g0482400 | |  | | - | |
| OS11T0484000-01 |  | OS11g0484000 | | Os11g0484000 | |  | | - | |
| OS11T0484500-01 |  | OS11g0484500 | | Os11g0484500 | |  | | - | |
| OS11T0490800-01 |  | OS11g0490800 | | Os11g0490800 | |  | | - | |
| OS11T0491400-01 |  | OS11g0491400 | | Os11g0491400 | | Up | | -Up | |
| OS11T0497000-01 |  | OS11g0497000 | | Os11g0497000 | |  | | - | |
| OS11T0514800-01 |  | OS11g0514800 | | Os11g0514800 | |  | | - | |
| OS11T0521500-01 | Up | OS11g0521500 | | Os11g0521500 | |  | | Up- | |
| OS11T0521900-03 |  | OS11g0521900 | | Os11g0521900 | |  | | - | |
| OS11T0524300-01 |  | OS11g0524300 | | Os11g0524300 | |  | | - | |
| OS11T0525600-02 |  | OS11g0525600 | | Os11g0525600 | |  | | - | |
| OS11T0528200-01 |  | OS11g0528200 | | Os11g0528200 | |  | | - | |
| OS11T0528500-01 |  | OS11g0528500 | | Os11g0528500 | |  | | - | |
| OS11T0533500-02 |  | OS11g0533500 | | Os11g0533500 | |  | | - | |
| OS11T0536800-02 |  | OS11g0536800 | | Os11g0536800 | |  | | - | |
| OS11T0538900-01 | Up | OS11g0538900 | | Os11g0538900 | |  | | Up- | |
| OS11T0539800-01 |  | OS11g0539800 | | Os11g0539800 | |  | | - | |
| OS11T0544100-00 |  | OS11g0544100 | | Os11g0544100 | |  | | - | |
| OS11T0544600-01 |  | OS11g0544600 | | Os11g0544600 | |  | | - | |
| OS11T0544700-01 |  | OS11g0544700 | | Os11g0544700 | |  | | - | |
| OS11T0544800-01 | Down | OS11g0544800 | | Os11g0544800 | | Down | | Down-Down | |
| OS11T0545600-01 |  | OS11g0545600 | | Os11g0545600 | |  | | - | |
| OS11T0546000-02 |  | OS11g0546000 | | Os11g0546000 | |  | | - | |
| OS11T0546300-01 |  | OS11g0546300 | | Os11g0546300 | |  | | - | |
| OS11T0546900-01 | Up | OS11g0546900 | | Os11g0546900 | |  | | Up- | |
| OS11T0549900-01 |  | OS11g0549900 | | Os11g0549900 | | Up | | -Up | |
| OS11T0557900-01 |  | OS11g0557900 | | Os11g0557900 | |  | | - | |
| OS11T0568500-01 |  | OS11g0568500 | | Os11g0568500 | |  | | - | |
| OS11T0568600-01 |  | OS11g0568600 | | Os11g0568600 | |  | | - | |
| OS11T0570400-01 |  | OS11g0570400 | | Os11g0570400 | |  | | - | |
| OS11T0572100-03 |  | OS11g0572100 | | Os11g0572100 | |  | | - | |
| OS11T0572700-00 |  | OS11g0572700 | | Os11g0572700 | |  | | - | |
| OS11T0585700-01 |  | OS11g0585700 | | Os11g0585700 | |  | | - | |
| OS11T0586300-01 |  | OS11g0586300 | | Os11g0586300 | | Up | | -Up | |
| OS11T0587500-01 |  | OS11g0587500 | | Os11g0587500 | |  | | - | |
| OS11T0588300-02 |  | OS11g0588300 | | Os11g0588300 | |  | | - | |
| OS11T0591100-01 | Up | OS11g0591100 | | Os11g0591100 | |  | | Up- | |
| OS11T0592000-01 |  | OS11g0592000 | | Os11g0592000 | | Down | | -Down | |
| OS11T0592100-01 |  | OS11g0592100 | | Os11g0592100 | |  | | - | |
| OS11T0592200-01 |  | OS11g0592200 | | Os11g0592200 | | Down | | -Down | |
| OS11T0592700-00 |  | OS11g0592700 | | Os11g0592700 | |  | | - | |
| OS11T0592900-01 |  | OS11g0592900 | | Os11g0592900 | |  | | - | |
| OS11T0594200-01 |  | OS11g0594200 | | Os11g0594200 | |  | | - | |
| OS11T0595200-01 |  | OS11g0595200 | | Os11g0595200 | |  | | - | |
| OS11T0599200-01 |  | OS11g0599200 | | Os11g0599200 | |  | | - | |
| OS11T0599500-00 |  | OS11g0599500 | | Os11g0599500 | |  | | - | |
| OS11T0602200-01 |  | OS11g0602200 | | Os11g0602200 | |  | | - | |
| OS11T0603200-01 |  | OS11g0603200 | | Os11g0603200 | | Up | | -Up | |
| OS11T0605500-01 |  | OS11g0605500 | | Os11g0605500 | |  | | - | |
| OS11T0607200-02 |  | OS11g0607200 | | Os11g0607200 | |  | | - | |
| OS11T0610700-01 |  | OS11g0610700 | | Os11g0610700 | |  | | - | |
| OS11T0610900-01 |  | OS11g0610900 | | Os11g0610900 | |  | | - | |
| OS11T0615100-01 |  | OS11g0615100 | | Os11g0615100 | |  | | - | |
| OS11T0615700-02 |  | OS11g0615700 | | Os11g0615700 | |  | | - | |
| OS11T0616200-01 |  | OS11g0616200 | | Os11g0616200 | |  | | - | |
| OS11T0620100-01 |  | OS11g0620100 | | Os11g0620100 | |  | | - | |
| OS11T0621500-01 |  | OS11g0621500 | | Os11g0621500 | |  | | - | |
| OS11T0629200-01 |  | OS11g0629200 | | Os11g0629200 | |  | | - | |
| OS11T0629500-01 |  | OS11g0629500 | | Os11g0629500 | |  | | - | |
| OS11T0636900-02 |  | OS11g0636900 | | Os11g0636900 | |  | | - | |
| OS11T0637700-02 |  | OS11g0637700 | | Os11g0637700 | |  | | - | |
| OS11T0638000-01 |  | OS11g0638000 | | Os11g0638000 | |  | | - | |
| OS11T0642800-01 |  | OS11g0642800 | | Os11g0642800 | |  | | - | |
| OS11T0643700-01 |  | OS11g0643700 | | Os11g0643700 | |  | | - | |
| OS11T0644100-00 |  | OS11g0644100 | | Os11g0644100 | |  | | - | |
| OS11T0644600-00 | Up | OS11g0644600 | | Os11g0644600 | |  | | Up- | |
| OS11T0644700-01 |  | OS11g0644700 | | Os11g0644700 | |  | | - | |
| OS11T0645200-01 |  | OS11g0645200 | | Os11g0645200 | |  | | - | |
| OS11T0645400-01 |  | OS11g0645400 | | Os11g0645400 | |  | | - | |
| OS11T0652100-01 |  | OS11g0652100 | | Os11g0652100 | |  | | - | |
| OS11T0653300-01 |  | OS11g0653300 | | Os11g0653300 | |  | | - | |
| OS11T0657100-01 |  | OS11g0657100 | | Os11g0657100 | |  | | - | |
| OS11T0658800-01 | Up | OS11g0658800 | | Os11g0658800 | | Up | | Up-Up | |
| OS11T0660300-01 |  | OS11g0660300 | | Os11g0660300 | |  | | - | |
| OS11T0660500-01 |  | OS11g0660500 | | Os11g0660500 | |  | | - | |
| OS11T0661300-01 |  | OS11g0661300 | | Os11g0661300 | |  | | - | |
| OS11T0671000-01 |  | OS11g0671000 | | Os11g0671000 | | Down | | -Down | |
| OS11T0673200-01 |  | OS11g0673200 | | Os11g0673200 | |  | | - | |
| OS11T0674400-00 |  | OS11g0674400 | | Os11g0674400 | |  | | - | |
| OS11T0683500-02 |  | OS11g0683500 | | Os11g0683500 | |  | | - | |
| OS11T0690332-00 |  | OS11g0690332 | | Os11g0690332 | |  | | - | |
| OS11T0691500-00 |  | OS11g0691500 | | Os11g0691500 | | Up | | -Up | |
| OS11T0704300-02 |  | OS11g0704300 | | Os11g0704300 | |  | | - | |
| OS11T0704600-03 |  | OS11g0704600 | | Os11g0704600 | |  | | - | |
| OS11T0707000-02 |  | OS11g0707000 | | Os11g0707000 | |  | | - | |
| OS11T0707700-01 |  | OS11g0707700 | | Os11g0707700 | |  | | - | |
| OS11T0707800-01 |  | OS11g0707800 | | Os11g0707800 | |  | | - | |
| OS11T0708400-02 |  | OS11g0708400 | | Os11g0708400 | |  | | - | |
| OS12T0102100-01 |  | OS12g0102100 | | Os12g0102100 | |  | | - | |
| OS12T0102350-01 |  | OS12g0102350 | | Os12g0102350 | |  | | - | |
| OS12T0102500-01 |  | OS12g0102500 | | Os12g0102500 | |  | | - | |
| OS12T0104766-00 |  | OS12g0104766 | | Os12g0104766 | |  | | - | |
| OS12T0105300-03 |  | OS12g0105300 | | Os12g0105300 | | Up | | -Up | |
| OS12T0106000-01 | Up | OS12g0106000 | | Os12g0106000 | | Up | | Up-Up | |
| OS12T0107500-01 |  | OS12g0107500 | | Os12g0107500 | |  | | - | |
| OS12T0109600-01 |  | OS12g0109600 | | Os12g0109600 | |  | | - | |
| OS12T0109900-01 |  | OS12g0109900 | | Os12g0109900 | |  | | - | |
| OS12T0112200-01 |  | OS12g0112200 | | Os12g0112200 | |  | | - | |
| OS12T0115000-00 |  | OS12g0115000 | | Os12g0115000 | |  | | - | |
| OS12T0115100-00 | Up | OS12g0115100 | | Os12g0115100 | |  | | Up- | |
| OS12T0115300-01 | Up | OS12g0115300 | | Os12g0115300 | | Down | | Up-Down | |
| OS12T0115500-01 |  | OS12g0115500 | | Os12g0115500 | |  | | - | |
| OS12T0115900-01 |  | OS12g0115900 | | Os12g0115900 | |  | | - | |
| OS12T0118400-02 |  | OS12g0118400 | | Os12g0118400 | |  | | - | |
| OS12T0121300-01 |  | OS12g0121300 | | Os12g0121300 | |  | | - | |
| OS12T0124000-04 |  | OS12g0124000 | | Os12g0124000 | |  | | - | |
| OS12T0124200-01 |  | OS12g0124200 | | Os12g0124200 | |  | | - | |
| OS12T0125400-01 |  | OS12g0125400 | | Os12g0125400 | |  | | - | |
| OS12T0126800-01 |  | OS12g0126800 | | Os12g0126800 | |  | | - | |
| OS12T0128600-01 |  | OS12g0128600 | | Os12g0128600 | |  | | - | |
| OS12T0129400-01 |  | OS12g0129400 | | Os12g0129400 | |  | | - | |
| OS12T0131000-01 |  | OS12g0131000 | | Os12g0131000 | |  | | - | |
| OS12T0133050-00 |  | OS12g0133050 | | Os12g0133050 | |  | | - | |
| OS12T0134200-01 |  | OS12g0134200 | | Os12g0134200 | |  | | - | |
| OS12T0136200-03 |  | OS12g0136200 | | Os12g0136200 | |  | | - | |
| OS12T0137200-01 |  | OS12g0137200 | | Os12g0137200 | |  | | - | |
| OS12T0138900-02 |  | OS12g0138900 | | Os12g0138900 | |  | | - | |
| OS12T0139600-01 |  | OS12g0139600 | | Os12g0139600 | |  | | - | |
| OS12T0143900-01 |  | OS12g0143900 | | Os12g0143900 | |  | | - | |
| OS12T0145100-01 |  | OS12g0145100 | | Os12g0145100 | | Up | | -Up | |
| OS12T0145700-01 |  | OS12g0145700 | | Os12g0145700 | |  | | - | |
| OS12T0150100-01 |  | OS12g0150100 | | Os12g0150100 | |  | | - | |
| OS12T0152700-02 |  | OS12g0152700 | | Os12g0152700 | |  | | - | |
| OS12T0155300-01 |  | OS12g0155300 | | Os12g0155300 | |  | | - | |
| OS12T0156400-01 |  | OS12g0156400 | | Os12g0156400 | |  | | - | |
| OS12T0157200-01 | Up | OS12g0157200 | | Os12g0157200 | |  | | Up- | |
| OS12T0159600-02 |  | OS12g0159600 | | Os12g0159600 | |  | | - | |
| OS12T0160500-01 |  | OS12g0160500 | | Os12g0160500 | |  | | - | |
| OS12T0162500-01 |  | OS12g0162500 | | Os12g0162500 | |  | | - | |
| OS12T0163500-01 |  | OS12g0163500 | | Os12g0163500 | |  | | - | |
| OS12T0164600-00 |  | OS12g0164600 | | Os12g0164600 | |  | | - | |
| OS12T0165000-01 |  | OS12g0165000 | | Os12g0165000 | |  | | - | |
| OS12T0165900-01 |  | OS12g0165900 | | Os12g0165900 | |  | | - | |
| OS12T0166000-00 |  | OS12g0166000 | | Os12g0166000 | |  | | - | |
| OS12T0168000-02 |  | OS12g0168000 | | Os12g0168000 | |  | | - | |
| OS12T0168700-02 |  | OS12g0168700 | | Os12g0168700 | |  | | - | |
| OS12T0169400-01 |  | OS12g0169400 | | Os12g0169400 | | Up | | -Up | |
| OS12T0169700-01 |  | OS12g0169700 | | Os12g0169700 | |  | | - | |
| OS12T0169800-00 |  | OS12g0169800 | | Os12g0169800 | |  | | - | |
| OS12T0170100-01 |  | OS12g0170100 | | Os12g0170100 | |  | | - | |
| OS12T0170700-01 |  | OS12g0170700 | | Os12g0170700 | |  | | - | |
| OS12T0171200-01 |  | OS12g0171200 | | Os12g0171200 | |  | | - | |
| OS12T0172500-01 |  | OS12g0172500 | | Os12g0172500 | |  | | - | |
| OS12T0173400-01 |  | OS12g0173400 | | Os12g0173400 | |  | | - | |
| OS12T0174700-00 |  | OS12g0174700 | | Os12g0174700 | |  | | - | |
| OS12T0175500-01 | Down | OS12g0175500 | | Os12g0175500 | |  | | Down- | |
| OS12T0176700-00 |  | OS12g0176700 | | Os12g0176700 | |  | | - | |
| OS12T0176800-01 |  | OS12g0176800 | | Os12g0176800 | |  | | - | |
| OS12T0177500-01 |  | OS12g0177500 | | Os12g0177500 | |  | | - | |
| OS12T0178100-01 |  | OS12g0178100 | | Os12g0178100 | |  | | - | |
| OS12T0178200-01 |  | OS12g0178200 | | Os12g0178200 | |  | | - | |
| OS12T0182200-01 |  | OS12g0182200 | | Os12g0182200 | |  | | - | |
| OS12T0182600-01 |  | OS12g0182600 | | Os12g0182600 | |  | | - | |
| OS12T0182700-01 |  | OS12g0182700 | | Os12g0182700 | |  | | - | |
| OS12T0182800-01 |  | OS12g0182800 | | Os12g0182800 | |  | | - | |
| OS12T0183100-03 |  | OS12g0183100 | | Os12g0183100 | |  | | - | |
| OS12T0183300-01 |  | OS12g0183300 | | Os12g0183300 | |  | | - | |
| OS12T0188700-02 |  | OS12g0188700 | | Os12g0188700 | |  | | - | |
| OS12T0189300-01 |  | OS12g0189300 | | Os12g0189300 | |  | | - | |
| OS12T0189400-01 |  | OS12g0189400 | | Os12g0189400 | |  | | - | |
| OS12T0190200-01 |  | OS12g0190200 | | Os12g0190200 | |  | | - | |
| OS12T0192500-02 |  | OS12g0192500 | | Os12g0192500 | |  | | - | |
| OS12T0193100-04 |  | OS12g0193100 | | Os12g0193100 | |  | | - | |
| OS12T0194700-01 |  | OS12g0194700 | | Os12g0194700 | |  | | - | |
| OS12T0198000-01 | Up | OS12g0198000 | | Os12g0198000 | | Up | | Up-Up | |
| OS12T0207500-00 |  | OS12g0207500 | | Os12g0207500 | |  | | - | |
| OS12T0207600-00 |  | OS12g0207600 | | Os12g0207600 | |  | | - | |
| OS12T0209000-00 |  | OS12g0209000 | | Os12g0209000 | |  | | - | |
| OS12T0209100-01 |  | OS12g0209100 | | Os12g0209100 | |  | | - | |
| OS12T0210300-01 |  | OS12g0210300 | | Os12g0210300 | |  | | - | |
| OS12T0210400-01 |  | OS12g0210400 | | Os12g0210400 | |  | | - | |
| OS12T0210800-01 |  | OS12g0210800 | | Os12g0210800 | |  | | - | |
| OS12T0222800-00 |  | OS12g0222800 | | Os12g0222800 | |  | | - | |
| OS12T0223300-01 |  | OS12g0223300 | | Os12g0223300 | |  | | - | |
| OS12T0225200-01 |  | OS12g0225200 | | Os12g0225200 | |  | | - | |
| OS12T0226700-01 | Up | OS12g0226700 | | Os12g0226700 | | Up | | Up-Up | |
| OS12T0230100-01 |  | OS12g0230100 | | Os12g0230100 | |  | | - | |
| OS12T0233400-01 |  | OS12g0233400 | | Os12g0233400 | |  | | - | |
| OS12T0235800-02 |  | OS12g0235800 | | Os12g0235800 | |  | | - | |
| OS12T0236400-01 |  | OS12g0236400 | | Os12g0236400 | |  | | - | |
| OS12T0236500-02 |  | OS12g0236500 | | Os12g0236500 | |  | | - | |
| OS12T0236900-01 |  | OS12g0236900 | | Os12g0236900 | |  | | - | |
| OS12T0238100-01 |  | OS12g0238100 | | Os12g0238100 | |  | | - | |
| OS12T0244100-01 | Down | OS12g0244100 | | Os12g0244100 | |  | | Down- | |
| OS12T0247700-01 | Down | OS12g0247700 | | Os12g0247700 | |  | | Down- | |
| OS12T0257000-01 |  | OS12g0257000 | | Os12g0257000 | |  | | - | |
| OS12T0263200-01 |  | OS12g0263200 | | Os12g0263200 | |  | | - | |
| OS12T0264500-01 |  | OS12g0264500 | | Os12g0264500 | |  | | - | |
| OS12T0265100-01 |  | OS12g0265100 | | Os12g0265100 | |  | | - | |
| OS12T0266000-01 |  | OS12g0266000 | | Os12g0266000 | |  | | - | |
| OS12T0270100-01 |  | OS12g0270100 | | Os12g0270100 | |  | | - | |
| OS12T0271700-02 |  | OS12g0271700 | | Os12g0271700 | | Up | | -Up | |
| OS12T0276100-01 |  | OS12g0276100 | | Os12g0276100 | |  | | - | |
| OS12T0277400-01 | Up | OS12g0277400 | | Os12g0277400 | |  | | Up- | |
| OS12T0277500-01 |  | OS12g0277500 | | Os12g0277500 | |  | | - | |
| OS12T0278800-02 |  | OS12g0278800 | | Os12g0278800 | |  | | - | |
| OS12T0283800-01 |  | OS12g0283800 | | Os12g0283800 | |  | | - | |
| OS12T0283900-02 |  | OS12g0283900 | | Os12g0283900 | |  | | - | |
| OS12T0285100-04 |  | OS12g0285100 | | Os12g0285100 | |  | | - | |
| OS12T0287200-01 |  | OS12g0287200 | | Os12g0287200 | |  | | - | |
| OS12T0287300-02 | Up | OS12g0287300 | | Os12g0287300 | |  | | Up- | |
| OS12T0288400-01 |  | OS12g0288400 | | Os12g0288400 | |  | | - | |
| OS12T0288600-01 |  | OS12g0288600 | | Os12g0288600 | |  | | - | |
| OS12T0290150-00 |  | OS12g0290150 | | Os12g0290150 | |  | | - | |
| OS12T0290600-01 |  | OS12g0290600 | | Os12g0290600 | |  | | - | |
| OS12T0291100-01 |  | OS12g0291100 | | Os12g0291100 | | Down | | -Down | |
| OS12T0297500-01 |  | OS12g0297500 | | Os12g0297500 | |  | | - | |
| OS12T0298600-00 |  | OS12g0298600 | | Os12g0298600 | |  | | - | |
| OS12T0403800-02 |  | OS12g0403800 | | Os12g0403800 | |  | | - | |
| OS12T0405100-01 |  | OS12g0405100 | | Os12g0405100 | |  | | - | |
| OS12T0405200-01 |  | OS12g0405200 | | Os12g0405200 | |  | | - | |
| OS12T0405700-02 |  | OS12g0405700 | | Os12g0405700 | |  | | - | |
| OS12T0406200-01 |  | OS12g0406200 | | Os12g0406200 | |  | | - | |
| OS12T0407500-01 |  | OS12g0407500 | | Os12g0407500 | |  | | - | |
| OS12T0409000-01 |  | OS12g0409000 | | Os12g0409000 | |  | | - | |
| OS12T0420200-01 |  | OS12g0420200 | | Os12g0420200 | |  | | - | |
| OS12T0420400-02 |  | OS12g0420400 | | Os12g0420400 | |  | | - | |
| OS12T0428000-01 |  | OS12g0428000 | | Os12g0428000 | |  | | - | |
| OS12T0428600-01 |  | OS12g0428600 | | Os12g0428600 | |  | | - | |
| OS12T0429200-01 |  | OS12g0429200 | | Os12g0429200 | |  | | - | |
| OS12T0430000-00 |  | OS12g0430000 | | Os12g0430000 | |  | | - | |
| OS12T0434400-00 |  | OS12g0434400 | | Os12g0434400 | |  | | - | |
| OS12T0443700-02 |  | OS12g0443700 | | Os12g0443700 | |  | | - | |
| OS12T0456200-01 |  | OS12g0456200 | | Os12g0456200 | |  | | - | |
| OS12T0465700-01 |  | OS12g0465700 | | Os12g0465700 | |  | | - | |
| OS12T0468600-03 |  | OS12g0468600 | | Os12g0468600 | |  | | - | |
| OS12T0478200-02 |  | OS12g0478200 | | Os12g0478200 | |  | | - | |
| OS12T0479900-01 |  | OS12g0479900 | | Os12g0479900 | |  | | - | |
| OS12T0481100-01 |  | OS12g0481100 | | Os12g0481100 | |  | | - | |
| OS12T0485000-02 |  | OS12g0485000 | | Os12g0485000 | |  | | - | |
| OS12T0485400-01 |  | OS12g0485400 | | Os12g0485400 | |  | | - | |
| OS12T0485800-01 |  | OS12g0485800 | | Os12g0485800 | |  | | - | |
| OS12T0488800-01 |  | OS12g0488800 | | Os12g0488800 | |  | | - | |
| OS12T0489100-01 |  | OS12g0489100 | | Os12g0489100 | |  | | - | |
| OS12T0496900-01 |  | OS12g0496900 | | Os12g0496900 | |  | | - | |
| OS12T0498500-01 |  | OS12g0498500 | | Os12g0498500 | |  | | - | |
| OS12T0498800-01 |  | OS12g0498800 | | Os12g0498800 | |  | | - | |
| OS12T0502000-01 |  | OS12g0502000 | | Os12g0502000 | |  | | - | |
| OS12T0502200-01 | Down | OS12g0502200 | | Os12g0502200 | |  | | Down- | |
| OS12T0502500-01 |  | OS12g0502500 | | Os12g0502500 | |  | | - | |
| OS12T0507200-01 |  | OS12g0507200 | | Os12g0507200 | |  | | - | |
| OS12T0507300-00 |  | OS12g0507300 | | Os12g0507300 | |  | | - | |
| OS12T0507600-01 |  | OS12g0507600 | | Os12g0507600 | |  | | - | |
| OS12T0508300-01 |  | OS12g0508300 | | Os12g0508300 | |  | | - | |
| OS12T0514000-01 |  | OS12g0514000 | | Os12g0514000 | |  | | - | |
| OS12T0514400-01 |  | OS12g0514400 | | Os12g0514400 | |  | | - | |
| OS12T0514500-01 |  | OS12g0514500 | | Os12g0514500 | |  | | - | |
| OS12T0514900-01 |  | OS12g0514900 | | Os12g0514900 | |  | | - | |
| OS12T0517200-01 |  | OS12g0517200 | | Os12g0517200 | |  | | - | |
| OS12T0520200-01 |  | OS12g0520200 | | Os12g0520200 | |  | | - | |
| OS12T0527800-01 |  | OS12g0527800 | | Os12g0527800 | |  | | - | |
| OS12T0529400-01 |  | OS12g0529400 | | Os12g0529400 | |  | | - | |
| OS12T0533700-01 |  | OS12g0533700 | | Os12g0533700 | |  | | - | |
| OS12T0534100-01 |  | OS12g0534100 | | Os12g0534100 | |  | | - | |
| OS12T0534200-01 |  | OS12g0534200 | | Os12g0534200 | | Down | | -Down | |
| OS12T0535300-00 |  | OS12g0535300 | | Os12g0535300 | |  | | - | |
| OS12T0535900-02 |  | OS12g0535900 | | Os12g0535900 | |  | | - | |
| OS12T0538300-01 |  | OS12g0538300 | | Os12g0538300 | |  | | - | |
| OS12T0538900-01 |  | OS12g0538900 | | Os12g0538900 | |  | | - | |
| OS12T0540700-01 |  | OS12g0540700 | | Os12g0540700 | |  | | - | |
| OS12T0540900-02 |  | OS12g0540900 | | Os12g0540900 | |  | | - | |
| OS12T0541000-01 |  | OS12g0541000 | | Os12g0541000 | |  | | - | |
| OS12T0541400-02 |  | OS12g0541400 | | Os12g0541400 | |  | | - | |
| OS12T0541500-02 | Down | OS12g0541500 | | Os12g0541500 | | Down | | Down-Down | |
| OS12T0541700-01 |  | OS12g0541700 | | Os12g0541700 | |  | | - | |
| OS12T0543600-00 |  | OS12g0543600 | | Os12g0543600 | |  | | - | |
| OS12T0548200-02 |  | OS12g0548200 | | Os12g0548200 | |  | | - | |
| OS12T0548300-03 |  | OS12g0548300 | | Os12g0548300 | | Down | | -Down | |
| OS12T0552500-01 |  | OS12g0552500 | | Os12g0552500 | |  | | - | |
| OS12T0555000-01 | Up | OS12g0555000 | | Os12g0555000 | |  | | Up- | |
| OS12T0555200-01 | Up | OS12g0555200 | | Os12g0555200 | |  | | Up- | |
| OS12T0555500-01 | Up | OS12g0555500 | | Os12g0555500 | |  | | Up- | |
| OS12T0556400-02 |  | OS12g0556400 | | Os12g0556400 | |  | | - | |
| OS12T0556600-02 |  | OS12g0556600 | | Os12g0556600 | |  | | - | |
| OS12T0557400-02 |  | OS12g0557400 | | Os12g0557400 | |  | | - | |
| OS12T0559200-01 |  | OS12g0559200 | | Os12g0559200 | |  | | - | |
| OS12T0560300-01 |  | OS12g0560300 | | Os12g0560300 | |  | | - | |
| OS12T0560400-01 |  | OS12g0560400 | | Os12g0560400 | |  | | - | |
| OS12T0562900-01 |  | OS12g0562900 | | Os12g0562900 | |  | | - | |
| OS12T0563600-03 |  | OS12g0563600 | | Os12g0563600 | |  | | - | |
| OS12T0564400-01 |  | OS12g0564400 | | Os12g0564400 | |  | | - | |
| OS12T0564600-01 |  | OS12g0564600 | | Os12g0564600 | |  | | - | |
| OS12T0566200-01 |  | OS12g0566200 | | Os12g0566200 | |  | | - | |
| OS12T0566300-01 |  | OS12g0566300 | | Os12g0566300 | |  | | - | |
| OS12T0566700-02 |  | OS12g0566700 | | Os12g0566700 | |  | | - | |
| OS12T0567700-01 |  | OS12g0567700 | | Os12g0567700 | |  | | - | |
| OS12T0568800-01 |  | OS12g0568800 | | Os12g0568800 | |  | | - | |
| OS12T0569300-01 |  | OS12g0569300 | | Os12g0569300 | | Up | | -Up | |
| OS12T0569500-01 |  | OS12g0569500 | | Os12g0569500 | | Down | | -Down | |
| OS12T0571200-01 |  | OS12g0571200 | | Os12g0571200 | |  | | - | |
| OS12T0571900-01 |  | OS12g0571900 | | Os12g0571900 | |  | | - | |
| OS12T0573200-01 |  | OS12g0573200 | | Os12g0573200 | |  | | - | |
| OS12T0574800-03 |  | OS12g0574800 | | Os12g0574800 | |  | | - | |
| OS12T0575000-01 |  | OS12g0575000 | | Os12g0575000 | |  | | - | |
| OS12T0575200-02 |  | OS12g0575200 | | Os12g0575200 | |  | | - | |
| OS12T0576600-02 | Up | OS12g0576600 | | Os12g0576600 | | Up | | Up-Up | |
| OS12T0578400-01 |  | OS12g0578400 | | Os12g0578400 | | Down | | -Down | |
| OS12T0582800-01 |  | OS12g0582800 | | Os12g0582800 | |  | | - | |
| OS12T0583300-01 | Up | OS12g0583300 | | Os12g0583300 | | Down | | Up-Down | |
| OS12T0583400-01 |  | OS12g0583400 | | Os12g0583400 | |  | | - | |
| OS12T0583500-02 |  | OS12g0583500 | | Os12g0583500 | |  | | - | |
| OS12T0583900-03 |  | OS12g0583900 | | Os12g0583900 | |  | | - | |
| OS12T0586400-00 |  | OS12g0586400 | | Os12g0586400 | |  | | - | |
| OS12T0586600-02 |  | OS12g0586600 | | Os12g0586600 | |  | | - | |
| OS12T0589100-01 |  | OS12g0589100 | | Os12g0589100 | |  | | - | |
| OS12T0596800-01 |  | OS12g0596800 | | Os12g0596800 | |  | | - | |
| OS12T0597300-01 |  | OS12g0597300 | | Os12g0597300 | |  | | - | |
| OS12T0597400-01 | Down | OS12g0597400 | | Os12g0597400 | | Down | | Down-Down | |
| OS12T0597500-02 |  | OS12g0597500 | | Os12g0597500 | |  | | - | |
| OS12T0600400-01 |  | OS12g0600400 | | Os12g0600400 | |  | | - | |
| OS12T0601200-01 |  | OS12g0601200 | | Os12g0601200 | |  | | - | |
| OS12T0604800-01 |  | OS12g0604800 | | Os12g0604800 | |  | | - | |
| OS12T0605300-01 |  | OS12g0605300 | | Os12g0605300 | |  | | - | |
| OS12T0605400-01 |  | OS12g0605400 | | Os12g0605400 | |  | | - | |
| OS12T0605800-01 |  | OS12g0605800 | | Os12g0605800 | |  | | - | |
| OS12T0607000-02 |  | OS12g0607000 | | Os12g0607000 | | Down | | -Down | |
| OS12T0607100-01 |  | OS12g0607100 | | Os12g0607100 | |  | | - | |
| OS12T0608600-02 |  | OS12g0608600 | | Os12g0608600 | |  | | - | |
| OS12T0609500-01 |  | OS12g0609500 | | Os12g0609500 | |  | | - | |
| OS12T0611200-01 |  | OS12g0611200 | | Os12g0611200 | |  | | - | |
| OS12T0613500-01 |  | OS12g0613500 | | Os12g0613500 | |  | | - | |
| OS12T0615400-01 |  | OS12g0615400 | | Os12g0615400 | |  | | - | |
| OS12T0615800-01 |  | OS12g0615800 | | Os12g0615800 | |  | | - | |
| OS12T0615900-01 |  | OS12g0615900 | | Os12g0615900 | |  | | - | |
| OS12T0616200-01 |  | OS12g0616200 | | Os12g0616200 | |  | | - | |
| OS12T0616600-01 |  | OS12g0616600 | | Os12g0616600 | |  | | - | |
| OS12T0616900-01 |  | OS12g0616900 | | Os12g0616900 | |  | | - | |
| OS12T0617800-01 |  | OS12g0617800 | | Os12g0617800 | |  | | - | |
| OS12T0617900-01 |  | OS12g0617900 | | Os12g0617900 | |  | | - | |
| OS12T0620400-01 |  | OS12g0620400 | | Os12g0620400 | |  | | - | |
| OS12T0623800-01 |  | OS12g0623800 | | Os12g0623800 | |  | | - | |
| OS12T0623900-01 |  | OS12g0623900 | | Os12g0623900 | |  | | - | |
| OS12T0625000-01 | Up | OS12g0625000 | | Os12g0625000 | |  | | Up- | |
| OS12T0628500-01 |  | OS12g0628500 | | Os12g0628500 | |  | | - | |
| OS12T0628600-01 | Up | OS12g0628600 | | Os12g0628600 | | Down | | Up-Down | |
| OS12T0630100-01 |  | OS12g0630100 | | Os12g0630100 | | Down | | -Down | |
| OS12T0630200-01 |  | OS12g0630200 | | Os12g0630200 | | Down | | -Down | |
| OS12T0630500-01 |  | OS12g0630500 | | Os12g0630500 | | Up | | -Up | |
| OS12T0630700-01 |  | OS12g0630700 | | Os12g0630700 | |  | | - | |
| OS12T0631800-03 |  | OS12g0631800 | | Os12g0631800 | |  | | - | |
| OS12T0632000-01 |  | OS12g0632000 | | Os12g0632000 | |  | | - | |
| OS12T0632700-01 |  | OS12g0632700 | | Os12g0632700 | |  | | - | |
| OS12T0637100-02 | Up | OS12g0637100 | | Os12g0637100 | |  | | Up- | |
| OS12T0638700-01 |  | OS12g0638700 | | Os12g0638700 | |  | | - | |
| OS12T0639600-02 |  | OS12g0639600 | | Os12g0639600 | |  | | - | |
| OS12T0640600-01 |  | OS12g0640600 | | Os12g0640600 | |  | | - | |
| OS12T0640800-01 |  | OS12g0640800 | | Os12g0640800 | |  | | - | |
| OS12T0641300-01 |  | OS12g0641300 | | Os12g0641300 | | Up | | -Up | |
| OS12T0641400-01 |  | OS12g0641400 | | Os12g0641400 | |  | | - | |
| Table S7 The Up-Up and Down-Down protein accession | | | | | | | | |  |
| **Protein accession** | | | **Type** | | Protein accession | | Type | |  |
| OS01T0118000-01 | | | Up-Up | | OS01T0111100-01 | | Down-Down | |  |
| OS01T0376700-01 | | | Up-Up | | OS01T0180300-01 | | Down-Down | |  |
| OS01T0667200-02 | | | Up-Up | | OS01T0611000-02 | | Down-Down | |  |
| OS01T0800500-01 | | | Up-Up | | OS01T0749200-01 | | Down-Down | |  |
| OS01T0897200-04 | | | Up-Up | | OS01T0805300-02 | | Down-Down | |  |
| OS01T0954000-01 | | | Up-Up | | OS01T0894700-01 | | Down-Down | |  |
| OS01T0970700-02 | | | Up-Up | | OS02T0168800-01 | | Down-Down | |  |
| OS02T0717500-01 | | | Up-Up | | OS02T0240300-01 | | Down-Down | |  |
| OS02T0823100-01 | | | Up-Up | | OS02T0257300-00 | | Down-Down | |  |
| OS03T0248600-02 | | | Up-Up | | OS02T0259600-01 | | Down-Down | |  |
| OS03T0758100-01 | | | Up-Up | | OS02T0553200-01 | | Down-Down | |  |
| OS04T0186400-02 | | | Up-Up | | OS02T0581100-01 | | Down-Down | |  |
| OS04T0434800-02 | | | Up-Up | | OS02T0677600-01 | | Down-Down | |  |
| OS04T0456700-01 | | | Up-Up | | OS02T0754300-01 | | Down-Down | |  |
| OS04T0486950-01 | | | Up-Up | | OS02T0822600-01 | | Down-Down | |  |
| OS04T0652700-01 | | | Up-Up | | OS03T0122200-01 | | Down-Down | |  |
| OS04T0683700-01 | | | Up-Up | | OS03T0125000-02 | | Down-Down | |  |
| OS05T0323800-01 | | | Up-Up | | OS03T0219900-01 | | Down-Down | |  |
| OS06T0593800-01 | | | Up-Up | | OS03T0265400-02 | | Down-Down | |  |
| OS06T0668200-01 | | | Up-Up | | OS03T0749500-01 | | Down-Down | |  |
| OS07T0503300-02 | | | Up-Up | | OS03T0751400-01 | | Down-Down | |  |
| OS07T0582400-01 | | | Up-Up | | OS03T0758900-01 | | Down-Down | |  |
| OS07T0586100-01 | | | Up-Up | | OS03T0811100-01 | | Down-Down | |  |
| OS07T0665200-01 | | | Up-Up | | OS03T0828100-02 | | Down-Down | |  |
| OS07T0679300-01 | | | Up-Up | | OS03T0843400-01 | | Down-Down | |  |
| OS08T0434100-01 | | | Up-Up | | OS03T0844900-01 | | Down-Down | |  |
| OS09T0327400-01 | | | Up-Up | | OS03T0856500-02 | | Down-Down | |  |
| OS09T0442300-01 | | | Up-Up | | OS04T0505200-03 | | Down-Down | |  |
| OS09T0478300-01 | | | Up-Up | | OS04T0591000-01 | | Down-Down | |  |
| OS09T0530300-01 | | | Up-Up | | OS04T0615700-01 | | Down-Down | |  |
| OS10T0178500-01 | | | Up-Up | | OS04T0691600-02 | | Down-Down | |  |
| OS10T0463800-01 | | | Up-Up | | OS05T0103200-02 | | Down-Down | |  |
| OS11T0216300-01 | | | Up-Up | | OS05T0291700-03 | | Down-Down | |  |
| OS11T0658800-01 | | | Up-Up | | OS05T0413200-01 | | Down-Down | |  |
| OS12T0106000-01 | | | Up-Up | | OS06T0136600-01 | | Down-Down | |  |
| OS12T0198000-01 | | | Up-Up | | OS07T0565600-02 | | Down-Down | |  |
| OS12T0226700-01 | | | Up-Up | | OS08T0276100-01 | | Down-Down | |  |
| OS12T0576600-02 | | | Up-Up | | OS08T0504500-01 | | Down-Down | |  |
|  | | |  | | OS08T0509100-01 | | Down-Down | |  |
|  | | |  | | OS09T0279500-03 | | Down-Down | |  |
|  | | |  | | OS09T0294000-02 | | Down-Down | |  |
|  | | |  | | OS09T0439500-01 | | Down-Down | |  |
|  | | |  | | OS09T0565200-02 | | Down-Down | |  |
|  | | |  | | OS10T0100300-01 | | Down-Down | |  |
|  | | |  | | OS11T0544800-01 | | Down-Down | |  |
|  | | |  | | OS12T0541500-02 | | Down-Down | |  |
|  | | |  | | OS12T0597400-01 | | Down-Down | |  |

| Table S8 The process and P value of Go， protein domain and KEGG pathway | | | | | | | | | |
| --- | --- | --- | --- | --- | --- | --- | --- | --- | --- |
| GO Terms Level 1 | | | | | | | Fisher's exact Pvalue | |  |
| Molecular Function | | | | | | | 0.000560072 | |  |
| Molecular Function | | | | | | | 0.000560072 | |  |
| Molecular Function | | | | | | | 0.000560072 | |  |
| Molecular Function | | | | | | | 0.000763654 | |  |
| Molecular Function | | | | | | | 0.000234717 | |  |
| Molecular Function | | | | | | | 0.001924111 | |  |
| Molecular Function | | | | | | | 0.003257866 | |  |
| Molecular Function | | | | | | | 0.000103377 | |  |
| Molecular Function | | | | | | | 0.000255327 | |  |
| Molecular Function | | | | | | | 0.000819183 | |  |
| Biological Process | | | | | | | 7.56E-05 | |  |
| Biological Process | | | | | | | 0.003736196 | |  |
| Biological Process | | | | | | | 0.041710064 | |  |
| Biological Process | | | | | | | 0.008462269 | |  |
| Biological Process | | | | | | | 0.02018265 | |  |
| Protein domain | | | | | | | p value | |  |
| UbiB domain | | | | | | | 0.001301287 | |  |
| Iron/zinc purple acid phosphatase-like C-terminal ... | | | | | | | 0.001665797 | |  |
| Purple acid phosphatase, N-terminal | | | | | | | 0.002073181 | |  |
| Purple acid phosphatase-like, N-terminal | | | | | | | 0.002073181 | |  |
| KEGG pathway | | | | | | | p value | |  |
| osa00010 | | | | | | | 0.001577863 | |  |
| osa01200 | | | | | | | 0.023965957 | |  |
| osa00630 | | | | | | | 0.052959227 | |  |
| osa00710 | | | | | | | 0.054535979 | |  |
| osa00500 | | | | | | | 0.059368887 | |  |
| osa01230 | | | | | | | 0.06846866 | |  |
|  | | | | | | |  | |  |
| The Table S9 unique peptides of six proteins | | | | | | | |  |  |
| Protein Accession | Peptide | | | | | | |  |  |
| OS01T0800500-01 | AQYLTSDPGYLGCK | | | | | | |  |  |
| OS04T0683700-01 | DAAGQVHLAGFPASAAAAAK，IVAQHFVVPVLPTK | | | | | | |  |  |
| OS06T0668200-01 | LAATLPDGGVLLLENVR | | | | | | |  |  |
| OS07T0586100-01 | SQFVYSNIGGIYR，SVSWDGVHFTEAANR | | | | | | |  |  |
| OS09T0478300-01 | NHQPIVQVLIDGK，SLATCTYELR | | | | | | |  |  |
| OS09T0530300-01 | NITFAPFGEQWR，TSLFVNAWAIGR | | | | | | |  |  |
| Table S10 the D value of each accession | | | | | |  |  |  |  |
| Number | | D |  |  |  |  |  |  |  |
| WR157 | | 0.922281 |  |  |  |  |  |  |  |
| WR411 | | 0.899654 |  |  |  |  |  |  |  |
| WR16 | | 0.895642 |  |  |  |  |  |  |  |
| WR161 | | 0.882338 |  |  |  |  |  |  |  |
| WR163 | | 0.873224 |  |  |  |  |  |  |  |
| WR154 | | 0.861236 |  |  |  |  |  |  |  |
| WR397 | | 0.822645 |  |  |  |  |  |  |  |
| WR171 | | 0.81662 |  |  |  |  |  |  |  |
| IAPAR-9 | | 0.810674 |  |  |  |  |  |  |  |
| WR412 | | 0.798298 |  |  |  |  |  |  |  |
| WR7 | | 0.796381 |  |  |  |  |  |  |  |
| WR153 | | 0.795973 |  |  |  |  |  |  |  |
| WR166 | | 0.787402 |  |  |  |  |  |  |  |
| WR162 | | 0.768152 |  |  |  |  |  |  |  |
| WR164 | | 0.757906 |  |  |  |  |  |  |  |
| WR144 | | 0.756366 |  |  |  |  |  |  |  |
| WR100 | | 0.746812 |  |  |  |  |  |  |  |
| WR404 | | 0.745584 |  |  |  |  |  |  |  |
| WR165 | | 0.744138 |  |  |  |  |  |  |  |
| WR408 | | 0.737508 |  |  |  |  |  |  |  |
| WR155 | | 0.734303 |  |  |  |  |  |  |  |
| WR148 | | 0.725429 |  |  |  |  |  |  |  |
| WR167 | | 0.724474 |  |  |  |  |  |  |  |
| WR158 | | 0.718781 |  |  |  |  |  |  |  |
| WR102 | | 0.717509 |  |  |  |  |  |  |  |
| WR410 | | 0.716688 |  |  |  |  |  |  |  |
| WR394 | | 0.716575 |  |  |  |  |  |  |  |
| WR399 | | 0.715053 |  |  |  |  |  |  |  |
| WR304 | | 0.714975 |  |  |  |  |  |  |  |
| WR156 | | 0.711996 |  |  |  |  |  |  |  |
| WR101 | | 0.703915 |  |  |  |  |  |  |  |
| WR421 | | 0.702496 |  |  |  |  |  |  |  |
| WR398 | | 0.699454 |  |  |  |  |  |  |  |
| WR152 | | 0.698112 |  |  |  |  |  |  |  |
| WR417 | | 0.692837 |  |  |  |  |  |  |  |
| WR3 | | 0.690779 |  |  |  |  |  |  |  |
| WR422 | | 0.689572 |  |  |  |  |  |  |  |
| WR393 | | 0.686373 |  |  |  |  |  |  |  |
| WR301 | | 0.684683 |  |  |  |  |  |  |  |
| WR413 | | 0.683135 |  |  |  |  |  |  |  |
| WR403 | | 0.677281 |  |  |  |  |  |  |  |
| WR419 | | 0.67718 |  |  |  |  |  |  |  |
| WR170 | | 0.670358 |  |  |  |  |  |  |  |
| WR150 | | 0.665237 |  |  |  |  |  |  |  |
| WR151 | | 0.664431 |  |  |  |  |  |  |  |
| WR405 | | 0.663829 |  |  |  |  |  |  |  |
| WR5 | | 0.662003 |  |  |  |  |  |  |  |
| WR145 | | 0.654853 |  |  |  |  |  |  |  |
| WR94 | | 0.650999 |  |  |  |  |  |  |  |
| WR97 | | 0.648934 |  |  |  |  |  |  |  |
| WR6 | | 0.643651 |  |  |  |  |  |  |  |
| WR13 | | 0.641457 |  |  |  |  |  |  |  |
| WR251 | | 0.636288 |  |  |  |  |  |  |  |
| WR243 | | 0.63597 |  |  |  |  |  |  |  |
| WR98 | | 0.634521 |  |  |  |  |  |  |  |
| WR18 | | 0.629159 |  |  |  |  |  |  |  |
| WR252 | | 0.625318 |  |  |  |  |  |  |  |
| WR244 | | 0.623031 |  |  |  |  |  |  |  |
| WR416 | | 0.619217 |  |  |  |  |  |  |  |
| WR96 | | 0.612281 |  |  |  |  |  |  |  |
| WR254 | | 0.608697 |  |  |  |  |  |  |  |
| WR30 | | 0.608481 |  |  |  |  |  |  |  |
| WR10 | | 0.605598 |  |  |  |  |  |  |  |
| WR9 | | 0.604805 |  |  |  |  |  |  |  |
| WR2 | | 0.603823 |  |  |  |  |  |  |  |
| WR99 | | 0.598909 |  |  |  |  |  |  |  |
| WR33 | | 0.593237 |  |  |  |  |  |  |  |
| WR12 | | 0.589285 |  |  |  |  |  |  |  |
| WR234 | | 0.58589 |  |  |  |  |  |  |  |
| WR242 | | 0.585605 |  |  |  |  |  |  |  |
| WR146 | | 0.582016 |  |  |  |  |  |  |  |
| WR418 | | 0.580676 |  |  |  |  |  |  |  |
| WR246 | | 0.57468 |  |  |  |  |  |  |  |
| WR31 | | 0.574155 |  |  |  |  |  |  |  |
| WR241 | | 0.573078 |  |  |  |  |  |  |  |
| WR168 | | 0.57205 |  |  |  |  |  |  |  |
| WR8 | | 0.570168 |  |  |  |  |  |  |  |
| WR395 | | 0.566674 |  |  |  |  |  |  |  |
| WR15 | | 0.564414 |  |  |  |  |  |  |  |
| WR21 | | 0.562326 |  |  |  |  |  |  |  |
| WR235 | | 0.554114 |  |  |  |  |  |  |  |
| WR299 | | 0.552457 |  |  |  |  |  |  |  |
| WR169 | | 0.550408 |  |  |  |  |  |  |  |
| WR250 | | 0.550051 |  |  |  |  |  |  |  |
| WR32 | | 0.548449 |  |  |  |  |  |  |  |
| WR159 | | 0.542343 |  |  |  |  |  |  |  |
| WR27 | | 0.541627 |  |  |  |  |  |  |  |
| WR396 | | 0.538495 |  |  |  |  |  |  |  |
| WR17 | | 0.533819 |  |  |  |  |  |  |  |
| WR409 | | 0.532291 |  |  |  |  |  |  |  |
| WR253 | | 0.530704 |  |  |  |  |  |  |  |
| WR95 | | 0.529471 |  |  |  |  |  |  |  |
| WR415 | | 0.526963 |  |  |  |  |  |  |  |
| WR247 | | 0.525043 |  |  |  |  |  |  |  |
| WR248 | | 0.519571 |  |  |  |  |  |  |  |
| WR19 | | 0.507211 |  |  |  |  |  |  |  |
| WR143 | | 0.506226 |  |  |  |  |  |  |  |
| WR240 | | 0.504168 |  |  |  |  |  |  |  |
| WR28 | | 0.503343 |  |  |  |  |  |  |  |
| WR172 | | 0.502637 |  |  |  |  |  |  |  |
| WR255 | | 0.498801 |  |  |  |  |  |  |  |
| WR238 | | 0.494564 |  |  |  |  |  |  |  |
| WR25 | | 0.487508 |  |  |  |  |  |  |  |
| WR239 | | 0.483698 |  |  |  |  |  |  |  |
| WR149 | | 0.465975 |  |  |  |  |  |  |  |
| WR298 | | 0.460371 |  |  |  |  |  |  |  |
| WR406 | | 0.459957 |  |  |  |  |  |  |  |
| WR236 | | 0.459911 |  |  |  |  |  |  |  |
| WR26 | | 0.459891 |  |  |  |  |  |  |  |
| WR249 | | 0.451766 |  |  |  |  |  |  |  |
| WR407 | | 0.451635 |  |  |  |  |  |  |  |
| WR24 | | 0.43709 |  |  |  |  |  |  |  |
| WR160 | | 0.435674 |  |  |  |  |  |  |  |
| WR147 | | 0.433513 |  |  |  |  |  |  |  |
| WR303 | | 0.41522 |  |  |  |  |  |  |  |
| WR20 | | 0.413515 |  |  |  |  |  |  |  |
| WR29 | | 0.413123 |  |  |  |  |  |  |  |
| WR306 | | 0.411084 |  |  |  |  |  |  |  |
| WR307 | | 0.410623 |  |  |  |  |  |  |  |
| WR245 | | 0.408609 |  |  |  |  |  |  |  |
| WR305 | | 0.407709 |  |  |  |  |  |  |  |
| WR302 | | 0.407383 |  |  |  |  |  |  |  |
| WR420 | | 0.404736 |  |  |  |  |  |  |  |
| WR233 | | 0.39561 |  |  |  |  |  |  |  |
| WR237 | | 0.389182 |  |  |  |  |  |  |  |
| WR402 | | 0.388651 |  |  |  |  |  |  |  |
| WR401 | | 0.381155 |  |  |  |  |  |  |  |
| WR400 | | 0.372566 |  |  |  |  |  |  |  |
| WR414 | | 0.333728 |  |  |  |  |  |  |  |
| WR11 | | 0.326906 |  |  |  |  |  |  |  |
| WR300 | | 0.306742 |  |  |  |  |  |  |  |
| WR14 | | 0.27009 |  |  |  |  |  |  |  |
| WR23 | | 0.268656 |  |  |  |  |  |  |  |
| WR22 | | 0.15139 |  |  |  |  |  |  |  |
| Annotation: Strong tlerance: D > 0.8 | | | |  |  |  |  |  |  |
| Tolerant: 0.8 > D > 0.5 | | |  |  |  |  |  |  |  |
| Moderate: 0.5 > D> 0.4 | | |  |  |  |  |  |  |  |
| Relatively sensitive: 0.4 > D > 0.2 | | | |  |  |  |  |  |  |
| Sensitive: D < 0.2 | | |  |  |  |  |  |  |  |
